# Nucleotide-level prediction of CircRNA-protein binding based on fully convolutional neural network

**DOI:** 10.3389/fgene.2023.1283404

**Published:** 2023-10-06

**Authors:** Zhen Shen, Wei Liu, ShuJun Zhao, QinHu Zhang, SiGuo Wang, Lin Yuan

**Affiliations:** ^1^ School of Computer and Software, Nanyang Institute of Technology, Nanyang, Henan, China; ^2^ EIT Institute for Advanced Study, Ningbo, Zhejiang, China; ^3^ Key Laboratory of Computing Power Network and Information Security, Ministry of Education, Shandong Computer Science Center, Qilu University of Technology (Shandong Academy of Sciences), Jinan, China; ^4^ Shandong Engineering Research Center of Big Data Applied Technology, Faculty of Computer Science and Technology, Qilu University of Technology (Shandong Academy of Sciences), Jinan, China; ^5^ Shandong Provincial Key Laboratory of Computer Networks, Shandong Fundamental Research Center for Computer Science, Jinan, China

**Keywords:** CircRNA-protein binding sites prediction, deep learning, fully convolutional neural networks, hard negative mining loss, nucleotide-level prediction

## Abstract

**Introduction:** CircRNA-protein binding plays a critical role in complex biological activity and disease. Various deep learning-based algorithms have been proposed to identify CircRNA-protein binding sites. These methods predict whether the CircRNA sequence includes protein binding sites from the sequence level, and primarily concentrate on analysing the sequence specificity of CircRNA-protein binding. For model performance, these methods are unsatisfactory in accurately predicting motif sites that have special functions in gene expression.

**Methods:** In this study, based on the deep learning models that implement pixel-level binary classification prediction in computer vision, we viewed the CircRNA-protein binding sites prediction as a nucleotide-level binary classification task, and use a fully convolutional neural networks to identify CircRNA-protein binding motif sites (CPBFCN).

**Results:** CPBFCN provides a new path to predict CircRNA motifs. Based on the MEME tool, the existing CircRNA-related and protein-related database, we analysed the motif functions discovered by CPBFCN. We also investigated the correlation between CircRNA sponge and motif distribution. Furthermore, by comparing the motif distribution with different input sequence lengths, we found that some motifs in the flanking sequences of CircRNA-protein binding region may contribute to CircRNA-protein binding.

**Conclusion:** This study contributes to identify circRNA-protein binding and provides help in understanding the role of circRNA-protein binding in gene expression regulation.

## 1 Introduction

Circular RNAs (CircRNAs) are special “noncoding” RNAs with a circular closed loop structure (Kristensen et al., 2019; Liu and Chen, 2022). Previous studies suggest that CircRNAs have greater biological stability compare other biomolecules, and directly or indirectly participates in gene expression regulation through the functional sites in CircRNA sequence. CircRNA-protein binding is a significant factor in gene expression regulation (Li et al., 2018a; Zang et al., 2020; Su et al., 2022a; Yang et al., 2022a; Su et al., 2022b; Wang et al., 2022). Therefore, CircRNA-RBP binding sites prediction is always the emphasis of CircRNA research. Biological experimental technology was first proposed to identify CircRNA-protein binding sites (Licatalosi et al., 2008; Hafner et al., 2010; König et al., 2010; Barnes and Kanhere, 2016; Gagliardi and Matarazzo, 2016). Despite the drawback of time-consuming and cost-heavy, these methods provide a wealth of dependable data relating CircRNA-protein binding sites. In the beginning, the statistical properties, such as k-mer frequency and secondary structure elements, were employed to represent RNA sequence (Zhang et al., 2011; Chen et al., 2014). And then, several conventional computing models based on statistical methods or machine learning methods were proposed to identify CircRNA-protein binding (Kumar et al., 2008; Liu et al., 2010; Li et al., 2017a; Li et al., 2017b). These methods are more suitable for biological research in terms of time, cost, and accuracy than biological experimental.

In conventional computing models, the hand-crafted features not only rely on the experience of researchers but also are difficult to optimize and easy to lose important features. When dealing with massive biological data, these methods still have a lot of room for improvement in time complexity, noise sensitivity, etc. With the improvement of GPU (Graphics Processing Unit) performance, deep learning models, such as CNN (Convolutional Neural Network), LSTM (Long Short-Term Memory), attention, and transformer, have become widely used in bioinformatics (Zhou and Troyanskaya, 2015; Quang and Xie, 2016; Abbasi et al., 2020; Le et al., 2021). The first model to predict protein binding sites in DNA/RNA using CNN was DeepBind (Alipanahi et al., 2015). Subsequently, an increasing number of deep learning models have been proposed for predicting protein binding sites (Shen et al., 2019), non-coding variants (Zhou and Troyanskaya, 2015), chromatin accessibility (Li et al., 2019), protein post-translational modification (Wang et al., 2019a), gene expression (Singh et al., 2016), etc. In CircRNA-protein binding prediction, multi-feature learning methods are commonly used to encode RNA sequence data with complex model structures (Jia et al., 2020; Wang and Lei, 2021; Yuan and Yang, 2021; Yang et al., 2022b; Li et al., 2022; Niu et al., 2022; Yu et al., 2022; Cao et al., 2023; Zhang et al., 2023). Wang et al. utilised the one-hot encoding method and CNN to predict cancer-specific CircRNA-protein binding sites (Wang et al., 2019b). Zhang et al. proposes CRIP to predict CircRNA-protein binding sites by using the codon encoding method and CNN-LSTM neural network (Zhang et al., 2019a). Ju et al. first split the RNA sequence into a 10-mer sequence, and use Glove to encode the 10-mer sequence, and then use CNN, bidirectional LSTM and CRF (Conditional Random Field) to extract features and predict motif sites (Ju et al., 2019). In iCricRBP-DHN, CircRNA sequence is presented via concatenation of encoded data using a K-tuple nucleotide frequency pattern and CircRNA2Vec. To facilitate feature learning, this method deploys deep multi-scale ResNet, bidirectional GRUs (Gate Recurrent Units), a self-attention mechanism to extract features (Yang et al., 2021).

Both machine-learning and deep learning methods consider predicting CircRNA-protein binding sites as a binary classification problem. Hence, the important concern is to select a negative sequence. Commonly used methods include selection from the upstream and downstream of protein binding sites, random generation, and search from the whole genome. However, there remains doubts concerning whether the sequences produced by these methods really meet the criteria of negative sequences. On the other hand, existing models extract various features from RNA sequences for prediction and achieve better performance. These methods make predictions at the sequence level, largely concentrating on the sequence specificity of protein binding sites. No attempt was made to identify motif sites at the nucleotide-level.

In computer vision, FCN (Fully Convolutional Network) can complete tasks such as image segmentation and image classification at pixel-level. As a result, FCN has been implemented for DNA sequence analysis at nucleotide-level (Wang et al., 2021; Zhang et al., 2021). In this study, we used FCN to predict CircRNA-protein binding motifs, which we call the CPBFCN model. For CPBFCN, it treats motif discovery as a nucleotide-level prediction task and can identify motif sites of various lengths. The known protein binding sites in the CircRNA sequence are considered as positive samples, while other sites are regarded as negative sample. This eliminates negative sequence generation in sequence-level models. For the whole CircRNA sequence, the ratio of motif sites and other sites is unbalanced, hard negative mining loss is used as the loss function to reduce the negative effect of unbalanced data on model performance. CPBFCN provides a new path to predict CircRNA motifs. The trained CPBFCN was used to extract motif from CircRNA sequence. In this study, we not only analyzed the function of motif found by CPBFCN but also their distribution and correlation with CircRNA sponge.

## 2 Materials and methods

### 2.1 Data

To evaluate model performance, 37 CircRNA-protein binding datasets were collected from CRIP and iCricRBP-DHN. Each dataset is used for individual training and testing purposes. We obtained 37 original experimentally validated circRNA-protein binding data from the CircRNA interactome database (https://circinteractome.nia.nih.gov/), which includes over 100,000 human CircRNA sequence information. Each entry in this database contains the location information of protein binding region in CircRNA sequence. To obtain positive samples, we started at the midpoint of protein binding region and extended upstream and downstream by 50-nt, respectively, a 101-nt short sequence is obtained as the positive samples. We also use the same method to generate 201-nt short sequence and 501-nt short sequence as positive samples. The negative sample is obtained by randomly selecting 101-nt/201-nt/501-nt short sequence from the remaining CircRNA sequence. To eliminate the effect of redundant sequences, CD-HIT is used to remove the redundant sequence with a threshold of 0.8. Details about experimental data used in this study are presented in Supplementary Data (see Supplementary Section “Experimental Data Used in This Study—Data Processing”). All three different length experimental datasets are used for hyper-parameter experiments and to evaluate the performance of CPBFCN and three baseline models. The number of sequence record of three different length experimental datasets is shown in Supplementary Table S1. Supplementary Table S2 shows the motif length information in 37 datasets.

CPBFCN is a nucleotide-level prediction model. Each site in the input CircRNA sequence has a label indicating whether the site belongs to the protein binding region. Based on the CircRNA-protein binding sequence data and the binding region information obtained from the circinteractome, we generate an array that is the same length (101, 201, or 501) as the CircRNA-protein binding data. Next, we need to identify the interval position in the array that corresponds to the protein binding region. Thirdly, each element within the interval is set to 1, and other elements in the array is set to 0. Additionally, a sequence-label file is generated for the competing methods. For every dataset, short sequence belongs to the positive sample are labelled with 1, and other sites in CircRNA sequence belongs to the negative sample are labelled with 0.

In this step, the input CircRNA-protein binding data is encoded using one-hot method. Four bases are represented as follows: $A\left(1,0,0,0\right),C\left(0,1,0,0\right),G\left(0,0,1,0\right),U\left(0,0,0,1\right)$ . If there are L records in the input data, and each record’s length is M, the encoded record is converted into a L×M×4 matrix.

### 2.2 Model construction

**FIGURE 1:**
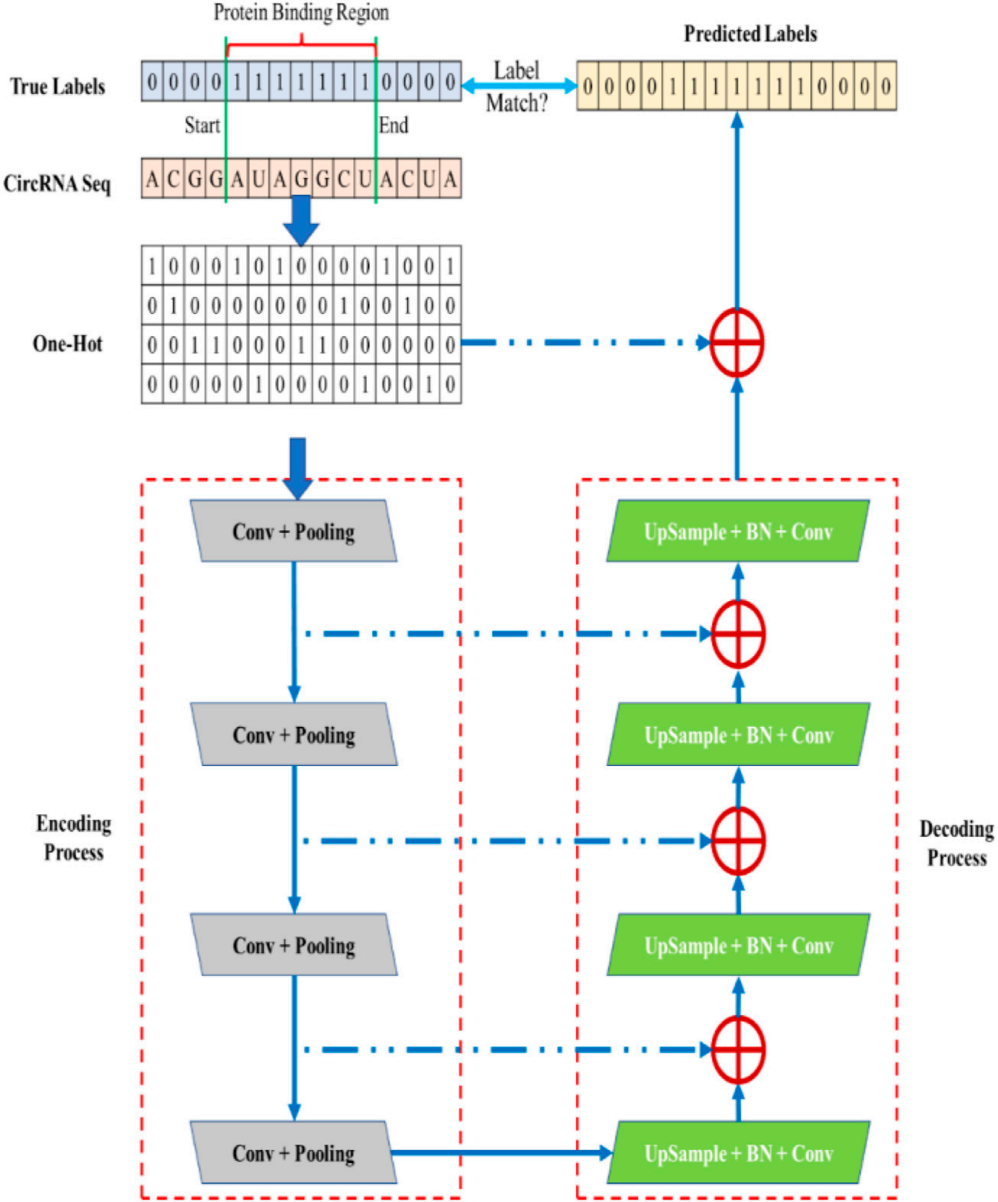
The workflow of CPBFCN.

CPBFCN is a deep learning model based on FCN, and its workflow is shown in Figure 1. CPBFCN involves two chief components: the encoding process and decoding process.

The aim of the encoding process is to extract features and reduce dimensionality. In contrast, the decoding process involves restoring the feature maps generated from the encoding process to the original data size through deconvolution operations. Moreover, the skip line is used to combine the deep semantic information with the shallow appearance features. These two modules are explained in further detail below.

#### 2.2.1 Data encoding process

This process is also known as down sampled, which contains three modules for feature extraction and an average pooling layer. The feature extraction module comprises three parts: a convolutional layer, a max-pooling layer, and a dropout layer. The computational process is shown in Eq. 1.

$$\begin{array}{l} MP\_out=MaxPooling\left(ReLU\left(Conv\left(k*id,b\right)\right)\right)\\ FE\_out=Dropout\left(MP\_out\right) \end{array}$$ (1)

where, *id* is a matrix representing the input data encoded by one-hot, *k* represents the convolutional kernel, and b is a bias term. Here, the convolutional kernel can be seen as a motif scanner, scoring each potential protein binding region in the CircRNA sequence. *MP_out* represents the output of the Max pooling layer, which decreases data dimensionality and chooses features for identifying protein binding regions. *Dropout* can reduce the adverse impact of overfitting on model performance.

Regardless of NLP or CV, numerous researchers have discovered that the context feature is crucial for improving deep learning model performance. To address this issue, various methods have been proposed (Li et al., 2018b; Jain et al., 2020). In genomic analysis, the context feature of motif sites in CircRNA sequences is also significant for RNA-protein binding. Therefore, the impact of the global average pooling on model performance is experimentally demonstrate in the experimental section.

#### 2.2.2 Data decoding process

This process contains four deconvolutional modules, each including four components: an upsample layer, a batch normalization (BN) layer, a ReLU layer, and a convolutional layer. The skip layer is denoted by a blue dashed line and performs a summation operation. The computation process is shown in Eq. 2.

BN_out=BNupsampleEP_out+FE_outRe_out=ReLUBN_outD+eConv=Convkup*Re_out,bup (2)

where, *BN_out* represents the output of BN layer. *FE_out* represents the output of the feature extraction module at the same level as the current deconvolutional module in the data encoding process. EP_out represents the output of the final feature extraction module. *k* ^*up*^ and *b* ^*up*^ represent the convolutional kernel and the bias term, respectively. The purpose of upsample is to restore the size of the output features of the data encoding process to be the same as the input data.

#### 2.2.3 Model loss function

In image segmentation, the objective is to separate the target from other information within the current image. Therefore, the target pixels in the image are considered as positive samples, while the remaining pixels are negative samples. In other words, image segmentation constitutes an imbalanced binary classification task. Conventional loss functions are not suitable to this, which involves imbalanced data. To address this issue, several methods have been proposed. HNM (hard negative mining) is one of the more commonly used methods (Ren et al., 2015). In this study, we apply the HNM-based loss function HNML (hard negative mining loss) proposed by (Zhang et al., 2021) to identify and predict CircRNA-protein binding motifs. The computation process is shown in Eq. 3.

losspos=CrossentropyDeConvpos+lossneg=CrossentropyDeConvneg+lossnegsort=topklossneg,ratio=Vloss=meanlosspos+meanlossnegsort (3)

Where *+DeConv* represents the output of the last deconvolutional module. V represents the value that determined top-k when selecting top-k loss. *Crossentropy* represents using cross entropy function the to calculate the loss value. *mean* represents calculating the average of loss value.

#### 2.2.4 Predicting CircRNA sequence motifs

Unlike the sequence-level prediction models, which can only predict whether a sequence is a bind to a protein, CPBFCN is a nucleotide-level model that can predict whether a nucleotide site binds to a protein. The outputs of CPBFCN require further processing before it can be used for predicting CircRNA motifs. We use the same approach as in CircCNN (Shen et al., 2022) proposed previously to predict motifs. This procedure consists of three steps. Firstly, the task of this step is to locate CircRNA-protein binding region, the same process described above is repeated in this step. Subsequently, the weights and outputs of first convolutional layer in the trained model were used to evaluate the potential motifs in the located regions. The highest-scored potential motif was selected as the predicted motif. Finally, PFMs (Position Frequency Matrixes) are computed by extracting the nucleotide frequency information from all aligned predicted motifs. TOMTOM is used to match the PFMs with known validated protein motifs.

## 3 Results

### 3.1 Experimental setting

In this study, three existing methods were used as baseline models for comparison with CPBFCN: CRIP, circSLNN, and iCircRBP-DHN. The evaluation of CPBFCN’s performance was based on IOU (Intersection over Union). IOU is a commonly used measure in image segmentation, which represents the overlapping ratio of the predicted labels and the true labels. iou_0 represents the ratio of predicted label 0 and true label 0. iou_1 represents the ratio of predicted label 1 and true label 1. miou (mean iou) represents the average of iou_0 and iou_1. Furthermore, this study employed three statistical indicators (*p*-value, e-value and q-value) to evaluate the performance of CPBFCN as compared to three baseline models in predicting motifs. The role of 5-fold cross validation in this study is to make full use of the experiment datasets to evaluate model performance when the experimental datasets are insufficient. In 5-fold cross validation, all data are divided into five parts, in which one of the segments is designated for testing purposes while the remaining segments are used for training.

Considering the effect of model structure, input data, loss function on model performance, four parameters were designed for hyper-parameter testing in this section: input data length change, the ratio value change in HNML, whether to use global average pooling, use BCE (Binary Cross-Entropy) or HNML as loss function. 19 datasets were involved in hyper-parameter experiments. Table 1 shows the different values of four parameters. Hardware platform information is shown in Table 2. The motif name in Ray2013_rbp_Homo_sapiens can be found in Supplementary Table S3. 24 hyper-parameter combinations were displayed in Supplementary Table S4.

**TABLE 1:** Model hyper parameter value.

Parameter	Value
Data Length	101, 201, 501
Loss Function	BCE, HNML
HNML Ratio	0.3, 0.5, 0.7
Pooling	Whether to use global average pooling

**TABLE 2:** Hardware platform information.

Server	DELL T7910
OS	Ubuntu 16.04 LTS
CPU	E5-2680V4 x2
Memory	128G
GPU	NVIDIA 2080Ti

**FIGURE 2:**
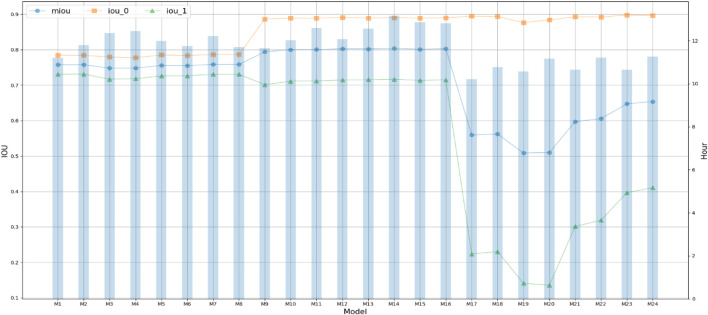
IOU and time comparison of CPBFCN with 24 parameter combination.

Figure 2 shows the performance comparison of CPBFCN across 24 parameter combinations. Supplementary Tables S5–S12 shows the comparisons of miou, iou_0, and iou_1 of CPBFCN on 19 datasets. From Figure 2, it is evident that iou_0 is optimal when the input length is 201 and 501, iou_1 is best when the input length is 101. When the input length is 201, miou is the mean of iou_0 and iou_1 and the decline of iou_1 is not significant, hence miou is optimal compared with other input lengths. Considering that CPBFCN is a nucleotide-level model, our goal is to predict the nucleotide sites labeled 1 in the input data. When the input length increases, the number of positive samples does not change, and more negative samples are introduced. A variation of miou is not indicative of CPBFCN being able to predict the motif sites more accurately. Therefore, we select the best parameters from 8 parameter combinations when the input length is 101. We have included the running times of different models in Figure 2. From Figure 2, we found that model with FCN and average pooling showed no advantage than model using only FCN. After a comprehensive consideration of Figure 1 and Supplementary Tables S4–S11, we opt M7 (101, HNML, 0.7) as the optimal parameter. The subsequent section will evaluate model performance across all 37 datasets and investigate the effect of model structure change on model performance.

### 3.2 CPBFCN performance comparison and analysis

**FIGURE 3:**
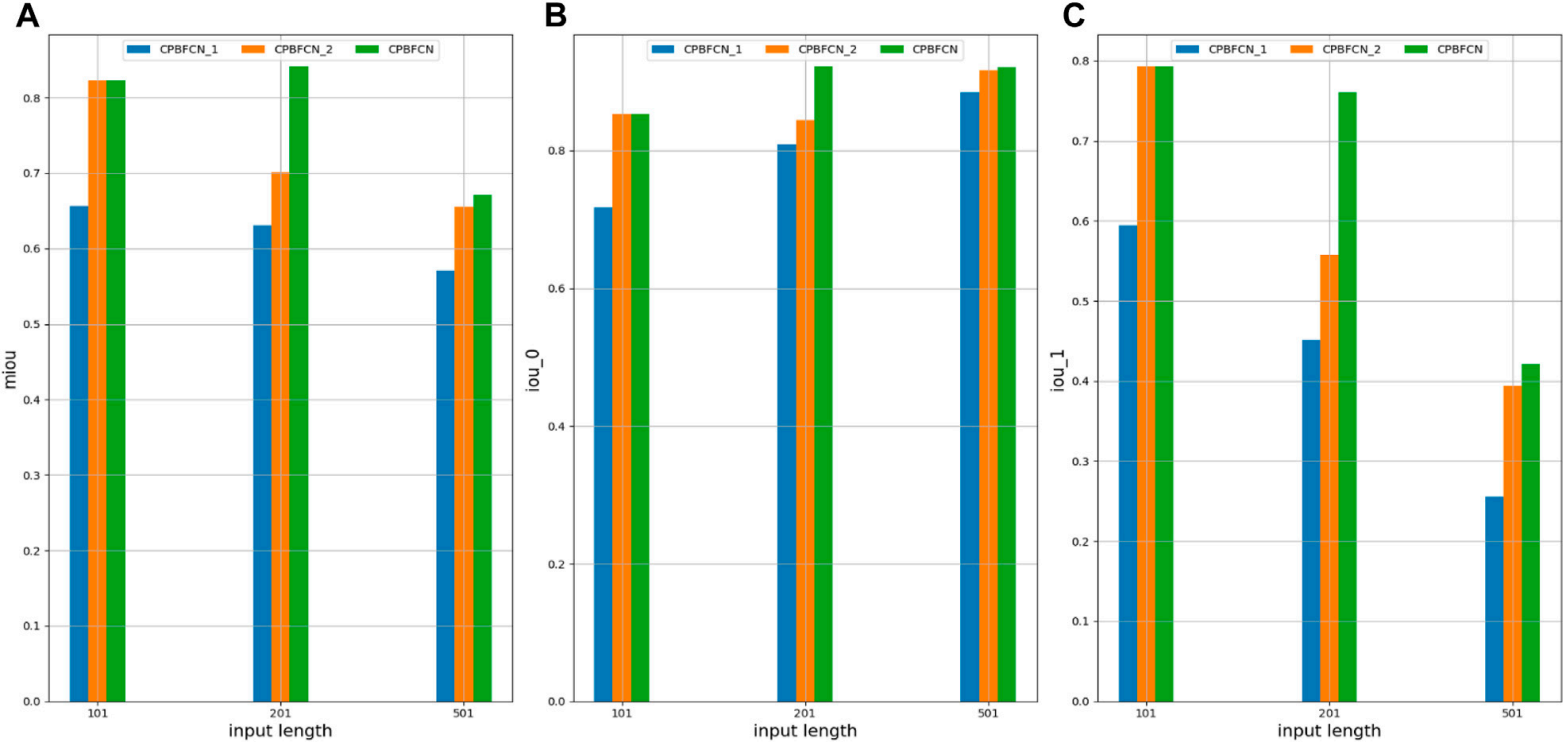
**(A)** miou comparison of CPBFCN_1, CPBFCN_2 and CPBFCN; **(B)** iou_0 comparison of CPBFCN_1, CPBFCN_2 and CPBFCN; **(C)** iou_1 comparison of CPBFCN_1, CPBFCN_2 and CPBFCN.

In this section, model performance was tested with optimal parameters across 37 datasets. Additionally, the influence of structural variations on model performance was assessed through the modification of the encoding and decoding modules within the model. Figure 3 and Supplementary Tables S13–S15 show the performance comparison of CPBFCN and its variations. Here, CPBFCN_1 represents CPBFCN with two encoding modules and two decoding modules, while CPBFCN_2 represents CPBFCN with three encoding modules and three decoding modules. Since the encoding module and the decoding module relate to feature extraction and data recovery respectively, the performance of CPBFCN_1 using only two encoding modules and two decoding modules is least desirable. For both CPBFCN_2 and CPBFCN, there is no significant performance when the input length is 101. However, for the input length is 201 and 501, CPBFCN exhibits a clear advantage. Overall, the performance of CPBFCN is still optimal.

During hyperparameter experimentation, we found that the miou and iou_0 of CPBFCN are optimal with an input length 201, although there is a downward trend in iou_1. In this section, we also found the same phenomenon when testing model performance with 37 datasets. However, there are still some exceptions. As shown in Supplementary Table S16, when the input length is 201, and six datasets EIF4A3, FOX2, IGF2BP1, IGF2BP2, IGF2BP3, and ZC3H7B are used to test model performance, three indicators miou, iou_0 and iou_1 display an upward trend. This suggests that an increased input length could potentially aid in identifying protein binding sites. In the next section, we will further examine this phenomenon by analyzing motif distribution.

### 3.3 Motif analysis

#### 3.3.1 Motif discovery performance analysis

In this section, we first extract motifs from 37 datasets using CPBFCN and three baseline models. Subsequently, we compare known motifs in RNA/Ray2013_rbp_Homo_sapiens and motifs predicted by four models by all four models using TOMTOM. Given that CPBFCN is a nucleotide-level model, we evaluate the performance of all four models using three metrics −log2 (*p*-value), −log2 (q-value), −log2 (e-value).

Supplementary Figure S1 displays the distribution of three metrics for CPBFCN and three baseline models. It is clear from this figure that CPBFCN does not have an advantage. Furthermore, after comparing Supplementary Tables S1, S3, we observed that only ten motifs coexist in the two tables: FUS, FXR1, FXR2, HNRNPC, HUR, IGF2BP2, IGF2BP3, LIN28A, QKI, TIA1. Table 3 shows the discovery performance comparison of CPBFCN and three baseline models in discovering 10 coexisting motifs by scanning the motif discovery data of four models. The comparison reveals that CPBFCN and iCircRBP-DHN can identify 5 motifs, whereas CRIP and circSLNN can identify 4 and 3 motifs respectively. By comparing the *p*-value, e-value, and q-value of CPBFCN and iCircRBP-DHN, it is evident that CPBFCN performs better than iCircRBP-DHN in identifying HUR, IGF2BP3, TIA1.

**TABLE 3:** Performance comparison of four models for 10 coexist motifs.

CPBFCN	CRIP	circSLNN	iCircRBP-DHN
HNRNPC 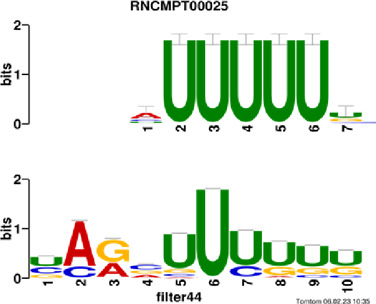 p-value: 1.00e−04	HNRNPC 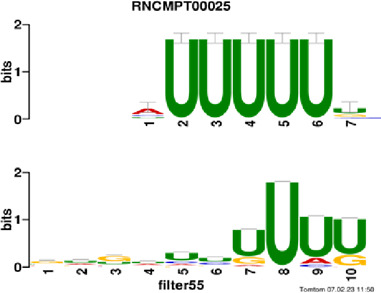 p-value: 1.85e−05	HNRNPC 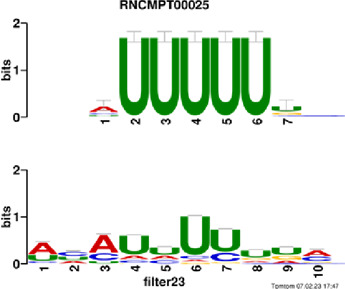 p-value: 6.41e−06	HNRNPC 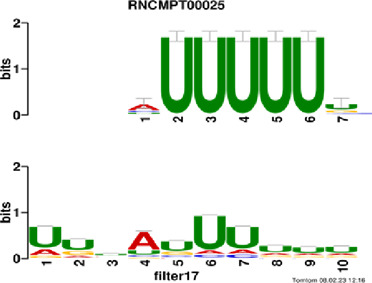 p-value: 3.92e−05
e-value: 1.02e−02	e-value: 1.89e−03	e-value: 6.54e−04	e-value: 4.00e−03
q-value: 2.45e−03	q-value: 8.07e−04	q-value: 5.11e−04	q-value: 1.71e−03
HUR 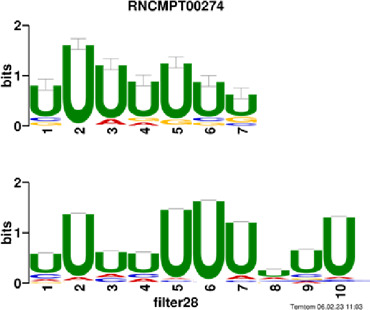 p-value: 2.02e−05	HUR 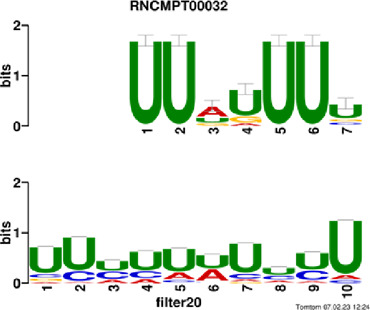 p-value: 4.47e−05	IGF2BP3 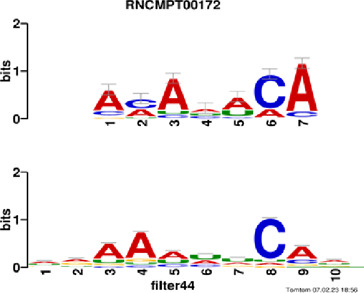 p-value: 3.78e−04	HUR 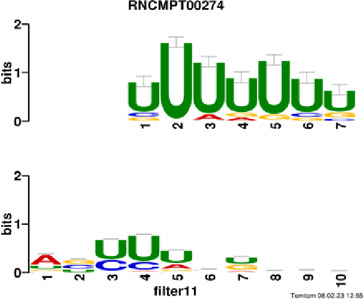 p-value: 2.51e−05
e-value: 2.06e−03	e-value: 4.56e−03	e-value: 3.85e−02	e-value: 2.56e−03
q-value: 1.79e−03	q-value: 3.33e−03	q-value: 2.95e−02	q-value: 2.18e−03
IGF2BP3 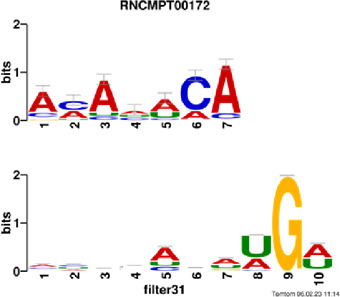 p-value: 4.78e−04	IGF2BP2 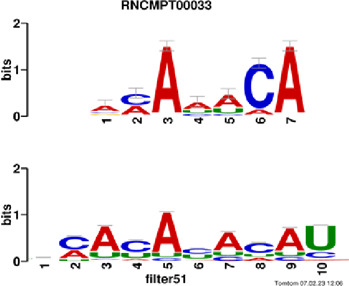 p-value: 8.57e−04	TIA1 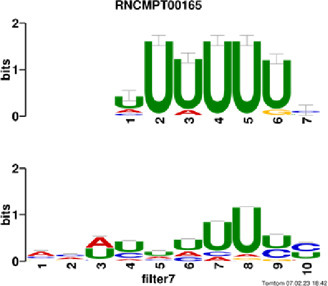 p-value: 9.97e−07	IGF2BP3 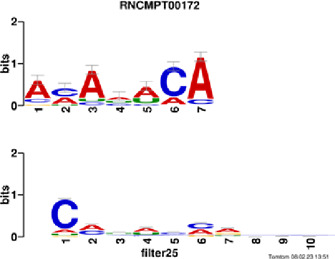 p-value: 1.44e−03
e-value: 4.88e−02	e-value: 8.74e−02	e-value: 1.02e−04	e-value: 1.47e−01
q-value: 4.53e−02	q-value: 2.06e−02	q-value: 4.40e−05	q-value: 4.02e−02
QKI 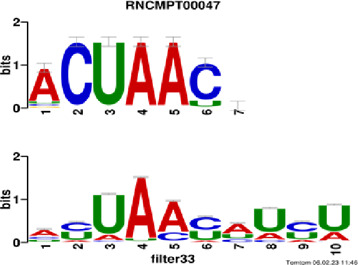 p-value: 1.03e−04	TIA1 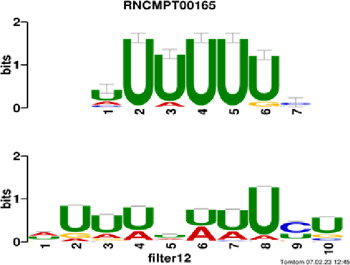 p-value: 2.65e−05	None	QKI 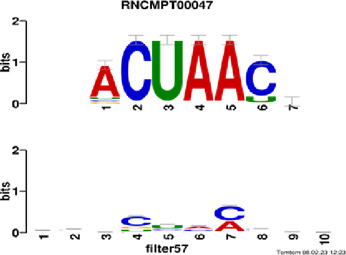 p-value: 4.84e−05
e-value: 1.05e−02	e-value: 2.71e−03		e-value: 4.93e−03
q-value: 9.95e−03	q-value: 2.34e−03		q-value: 4.63e−03
TIA1 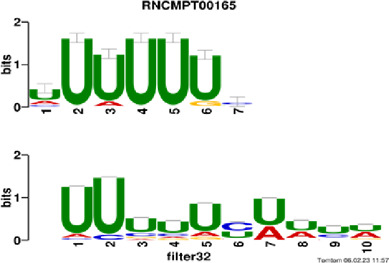 p-value: 9.70e−05	None	None	TIA1 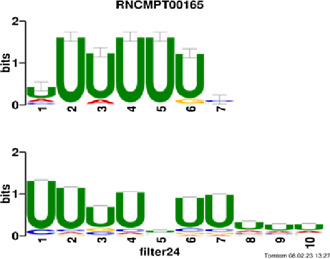 p-value: 3.65e−04
e-value: 9.89e−03			e-value: 3.73e−02
q-value: 4.24e−03			q-value: 6.36e−03

What’s more, Supplementary Tables S17–S20 display the top 5 motif logos found by CPBFCN, CRIP, circSLNN and iCircRBP-DHN for each dataset that correspond to the known database RNA/Ray2013_rbp_Homo_sapiens. All motif information (including match or not match with the known motifs in RNA/Ray2013_rbp_Homo_sapiens) is provided in the xlsx file Supplementary Data S2, which contain twelve sheets: FCN_all_motif, FCN_motif_match_known_motifdb, FCN_match_motif_sorted, CRIP_all_motif, CRIP_motif_match_known_motifdb, CRIP_match_motif_sorted, circSLNN_all_motif, SLNN_motif_match_known_motifdb, SLNN_match_motif_sorted, iCircRBP-DHN_all_motif, DHN _motif_match_known_motifdb, DHN_match_motif_sorted (Supplementary Tables S21–S32). Supplementary Figure S2 shows the performance comparison of three baseline models. In summary, for the task of motif discovery, CPBFCN does not hold a significant advantage over the other three baseline models. However, it still provides a novel avenue for feature learning and motif identification.

**FIGURE 4:**
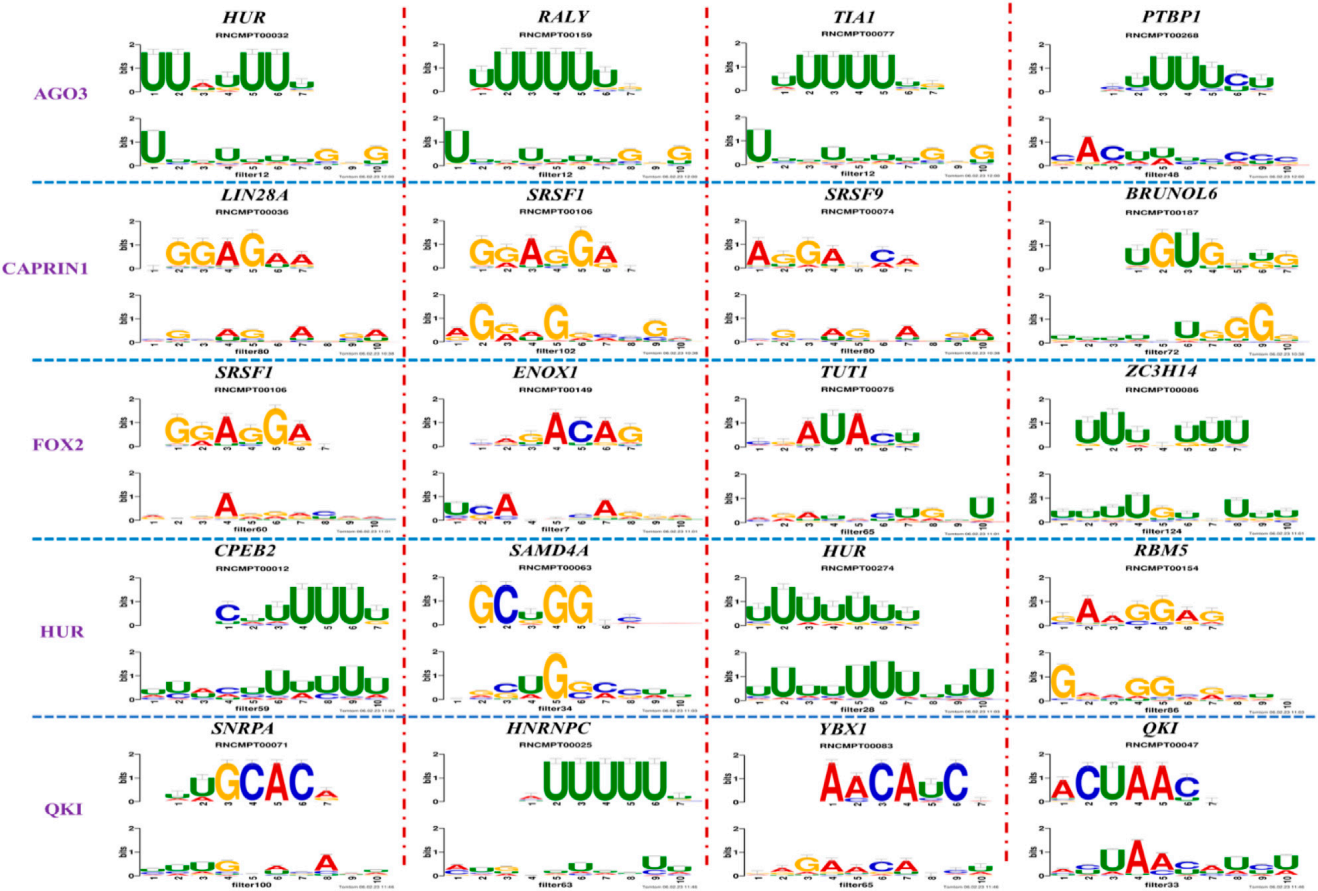
Some motif logos found by CPBFCN.

Figure 4 and Table 4 display motifs found by CPBFCN. According to the information obtained from protein databases and protein-related literature, some motifs play critical roles in gene expression regulation. For instance, the expression of specific factor E2F1 is related to transcription and cell proliferation, and RALY can impact the expression of E2F1, and thus regulate gene expression by modulating the expression of E2F1 (Cornella et al., 2017). LIN28A can not only recruit Tet1 to genomic binding sites, but the coordinated regulation of LIN28A and Tet1 can affect DNA methylation and gene expression (Zeng et al., 2016). SRSF1 is closely related to the immune system gene expression regulation (Paz et al., 2021).

**TABLE 4:** Some motif information found by CPBFCN.

Protein name	Motif found by CPBFCN	Known motif in database	Known motif sequence	Gene annotation	*p*-value	e-value	q-value
AGO3	UUUUUUUGGG	RNCMPT00032	UUAUUUU	HuR	0.000146	0.014931	0.005942
	GGGAUCGAGC	RNCMPT00067	GGAAGGA	SRSF9	0.000111	0.01128	0.010108
	UUUUUUUGGG	RNCMPT00159	UUUUUUG	RALY	0.000166	0.016972	0.005942
	UUUUUUUGGG	RNCMPT00274	UUUUUUU	HuR	0.00021	0.021456	0.005942
CAPRIN1	CCUCUACAAG	RNCMPT00172	ACAAACA	IGF2BP3	4.98E−05	0.005081	0.004714
	CAGAUUGACU	RNCMPT00184	AGUGUGA	RBM24	0.000328	0.033406	0.033245
	CCUCUACAAG	RNCMPT00033	ACAAACA	IGF2BP2	0.000494	0.050382	0.01804
	CGCAGAAGGA	RNCMPT00036	CGGAGAA	LIN28A	0.000425	0.04334	0.031237
FOX2	AGAUACUGUU	RNCMPT00075	CGAUACU	TUT1	6.21E−05	0.006331	0.006301
	AGGAGGACAA	RNCMPT00106	GGAGGAC	SRSF1	0.000122	0.012459	0.009772
	UUUUGUGUUU	RNCMPT00004	UGUGUGU	BRUNOL4	0.000298	0.030405	0.010691
	UUUUGUGUUU	RNCMPT00086	UUUGUUU	ZC3H14	0.000321	0.032775	0.010691
HUR	UUACUUUUUU	RNCMPT00012	CUUUUUU	CPEB2	4.75E−06	0.000484	0.000208
	UUACUUUUUU	RNCMPT00158	CUUUUUU	CPEB4	4.75E−06	0.000484	0.000208
	UGCUGGCCUU	RNCMPT00063	GCUGGAC	SAMD4A	6.01E−06	0.000613	0.000584
	UUUUUUUUUU	RNCMPT00274	UUUUUUU	HuR	2.02E−05	0.002064	0.001788
QKI	UUUGCACAAU	RNCMPT00071	UUGCACA	SNRPA	3.77E−05	0.003848	0.003516
	AUGAUUUUUU	RNCMPT00025	AUUUUUU	HNRNPC	0.000129	0.013187	0.005912
	AUGAUUUUUU	RNCMPT00167	AUUUUUU	HNRNPCL1	0.000129	0.013187	0.005912
	ACUAACAUCU	RNCMPT00047	ACUAACA	QKI	0.000103	0.010481	0.009947

With the development of biological experimental technology, more and more proteins are discovered to be significantly linked to the occurrence, development, metastasis, and treatment of complex malignant diseases. Table 5 shows the correlation between motifs found by CPBFCN and complex diseases. In Hepatocellular Carcinoma, the expression level of ZC3H14 has an obvious negative correlation with Hepatocellular Carcinoma progression. That is to say, ZC3H14 can not only serve as a tumor suppressor, but also a potential prognostic biomarker for Hepatocellular Carcinoma patients (Zhang et al., 2019b). SNRPA plays a critical role in gastric tumor size and progression through modulating nerve growth factor, and also be used as a prognostic biomarker for gastric cancer (Dou et al., 2018). Overexpression of CORO1C can promote the invasion and metastasis of breast cancer cells, and the upregulation or downregulation of YBX1 can promote or inhibit the expression of CORO1C. Therefore, the relationship between YBX1 and CORO1C provides a new way of inhibiting breast cancer cell metastasis (Lim et al., 2017). Overexpression of RBFOX1 enhances the Permeability of the Blood-Tumor Barrier through the LINC00673/MAFF pathway, which provides a new method for enhancing the efficacy of cancer therapy (Shen et al., 2020).

**TABLE 5:** Motifs found by CPBFCN are closely related to disease.

Protein name	Motif found by CPBFCN	Known motif in database	Known motif sequence	Gene annotation	Disease
FOX2	UUUUGUGUUU	RNCMPT00086	UUUGUUU	ZC3H14	Hepatocellular Carcinoma
IGF2BP2	UCAAGAAAAU	RNCMPT00064	AGAAAAA	SART3	Colorectal Cancer
HUR	GAAGGCGCUA	RNCMPT00154	GAAGGAG	RBM5	Lung Cancer
QKI	UUUGCACAAU	RNCMPT00071	UUGCACA	SNRPA	Gastric Cancer
	GAGAACAUCU	RNCMPT00083	AACAUCA	YBX1	Breast Cancer
AGO1	UACCUUUUCU	RNCMPT00079	UUUUUUC	U2AF2	Non-Small Cell Lung Cancer
TIA1	UCUGCAUGCC	RNCMPT00168	UGCAUGC	RBFOX1	Blood Tumor Barrier

Most motifs found by CPBFCN are related to gene expression regulation or cancer occurrence, metastasis, invasion, etc. Research have demonstrated that CircRNA’s RBP sponge function also play a role in gene expression regulation. Our future research will focus on two areas: CircRNA formation regulation and CircRNA-related gene expression (or disease) regulation. For CircRNA formation regulation, our aim is to gather pre-mRNA, protein and other data related to CircRNA formation, construct a regulatory network for CircRNA formation, and investigate the underlying mechanism that control CircRNA formation. For CircRNA-related gene expression regulation, our aim is to gather CircRNA, protein, miRNA, and other data related to gene expression regulation and disease, construct a regulatory network based on a heterogeneous graph neural network, and explore the gene expression (or disease) regulation related to CircRNA. Finally, CircRNA formation network and CircRNA-gene expression (include disease) network were combined to investigate the relationship between CircRNA formation and gene expression (or disease) regulation. Our study aims to uncover new CircRNA-related regulatory pathways and identify potential targets for disease treatment.

### 3.4 Motif distribution analysis

#### 3.4.1 Distribution analysis of motif directly found by CPBFCN

**FIGURE 5:**
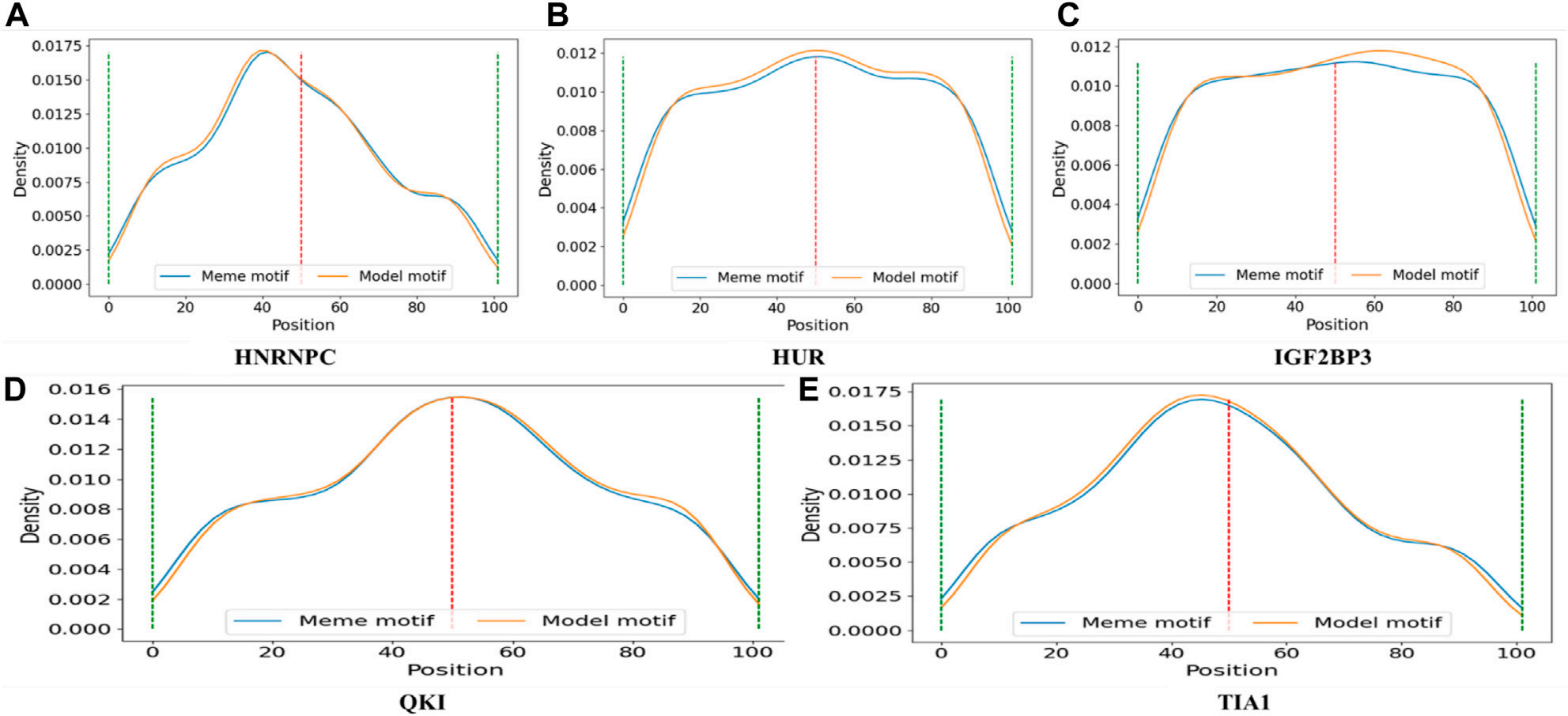
**(A)** The distribution of motif HNRNPC found by CPBFCN (model motif) and motif HNRNPC in RNA/Ray2013_rbp_Homo_sapiens (meme motif); **(B)** The distribution of motif HUR found by CPBFCN (model motif) and motif HUR in RNA/Ray2013_rbp_Homo_sapiens (meme motif); **(C)** The distribution of motif IGF2BP3 found by CPBFCN (model motif) and motif IGF2BP3 in RNA/Ray2013_rbp_Homo_sapiens (meme motif); **(D)** The distribution of motif QKI found by CPBFCN (model motif) and motif QKI in RNA/Ray2013_rbp_Homo_sapiens (meme motif); **(E)** The distribution of motif TIA1 found by CPBFCN (model motif) and motif TIA1 in RNA/Ray2013_rbp_Homo_sapiens (meme motif). Here, meme motif represents the motif sequence in RNA/Ray2013_rbp_Homo_sapiens, model motif represents the motif sequence found by CPBFCN.

Table 4 displays 5 motifs directly found by CPBFCN: HNRNPC, HUR, IGF2BP3, QKI, and TIA1. According to the details described in the “Data” section, we first need to find the midpoint of the protein binding region, and then select 50 sites from the upstream and downstream to generate experimental data. Thus, these motifs should be lie in the middle of the experimental data range. Figure 5 confirms our previous assumptions concerning the corresponding distribution of HUR and QKI. For the remaining three motifs, their primary distribution areas have minor variations from the midpoint, this aligns with our initial expectations. In general, CPBFCN successfully predicted motif-binding regions located in the central region of input sequence.

#### 3.4.2 Motif distribution and CircRNA sponge analysis

**FIGURE 6:**
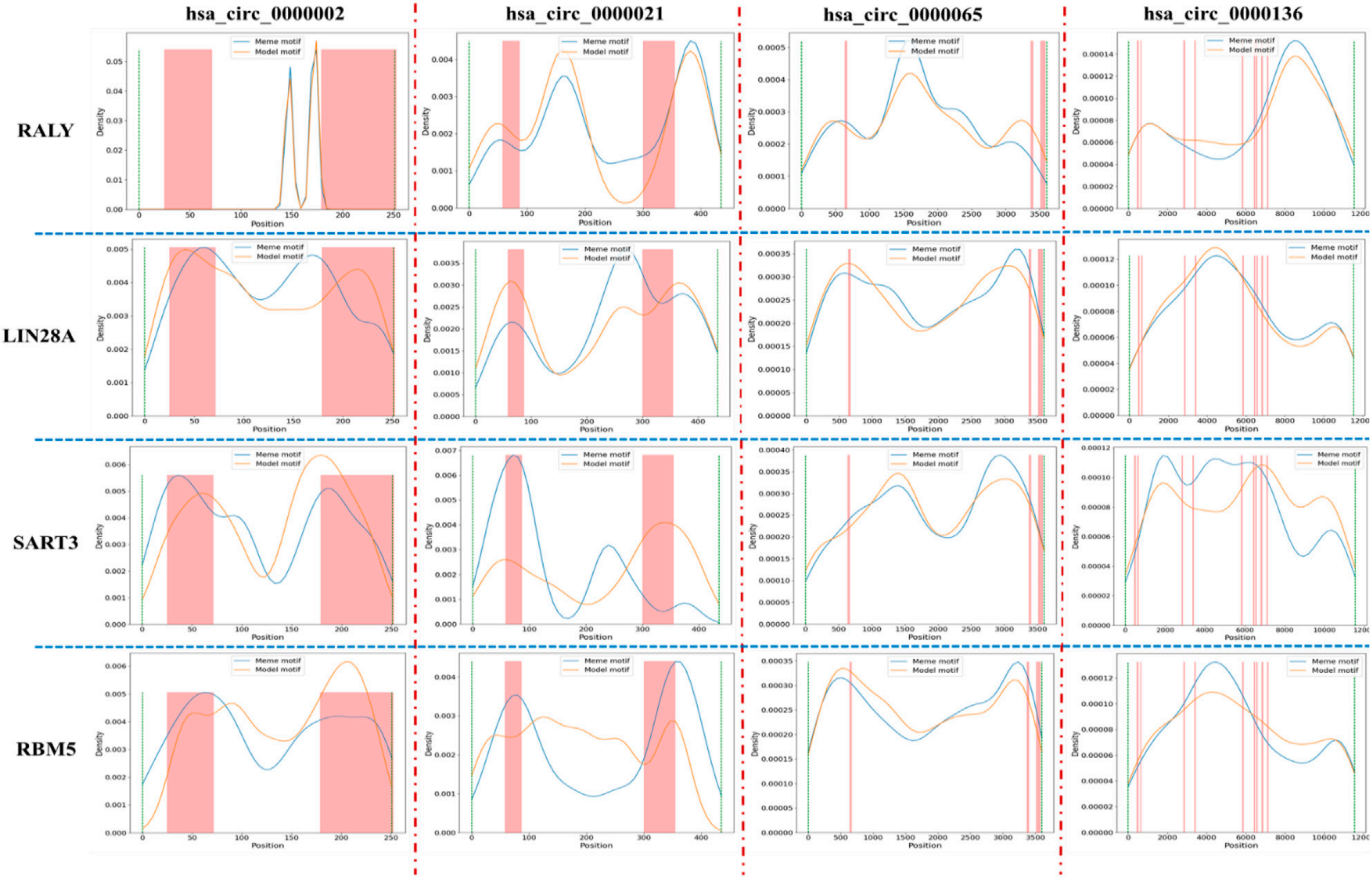
The relative positional between protein binding region and special motif distribution in CircRNA. Here, blue line and yellow line represents the distribution of meme motif and model motif, respectively, and the pink bar represents the protein binding region in CircRNA sequence. Due to the different length of CircRNA, the column width may be thick or thin.

In the “motif discovery performance analysis” section, we have presented several protein cases that are implicated in gene expression regulation and cancer. Due to the interaction between protein and CircRNA, the expression level of these proteins is affected by CircRNA. This is also referred to as CircRNA sponge. To examine the correlation between CircRNA sponge region and motif distribution, we tallied the protein-binding position of all CircRNAs in circinteractome, and merged them into a file that outlines the protein-binding area of each CircRNA sequence. Then, we selected four motifs (RALY, LIN28A, SART3, RBM5) and four CircRNAs (hsa_circ_0000002, has_circ_0000021, hsa_circ_0000065, hsa_circ_0000136). The distribution of four motifs in CircRNA sequence is depicted in Figure 6.

From Figure 6, we found that the main distribution regions of LIN28A, SART3, and RBM5 in hsa_circ_0000002 and hsa_circ_0000021 largely overlaps with the protein-binding region in CircRNA sequence. However, there are only a few motif distribution regions in hsa_circ_0000065 and hsa_circ_0000136 that overlaps with the protein-binding region. For the motif RALY, its main distribution region only marginally overlaps with the protein-binding region in four CircRNA. Due to the large number of motifs and CircRNA, we will present the distribution of only four motifs in four CircRNA. Generally, the overlap of the main distribution region and CircRNA sponge region indicates that CircRNA can act as a sponge and influence protein expression level through CircRNA-protein binding, thereby regulating gene expression and cancer. In future research, we will collaborate with medical institutions and scientific research institutions to further confirm the relationship between CircRNA sponge and protein and explore potential regulatory pathways. There is an expectation that this research will aid in the treatment of complex diseases.

#### 3.4.3 Some short sequences help CPBFCN to predict CircRNA-protein binding site

During experiments, we found that when the input length is 201, the miou, iou_0, iou_1 of six datasets (EIF4A3, FOX2, IGF2BP1, IGF2BP2, IGF2BP3, and ZC3H7B) outperform those with input length 101. It is well-established that deep learning models necessitate a significant amount of training data for the extraction of adequate features. Increasing the input data length may potentially improve model performance. Additionally, we are still thinking about whether there are some short sequences in the added sequence that can help identify CircRNA-protein binding sites. Here, we first divide each record in each dataset into short sequences with length 12, then count the positions of these short sequences across all records, and finally generate a distribution figure corresponding to each short sequence.

**FIGURE 7:**
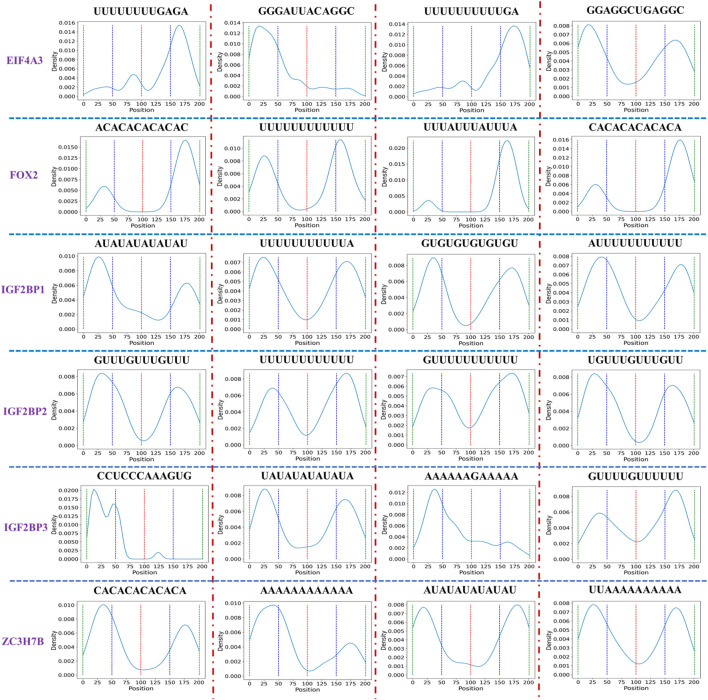
Distribution of the short sequence with high occurrences in six datasets. To facilitate the statistics of short sequence distribution, we divide the sequence into four intervals, each interval length is 50, and the red, blue, and green dotted lines represent the boundaries of each interval. The peaks in each interval represent that a short sequence appears more frequently in the current interval.

Table 6 shows motif numbers in six datasets before and after threshold filtering. While the total count of motif within six datasets is substantial, only a small number of motifs with high occurrences remain after the filtering process. If the threshold set for all six datasets is too high, motifs may not be found in some datasets, such as FOX2, IGF2BP2, ZC3H7B. Conversely, a low threshold may identify numerous candidate motifs. Therefore, different thresholds were set for six datasets. Figure 7 shows the distribution of some motifs with high occurrences in six datasets. The distribution of all motifs is shown in Supplementary Figures S3–S8. From Figure 7 and Supplementary Figures S3–S8, we found that the main distribution region of motif is divided into four types: left flank of the original binding region, right flank of the original binding region, both the left flank and right flank, and the original binding region. This suggests that alongside motif distribution within the original binding region, other motifs might assist in identifying protein binding sites independently or collectively. In our future research, besides acquiring more experimental data, we will also aim to collaborate with research institutions to investigate if these sequences can help identify protein binding sites via biological experiments.

**TABLE 6:** Motifs with high occurrences in six datasets.

Protein name	Motif number	Threshold	Motif number after threshold filtering
EIF4A3	2146479	50	127
FOX2	111833	10	5
IGF2BP1	2460754	30	72
IGF2BP2	1337480	30	37
IGF2BP3	2387811	30	98
ZC3H7B	1881151	30	56

## 4 Discussion

CircRNA-protein binding is a crucial factor in complex biological activity and disease development. The prediction of CircRNA-protein binding motifs helps to unveil the role of CircRNA in gene expression regulation. In this study, CPBFCN was used to predict CircRNA-protein binding motif. As a nucleotide-level model, CPBFCN uses CircRNA sequence as input data. Only a small fraction of CircRNA sequence contains protein binding sites, with other sites being considered negative samples, and the proportion of positive and negative samples is unbalanced. To address this issue, hard negative mining loss was introduced. Despite the lack of a significant advantage of CPBFCN, it still provides a new path for identifying CircRNA motifs**.** Further analysis of the motif distribution showed that the overlap between motif main distribution region and CircRNA sponge region is more favorable for to the regulatory function of CircRNA in the biological process, and some short sequences help to identify CircRNA-protein binding sites.

For future research, we have two directions. One is to enhance the motif prediction ability of CPBFCN by fine-tuning the model structure and parameters. The other is based on CPBFCN and includes three subtasks. Firstly, based on the experimental result of CPBFCN and CircCNN, combined with other CircRNA-related data, we will construct a CircRNA formation regulation network by integrating the experimental outcomes of CPBFCN and CircCNN with other CircRNA-related data, and then explore the regulatory mechanism behind CircRNA formation. Secondly, make full use of CPBFCN to identify protein binding regions in CircRNA sequence, in-depth study the role of CircRNA sponge, integrate biological data such as miRNA, protein, and construct CircRNA-gene expression (and disease) regulation network, reveal the function of CircRNA in biological activity. Thirdly, CPBFCN is a nucleotide-level model that can be used to identify whether the CircRNA sequence site is mutated, and then to study the impact of CircRNA site mutation on gene expression and disease regulation.

## Data Availability

Publicly available datasets were analyzed in this study. This data can be found here: https://github.com/kavin525zhang/CRIP and https://github.com/szhh521/CPBFCN.
