# Supplementary material for: Nucleotide-level prediction of CircRNA-protein binding based on fully convolutional neural network
Source: Front Genet. 2023 Oct 6;14:1283404. doi: 10.3389/fgene.2023.1283404 (PMC10587422; doi:10.3389/fgene.2023.1283404)
Supplement: Supplementary file 1 [file DataSheet1.PDF]

# Supplementary data for

# Nucleotide-Level Prediction of CircRNA-Protein Binding based on Fully Convolutional Neural Network

Zhen Shen[1], Wei Liu[1], ShuJun Zhao[1], QinHu Zhang[2], SiGuo Wang[2], and Lin Yuan[3,4,5*]

[1]School of Computer and Software, Nanyang Institute of Technology, Changjiang Road 80, Nanyang, Henan 473004, China.
[2] EIT Institute for Advanced Study, Ningbo, Zhejiang, 315201, China.
[3]Key Laboratory of Computing Power Network and Information Security, Ministry of Education, Shandong Computer Science Center, Qilu University of Technology (Shandong Academy of Sciences), Jinan, China
[4]Shandong Engineering Research Center of Big Data Applied Technology, Faculty of Computer Science and Technology, Qilu University of Technology (Shandong Academy of Sciences), Jinan, China
[5]Shandong Provincial Key Laboratory of Computer Networks, Shandong Fundamental Research Center for Computer Science, Jinan, China

## Section 1 Experimental Data Used in This Study

***Data Sources***

In this study, 37 CircRNA-protein binding datasets were used to evaluate model performance. These datasets were also used by various existing deep learning-based CircRNA-protein binding prediction models, like CRIP, DeCban, and circSLNN. CRIP was the first deep learning model using these datasets. After reading the data generation methods in CRIP, I downloaded 37 files from the website (Circular RNA Interactome) that contain CircRNA-protein binding information and used the same method to obtain sequence files. After comparing with the existing data, the authenticity of these datasets has also been confirmed. To ensure data consistency among different models, we use the same experimental data in this study. In the next section, we will describe how the sequence data is generated and processed.

***Data Processing***

To generate CircRNA-protein binding sequence, there are three steps:

1. Create bed file: for each CircRNA-protein binding dataset, get chromatin name, motif start site and end site, +/- strand information from motif data downloaded from the website (Circular RNA Interactome), taking the middle point of the motif region as the center, combined with the input data length we need, confirm the start and end positions of the subsequence containing the motif region in genome and finally generate bed file.
2. Obtain fasta file: referring to the hg19 genome file, use bedtools—getfasta to generate the fasta file.

3. Remove redundant sequence by using CD-HIT with threshold 0.8

After the above steps, we obtained 37 self-generated datasets. By comparing with 37 datasets obtained from CRIP, we confirm that the above steps are correct. Table S1 shows the record number of each dataset. Table S2 shows the motif information in each dataset. From Table S2, we found that the max length of the CircRNA-protein binding region is larger than 100. In most datasets, the proportion of CircRNA-protein binding region length less than 100 is greater than 80%. Only FOX2 is an exception, the proportion is only 43%. These data can help when we make model adjustments and parameter selections.

**Table S1.** Number of positive sequence and negative sequence with different input length

| Data | 101 | | 201 | | 501 | |
|---|---|---|---|---|---|---|
| | positive | negative | positive | negative | positive | negative |
| AGO1 | 17318 | 17318 | 16368 | 14857 | 12774 | 11505 |
| AGO2 | 20000 | 20000 | 17593 | 15773 | 11243 | 10070 |
| AGO3 | 3124 | 3124 | 3058 | 2812 | 2680 | 2515 |
| ALKBH5 | 770 | 770 | 765 | 722 | 687 | 668 |
| AUF1 | 2896 | 2896 | 2872 | 2641 | 2760 | 2595 |
| C17ORF85 | 1016 | 1016 | 1005 | 950 | 861 | 842 |
| C22ORF28 | 5292 | 5292 | 5161 | 4800 | 3952 | 3610 |
| CAPRIN1 | 5298 | 5298 | 5234 | 4858 | 4333 | 3963 |
| DGCR8 | 20000 | 20000 | 19567 | 17665 | 16816 | 14747 |
| EIF4A3 | 20000 | 20000 | 18202 | 16322 | 12115 | 10869 |
| EWSR1 | 4695 | 4695 | 4619 | 4301 | 4270 | 3900 |
| FMRP | 20000 | 20000 | 17542 | 15749 | 10379 | 9221 |
| FOX2 | 605 | 605 | 600 | 563 | 582 | 563 |
| FUS | 20000 | 20000 | 19232 | 17332 | 16631 | 14630 |
| FXR1 | 927 | 927 | 910 | 862 | 849 | 830 |
| FXR2 | 5635 | 5635 | 5454 | 5069 | 4763 | 4388 |
| HNRNPC | 14224 | 14224 | 4438 | 4150 | 4866 | 4457 |
| HUR | 20000 | 20000 | 19353 | 17455 | 11957 | 10731 |
| IGF2BP1 | 20000 | 20000 | 19761 | 17856 | 14684 | 12829 |
| IGF2BP2 | 10000 | 10000 | 9868 | 8797 | 7168 | 6563 |
| IGF2BP3 | 20000 | 20000 | 19739 | 17834 | 14294 | 12548 |
| LIN28A | 18277 | 18277 | 17511 | 15727 | 14436 | 12687 |
| LIN28B | 7888 | 7888 | 7647 | 6744 | 6759 | 6196 |
| METTL3 | 2666 | 2666 | 2571 | 2389 | 2343 | 2261 |
| MOV10 | 5889 | 5889 | 5736 | 5340 | 4755 | 4380 |
| PTB | 20000 | 20000 | 19761 | 17856 | 15671 | 13808 |
| PUM2 | 2329 | 2329 | 2291 | 2180 | 2104 | 2023 |
| QKI | 1033 | 1033 | 1026 | 970 | 991 | 949 |
| SFRS1 | 8595 | 8595 | 8160 | 7219 | 6679 | 6119 |
| TAF15 | 1467 | 1467 | 1444 | 1376 | 1321 | 1261 |
| TDP43 | 5484 | 5484 | 5406 | 5026 | 4912 | 4458 |
| TIA1 | 2202 | 2202 | 2197 | 2093 | 1914 | 1841 |
| TIAL1 | 5456 | 5456 | 5445 | 5061 | 4704 | 4329 |
| TNRC6 | 1101 | 1101 | 1096 | 1039 | 1017 | 970 |
| U2AF65 | 8224 | 8224 | 7989 | 7048 | 6903 | 6337 |

| | | | | | | |
|---|---|---|---|---|---|---|
| WTAP | 446 | 446 | 428 | 404 | 365 | 348 |
| ZC3H7B | 13119 | 13119 | 13025 | 11696 | 11213 | 10040 |

**Table S2.** Motif length information in 37 datasets.

| Data | Record Number | Motif length | | | | The ratio of motif length less than 100 | The ratio of motif length less than 50 |
|---|---|---|---|---|---|---|---|
| | | Max | Min | Average | Median | | |
| AGO1 | 17318 | 175 | 21 | 30 | 27 | 100% | 95% |
| AGO2 | 20000 | 1217 | 21 | 42 | 37 | 97% | 82% |
| AGO3 | 3124 | 138 | 21 | 26 | 24 | 100% | 99% |
| ALKBH5 | 770 | 137 | 22 | 36 | 31 | 100% | 88% |
| AUF1 | 2896 | 154 | 21 | 30 | 29 | 100% | 99% |
| C17ORF85 | 1016 | 93 | 21 | 31 | 29 | 100% | 95% |
| C22ORF28 | 5292 | 241 | 21 | 38 | 34 | 99% | 83% |
| CAPRIN1 | 5298 | 328 | 21 | 48 | 39 | 95% | 67% |
| DGCR8 | 20000 | 222 | 21 | 33 | 30 | 100% | 92% |
| EIF4A3 | 20000 | 1453 | 21 | 59 | 49 | 89% | 51% |
| EWSR1 | 4695 | 224 | 21 | 31 | 29 | 100% | 96% |
| FMRP | 20000 | 496 | 21 | 38 | 31 | 97% | 83% |
| FOX2 | 605 | 14803 | 48 | 437 | 114 | 43% | 2% |
| FUS | 20000 | 76 | 19 | 31 | 29 | 100% | 97% |
| FXR1 | 927 | 46 | 21 | 24 | 24 | 100% | 100% |
| FXR2 | 5635 | 122 | 21 | 26 | 24 | 100% | 98% |
| HNRNPC | 14224 | 316 | 21 | 34 | 30 | 99% | 92% |
| HUR | 20000 | 3523 | 21 | 49 | 40 | 96% | 81% |
| IGF2BP1 | 20000 | 1292 | 21 | 65 | 55 | 89% | 41% |
| IGF2BP2 | 10000 | 591 | 21 | 62 | 53 | 89% | 44% |
| IGF2BP3 | 20000 | 844 | 21 | 70 | 58 | 85% | 36% |
| LIN28A | 18277 | 254 | 21 | 40 | 33 | 97% | 82% |
| LIN28B | 7888 | 1493 | 21 | 49 | 29 | 90% | 82% |
| METTL3 | 2666 | 368 | 21 | 35 | 31 | 99% | 91% |
| MOV10 | 5889 | 132 | 21 | 39 | 36 | 100% | 82% |
| PTB | 20000 | 1256 | 21 | 54 | 45 | 92% | 66% |
| PUM2 | 2329 | 161 | 21 | 36 | 32 | 100% | 92% |
| QKI | 1033 | 73 | 21 | 33 | 32 | 100% | 97% |
| SFRS1 | 8595 | 269 | 21 | 45 | 41 | 99% | 74% |
| TAF15 | 1467 | 165 | 21 | 28 | 27 | 100% | 99% |
| TDP43 | 5484 | 586 | 19 | 46 | 34 | 94% | 73% |
| TIA1 | 2202 | 300 | 21 | 41 | 34 | 97% | 79% |
| TIAL1 | 5456 | 444 | 21 | 45 | 35 | 95% | 75% |
| TNRC6 | 1101 | 209 | 21 | 31 | 29 | 99% | 96% |
| U2AF65 | 8224 | 777 | 21 | 36 | 31 | 99% | 88% |
| WTAP | 446 | 314 | 21 | 39 | 31 | 97% | 85% |
| ZC3H7B | 13119 | 1219 | 21 | 69 | 52 | 84% | 47% |

***Data Use***

37 datasets with length 101 used in this study come from CRIP. Of course, we also used the steps described in the previous section to confirm that these datasets are true and reliable. In addition, to explore the impact of the upstream and downstream short sequences of CircRNA-protein binding region on the motif sites identification, we used the same method to generate input data with lengths 201 and 501. Figure 1 shows the performance comparison of CPBFCN with 24 parameter combinations (see Table S4). From Figure 1 in the paper, we found that when the input data length increases, CPBFCN has no advantage in motif site identification and running time. This is not what we want, so we finally choose the input data length to be 101.

## Section 2 Motif Distribution

In this study, we use motif distribution for three purposes: motif prediction performance test, CircRNA sponge, and short sequence that help identify CircRNA-protein binding sites. Two matching methods were used in this study: exact matching and fuzzy matching. Since the motif length is only 12, it is difficult to show motif distribution using the start site and end site to represent the short sequence position. Regardless of the matching method, we use the midpoint between the start site and end site of the short sequence to represent the short sequence position in the CircRNA sequence. By counting the occurrence positions of the middle points sites, a density distribution figure is finally generated.

For the motif prediction performance test, whether the motif is found by the model or the motif in the database, it is obtained through statistical analysis. If the exact matching method is used, it is difficult to find completely consistent short sequences in experimental datasets. Therefore, we use the fuzzy matching method. When the matching motif site number exceeds half the motif length, the match is considered successful. According to the data generation mechanism in this paper, if the main distribution area of the motif directly found by CPBFCN is in the middle of the sequence, it means that the model has a better ability to identify motifs.

For the CircRNA sponge, by identifying whether the distribution region of the motif in CircRNA sequence overlaps with known protein binding regions to determine whether a CircRNA can affect the protein expression level or not, and then achieve the goal of regulating gene expression.

To find the motif that can help identify CircRNA-protein binding sites, we do not use the motif found by CPBFCN. We analyzed the short sequences that appeared in 6 datasets, counted the number of short sequences with length 12 that appeared in the datasets, used the exact matching method to count the position of each short sequence, and finally generated the motif distribution figure. By comparing whether the main distribution area is in the newly added sequence to determine whether the short sequence can help identify motif sites.

## Section 3 Supplementary data for experimental results

**Table S3.** Motif name in Ray2013_rbp_Homo_sapiens

| No | Name | No | Name | No | Name | No | Name |
|---|---|---|---|---|---|---|---|
| 1 | A1CF | 21 | HNRNPCL1 | 41 | PCBP1 | 61 | SAMD4A |
| 2 | ANKHD1 | 22 | HNRNPH2 | 42 | PCBP2 | 62 | SART3 |
| 3 | BRUNOL4 | 23 | HNRNPK | 43 | PPRC1 | 63 | SFPQ |

| | | | | | | | |
|---|---|---|---|---|---|---|---|
| 4 | BRUNOL5 | 24 | HNRNPL | 44 | PTBP1 | 64 | SNRNP70 |
| 5 | BRUNOL6 | 25 | HNRPLL | 45 | QKI | 65 | SNRPA |
| 6 | CNOT4 | 26 | HuR | 46 | RALY | 66 | SRSF1 |
| 7 | CPEB2 | 27 | IGF2BP2 | 47 | RBFOX1 | 67 | SRSF10 |
| 8 | CPEB4 | 28 | IGF2BP3 | 48 | RBM24 | 68 | SRSF2 |
| 9 | DAZAP1 | 29 | KHDRBS1 | 49 | RBM28 | 69 | SRSF7 |
| 10 | ENOX1 | 30 | KHDRBS2 | 50 | RBM3 | 70 | SRSF9 |
| 11 | ESRP2 | 31 | KHDRBS3 | 51 | RBM4 | 71 | TARDBP |
| 12 | FMR1 | 32 | LIN28A | 52 | RBM41 | 72 | TIA1 |
| 13 | FUS | 33 | MATR3 | 53 | RBM42 | 73 | TUT1 |
| 14 | FXR1 | 34 | MBNL1 | 54 | RBM45 | 74 | U2AF2 |
| 15 | FXR2 | 35 | MSI1 | 55 | RBM46 | 75 | YBX1 |
| 16 | G3BP2 | 36 | PABPC1 | 56 | RBM5 | 76 | YBX2 |
| 17 | HNRNPA1 | 37 | PABPC3 | 57 | RBM6 | 77 | ZC3H10 |
| 18 | HNRNPA1 L2 | 38 | PABPC4 | 58 | RBM8A | 78 | ZC3H14 |
| 19 | HNRNPA2 B1 | 39 | PABPC5 | 59 | RBMS1 | 79 | ZCRB1 |
| 20 | HNRNPC | 40 | PABPN1 | 60 | RBMS3 | 80 | ZNF638 |

Note: File "Ray2013_rbp_Homo_sapiens" contains 102 motif records, some of which have the same motif name and have different letter-probability matrix. After removing duplicate information, there are finally 80 motif records.

**Table S4.** 24 combinations of 4 parameters

| | | | |
|---|---|---|---|
| M1 | (101, BCE, FCN) | M13 | (201, HNML-0.5, FCN) |
| M2 | (101, BCE, FCNA) | M14 | (201, HNML-0.5, FCNA) |
| M3 | (101, HNML-0.3, FCN) | M15 | (201, HNML-0.7, FCN) |
| M4 | (101, HNML-0.3, FCNA) | M16 | (201, HNML-0.7, FCNA) |
| M5 | (101, HNML-0.5, FCN) | M17 | (501, BCE, FCN) |
| M6 | (101, HNML-0.5, FCNA) | M18 | (501, BCE, FCNA) |
| M7 | (101, HNML-0.7, FCN) | M19 | (501, HNML-0.3, FCN) |
| M8 | (101, HNML-0.7, FCNA) | M20 | (501, HNML-0.3, FCNA) |
| M9 | (201, BCE, FCN) | M21 | (501, HNML-0.5, FCN) |
| M10 | (201, BCE, FCNA) | M22 | (501, HNML-0.5, FCNA) |
| M11 | (201, HNML-0.3, FCN) | M23 | (501, HNML-0.7, FCN) |
| M12 | (201, HNML-0.3, FCNA) | M24 | (501, HNML-0.7, FCNA) |

Note: FCN and FCNA represents whether to use global average pooling.

**Table S5.** 19 datasets performance comparison of CPBFCN with the parameter: 101/201/501, BCE, and FCN.

| Data | 101, BCE, FCN | | | 201, BCE, FCN | | | 501, BCE, FCN | | |
|---|---|---|---|---|---|---|---|---|---|
| | miou | iou_0 | iou_1 | miou | iou_0 | iou_1 | miou | iou_0 | iou_1 |
| AGO2 | 0.8051 | 0.8467 | 0.7634 | 0.8189 | 0.9205 | 0.7172 | 0.4576 | 0.9152 | 0.0001 |
| ALKBH5 | 0.8425 | 0.8888 | 0.7961 | 0.8387 | 0.9372 | 0.7403 | 0.4641 | 0.9229 | 0.0052 |
| CAPRIN1 | 0.7645 | 0.8028 | 0.7263 | 0.7276 | 0.8746 | 0.5806 | 0.4684 | 0.8871 | 0.0497 |
| EIF4A3 | 0.679 | 0.703 | 0.6551 | 0.7684 | 0.8603 | 0.6766 | 0.5172 | 0.8866 | 0.1478 |
| FMRP | 0.8063 | 0.8634 | 0.7493 | 0.8094 | 0.9232 | 0.6956 | 0.5505 | 0.9286 | 0.1724 |
| FOX2 | 0.4394 | 0.3116 | 0.5673 | 0.6234 | 0.6034 | 0.6435 | 0.3763 | 0.6276 | 0.125 |

| | | | | | | | | | |
|---|---|---|---|---|---|---|---|---|---|
| FUS | 0.9022 | 0.939 | 0.8654 | 0.891 | 0.9644 | 0.8175 | 0.6558 | 0.9552 | 0.3564 |
| FXR1 | 0.9608 | 0.9805 | 0.9412 | 0.9214 | 0.9804 | 0.8625 | 0.7354 | 0.9732 | 0.4977 |
| IGF2BP1 | 0.7011 | 0.6974 | 0.7049 | 0.7928 | 0.8679 | 0.7177 | 0.5624 | 0.8827 | 0.242 |
| IGF2BP2 | 0.7144 | 0.7174 | 0.7114 | 0.7993 | 0.8757 | 0.7229 | 0.5255 | 0.8757 | 0.1753 |
| IGF2BP3 | 0.6691 | 0.6625 | 0.6757 | 0.7753 | 0.8501 | 0.7005 | 0.659 | 0.879 | 0.4389 |
| LIN28A | 0.7985 | 0.8513 | 0.7456 | 0.8098 | 0.9199 | 0.6997 | 0.5274 | 0.9221 | 0.1326 |
| LIN28B | 0.7891 | 0.8587 | 0.7195 | 0.7708 | 0.9027 | 0.639 | 0.6132 | 0.9225 | 0.3038 |
| PTB | 0.7417 | 0.7712 | 0.7122 | 0.7879 | 0.8861 | 0.6897 | 0.6672 | 0.912 | 0.4223 |
| TDP43 | 0.7505 | 0.805 | 0.696 | 0.793 | 0.9038 | 0.6822 | 0.6537 | 0.9268 | 0.3806 |
| TIA1 | 0.7914 | 0.8408 | 0.742 | 0.8174 | 0.9184 | 0.7164 | 0.6074 | 0.9142 | 0.3006 |
| TIAL1 | 0.7713 | 0.8214 | 0.7212 | 0.811 | 0.9119 | 0.7101 | 0.6353 | 0.9291 | 0.3416 |
| WTAP | 0.8049 | 0.8599 | 0.7498 | 0.7954 | 0.9112 | 0.6796 | 0.4718 | 0.9049 | 0.0388 |
| ZC3H7B | 0.6615 | 0.6841 | 0.6389 | 0.7329 | 0.8323 | 0.6336 | 0.4744 | 0.8278 | 0.121 |
| Avg | 0.7575 | 0.7845 | 0.7306 | 0.7939 | 0.8865 | 0.7013 | 0.5591 | 0.8944 | 0.2238 |

**Table S6.** 19 datasets performance comparison of CPBFCN with the parameter: 101/201/501, BCE, and FCNA.

| Data | 101, BCE, FCNA | | | 201, BCE, FCNA | | | 501, BCE, FCNA | | |
|---|---|---|---|---|---|---|---|---|---|
| | miou | iou_0 | iou_1 | miou | iou_0 | iou_1 | miou | iou_0 | iou_1 |
| AGO2 | 0.8052 | 0.8473 | 0.7631 | 0.8191 | 0.9204 | 0.7177 | 0.483 | 0.9026 | 0.0635 |
| ALKBH5 | 0.8422 | 0.8898 | 0.7946 | 0.8384 | 0.9382 | 0.7386 | 0.4645 | 0.9234 | 0.0057 |
| CAPRIN1 | 0.763 | 0.8005 | 0.7255 | 0.796 | 0.8987 | 0.6932 | 0.4934 | 0.8922 | 0.0946 |
| EIF4A3 | 0.679 | 0.7011 | 0.657 | 0.7682 | 0.8615 | 0.6749 | 0.5782 | 0.9008 | 0.2556 |
| FMRP | 0.8063 | 0.8619 | 0.7508 | 0.8112 | 0.9236 | 0.6988 | 0.5735 | 0.932 | 0.2151 |
| FOX2 | 0.4367 | 0.3088 | 0.5645 | 0.6252 | 0.5987 | 0.6517 | 0.4022 | 0.6318 | 0.1726 |
| FUS | 0.9022 | 0.9392 | 0.8652 | 0.8935 | 0.9653 | 0.8217 | 0.6816 | 0.9488 | 0.4144 |
| FXR1 | 0.9633 | 0.9816 | 0.9449 | 0.9327 | 0.9831 | 0.8822 | 0.7254 | 0.9727 | 0.4782 |
| IGF2BP1 | 0.7015 | 0.6962 | 0.7069 | 0.7942 | 0.8687 | 0.7198 | 0.5944 | 0.8741 | 0.3147 |
| IGF2BP2 | 0.7147 | 0.7157 | 0.7137 | 0.8006 | 0.8763 | 0.7248 | 0.6542 | 0.893 | 0.4153 |
| IGF2BP3 | 0.669 | 0.663 | 0.6749 | 0.7773 | 0.851 | 0.7035 | 0.519 | 0.8107 | 0.2272 |
| LIN28A | 0.7983 | 0.8504 | 0.7462 | 0.8107 | 0.9203 | 0.7011 | 0.5073 | 0.9124 | 0.1022 |
| LIN28B | 0.789 | 0.8586 | 0.7195 | 0.7717 | 0.9019 | 0.6414 | 0.6021 | 0.9223 | 0.2818 |
| PTB | 0.7419 | 0.7717 | 0.7122 | 0.789 | 0.8874 | 0.6905 | 0.6151 | 0.9121 | 0.3182 |
| TDP43 | 0.7495 | 0.805 | 0.6939 | 0.7951 | 0.9039 | 0.6862 | 0.5765 | 0.916 | 0.237 |
| TIA1 | 0.7931 | 0.8417 | 0.7445 | 0.8186 | 0.922 | 0.7153 | 0.6091 | 0.922 | 0.2961 |
| TIAL1 | 0.7721 | 0.8193 | 0.725 | 0.8133 | 0.9128 | 0.7139 | 0.7052 | 0.9384 | 0.472 |
| WTAP | 0.8065 | 0.8612 | 0.7519 | 0.8128 | 0.9247 | 0.7009 | 0.4664 | 0.9212 | 0.0116 |
| ZC3H7B | 0.6618 | 0.6843 | 0.6392 | 0.7338 | 0.8329 | 0.6346 | 0.4304 | 0.8582 | 0.0026 |
| Avg | 0.7576 | 0.7841 | 0.7312 | 0.8001 | 0.889 | 0.7111 | 0.5622 | 0.8939 | 0.2304 |

**Table S7.** 19 datasets performance comparison of CPBFCN with the parameter: 101/201/501, HNML_0.3, and FCN.

| Data | 101, HNML-0.3, FCN | | | 201, HNML-0.3, FCN | | | 501, HNML-0.3, FCN | | |
|---|---|---|---|---|---|---|---|---|---|
| | miou | iou_0 | iou_1 | miou | iou_0 | iou_1 | miou | iou_0 | iou_1 |
| AGO2 | 0.8061 | 0.8484 | 0.7638 | 0.8227 | 0.9223 | 0.7231 | 0.4602 | 0.9032 | 0.0172 |
| ALKBH5 | 0.8347 | 0.8834 | 0.7859 | 0.8389 | 0.9379 | 0.7399 | 0.4669 | 0.914 | 0.0198 |
| CAPRIN1 | 0.7543 | 0.7985 | 0.7101 | 0.797 | 0.8997 | 0.6942 | 0.4576 | 0.8801 | 0.0352 |
| EIF4A3 | 0.6732 | 0.7024 | 0.644 | 0.7707 | 0.8629 | 0.6785 | 0.4499 | 0.8381 | 0.0616 |

| | | | | | | | | | |
|---|---|---|---|---|---|---|---|---|---|
| FMRP | 0.8063 | 0.8628 | 0.7499 | 0.8168 | 0.926 | 0.7077 | 0.4644 | 0.9045 | 0.0244 |
| FOX2 | 0.4187 | 0.2934 | 0.544 | 0.6085 | 0.5855 | 0.6315 | 0.3798 | 0.5808 | 0.1789 |
| FUS | 0.9077 | 0.9424 | 0.873 | 0.8996 | 0.9671 | 0.832 | 0.4699 | 0.9274 | 0.0125 |
| FXR1 | 0.9619 | 0.981 | 0.9428 | 0.938 | 0.9842 | 0.8918 | 0.8109 | 0.9793 | 0.6425 |
| IGF2BP1 | 0.6746 | 0.6818 | 0.6674 | 0.7966 | 0.8715 | 0.7217 | 0.4425 | 0.8388 | 0.0463 |
| IGF2BP2 | 0.6852 | 0.7026 | 0.6679 | 0.7996 | 0.8764 | 0.7228 | 0.4422 | 0.8267 | 0.0577 |
| IGF2BP3 | 0.6437 | 0.6455 | 0.6419 | 0.7777 | 0.8527 | 0.7028 | 0.4312 | 0.8425 | 0.0199 |
| LIN28A | 0.7984 | 0.8515 | 0.7453 | 0.8156 | 0.9218 | 0.7094 | 0.4618 | 0.9096 | 0.014 |
| LIN28B | 0.7866 | 0.8579 | 0.7152 | 0.7682 | 0.902 | 0.6344 | 0.514 | 0.9027 | 0.1252 |
| PTB | 0.7392 | 0.7705 | 0.7079 | 0.7897 | 0.8878 | 0.6916 | 0.4467 | 0.8768 | 0.0166 |
| TDP43 | 0.7395 | 0.7981 | 0.6808 | 0.7941 | 0.905 | 0.6832 | 0.7046 | 0.9307 | 0.4784 |
| TIA1 | 0.7808 | 0.8332 | 0.7285 | 0.818 | 0.9209 | 0.7151 | 0.6533 | 0.9214 | 0.3852 |
| TIAL1 | 0.7645 | 0.8198 | 0.7092 | 0.811 | 0.9123 | 0.7097 | 0.6979 | 0.9298 | 0.466 |
| WTAP | 0.8049 | 0.8619 | 0.7478 | 0.8125 | 0.9228 | 0.7023 | 0.4767 | 0.8953 | 0.058 |
| ZC3H7B | 0.6324 | 0.6707 | 0.5941 | 0.7347 | 0.8346 | 0.6348 | 0.4318 | 0.8424 | 0.0212 |
| Avg | 0.748 | 0.7793 | 0.7168 | 0.8005 | 0.8891 | 0.7119 | 0.5085 | 0.876 | 0.1411 |

**Table S8.** 19 datasets performance comparison of CPBFCN with the parameter: 101/201/501, HNML_0.3, and FCNA.

| Data | 101, HNML-0.3, FCNA | | | 201, HNML-0.3, FCNA | | | 501, HNML-0.3, FCNA | | |
|---|---|---|---|---|---|---|---|---|---|
| | miou | iou_0 | iou_1 | miou | iou_0 | iou_1 | miou | iou_0 | iou_1 |
| AGO2 | 0.8058 | 0.8486 | 0.7629 | 0.8235 | 0.9222 | 0.7247 | 0.4587 | 0.9105 | 0.007 |
| ALKBH5 | 0.8404 | 0.8869 | 0.7938 | 0.8409 | 0.9398 | 0.742 | 0.4661 | 0.9164 | 0.0158 |
| CAPRIN1 | 0.7564 | 0.7984 | 0.7144 | 0.7974 | 0.8986 | 0.6961 | 0.4536 | 0.8837 | 0.0235 |
| EIF4A3 | 0.6708 | 0.6968 | 0.6449 | 0.7713 | 0.8637 | 0.679 | 0.4439 | 0.874 | 0.0138 |
| FMRP | 0.8066 | 0.8633 | 0.7499 | 0.8177 | 0.9263 | 0.709 | 0.4619 | 0.9217 | 0.0021 |
| FOX2 | 0.3973 | 0.2527 | 0.542 | 0.6138 | 0.5971 | 0.6304 | 0.3899 | 0.5906 | 0.1892 |
| FUS | 0.9072 | 0.9424 | 0.8719 | 0.9008 | 0.9676 | 0.8339 | 0.469 | 0.9369 | 0.0012 |
| FXR1 | 0.9659 | 0.983 | 0.9487 | 0.9398 | 0.9847 | 0.8949 | 0.8162 | 0.9806 | 0.6519 |
| IGF2BP1 | 0.6739 | 0.681 | 0.6668 | 0.7971 | 0.871 | 0.7232 | 0.4361 | 0.8461 | 0.0261 |
| IGF2BP2 | 0.6875 | 0.7043 | 0.6708 | 0.8005 | 0.8776 | 0.7233 | 0.4366 | 0.8653 | 0.0079 |
| IGF2BP3 | 0.6423 | 0.648 | 0.6366 | 0.7784 | 0.853 | 0.7038 | 0.4298 | 0.849 | 0.0106 |
| LIN28A | 0.7984 | 0.8513 | 0.7455 | 0.8174 | 0.9227 | 0.712 | 0.461 | 0.9106 | 0.0114 |
| LIN28B | 0.785 | 0.8566 | 0.7135 | 0.7725 | 0.9039 | 0.6412 | 0.4566 | 0.8887 | 0.0246 |
| PTB | 0.7381 | 0.7709 | 0.7053 | 0.7921 | 0.8896 | 0.6947 | 0.4466 | 0.8763 | 0.0168 |
| TDP43 | 0.7402 | 0.8002 | 0.6802 | 0.7974 | 0.906 | 0.6888 | 0.7585 | 0.946 | 0.5711 |
| TIA1 | 0.7872 | 0.8388 | 0.7356 | 0.8193 | 0.9212 | 0.7174 | 0.6721 | 0.9347 | 0.4096 |
| TIAL1 | 0.7638 | 0.8178 | 0.7097 | 0.8128 | 0.9122 | 0.7134 | 0.7191 | 0.9369 | 0.5013 |
| WTAP | 0.8051 | 0.8608 | 0.7493 | 0.8155 | 0.9253 | 0.7056 | 0.4766 | 0.8969 | 0.0563 |
| ZC3H7B | 0.6361 | 0.6687 | 0.6035 | 0.7381 | 0.8367 | 0.6395 | 0.4323 | 0.8333 | 0.0313 |
| Avg | 0.7478 | 0.7774 | 0.7182 | 0.8024 | 0.8905 | 0.7144 | 0.5097 | 0.8841 | 0.1353 |

**Table S9.** 19 datasets performance comparison of CPBFCN with the parameter: 101/201/501, HNML_0.5, and FCN.

| Data | 101, HNML-0.5, FCN | | | 201, HNML-0.5, FCN | | | 501, HNML-0.5, FCN | | |
|---|---|---|---|---|---|---|---|---|---|
| | miou | iou_0 | iou_1 | miou | iou_0 | iou_1 | miou | iou_0 | iou_1 |
| AGO2 | 0.8077 | 0.8498 | 0.7657 | 0.8223 | 0.9218 | 0.7228 | 0.4583 | 0.9051 | 0.0116 |
| ALKBH5 | 0.8403 | 0.8873 | 0.7934 | 0.8378 | 0.9375 | 0.7381 | 0.4659 | 0.9128 | 0.019 |

| | | | | | | | | | |
|---|---|---|---|---|---|---|---|---|---|
| CAPRIN1 | 0.7621 | 0.8014 | 0.7229 | 0.7956 | 0.8969 | 0.6943 | 0.4691 | 0.8593 | 0.0789 |
| EIF4A3 | 0.6782 | 0.7025 | 0.6538 | 0.7719 | 0.8633 | 0.6805 | 0.7096 | 0.9136 | 0.5056 |
| FMRP | 0.8101 | 0.864 | 0.7562 | 0.8164 | 0.9249 | 0.7078 | 0.6159 | 0.9328 | 0.299 |
| FOX2 | 0.4218 | 0.3189 | 0.5248 | 0.6212 | 0.5941 | 0.6483 | 0.3796 | 0.6015 | 0.1577 |
| FUS | 0.9074 | 0.9423 | 0.8724 | 0.8982 | 0.9667 | 0.8297 | 0.6472 | 0.948 | 0.3463 |
| FXR1 | 0.9607 | 0.9805 | 0.941 | 0.9413 | 0.9848 | 0.8978 | 0.8046 | 0.9791 | 0.63 |
| IGF2BP1 | 0.6933 | 0.6954 | 0.6912 | 0.7983 | 0.8713 | 0.7253 | 0.6986 | 0.8999 | 0.4972 |
| IGF2BP2 | 0.7063 | 0.7137 | 0.6989 | 0.8002 | 0.8745 | 0.7259 | 0.679 | 0.8952 | 0.4628 |
| IGF2BP3 | 0.6601 | 0.6619 | 0.6584 | 0.7804 | 0.8529 | 0.708 | 0.5865 | 0.8529 | 0.3201 |
| LIN28A | 0.8023 | 0.8534 | 0.7512 | 0.8157 | 0.9215 | 0.7099 | 0.6765 | 0.933 | 0.42 |
| LIN28B | 0.7887 | 0.859 | 0.7184 | 0.7717 | 0.9033 | 0.6402 | 0.6361 | 0.9145 | 0.3578 |
| PTB | 0.7422 | 0.7733 | 0.711 | 0.7923 | 0.8884 | 0.6961 | 0.5424 | 0.8985 | 0.1864 |
| TDP43 | 0.749 | 0.805 | 0.6929 | 0.7961 | 0.904 | 0.6882 | 0.7088 | 0.9294 | 0.4882 |
| TIA1 | 0.7914 | 0.8407 | 0.742 | 0.8168 | 0.9196 | 0.714 | 0.649 | 0.9233 | 0.3747 |
| TIAL1 | 0.7704 | 0.8212 | 0.7196 | 0.8122 | 0.9115 | 0.7129 | 0.6944 | 0.9298 | 0.4589 |
| WTAP | 0.8057 | 0.8611 | 0.7504 | 0.8129 | 0.9217 | 0.7042 | 0.4838 | 0.8964 | 0.0711 |
| ZC3H7B | 0.6559 | 0.6829 | 0.6289 | 0.739 | 0.8355 | 0.6424 | 0.4364 | 0.8308 | 0.042 |
| Avg Value | 0.7555 | 0.785 | 0.726 | 0.8021 | 0.8892 | 0.7151 | 0.5969 | 0.8924 | 0.3014 |

**Table S10.** 19 datasets performance comparison of CPBFCN with the parameter: 101/201/501, HNML_0.5, and FCNA.

| Data | 101, HNML-0.5, FCNA | | | 201, HNML-0.5, FCNA | | | 501, HNML-0.5, FCNA | | |
|---|---|---|---|---|---|---|---|---|---|
| | miou | iou_0 | iou_1 | miou | iou_0 | iou_1 | miou | iou_0 | iou_1 |
| AGO2 | 0.8073 | 0.8494 | 0.7652 | 0.8231 | 0.9222 | 0.724 | 0.4592 | 0.8922 | 0.0261 |
| ALKBH5 | 0.8378 | 0.8854 | 0.7902 | 0.8407 | 0.9389 | 0.7424 | 0.4657 | 0.9212 | 0.0103 |
| CAPRIN1 | 0.7608 | 0.8027 | 0.7189 | 0.7983 | 0.8992 | 0.6973 | 0.4567 | 0.8897 | 0.0238 |
| EIF4A3 | 0.6781 | 0.703 | 0.6531 | 0.7726 | 0.8636 | 0.6816 | 0.7053 | 0.9147 | 0.4959 |
| FMRP | 0.8101 | 0.8644 | 0.7559 | 0.8166 | 0.9258 | 0.7075 | 0.5592 | 0.9212 | 0.1971 |
| FOX2 | 0.4243 | 0.2923 | 0.5563 | 0.6223 | 0.5988 | 0.6457 | 0.3889 | 0.5385 | 0.2392 |
| FUS | 0.9073 | 0.9424 | 0.8723 | 0.8985 | 0.9667 | 0.8304 | 0.748 | 0.9606 | 0.5353 |
| FXR1 | 0.9592 | 0.9798 | 0.9387 | 0.9376 | 0.984 | 0.8912 | 0.8149 | 0.9798 | 0.65 |
| IGF2BP1 | 0.6932 | 0.698 | 0.6883 | 0.799 | 0.8706 | 0.7275 | 0.6326 | 0.8879 | 0.3772 |
| IGF2BP2 | 0.7062 | 0.7138 | 0.6986 | 0.8023 | 0.877 | 0.7276 | 0.6793 | 0.8975 | 0.4611 |
| IGF2BP3 | 0.6602 | 0.6585 | 0.6618 | 0.7817 | 0.8537 | 0.7096 | 0.5685 | 0.8715 | 0.2656 |
| LIN28A | 0.802 | 0.8528 | 0.7513 | 0.8166 | 0.922 | 0.7111 | 0.6265 | 0.9293 | 0.3237 |
| LIN28B | 0.7887 | 0.8593 | 0.7182 | 0.7729 | 0.9026 | 0.6432 | 0.6523 | 0.9176 | 0.3871 |
| PTB | 0.742 | 0.773 | 0.711 | 0.7931 | 0.8889 | 0.6974 | 0.6327 | 0.9135 | 0.3519 |
| TDP43 | 0.7496 | 0.8072 | 0.6919 | 0.7976 | 0.9044 | 0.6907 | 0.748 | 0.9421 | 0.5538 |
| TIA1 | 0.7896 | 0.8407 | 0.7384 | 0.8195 | 0.9206 | 0.7183 | 0.6674 | 0.9275 | 0.4074 |
| TIAL1 | 0.7691 | 0.8202 | 0.7179 | 0.8132 | 0.9115 | 0.7149 | 0.725 | 0.9373 | 0.5126 |
| WTAP | 0.8056 | 0.8608 | 0.7504 | 0.8187 | 0.9273 | 0.7102 | 0.4921 | 0.9033 | 0.0809 |
| ZC3H7B | 0.6548 | 0.6856 | 0.6241 | 0.7408 | 0.8366 | 0.645 | 0.4836 | 0.8066 | 0.1606 |
| Avg Value | 0.755 | 0.7836 | 0.7264 | 0.8034 | 0.8902 | 0.7166 | 0.6056 | 0.8922 | 0.3189 |

**Table S11.** 19 datasets performance comparison of CPBFCN with the parameter: 101/201/501,

HNML_0.7, and FCN.

| Data | 101, HNML-0.7, FCN | | | 201, HNML-0.7, FCN | | | 501, HNML-0.7, FCN | | |
|---|---|---|---|---|---|---|---|---|---|
| | miou | iou_0 | iou_1 | miou | iou_0 | iou_1 | miou | iou_0 | iou_1 |
| AGO2 | 0.808 | 0.8491 | 0.7669 | 0.8217 | 0.9213 | 0.7221 | 0.6447 | 0.9159 | 0.3734 |
| ALKBH5 | 0.8397 | 0.8865 | 0.7929 | 0.8385 | 0.9383 | 0.7386 | 0.4726 | 0.8977 | 0.0475 |
| CAPRIN1 | 0.7635 | 0.8015 | 0.7255 | 0.7945 | 0.8961 | 0.6929 | 0.5799 | 0.8878 | 0.2721 |
| EIF4A3 | 0.6799 | 0.7027 | 0.6571 | 0.7712 | 0.8629 | 0.6794 | 0.713 | 0.9142 | 0.5118 |
| FMRP | 0.8101 | 0.8646 | 0.7556 | 0.8138 | 0.9237 | 0.7039 | 0.673 | 0.9338 | 0.4122 |
| FOX2 | 0.4383 | 0.3321 | 0.5445 | 0.6238 | 0.6048 | 0.6428 | 0.3998 | 0.6018 | 0.1979 |
| FUS | 0.907 | 0.9418 | 0.8722 | 0.8972 | 0.9665 | 0.828 | 0.7629 | 0.9619 | 0.564 |
| FXR1 | 0.9617 | 0.981 | 0.9425 | 0.9347 | 0.9832 | 0.8862 | 0.8042 | 0.9786 | 0.6297 |
| IGF2BP1 | 0.7017 | 0.7004 | 0.703 | 0.798 | 0.87 | 0.726 | 0.6935 | 0.902 | 0.4849 |
| IGF2BP2 | 0.7127 | 0.7169 | 0.7086 | 0.8007 | 0.8755 | 0.726 | 0.671 | 0.8848 | 0.4572 |
| IGF2BP3 | 0.6689 | 0.6671 | 0.6707 | 0.7805 | 0.8525 | 0.7086 | 0.6879 | 0.8874 | 0.4883 |
| LIN28A | 0.8023 | 0.853 | 0.7516 | 0.8138 | 0.9204 | 0.7073 | 0.6949 | 0.9371 | 0.4527 |
| LIN28B | 0.7878 | 0.857 | 0.7186 | 0.7702 | 0.9009 | 0.6395 | 0.6382 | 0.9095 | 0.3669 |
| PTB | 0.743 | 0.7717 | 0.7143 | 0.7914 | 0.8864 | 0.6964 | 0.701 | 0.9156 | 0.4864 |
| TDP43 | 0.7517 | 0.8053 | 0.6981 | 0.7952 | 0.9027 | 0.6877 | 0.6923 | 0.9263 | 0.4584 |
| TIA1 | 0.793 | 0.8417 | 0.7443 | 0.816 | 0.9189 | 0.7131 | 0.6376 | 0.9168 | 0.3583 |
| TIAL1 | 0.7714 | 0.8213 | 0.7214 | 0.8093 | 0.91 | 0.7085 | 0.6793 | 0.9213 | 0.4373 |
| WTAP | 0.8081 | 0.8628 | 0.7534 | 0.8143 | 0.9244 | 0.7042 | 0.5137 | 0.9065 | 0.1209 |
| ZC3H7B | 0.6616 | 0.6848 | 0.6384 | 0.7386 | 0.8353 | 0.6418 | 0.6378 | 0.8581 | 0.4175 |
| Avg Value | 0.7584 | 0.7864 | 0.7305 | 0.8012 | 0.8891 | 0.7133 | 0.6472 | 0.8977 | 0.3967 |

**Table S12.** 19 datasets performance comparison of CPBFCN with the parameter: 101/201/501, HNML_0.7, and FCNA.

| Data | 101, HNML-0.7, FCN | | | 201, HNML-0.7, FCN | | | 501, HNML-0.7, FCN | | |
|---|---|---|---|---|---|---|---|---|---|
| | miou | iou_0 | iou_1 | miou | iou_0 | iou_1 | miou | iou_0 | iou_1 |
| AGO2 | 0.8076 | 0.8491 | 0.766 | 0.8213 | 0.9209 | 0.7216 | 0.6553 | 0.9215 | 0.3891 |
| ALKBH5 | 0.8399 | 0.8874 | 0.7923 | 0.8408 | 0.9394 | 0.7422 | 0.4789 | 0.8969 | 0.0609 |
| CAPRIN1 | 0.7636 | 0.8018 | 0.7255 | 0.7972 | 0.8986 | 0.6957 | 0.603 | 0.8999 | 0.3062 |
| EIF4A3 | 0.68 | 0.7027 | 0.6573 | 0.7718 | 0.8625 | 0.6811 | 0.7053 | 0.9169 | 0.4937 |
| FMRP | 0.8099 | 0.864 | 0.7557 | 0.8143 | 0.9239 | 0.7047 | 0.6887 | 0.9358 | 0.4416 |
| FOX2 | 0.4403 | 0.3254 | 0.5552 | 0.6212 | 0.6066 | 0.6358 | 0.4179 | 0.5339 | 0.3019 |
| FUS | 0.9062 | 0.9413 | 0.8712 | 0.8953 | 0.9652 | 0.8253 | 0.7565 | 0.9629 | 0.5501 |
| FXR1 | 0.9608 | 0.9804 | 0.9412 | 0.9407 | 0.9847 | 0.8966 | 0.8128 | 0.9791 | 0.6465 |
| IGF2BP1 | 0.7014 | 0.6993 | 0.7035 | 0.7987 | 0.8709 | 0.7265 | 0.6846 | 0.8986 | 0.4706 |
| IGF2BP2 | 0.712 | 0.7176 | 0.7064 | 0.8002 | 0.8762 | 0.7243 | 0.6987 | 0.9053 | 0.4921 |
| IGF2BP3 | 0.6692 | 0.6667 | 0.6718 | 0.7816 | 0.8528 | 0.7103 | 0.6624 | 0.8837 | 0.4411 |
| LIN28A | 0.8022 | 0.8538 | 0.7506 | 0.8144 | 0.9198 | 0.709 | 0.6892 | 0.933 | 0.4454 |
| LIN28B | 0.7885 | 0.8579 | 0.7191 | 0.7711 | 0.9004 | 0.6417 | 0.6539 | 0.9121 | 0.3957 |
| PTB | 0.7435 | 0.7734 | 0.7137 | 0.7926 | 0.8884 | 0.6968 | 0.6995 | 0.9165 | 0.4825 |
| TDP43 | 0.751 | 0.8062 | 0.6957 | 0.797 | 0.9047 | 0.6892 | 0.7427 | 0.9415 | 0.5439 |
| TIA1 | 0.7927 | 0.8401 | 0.7452 | 0.819 | 0.9204 | 0.7176 | 0.6527 | 0.9228 | 0.3827 |
| TIAL1 | 0.7719 | 0.8214 | 0.7225 | 0.8111 | 0.9103 | 0.7119 | 0.7093 | 0.9343 | 0.4844 |
| WTAP | 0.8046 | 0.859 | 0.7501 | 0.817 | 0.9261 | 0.7079 | 0.5207 | 0.9041 | 0.1372 |
| ZC3H7B | 0.6614 | 0.6869 | 0.6359 | 0.7394 | 0.8352 | 0.6436 | 0.585 | 0.8325 | 0.3375 |
| Avg Value | 0.7582 | 0.786 | 0.7305 | 0.8024 | 0.8898 | 0.7148 | 0.6535 | 0.8964 | 0.4107 |

**Table S13.** 37 datasets performance comparison of CPBFCN, CPBFCN_1 and CPBFCN_2 with the parameter: 101, HNML_0.7.

| | CPBFCN_1 | | | CPBFCN_2 | | | CPBFCN | | |
|---|---|---|---|---|---|---|---|---|---|
| | miou | iou_0 | iou_1 | miou | iou_0 | iou_1 | miou | iou_0 | iou_1 |
| AGO1 | 0.7606 | 0.8443 | 0.6769 | 0.8984 | 0.9385 | 0.8583 | 0.8987 | 0.9386 | 0.8589 |
| AGO2 | 0.6472 | 0.7018 | 0.5926 | 0.8072 | 0.8482 | 0.7661 | 0.808 | 0.8491 | 0.7669 |
| AGO3 | 0.7557 | 0.8651 | 0.6463 | 0.9378 | 0.9672 | 0.9083 | 0.9385 | 0.9677 | 0.9094 |
| ALKBH5 | 0.4432 | 0.5501 | 0.3362 | 0.843 | 0.8885 | 0.7974 | 0.8397 | 0.8865 | 0.7929 |
| AUF1 | 0.9066 | 0.9425 | 0.8706 | 0.9624 | 0.9769 | 0.9479 | 0.9637 | 0.9779 | 0.9496 |
| C17ORF85 | 0.511 | 0.6464 | 0.3757 | 0.8736 | 0.9201 | 0.8272 | 0.874 | 0.9201 | 0.8279 |
| C22ORF28 | 0.5971 | 0.6643 | 0.5299 | 0.8242 | 0.8682 | 0.7801 | 0.8251 | 0.8681 | 0.7821 |
| CAPRIN1 | 0.5746 | 0.623 | 0.5262 | 0.7613 | 0.8 | 0.7227 | 0.7635 | 0.8015 | 0.7255 |
| DGCR8 | 0.7207 | 0.8007 | 0.6407 | 0.8704 | 0.9142 | 0.8266 | 0.8703 | 0.9141 | 0.8266 |
| EIF4A3 | 0.5737 | 0.5897 | 0.5577 | 0.6805 | 0.7051 | 0.6558 | 0.6799 | 0.7027 | 0.6571 |
| EWSR1 | 0.8184 | 0.8831 | 0.7536 | 0.9358 | 0.9593 | 0.9123 | 0.9357 | 0.9593 | 0.9122 |
| FMRP | 0.6712 | 0.7483 | 0.5942 | 0.8096 | 0.8644 | 0.7548 | 0.8101 | 0.8646 | 0.7556 |
| FOX2 | 0.3862 | 0.2931 | 0.4794 | 0.4369 | 0.3137 | 0.56 | 0.4383 | 0.3321 | 0.5445 |
| FUS | 0.7289 | 0.8186 | 0.6391 | 0.9057 | 0.941 | 0.8705 | 0.907 | 0.9418 | 0.8722 |
| FXR1 | 0.886 | 0.9433 | 0.8286 | 0.9611 | 0.9805 | 0.9416 | 0.9617 | 0.981 | 0.9425 |
| FXR2 | 0.7624 | 0.8632 | 0.6616 | 0.943 | 0.9697 | 0.9164 | 0.9424 | 0.9694 | 0.9154 |
| HNRNPC | 0.7883 | 0.8492 | 0.7274 | 0.8973 | 0.93 | 0.8645 | 0.8986 | 0.931 | 0.8663 |
| HUR | 0.6881 | 0.7197 | 0.6565 | 0.8465 | 0.8692 | 0.8238 | 0.8456 | 0.8695 | 0.8217 |
| IGF2BP1 | 0.6031 | 0.5939 | 0.6123 | 0.7035 | 0.7021 | 0.705 | 0.7017 | 0.7004 | 0.703 |
| IGF2BP2 | 0.5949 | 0.5937 | 0.5961 | 0.7131 | 0.7191 | 0.707 | 0.7127 | 0.7169 | 0.7086 |
| IGF2BP3 | 0.5649 | 0.5503 | 0.5795 | 0.6707 | 0.6666 | 0.6747 | 0.6689 | 0.6671 | 0.6707 |
| LIN28A | 0.6473 | 0.7161 | 0.5785 | 0.802 | 0.8529 | 0.7511 | 0.8023 | 0.853 | 0.7516 |
| LIN28B | 0.6791 | 0.7751 | 0.5831 | 0.7877 | 0.8572 | 0.7183 | 0.7878 | 0.857 | 0.7186 |
| METTL3 | 0.5682 | 0.6628 | 0.4736 | 0.8481 | 0.8973 | 0.799 | 0.8471 | 0.8961 | 0.7981 |
| MOV10 | 0.6492 | 0.7175 | 0.5809 | 0.841 | 0.877 | 0.805 | 0.8416 | 0.8774 | 0.8057 |
| PTB | 0.6195 | 0.6562 | 0.5829 | 0.7434 | 0.7737 | 0.713 | 0.743 | 0.7717 | 0.7143 |
| PUM2 | 0.7395 | 0.809 | 0.6701 | 0.8999 | 0.9276 | 0.8721 | 0.9027 | 0.9303 | 0.8751 |
| QKI | 0.7311 | 0.8212 | 0.641 | 0.9288 | 0.953 | 0.9046 | 0.9317 | 0.9547 | 0.9086 |
| SFRS1 | 0.6835 | 0.7042 | 0.6629 | 0.8418 | 0.862 | 0.8217 | 0.8422 | 0.8623 | 0.822 |
| TAF15 | 0.9022 | 0.9436 | 0.8608 | 0.9547 | 0.9738 | 0.9355 | 0.9575 | 0.9753 | 0.9396 |
| TDP43 | 0.5731 | 0.6361 | 0.5101 | 0.7498 | 0.8071 | 0.6925 | 0.7517 | 0.8053 | 0.6981 |
| TIA1 | 0.6107 | 0.6853 | 0.5361 | 0.7942 | 0.8411 | 0.7473 | 0.793 | 0.8417 | 0.7443 |
| TIAL1 | 0.6175 | 0.681 | 0.554 | 0.7738 | 0.8218 | 0.7257 | 0.7714 | 0.8213 | 0.7214 |
| TNRC6 | 0.5947 | 0.7325 | 0.457 | 0.8922 | 0.9336 | 0.8508 | 0.8933 | 0.9349 | 0.8516 |
| U2AF65 | 0.692 | 0.7724 | 0.6116 | 0.838 | 0.8872 | 0.7887 | 0.8374 | 0.8871 | 0.7878 |
| WTAP | 0.4672 | 0.6109 | 0.3235 | 0.8073 | 0.8643 | 0.7504 | 0.8081 | 0.8628 | 0.7534 |
| ZC3H7B | 0.5182 | 0.5453 | 0.4912 | 0.6617 | 0.6869 | 0.6365 | 0.6616 | 0.6848 | 0.6384 |
| Avg Value | 0.6561 | 0.7177 | 0.5946 | 0.8229 | 0.8529 | 0.7928 | 0.8231 | 0.8534 | 0.7929 |

**Table S14.** 37 datasets performance comparison of CPBFCN, CPBFCN_1 and CPBFCN_2 with the parameter: 201, HNML_0.7.

| | CPBFCN_1 | | | CPBFCN_2 | | | CPBFCN | | |
|---|---|---|---|---|---|---|---|---|---|
| | miou | iou_0 | iou_1 | miou | iou_0 | iou_1 | miou | iou_0 | iou_1 |
| AGO1 | 0.7132 | 0.8933 | 0.5331 | 0.8035 | 0.931 | 0.676 | 0.8774 | 0.9599 | 0.795 |
| AGO2 | 0.5796 | 0.7806 | 0.3785 | 0.7263 | 0.8632 | 0.5895 | 0.8217 | 0.9213 | 0.7221 |
| AGO3 | 0.6708 | 0.8943 | 0.4474 | 0.7497 | 0.9242 | 0.5751 | 0.8972 | 0.9716 | 0.8227 |

| | | | | | | | | | |
|---|---|---|---|---|---|---|---|---|---|
| ALKBH5 | 0.4312 | 0.7582 | 0.1041 | 0.4679 | 0.7671 | 0.1687 | 0.8385 | 0.9383 | 0.7386 |
| AUF1 | 0.859 | 0.9535 | 0.7645 | 0.9118 | 0.9718 | 0.8518 | 0.9582 | 0.9869 | 0.9296 |
| C17ORF85 | 0.4644 | 0.7868 | 0.142 | 0.5214 | 0.8034 | 0.2395 | 0.8458 | 0.9475 | 0.744 |
| C22ORF28 | 0.5386 | 0.7522 | 0.3251 | 0.5639 | 0.7692 | 0.3585 | 0.8294 | 0.9288 | 0.73 |
| CAPRIN1 | 0.5515 | 0.7411 | 0.3619 | 0.5981 | 0.7557 | 0.4406 | 0.7945 | 0.8961 | 0.6929 |
| DGCR8 | 0.6772 | 0.8609 | 0.4934 | 0.7845 | 0.9156 | 0.6533 | 0.8661 | 0.9526 | 0.7796 |
| EIF4A3 | 0.6107 | 0.7433 | 0.4781 | 0.7024 | 0.8089 | 0.5958 | 0.7712 | 0.8629 | 0.6794 |
| EWSR1 | 0.7787 | 0.9243 | 0.6332 | 0.8207 | 0.9368 | 0.7046 | 0.9283 | 0.9765 | 0.8802 |
| FMRP | 0.6267 | 0.8261 | 0.4273 | 0.7305 | 0.8789 | 0.5821 | 0.8138 | 0.9237 | 0.7039 |
| FOX2 | 0.3762 | 0.3333 | 0.4191 | 0.5592 | 0.478 | 0.6404 | 0.6238 | 0.6048 | 0.6428 |
| FUS | 0.6633 | 0.8651 | 0.4614 | 0.7767 | 0.9143 | 0.639 | 0.8972 | 0.9665 | 0.828 |
| FXR1 | 0.8223 | 0.9529 | 0.6918 | 0.8705 | 0.9662 | 0.7749 | 0.9347 | 0.9832 | 0.8862 |
| FXR2 | 0.7099 | 0.9048 | 0.5149 | 0.7511 | 0.9202 | 0.582 | 0.915 | 0.9763 | 0.8536 |
| HNRNPC | 0.6833 | 0.8633 | 0.5033 | 0.7637 | 0.9054 | 0.622 | 0.8751 | 0.9536 | 0.7966 |
| HUR | 0.627 | 0.7887 | 0.4653 | 0.7599 | 0.8736 | 0.6463 | 0.8527 | 0.9283 | 0.777 |
| IGF2BP1 | 0.6261 | 0.7405 | 0.5117 | 0.7374 | 0.8195 | 0.6553 | 0.798 | 0.87 | 0.726 |
| IGF2BP2 | 0.6324 | 0.7555 | 0.5093 | 0.7145 | 0.8019 | 0.6271 | 0.8007 | 0.8755 | 0.726 |
| IGF2BP3 | 0.6125 | 0.7232 | 0.5018 | 0.6506 | 0.741 | 0.5602 | 0.7805 | 0.8525 | 0.7086 |
| LIN28A | 0.6098 | 0.8008 | 0.4188 | 0.7383 | 0.8794 | 0.5971 | 0.8138 | 0.9204 | 0.7073 |
| LIN28B | 0.63 | 0.8327 | 0.4273 | 0.6631 | 0.8428 | 0.4834 | 0.7702 | 0.9009 | 0.6395 |
| METTL3 | 0.5463 | 0.7953 | 0.2974 | 0.5849 | 0.8257 | 0.3441 | 0.8482 | 0.944 | 0.7524 |
| MOV10 | 0.6063 | 0.8014 | 0.4112 | 0.6376 | 0.8218 | 0.4533 | 0.8657 | 0.9442 | 0.7872 |
| PTB | 0.5931 | 0.7626 | 0.4236 | 0.7174 | 0.834 | 0.6007 | 0.7914 | 0.8864 | 0.6964 |
| PUM2 | 0.7141 | 0.8775 | 0.5507 | 0.7897 | 0.9161 | 0.6634 | 0.8986 | 0.9617 | 0.8355 |
| QKI | 0.7238 | 0.9002 | 0.5474 | 0.795 | 0.9299 | 0.6601 | 0.921 | 0.9729 | 0.869 |
| SFRS1 | 0.6614 | 0.8147 | 0.5081 | 0.7313 | 0.8552 | 0.6073 | 0.8645 | 0.9354 | 0.7937 |
| TAF15 | 0.8498 | 0.9527 | 0.747 | 0.8993 | 0.9695 | 0.8292 | 0.9384 | 0.9815 | 0.8953 |
| TDP43 | 0.6045 | 0.7786 | 0.4304 | 0.6493 | 0.8167 | 0.4819 | 0.7952 | 0.9027 | 0.6877 |
| TIA1 | 0.6181 | 0.8074 | 0.4287 | 0.6789 | 0.8441 | 0.5138 | 0.816 | 0.9189 | 0.7131 |
| TIAL1 | 0.6399 | 0.813 | 0.4667 | 0.6915 | 0.8405 | 0.5425 | 0.8093 | 0.91 | 0.7085 |
| TNRC6 | 0.5429 | 0.8284 | 0.2574 | 0.6118 | 0.8506 | 0.3729 | 0.8733 | 0.958 | 0.7885 |
| U2AF65 | 0.6864 | 0.8619 | 0.5109 | 0.7451 | 0.8942 | 0.596 | 0.8503 | 0.9429 | 0.7577 |
| WTAP | 0.4713 | 0.7745 | 0.1681 | 0.5089 | 0.7557 | 0.2621 | 0.8143 | 0.9244 | 0.7042 |
| ZC3H7B | 0.5634 | 0.7018 | 0.4249 | 0.5325 | 0.6241 | 0.4408 | 0.7386 | 0.8353 | 0.6418 |
| Avg Value | 0.6302 | 0.8093 | 0.451 | 0.7011 | 0.8445 | 0.5576 | 0.8413 | 0.9221 | 0.7606 |

**Table S15.** 37 datasets performance comparison of CPBFCN, CPBFCN_1 and CPBFCN_2 with the parameter: 501, HNML_0.7.

| | CPBFCN_1 | | | CPBFCN_2 | | | CPBFCN | | |
|---|---|---|---|---|---|---|---|---|---|
| | miou | iou_0 | iou_1 | miou | iou_0 | iou_1 | miou | iou_0 | iou_1 |
| AGO1 | 0.5991 | 0.9219 | 0.2763 | 0.7004 | 0.9539 | 0.4469 | 0.717 | 0.9542 | 0.4798 |
| AGO2 | 0.491 | 0.8627 | 0.1193 | 0.6243 | 0.9074 | 0.3411 | 0.6447 | 0.9159 | 0.3734 |
| AGO3 | 0.5887 | 0.9362 | 0.2411 | 0.6745 | 0.9573 | 0.3916 | 0.678 | 0.9585 | 0.3974 |
| ALKBH5 | 0.4667 | 0.9102 | 0.0231 | 0.47 | 0.9123 | 0.0277 | 0.4726 | 0.8977 | 0.0475 |
| AUF1 | 0.7736 | 0.9652 | 0.5821 | 0.8635 | 0.9819 | 0.7451 | 0.8717 | 0.9828 | 0.7607 |
| C17ORF85 | 0.477 | 0.9031 | 0.051 | 0.4844 | 0.9179 | 0.0509 | 0.4876 | 0.9195 | 0.0558 |
| C22ORF28 | 0.4849 | 0.8858 | 0.0839 | 0.531 | 0.8971 | 0.165 | 0.547 | 0.8942 | 0.1998 |
| CAPRIN1 | 0.4956 | 0.8585 | 0.1327 | 0.5701 | 0.8879 | 0.2523 | 0.5799 | 0.8878 | 0.2721 |
| DGCR8 | 0.5761 | 0.9085 | 0.2436 | 0.6813 | 0.9449 | 0.4177 | 0.7221 | 0.9518 | 0.4924 |
| EIF4A3 | 0.5638 | 0.8483 | 0.2793 | 0.6767 | 0.8964 | 0.4571 | 0.713 | 0.9142 | 0.5118 |
| EWSR1 | 0.6892 | 0.947 | 0.4314 | 0.776 | 0.9669 | 0.5852 | 0.7832 | 0.9671 | 0.5992 |
| FMRP | 0.5371 | 0.8824 | 0.1918 | 0.6434 | 0.9238 | 0.3631 | 0.673 | 0.9338 | 0.4122 |

| | | | | | | | | | |
|---|---|---|---|---|---|---|---|---|---|
| FOX2 | 0.376 | 0.5653 | 0.1867 | 0.388 | 0.5927 | 0.1834 | 0.3998 | 0.6018 | 0.1979 |
| FUS | 0.5548 | 0.8984 | 0.2112 | 0.7036 | 0.9504 | 0.4569 | 0.7629 | 0.9619 | 0.564 |
| FXR1 | 0.7475 | 0.9674 | 0.5276 | 0.7889 | 0.9768 | 0.601 | 0.8042 | 0.9786 | 0.6297 |
| FXR2 | 0.6156 | 0.9403 | 0.291 | 0.7006 | 0.9601 | 0.4411 | 0.6981 | 0.9578 | 0.4384 |
| HNRNPC | 0.6466 | 0.926 | 0.3671 | 0.7747 | 0.9611 | 0.5883 | 0.7794 | 0.9616 | 0.5972 |
| HUR | 0.5395 | 0.855 | 0.224 | 0.6556 | 0.9094 | 0.4018 | 0.6877 | 0.9206 | 0.4548 |
| IGF2BP1 | 0.5649 | 0.8312 | 0.2987 | 0.6766 | 0.8876 | 0.4655 | 0.6935 | 0.902 | 0.4849 |
| IGF2BP2 | 0.559 | 0.8288 | 0.2893 | 0.6612 | 0.8863 | 0.4361 | 0.671 | 0.8848 | 0.4572 |
| IGF2BP3 | 0.5492 | 0.8131 | 0.2854 | 0.6686 | 0.8755 | 0.4617 | 0.6879 | 0.8874 | 0.4883 |
| LIN28A | 0.5428 | 0.8799 | 0.2057 | 0.6405 | 0.9168 | 0.3643 | 0.6949 | 0.9371 | 0.4527 |
| LIN28B | 0.5738 | 0.8924 | 0.2551 | 0.6353 | 0.9091 | 0.3615 | 0.6382 | 0.9095 | 0.3669 |
| METTL3 | 0.5099 | 0.8944 | 0.1254 | 0.5808 | 0.9207 | 0.241 | 0.584 | 0.919 | 0.249 |
| MOV10 | 0.511 | 0.8831 | 0.1389 | 0.6522 | 0.925 | 0.3794 | 0.7002 | 0.9354 | 0.4649 |
| PTB | 0.5126 | 0.8497 | 0.1756 | 0.6651 | 0.9033 | 0.4269 | 0.701 | 0.9156 | 0.4864 |
| PUM2 | 0.644 | 0.9189 | 0.3691 | 0.7426 | 0.9546 | 0.5306 | 0.7474 | 0.9556 | 0.5391 |
| QKI | 0.7035 | 0.9513 | 0.4557 | 0.7509 | 0.9603 | 0.5416 | 0.7598 | 0.963 | 0.5566 |
| SFRS1 | 0.5523 | 0.8747 | 0.2299 | 0.6299 | 0.906 | 0.3538 | 0.6484 | 0.9169 | 0.3799 |
| TAF15 | 0.7564 | 0.9645 | 0.5482 | 0.8148 | 0.9755 | 0.654 | 0.8202 | 0.9761 | 0.6643 |
| TDP43 | 0.5954 | 0.8796 | 0.3113 | 0.6785 | 0.919 | 0.438 | 0.6923 | 0.9263 | 0.4584 |
| TIA1 | 0.5827 | 0.8852 | 0.2801 | 0.6501 | 0.923 | 0.3773 | 0.6376 | 0.9168 | 0.3583 |
| TIAL1 | 0.589 | 0.8881 | 0.29 | 0.6775 | 0.9214 | 0.4335 | 0.6793 | 0.9213 | 0.4373 |
| TNRC6 | 0.5244 | 0.9129 | 0.1359 | 0.5623 | 0.9279 | 0.1967 | 0.5726 | 0.9314 | 0.2138 |
| U2AF65 | 0.6151 | 0.9165 | 0.3136 | 0.7145 | 0.95 | 0.4791 | 0.7169 | 0.9459 | 0.488 |
| WTAP | 0.5065 | 0.8957 | 0.1173 | 0.4974 | 0.8933 | 0.1016 | 0.5137 | 0.9065 | 0.1209 |
| ZC3H7B | 0.4872 | 0.8136 | 0.1607 | 0.6312 | 0.8591 | 0.4033 | 0.6378 | 0.8581 | 0.4175 |
| Avg Value | 0.5703 | 0.8853 | 0.2554 | 0.6551 | 0.9166 | 0.3936 | 0.6708 | 0.9208 | 0.4208 |

**Table S16.** 37 datasets performance comparison of CPBFCN with the parameter: 101/201/501, HNML_0.7.

| | 101 | | | 201 | | | 501 | | |
|---|---|---|---|---|---|---|---|---|---|
| | miou | iou_0 | iou_1 | miou | iou_0 | iou_1 | miou | iou_0 | iou_1 |
| AGO1 | 0.8987 | 0.9386 | 0.8589 | 0.8774 | 0.9599 | 0.795 | 0.717 | 0.9542 | 0.4798 |
| AGO2 | 0.808 | 0.8491 | 0.7669 | 0.8217 | 0.9213 | 0.7221 | 0.6447 | 0.9159 | 0.3734 |
| AGO3 | 0.9385 | 0.9677 | 0.9094 | 0.8972 | 0.9716 | 0.8227 | 0.678 | 0.9585 | 0.3974 |
| ALKBH5 | 0.8397 | 0.8865 | 0.7929 | 0.8385 | 0.9383 | 0.7386 | 0.4726 | 0.8977 | 0.0475 |
| AUF1 | 0.9637 | 0.9779 | 0.9496 | 0.9582 | 0.9869 | 0.9296 | 0.8717 | 0.9828 | 0.7607 |
| C17ORF85 | 0.874 | 0.9201 | 0.8279 | 0.8458 | 0.9475 | 0.744 | 0.4876 | 0.9195 | 0.0558 |
| C22ORF28 | 0.8251 | 0.8681 | 0.7821 | 0.8294 | 0.9288 | 0.73 | 0.547 | 0.8942 | 0.1998 |
| CAPRIN1 | 0.7635 | 0.8015 | 0.7255 | 0.7945 | 0.8961 | 0.6929 | 0.5799 | 0.8878 | 0.2721 |
| DGCR8 | 0.8703 | 0.9141 | 0.8266 | 0.8661 | 0.9526 | 0.7796 | 0.7221 | 0.9518 | 0.4924 |
| EIF4A3 | **0.6799** | **0.7027** | **0.6571** | **0.7712** | **0.8629** | **0.6794** | 0.713 | 0.9142 | 0.5118 |
| EWSR1 | 0.9357 | 0.9593 | 0.9122 | 0.9283 | 0.9765 | 0.8802 | 0.7832 | 0.9671 | 0.5992 |
| FMRP | 0.8101 | 0.8646 | 0.7556 | 0.8138 | 0.9237 | 0.7039 | 0.673 | 0.9338 | 0.4122 |
| FOX2 | **0.4383** | **0.3321** | **0.5445** | **0.6238** | **0.6048** | **0.6428** | 0.3998 | 0.6018 | 0.1979 |
| FUS | 0.907 | 0.9418 | 0.8722 | 0.8972 | 0.9665 | 0.828 | 0.7629 | 0.9619 | 0.564 |
| FXR1 | 0.9617 | 0.981 | 0.9425 | 0.9347 | 0.9832 | 0.8862 | 0.8042 | 0.9786 | 0.6297 |
| FXR2 | 0.9424 | 0.9694 | 0.9154 | 0.915 | 0.9763 | 0.8536 | 0.6981 | 0.9578 | 0.4384 |
| HNRNPC | 0.8986 | 0.931 | 0.8663 | 0.8751 | 0.9536 | 0.7966 | 0.7794 | 0.9616 | 0.5972 |
| HUR | 0.8456 | 0.8695 | 0.8217 | 0.8527 | 0.9283 | 0.777 | 0.6877 | 0.9206 | 0.4548 |
| IGF2BP1 | **0.7017** | **0.7004** | **0.703** | **0.798** | **0.87** | **0.726** | 0.6935 | 0.902 | 0.4849 |

| | | | | | | | | | |
|---|---|---|---|---|---|---|---|---|---|
| IGF2BP2 | **0.7127** | **0.7169** | **0.7086** | **0.8007** | **0.8755** | **0.726** | 0.671 | 0.8848 | 0.4572 |
| IGF2BP3 | **0.6689** | **0.6671** | **0.6707** | **0.7805** | **0.8525** | **0.7086** | 0.6879 | 0.8874 | 0.4883 |
| LIN28A | 0.8023 | 0.853 | 0.7516 | 0.8138 | 0.9204 | 0.7073 | 0.6949 | 0.9371 | 0.4527 |
| LIN28B | 0.7878 | 0.857 | 0.7186 | 0.7702 | 0.9009 | 0.6395 | 0.6382 | 0.9095 | 0.3669 |
| METTL3 | 0.8471 | 0.8961 | 0.7981 | 0.8482 | 0.944 | 0.7524 | 0.584 | 0.919 | 0.249 |
| MOV10 | 0.8416 | 0.8774 | 0.8057 | 0.8657 | 0.9442 | 0.7872 | 0.7002 | 0.9354 | 0.4649 |
| PTB | 0.743 | 0.7717 | 0.7143 | 0.7914 | 0.8864 | 0.6964 | 0.701 | 0.9156 | 0.4864 |
| PUM2 | 0.9027 | 0.9303 | 0.8751 | 0.8986 | 0.9617 | 0.8355 | 0.7474 | 0.9556 | 0.5391 |
| QKI | 0.9317 | 0.9547 | 0.9086 | 0.921 | 0.9729 | 0.869 | 0.7598 | 0.963 | 0.5566 |
| SFRS1 | 0.8422 | 0.8623 | 0.822 | 0.8645 | 0.9354 | 0.7937 | 0.6484 | 0.9169 | 0.3799 |
| TAF15 | 0.9575 | 0.9753 | 0.9396 | 0.9384 | 0.9815 | 0.8953 | 0.8202 | 0.9761 | 0.6643 |
| TDP43 | 0.7517 | 0.8053 | 0.6981 | 0.7952 | 0.9027 | 0.6877 | 0.6923 | 0.9263 | 0.4584 |
| TIA1 | 0.793 | 0.8417 | 0.7443 | 0.816 | 0.9189 | 0.7131 | 0.6376 | 0.9168 | 0.3583 |
| TIAL1 | 0.7714 | 0.8213 | 0.7214 | 0.8093 | 0.91 | 0.7085 | 0.6793 | 0.9213 | 0.4373 |
| TNRC6 | 0.8933 | 0.9349 | 0.8516 | 0.8733 | 0.958 | 0.7885 | 0.5726 | 0.9314 | 0.2138 |
| U2AF65 | 0.8374 | 0.8871 | 0.7878 | 0.8503 | 0.9429 | 0.7577 | 0.7169 | 0.9459 | 0.488 |
| WTAP | 0.8081 | 0.8628 | 0.7534 | 0.8143 | 0.9244 | 0.7042 | 0.5137 | 0.9065 | 0.1209 |
| ZC3H7B | **0.6616** | **0.6848** | **0.6384** | **0.7386** | **0.8353** | **0.6418** | 0.6378 | 0.8581 | 0.4175 |
| Avg Value | 0.8231 | 0.8534 | 0.7929 | 0.8413 | 0.9221 | 0.7606 | 0.6708 | 0.9208 | 0.4208 |

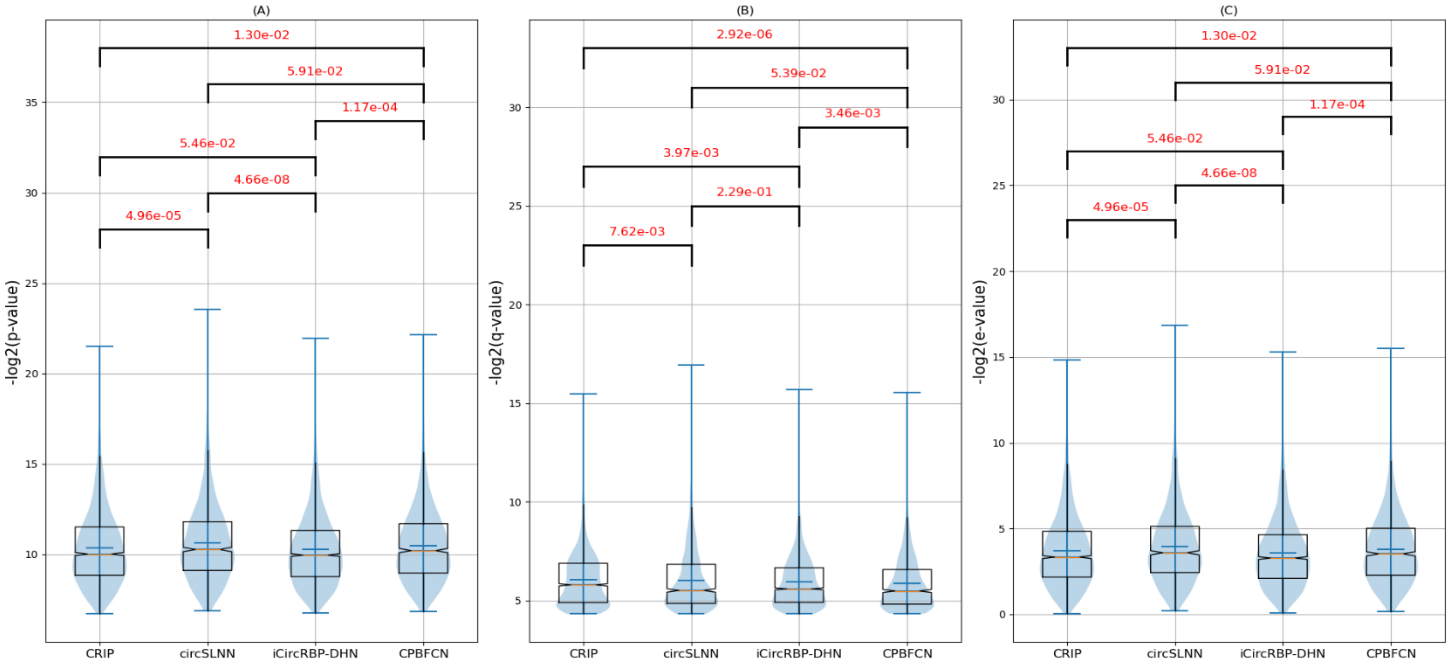


**Figure S1**. Three indicators comparison of four motif discovery methods CPBFCN, CRIP, circSLNN, iCricRBP-DHN. From left to right are -log2(p-value) (A), -log2(q-value) (B), -log2(e-value) (C).

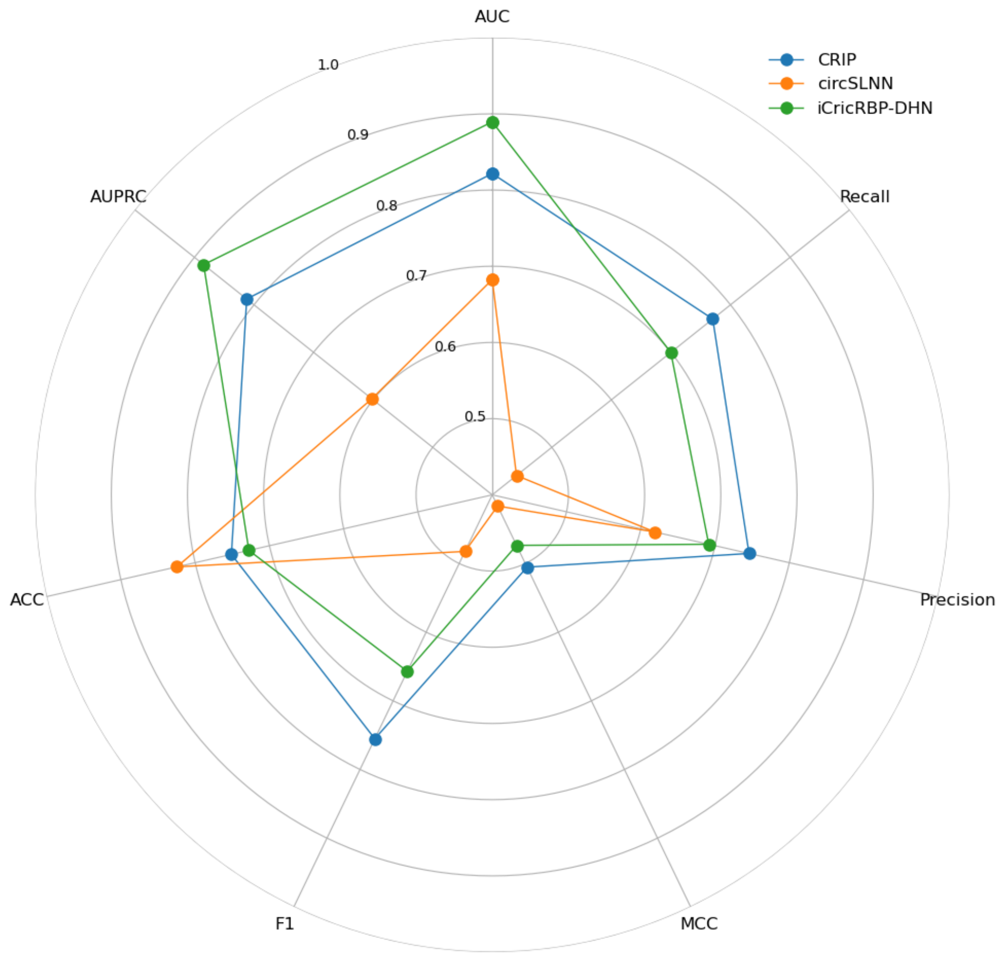


**Figure S2.** Performance comparison of three baseline models.

Table S17. Top5 Motif found by CPBFCN in each dataset. Here, the sum of p-value, e-value and q-value was used to rank all the matched motifs (ascending order).

| Protein Name | Motif | | | | |
|---|---|---|---|---|---|
| AGO1 | PTBP1 | CPEB4 | CPEB2 | PCBP1 | ENOX1 |
| | 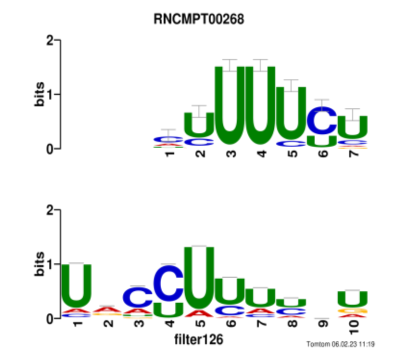 | 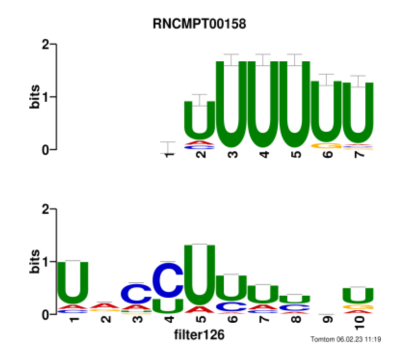 | 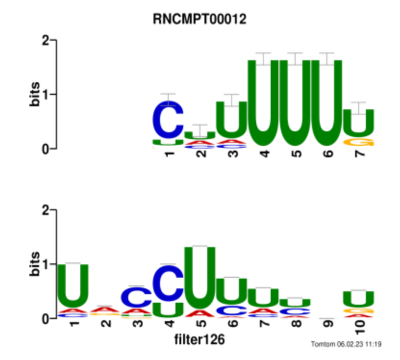 | 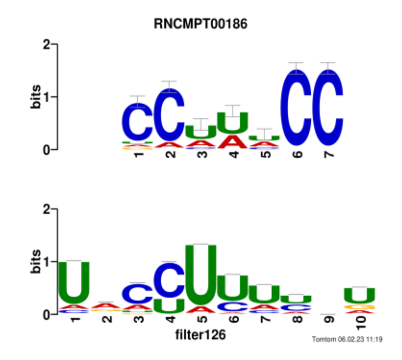 | 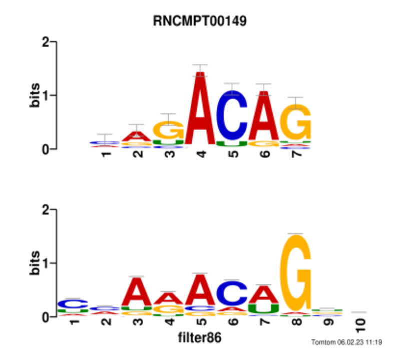 |
| AGO2 | PABPC4 | PCBP1 | SART3 | PABPC1 | PTBP1 |
| | 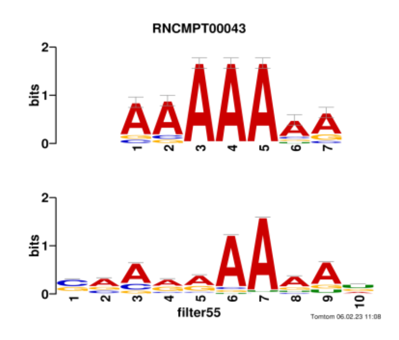 | 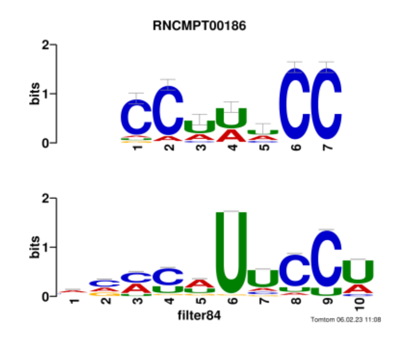 | 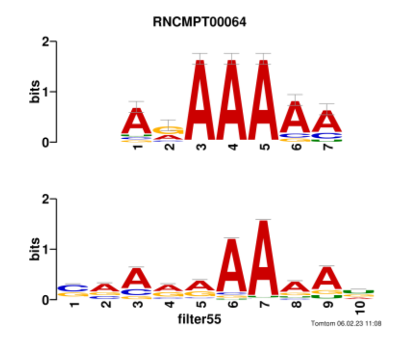 | 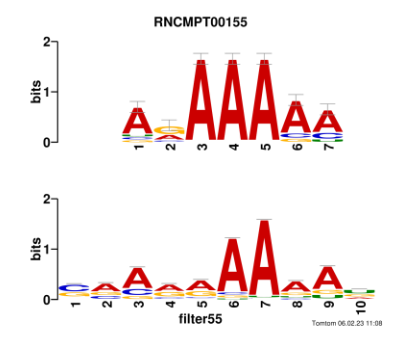 | 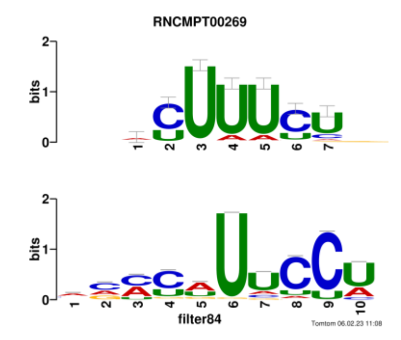 |
| AGO3 | HUR | SRSF9 | RALY | HUR | TIA1 |
| | 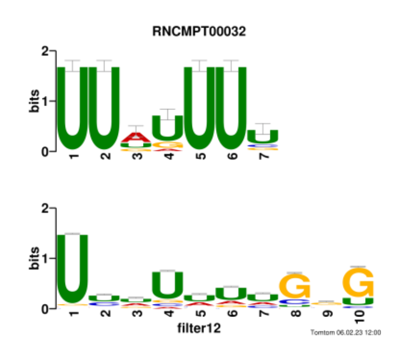 | 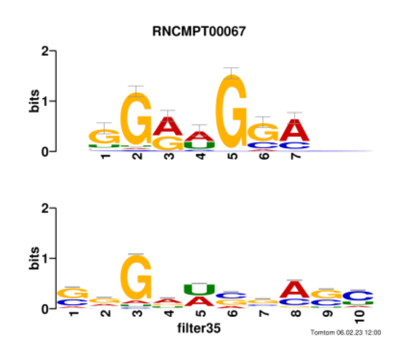 | 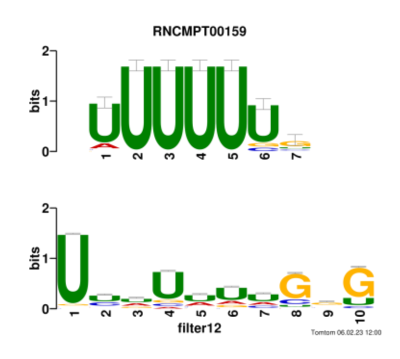 | 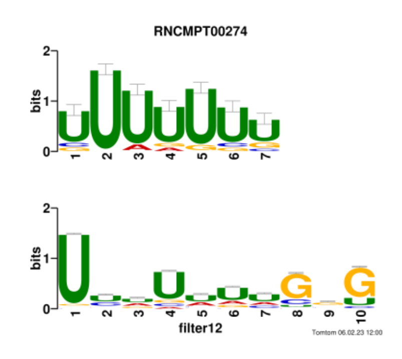 | 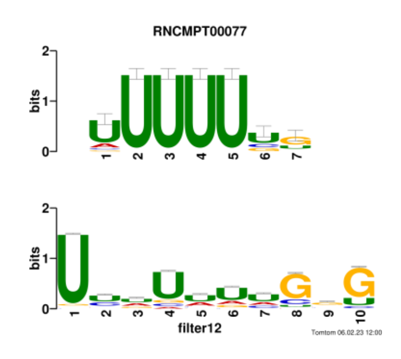 |

**ALKBH5**

PCBP2
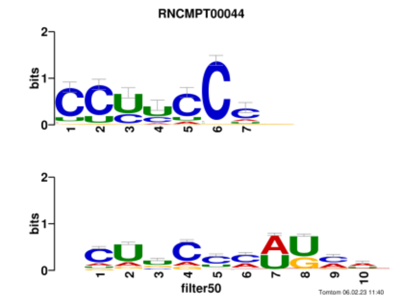


MBNL1
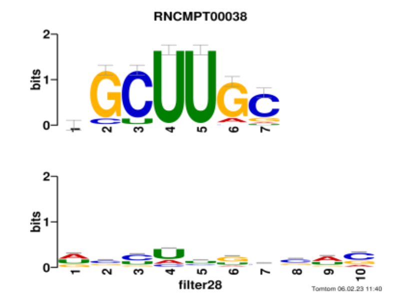


CNOT4
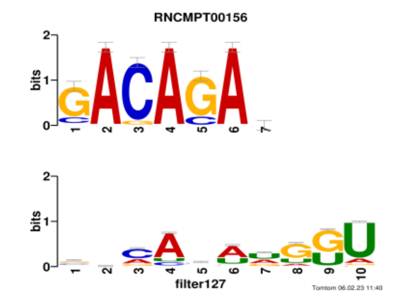


MBNL1
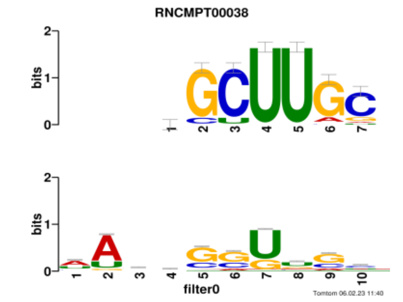


RBFOX1
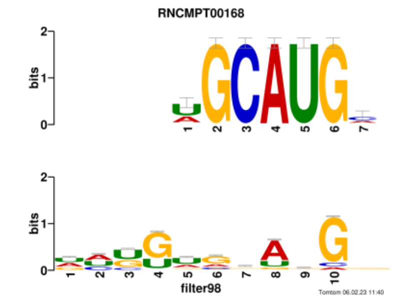


**AUF1**

HUR
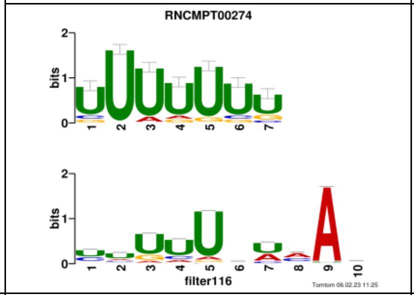


RALY
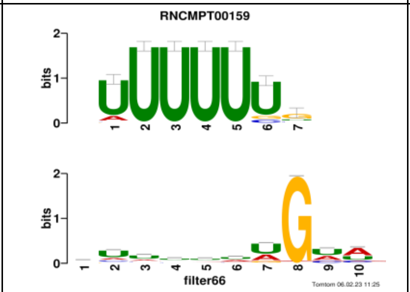


TIA1
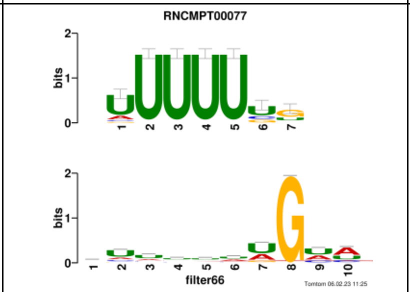


PTBP1
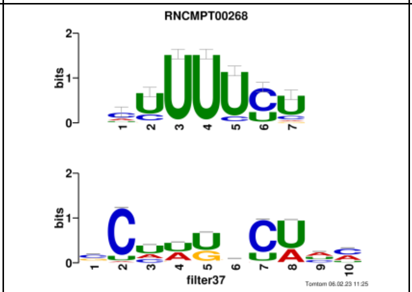


HNRNPC
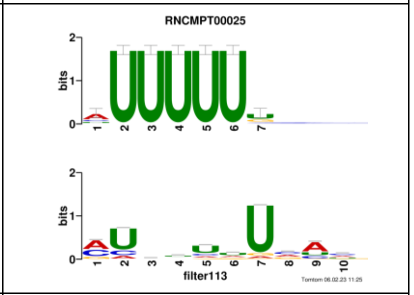


**C17ORF85**

SNRNP70
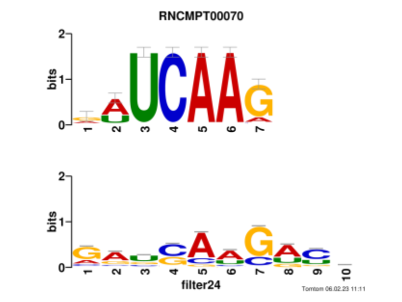


PABPN1
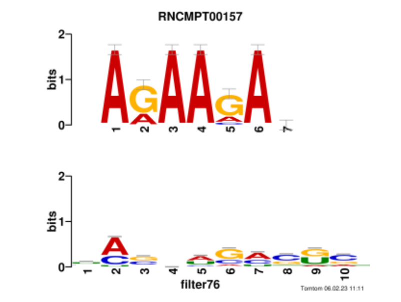


SAMD4A
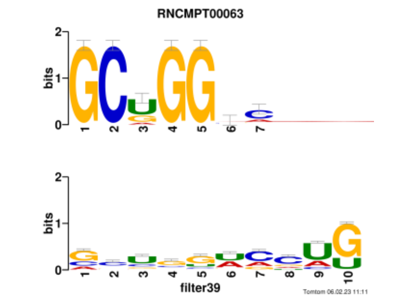


RBM45
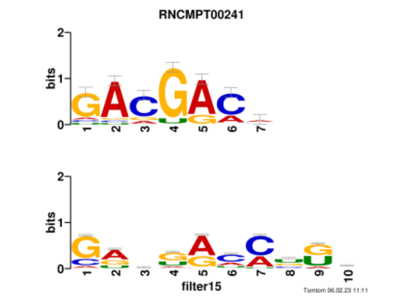


PABPC3
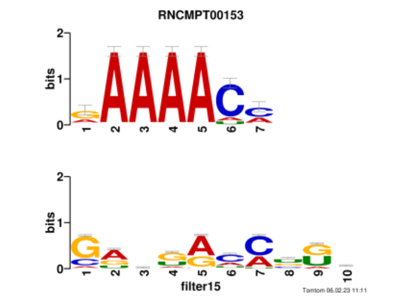


**C22ORF28**

PCBP2
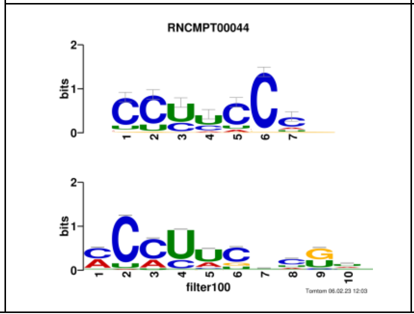


ENOX1
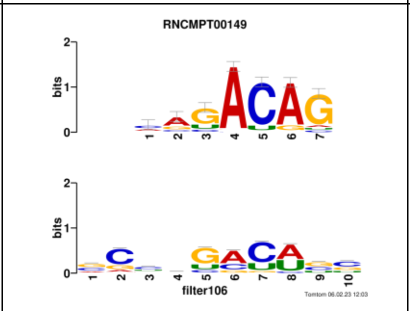


QKI
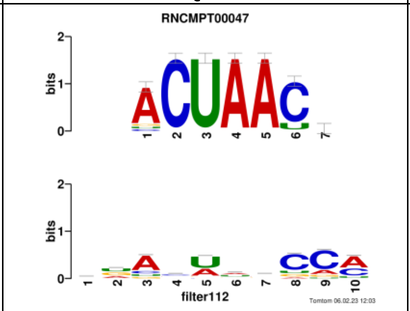


PCBP1
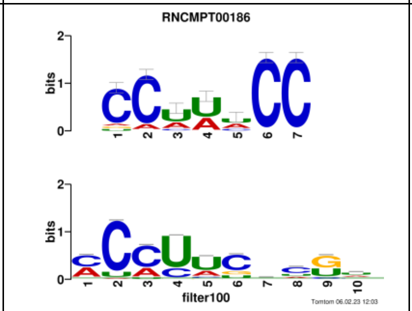


YBX1
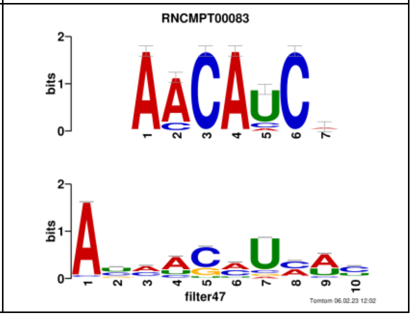

| | | | | | |
|---|---|---|---|---|---|
| CAPRIN1 | IGF2BP3 | RBM24 | IGF2BP2 | LIN28A | HNRNPL |
| | 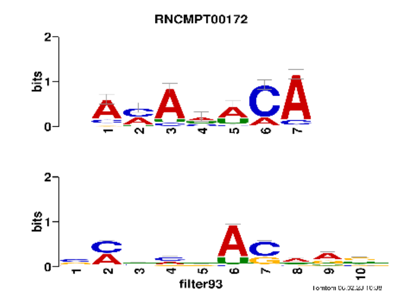 | 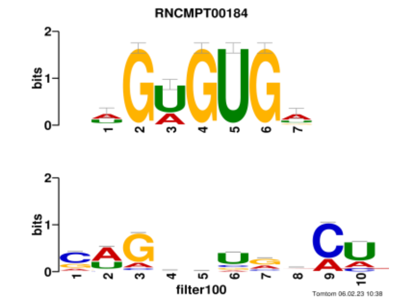 | 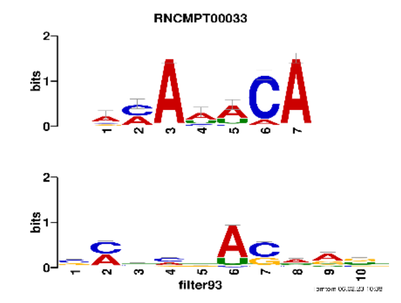 | 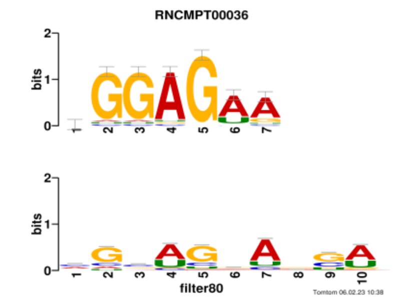 | 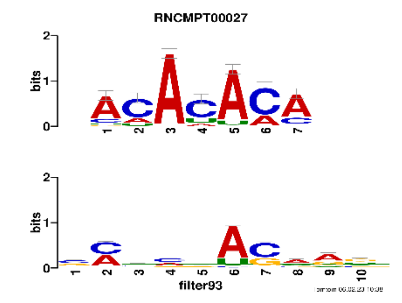 |
| DGCR8 | HNRNPK | SNRPA | SRSF10 | PCBP1 | SART3 |
| | 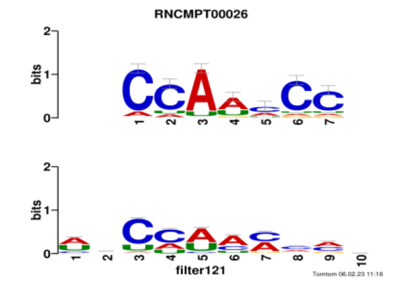 | 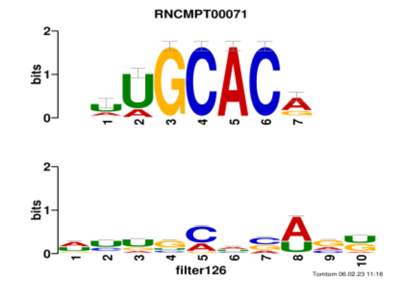 | 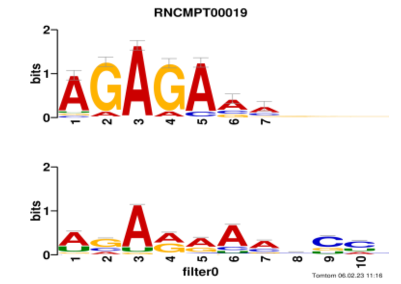 | 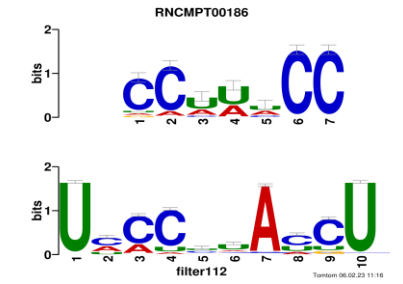 | 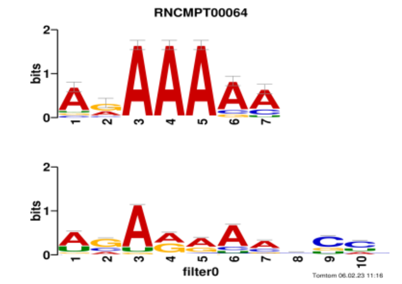 |
| EIF4A3 | U2AF2 | HuR | HuR | PTBP1 | HNRNPCL1 |
| | 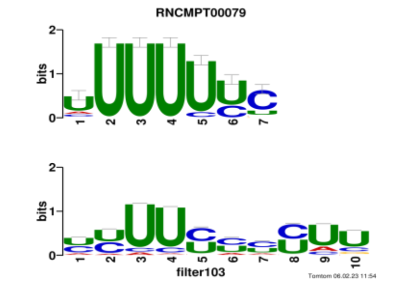 | 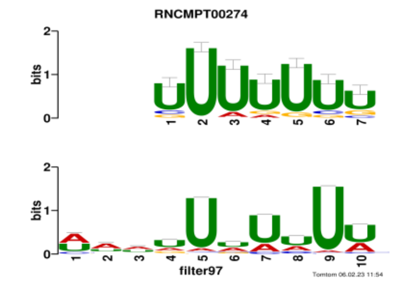 | 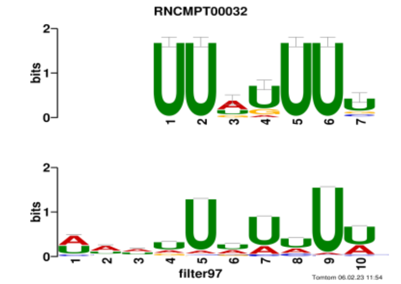 | 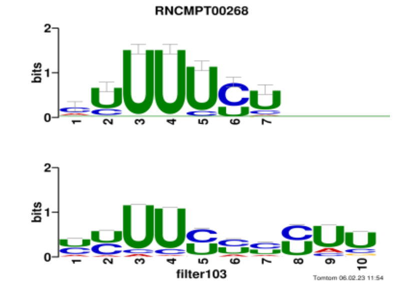 | 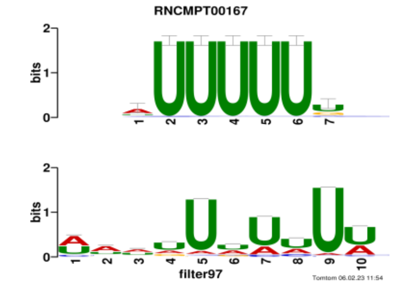 |
| EWSR1 | PTBP1 | PTBP1 | CPEB2 | CPEB4 | RALY |
| | 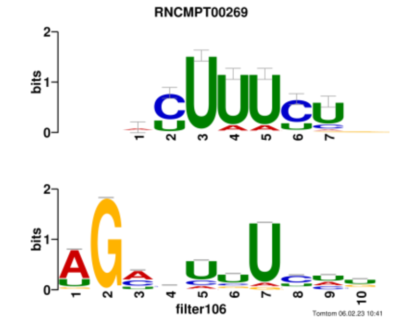 | 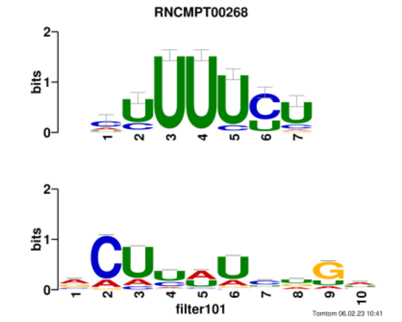 | 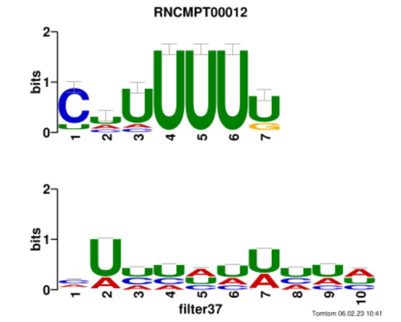 | 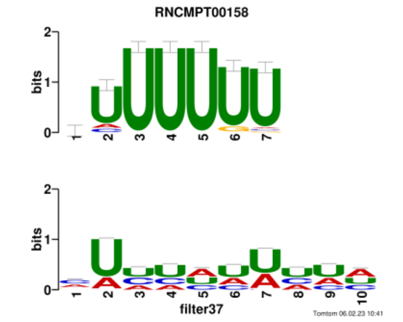 | 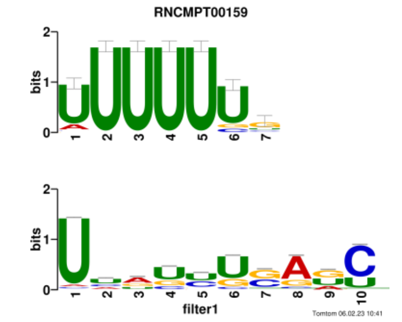 |

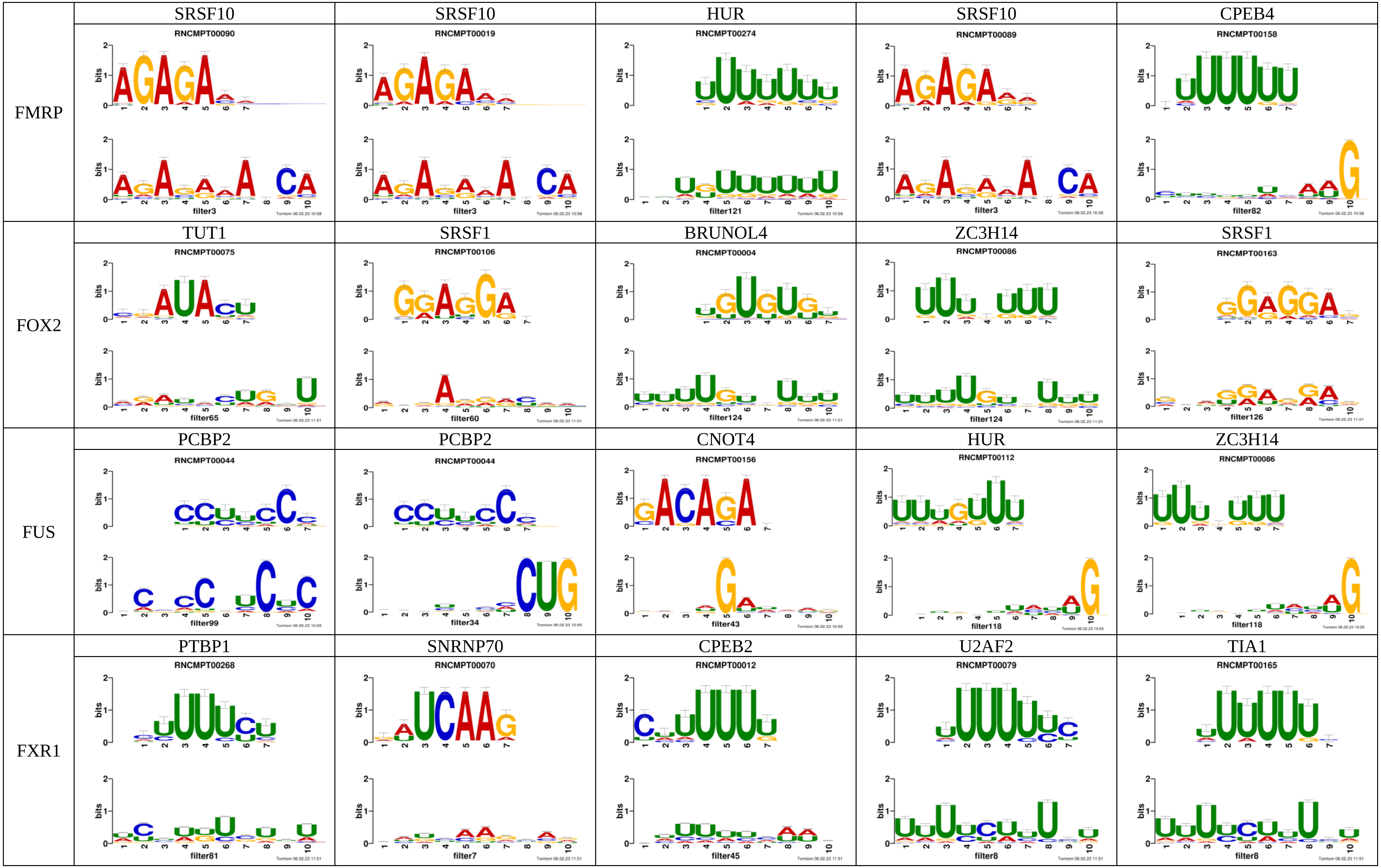
FMRP
SRSF10 RNCMPT00090 filter3
SRSF10 RNCMPT00019 filter3
HUR RNCMPT00274 filter121
SRSF10 RNCMPT00089 filter3
CPEB4 RNCMPT00158 filter82
FOX2
TUT1 RNCMPT00075 filter65
SRSF1 RNCMPT00106 filter60
BRUNOL4 RNCMPT00004 filter124
ZC3H14 RNCMPT00086 filter124
SRSF1 RNCMPT00163 filter126
FUS
PCBP2 RNCMPT00044 filter99
PCBP2 RNCMPT00044 filter34
CNOT4 RNCMPT00156 filter43
HUR RNCMPT00112 filter118
ZC3H14 RNCMPT00086 filter118
FXR1
PTBP1 RNCMPT00268 filter81
SNRNP70 RNCMPT00070 filter7
CPEB2 RNCMPT00012 filter45
U2AF2 RNCMPT00079 filter8
TIA1 RNCMPT00165 filter8

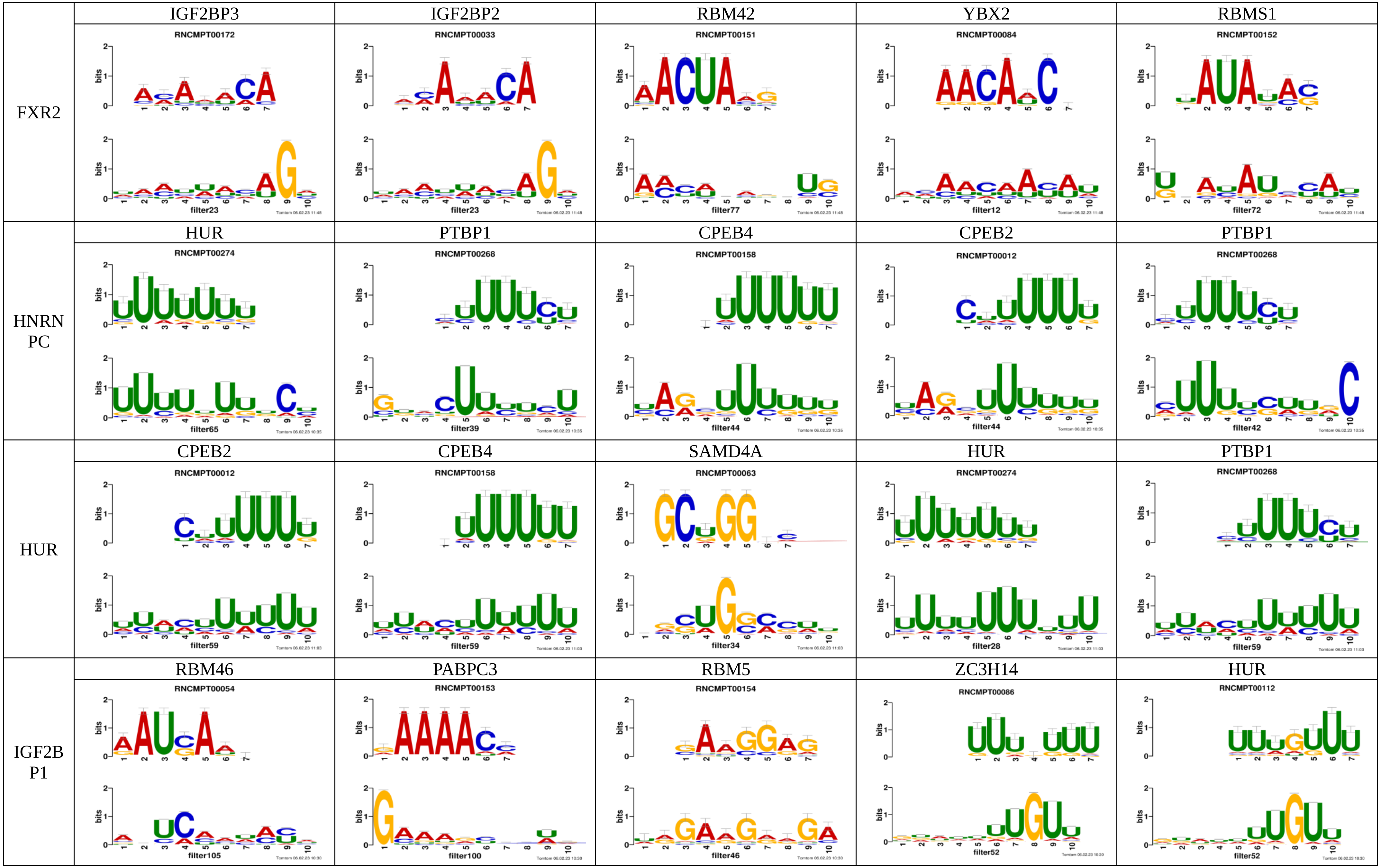
FXR2
IGF2BP3
RNCMPT00172
filter23
IGF2BP2
RNCMPT00033
filter23
RBM42
RNCMPT00151
filter77
YBX2
RNCMPT00084
filter12
RBMS1
RNCMPT00152
filter72
HNRNPC
HUR
RNCMPT00274
filter65
PTBP1
RNCMPT00268
filter39
CPEB4
RNCMPT00158
filter44
CPEB2
RNCMPT00012
filter44
PTBP1
RNCMPT00268
filter42
HUR
CPEB2
RNCMPT00012
filter59
CPEB4
RNCMPT00158
filter59
SAMD4A
RNCMPT00063
filter34
HUR
RNCMPT00274
filter28
PTBP1
RNCMPT00268
filter59
IGF2BP1
RBM46
RNCMPT00054
filter105
PABPC3
RNCMPT00153
filter100
RBM5
RNCMPT00154
filter46
ZC3H14
RNCMPT00086
filter52
HUR
RNCMPT00112
filter52

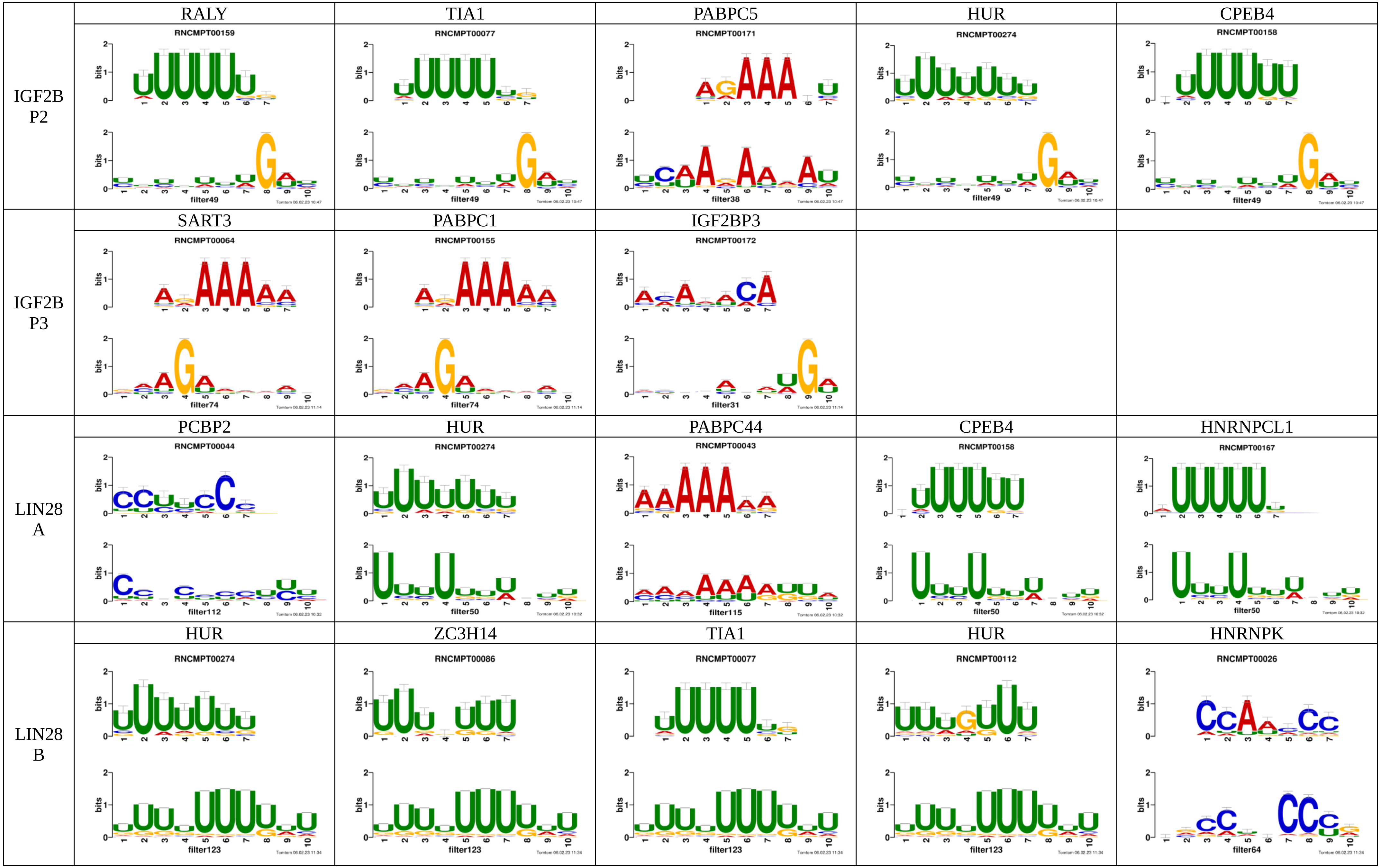

IGF2BP2
RALY
RNCMPT00159
filter49
TIA1
RNCMPT00077
filter49
PABPC5
RNCMPT00171
filter38
HUR
RNCMPT00274
filter49
CPEB4
RNCMPT00158
filter49
IGF2BP3
SART3
RNCMPT00064
filter74
PABPC1
RNCMPT00155
filter74
IGF2BP3
RNCMPT00172
filter31
LIN28A
PCBP2
RNCMPT00044
filter112
HUR
RNCMPT00274
filter50
PABPC44
RNCMPT00043
filter115
CPEB4
RNCMPT00158
filter50
HNRNPCL1
RNCMPT00167
filter50
LIN28B
HUR
RNCMPT00274
filter123
ZC3H14
RNCMPT00086
filter123
TIA1
RNCMPT00077
filter123
HUR
RNCMPT00112
filter123
HNRNPK
RNCMPT00026
filter64

| | | | | | |
|---|---|---|---|---|---|
| METTL3 | TIA1 | U2AF2 | HUR | RBM6 | SAMD4A |
| | 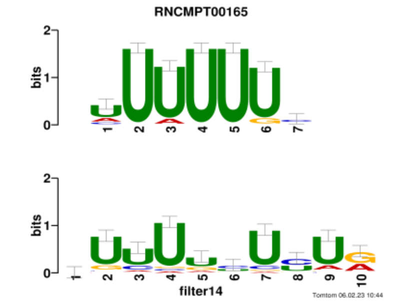  | 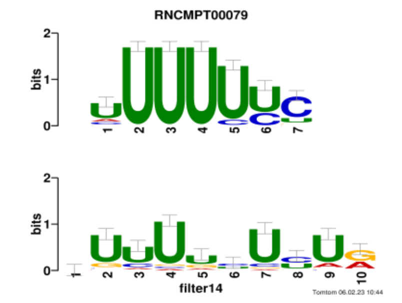  | 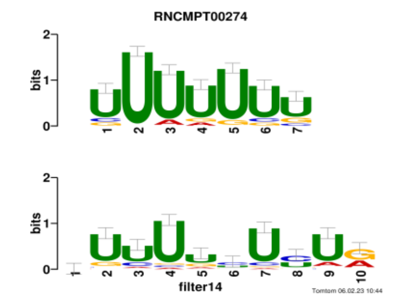  | 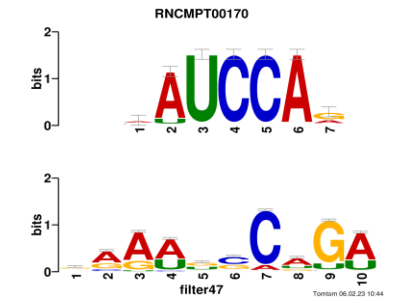  | 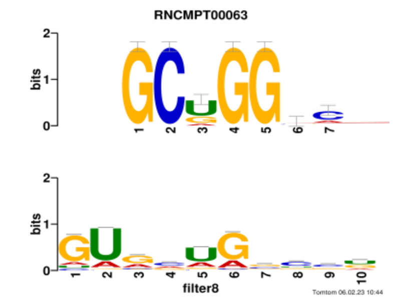  |
| MOV10 | QKI | PTBP1 | HUR | BRUNOL5 | BRUNOL4 |
| | 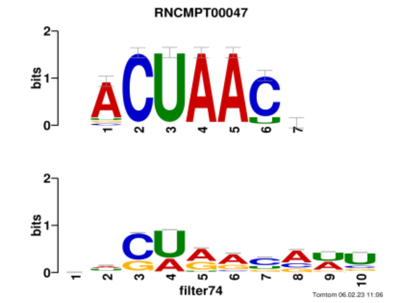  | 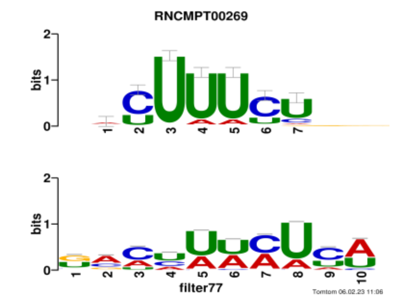  | 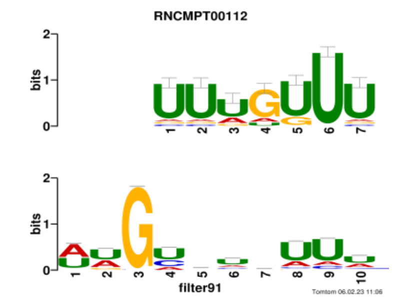  | 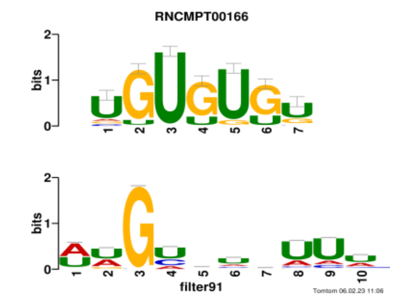  | 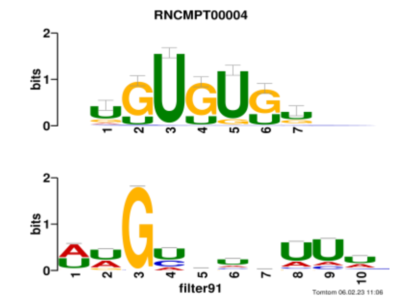  |
| PTB | PCBP2 | HNRNPC | HNRNPCL1 | PCBP1 | KHDRBS2 |
| | 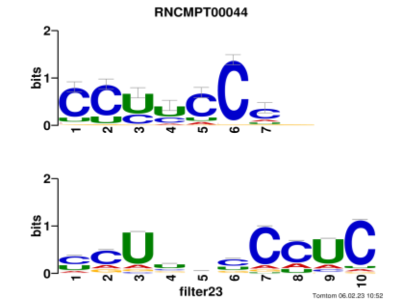  | 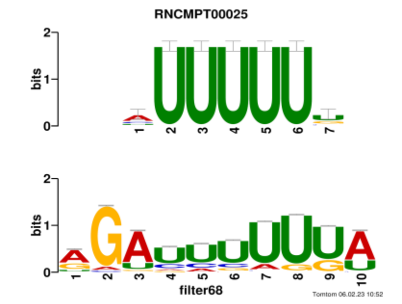  | 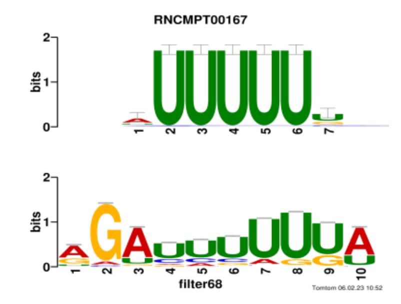  | 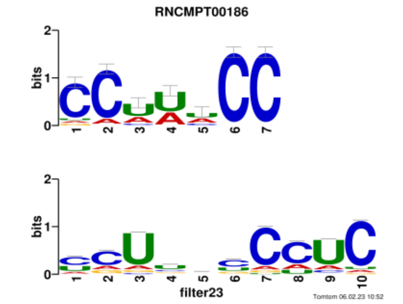  | 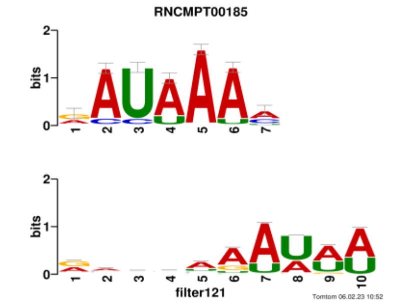  |
| PUM2 | YBX1 | YBX1 | PABPC3 | PTBP1 | CPEB4 |
| | 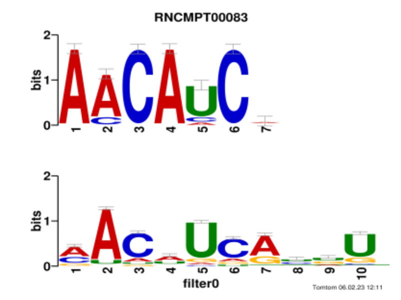  | 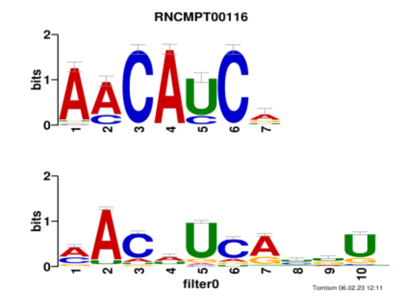  | 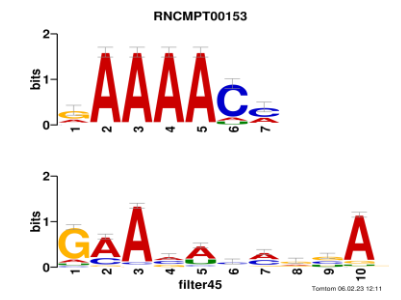  | 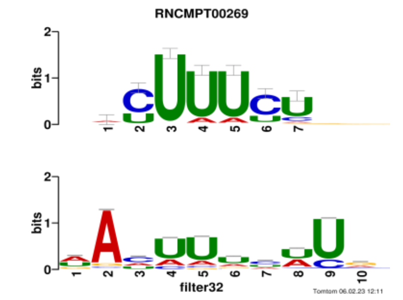  | 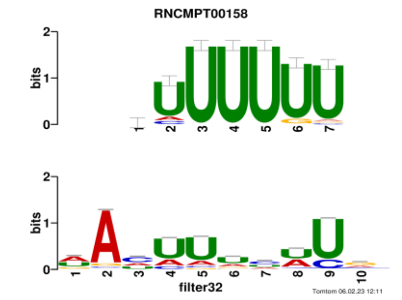  |

| | | | | | |
|---|---|---|---|---|---|
| QKI | SNRPA | HNRNPC | HNRNPCL1 | QKI | SRSF1 |
| | 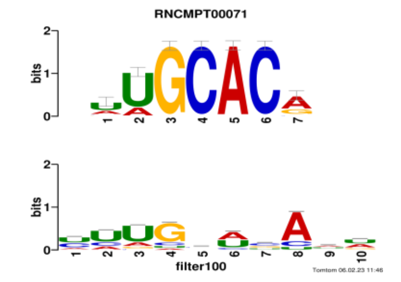  | 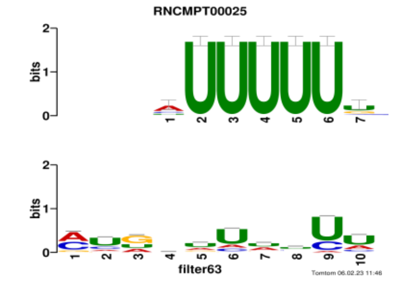  | 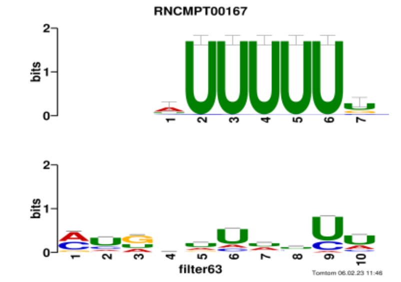  | 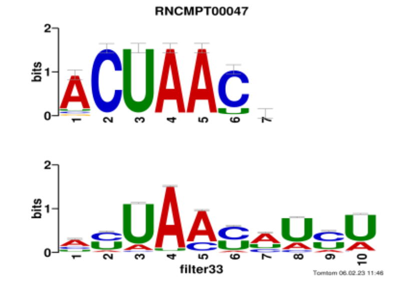  | 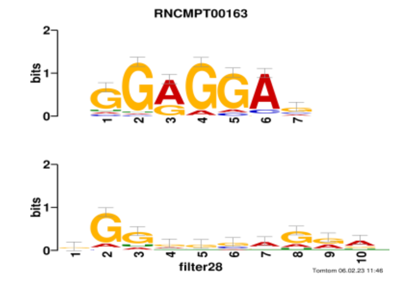  |
| SFRS1 | LIN28A | SAMD4A | PCBP2 | YBX1 | YBX1 |
| | 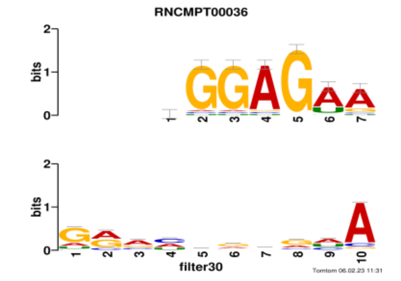  | 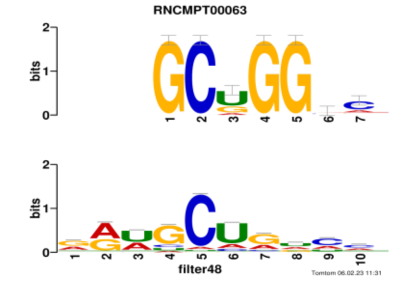  | 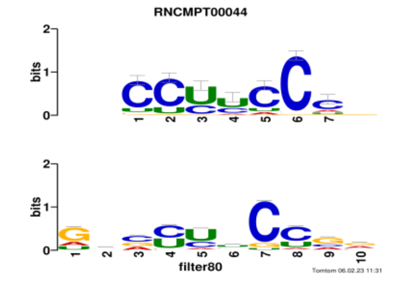  | 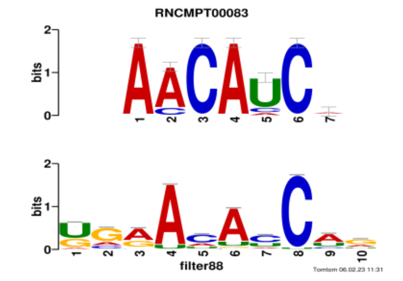  | 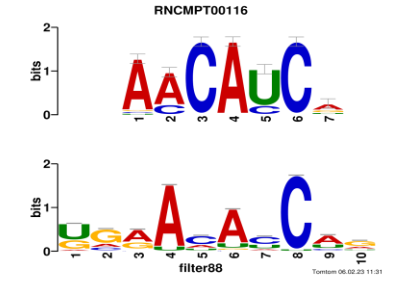  |
| TAF15 | SRSF1 | SRSF1 | U2AF2 | CPEB2 | TIA1 |
| | 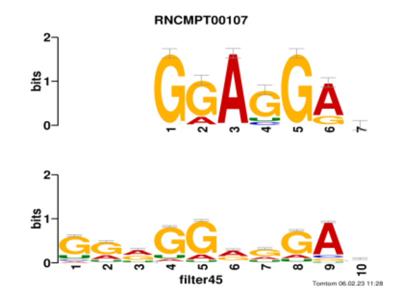  | 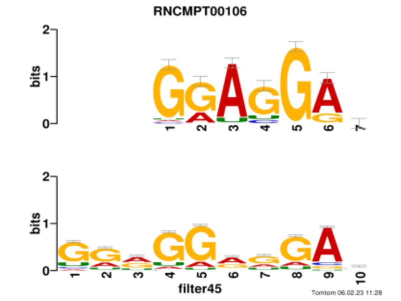  | 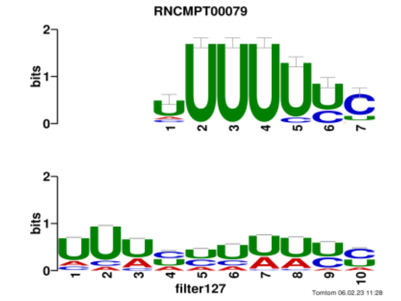  | 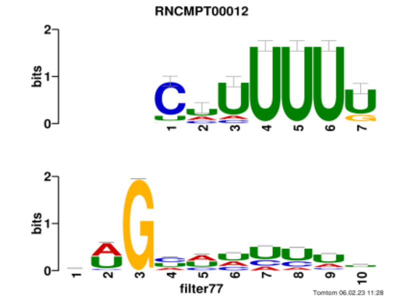  | 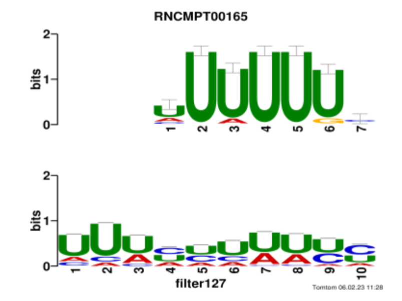  |
| TDP43 | CPEB2 | BRUNOL5 | PCBP2 | HNRPLL | HNRNPL |
| | 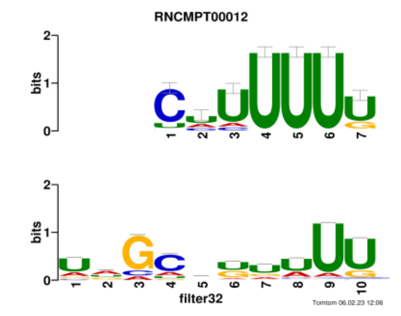  | 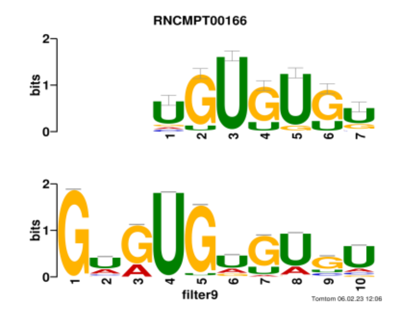  | 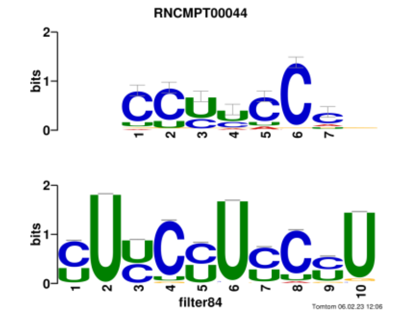  | 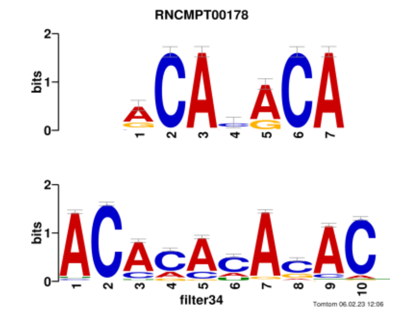  | 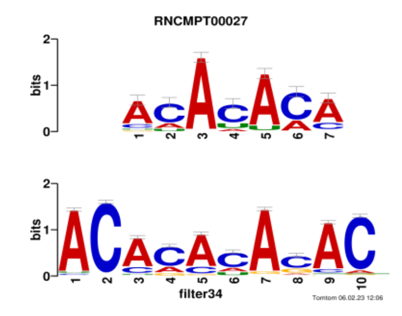  |

| | | | | | |
|---|---|---|---|---|---|
| TIA1 | PCBP2 | PTBP1 | HUR | PCBP2 | TIA1 |
| | 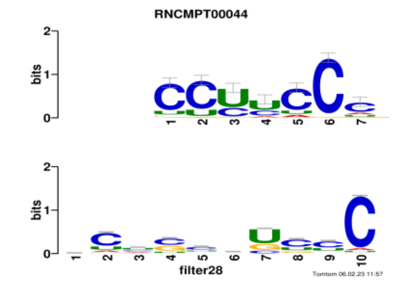 | 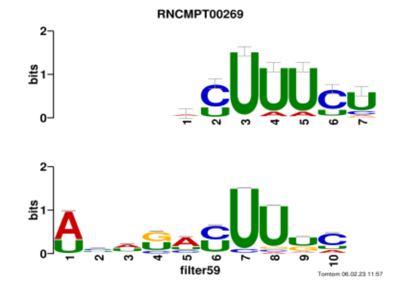 | 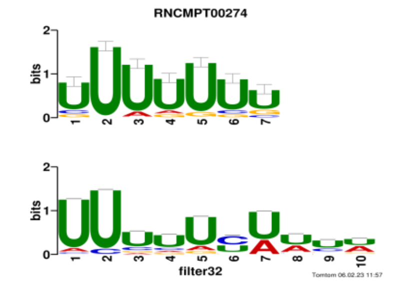 | 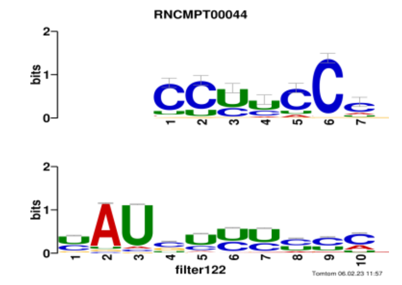 | 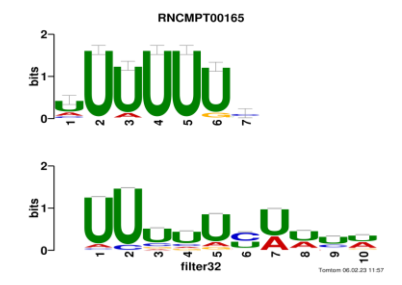 |
| TIAL1 | HUR | HUR | PCBP2 | HUR | TIA1 |
| | 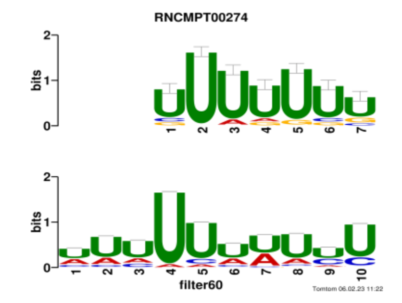 | 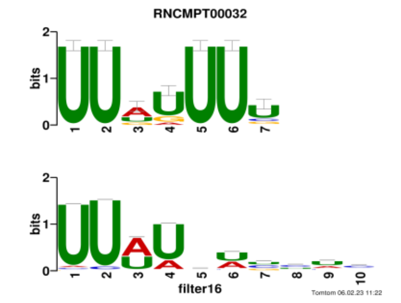 | 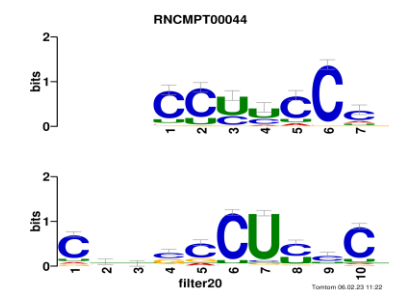 | 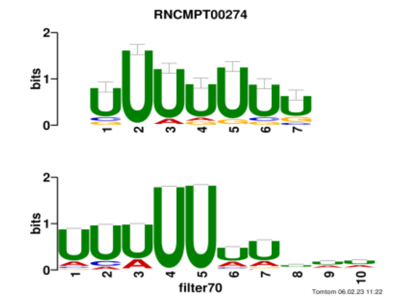 | 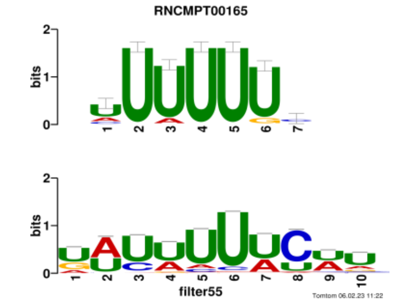 |
| TNRC 6 | HNRNPL | U2AF2 | TIA1 | HNRNPCL1 | HNRNPC |
| | 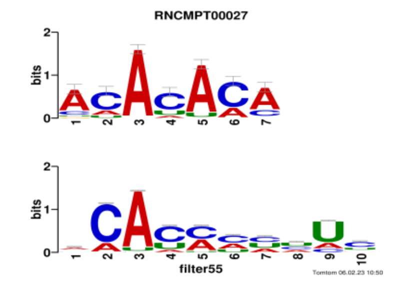 | 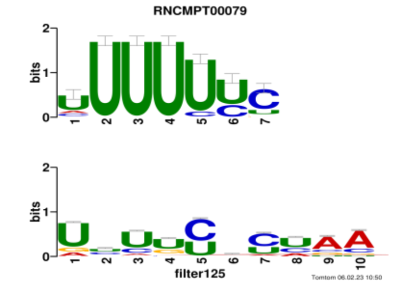 | 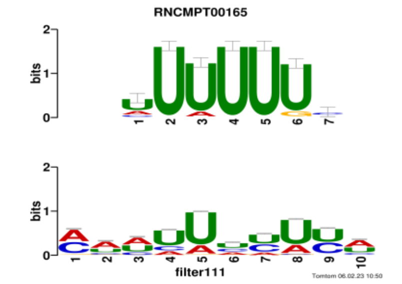 | 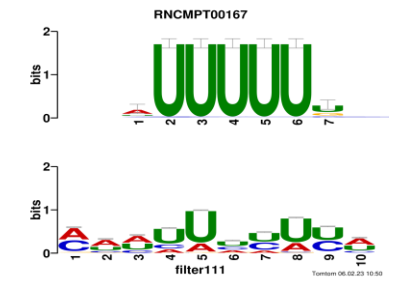 | 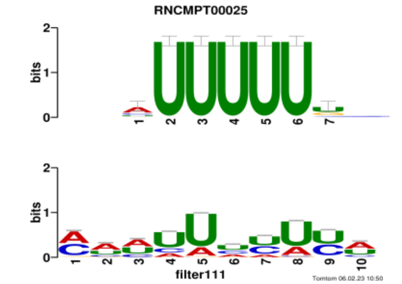 |
| U2AF 65 | U2AF2 | HNRNPC | HNRNPCL1 | U2AF2 | ZC3H14 |
| | 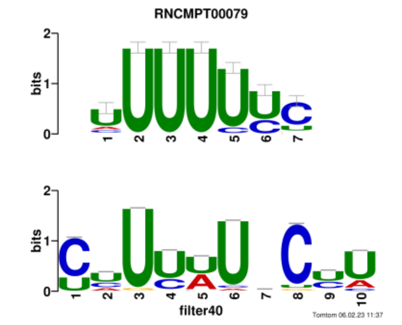 | 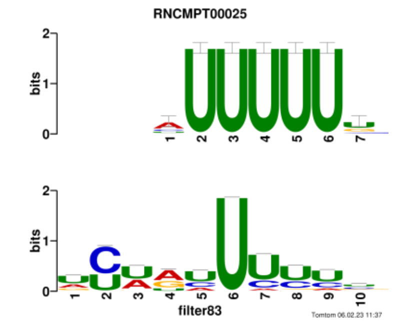 | 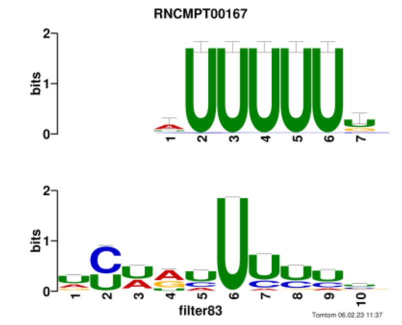 | 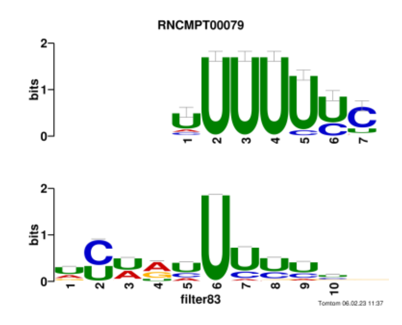 | 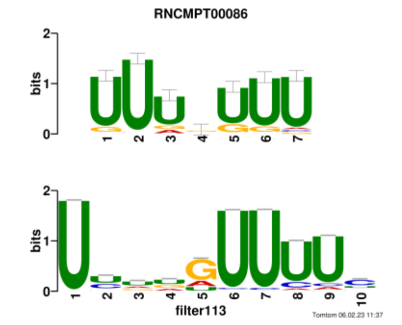 |

| | | | | | |
|---|---|---|---|---|---|
| WTAP | PCBP2 | PCBP2 | LIN28A | PPRC1 | SRSF1 |
| | 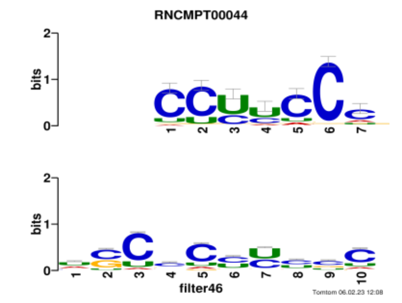  | 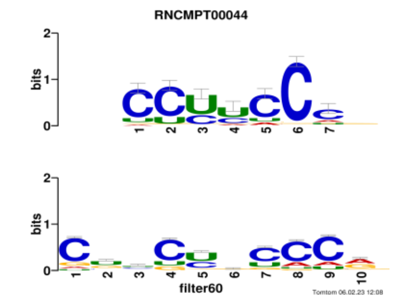  | 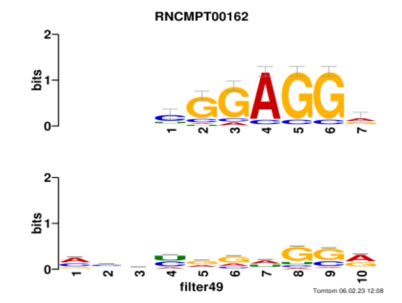  | 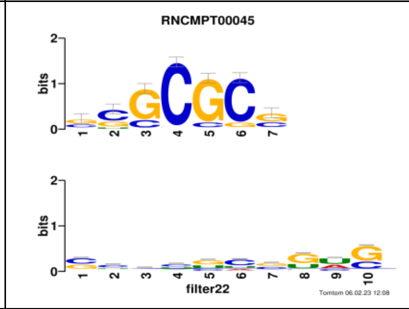  | 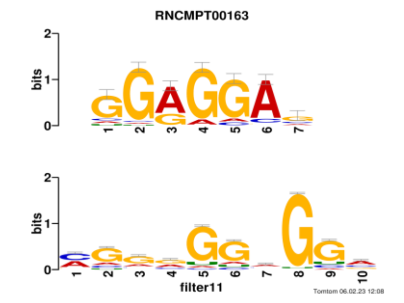  |
| ZC3H 7B | CPEB4 | CPEB2 | PTBP1 | HUR | CPEB4 |
| | 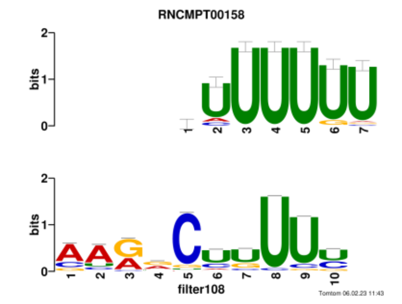  | 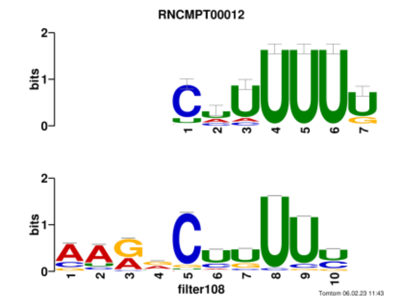  | 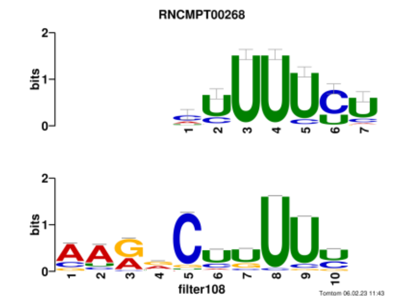  | 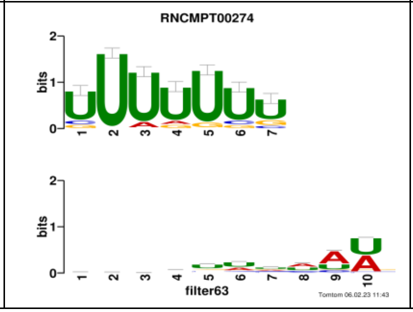  | 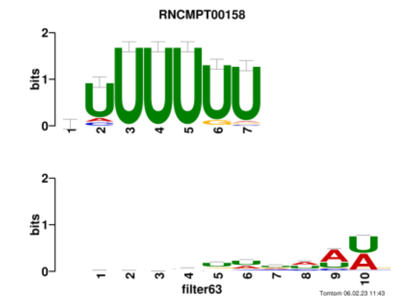  |

**Table S18.** Top5 Motif found by CRIP in each dataset. Here, the sum of p-value, e-value and q-value was used to rank all the matched motifs (ascending order).

| Protein Name | Motif | | | | |
|---|---|---|---|---|---|
| AGO1 | PCBP2 | PABPC3 | U2AF2 | PABPN1 | TIA1 |
| | 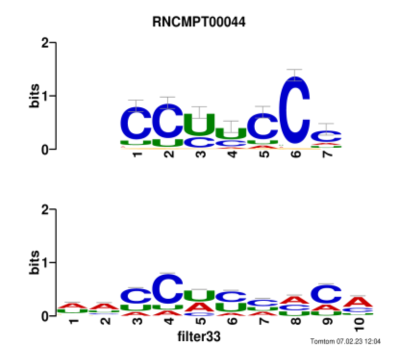  | 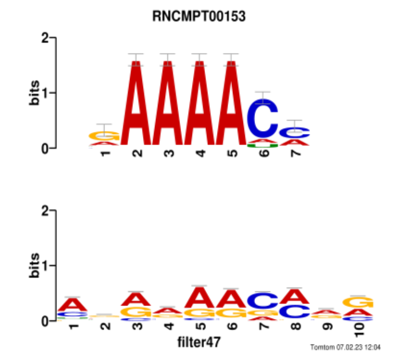  | 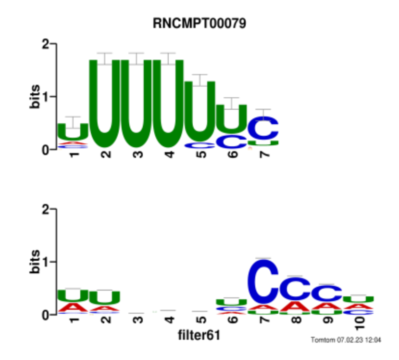  | 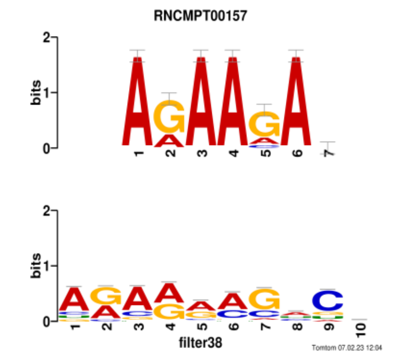  | 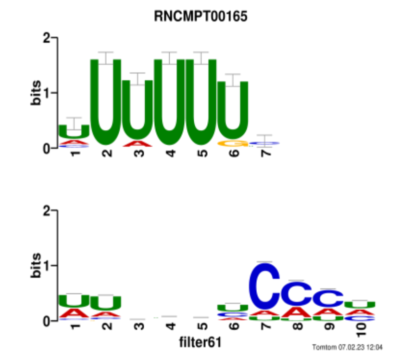  |
| AGO2 | PCBP2 | HUR | CPEB4 | PCBP2 | PCBP2 |
| | 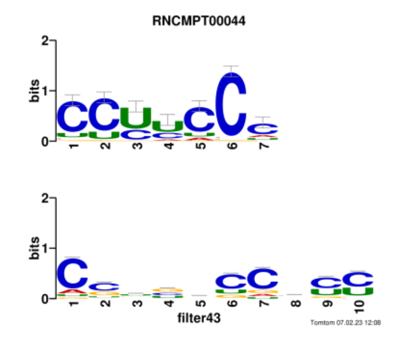  | 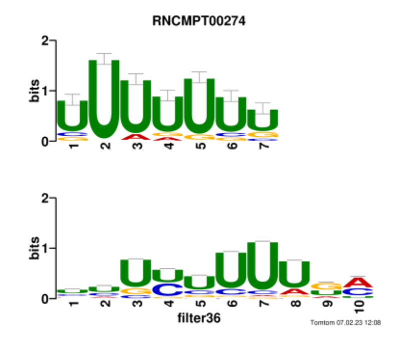  | 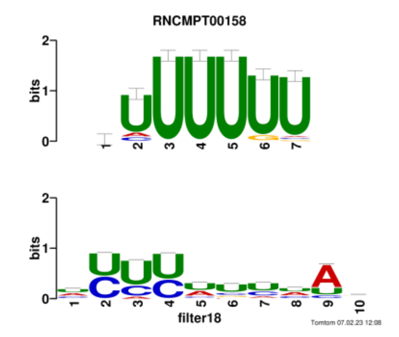  | 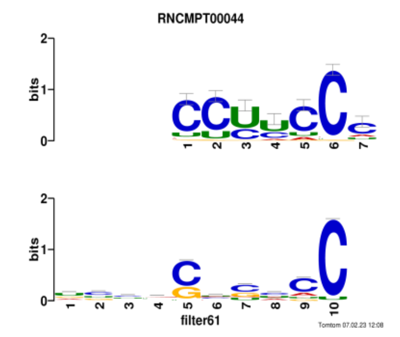  | 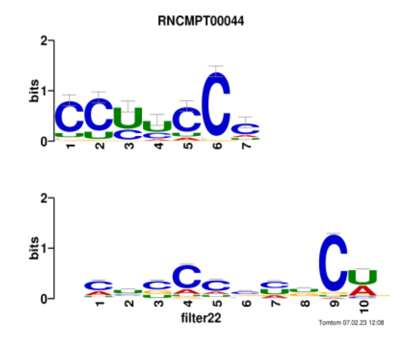  |
| AGO3 | ZC3H10 | CPEB4 | CPEB4 | PABPC4 | CPEB2 |
| | 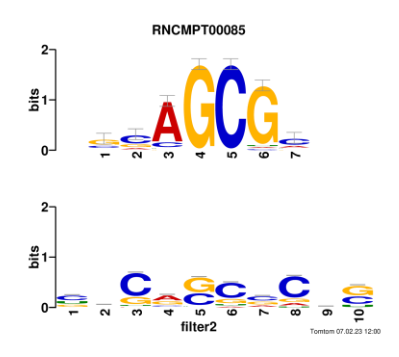  | 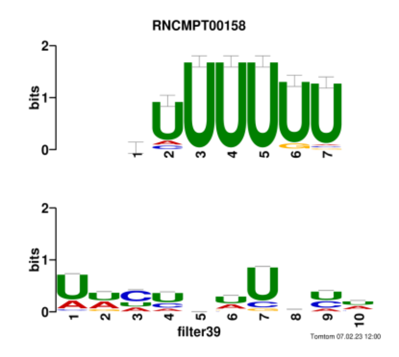  | 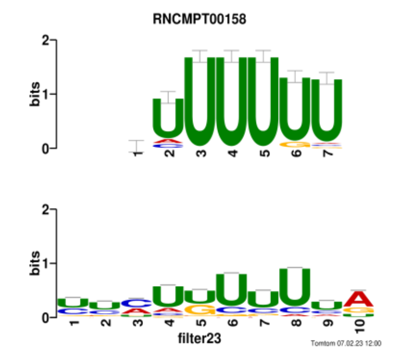  | 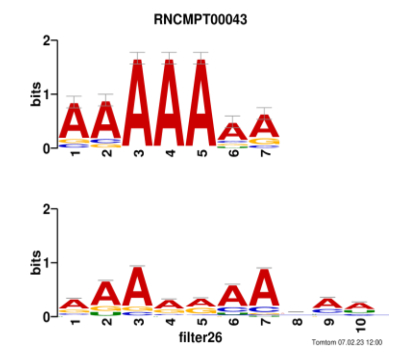  | 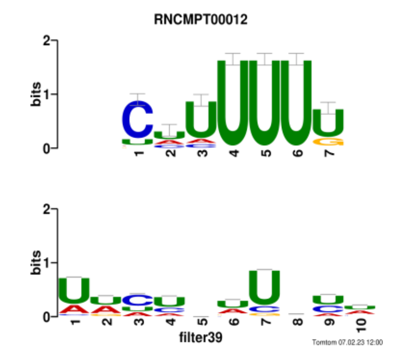  |

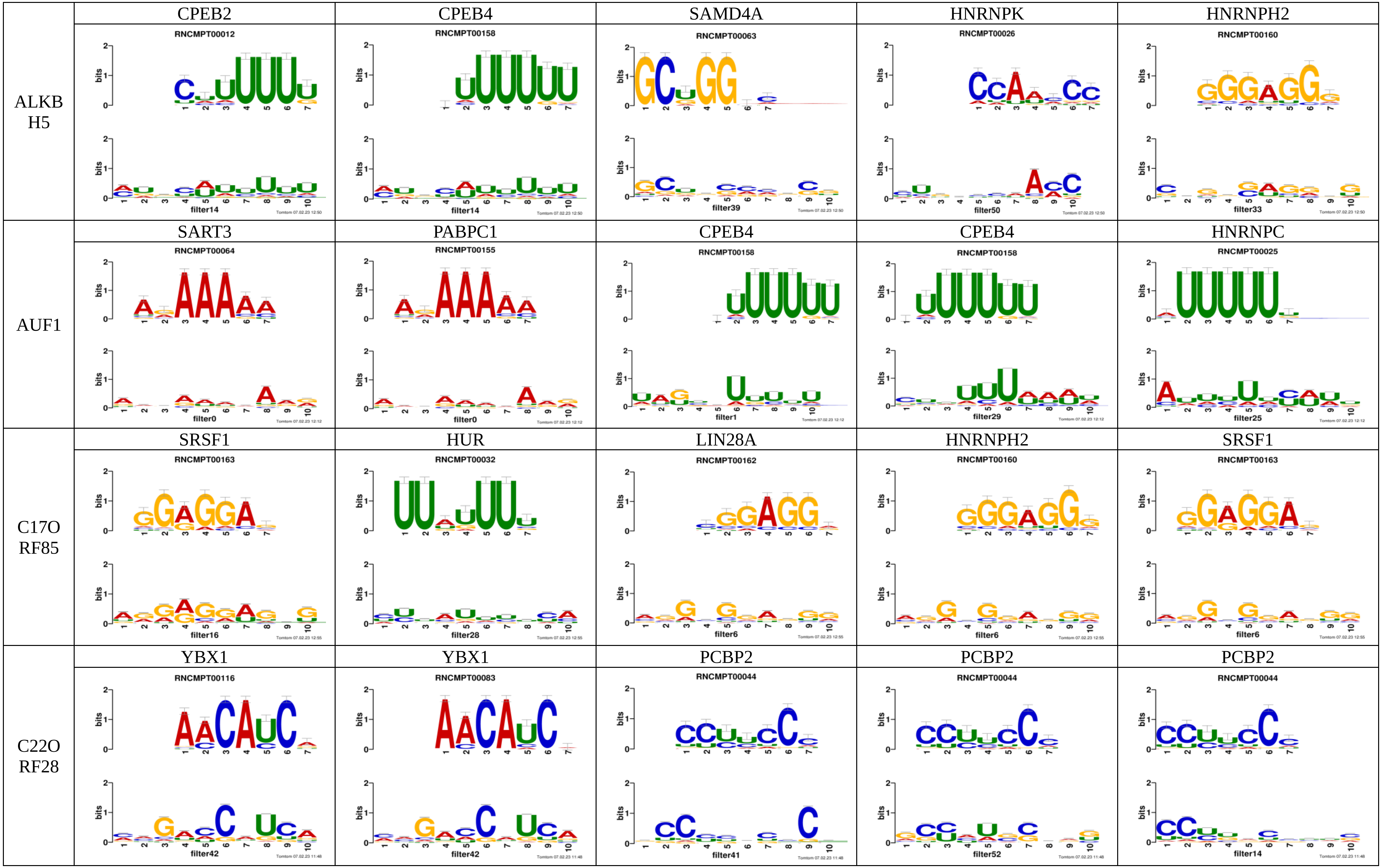
ALKBH5
CPEB2
RNCMPT00012
filter14
CPEB4
RNCMPT00158
filter14
SAMD4A
RNCMPT00063
filter39
HNRNPK
RNCMPT00026
filter50
HNRNPH2
RNCMPT00160
filter33
AUF1
SART3
RNCMPT00064
filter0
PABPC1
RNCMPT00155
filter0
CPEB4
RNCMPT00158
filter1
CPEB4
RNCMPT00158
filter29
HNRNPC
RNCMPT00025
filter25
C17ORF85
SRSF1
RNCMPT00163
filter16
HUR
RNCMPT00032
filter28
LIN28A
RNCMPT00162
filter6
HNRNPH2
RNCMPT00160
filter6
SRSF1
RNCMPT00163
filter6
C22ORF28
YBX1
RNCMPT00116
filter42
YBX1
RNCMPT00083
filter42
PCBP2
RNCMPT00044
filter41
PCBP2
RNCMPT00044
filter52
PCBP2
RNCMPT00044
filter14
bits

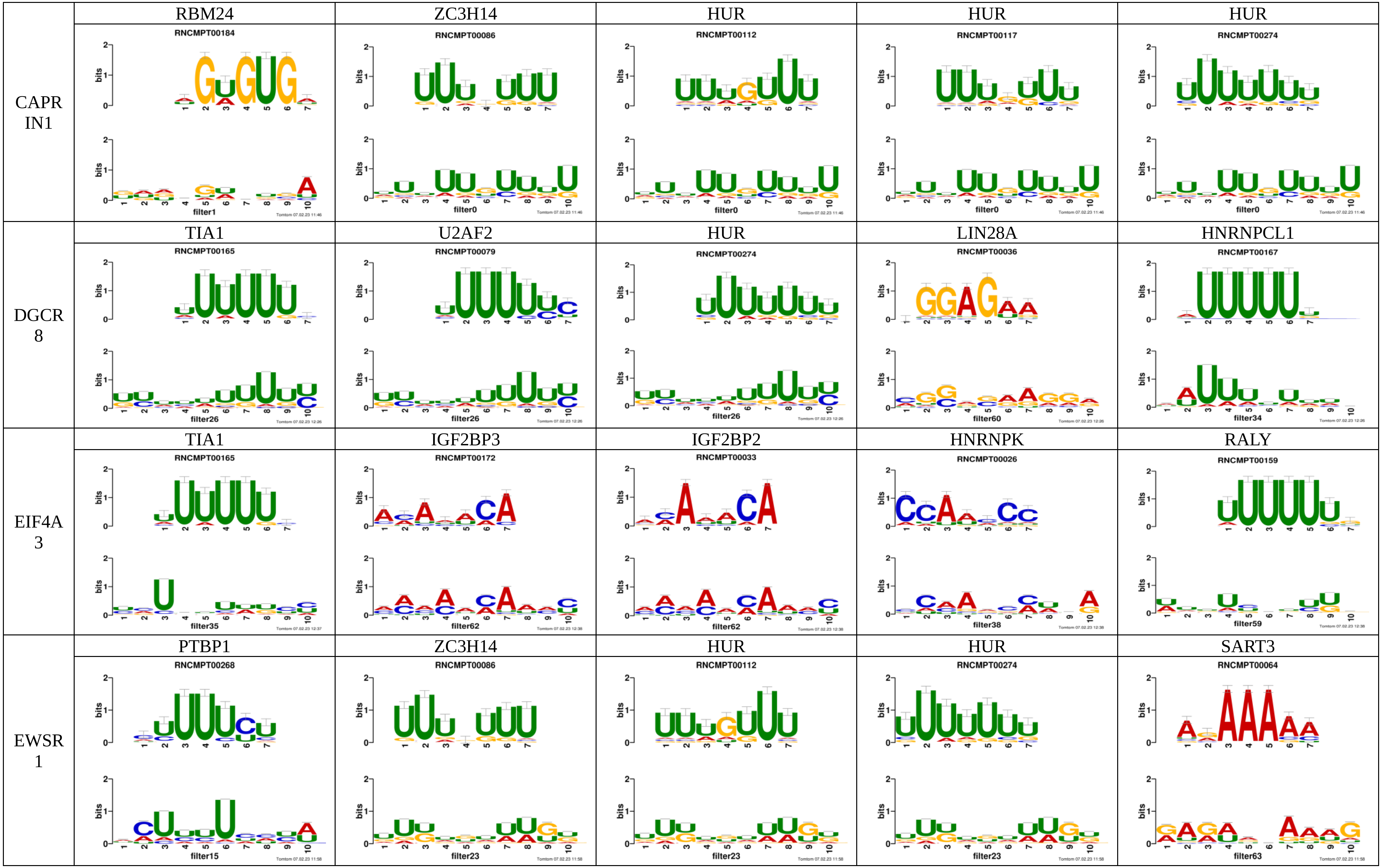

CAPRIN1
RBM24
RNCMPT00184
filter1
ZC3H14
RNCMPT00086
filter0
HUR
RNCMPT00112
filter0
HUR
RNCMPT00117
filter0
HUR
RNCMPT00274
filter0
DGCR8
TIA1
RNCMPT00165
filter26
U2AF2
RNCMPT00079
filter26
HUR
RNCMPT00274
filter26
LIN28A
RNCMPT00036
filter60
HNRNPCL1
RNCMPT00167
filter34
EIF4A3
TIA1
RNCMPT00165
filter35
IGF2BP3
RNCMPT00172
filter62
IGF2BP2
RNCMPT00033
filter62
HNRNPK
RNCMPT00026
filter38
RALY
RNCMPT00159
filter59
EWSR1
PTBP1
RNCMPT00268
filter15
ZC3H14
RNCMPT00086
filter23
HUR
RNCMPT00112
filter23
HUR
RNCMPT00274
filter23
SART3
RNCMPT00064
filter63

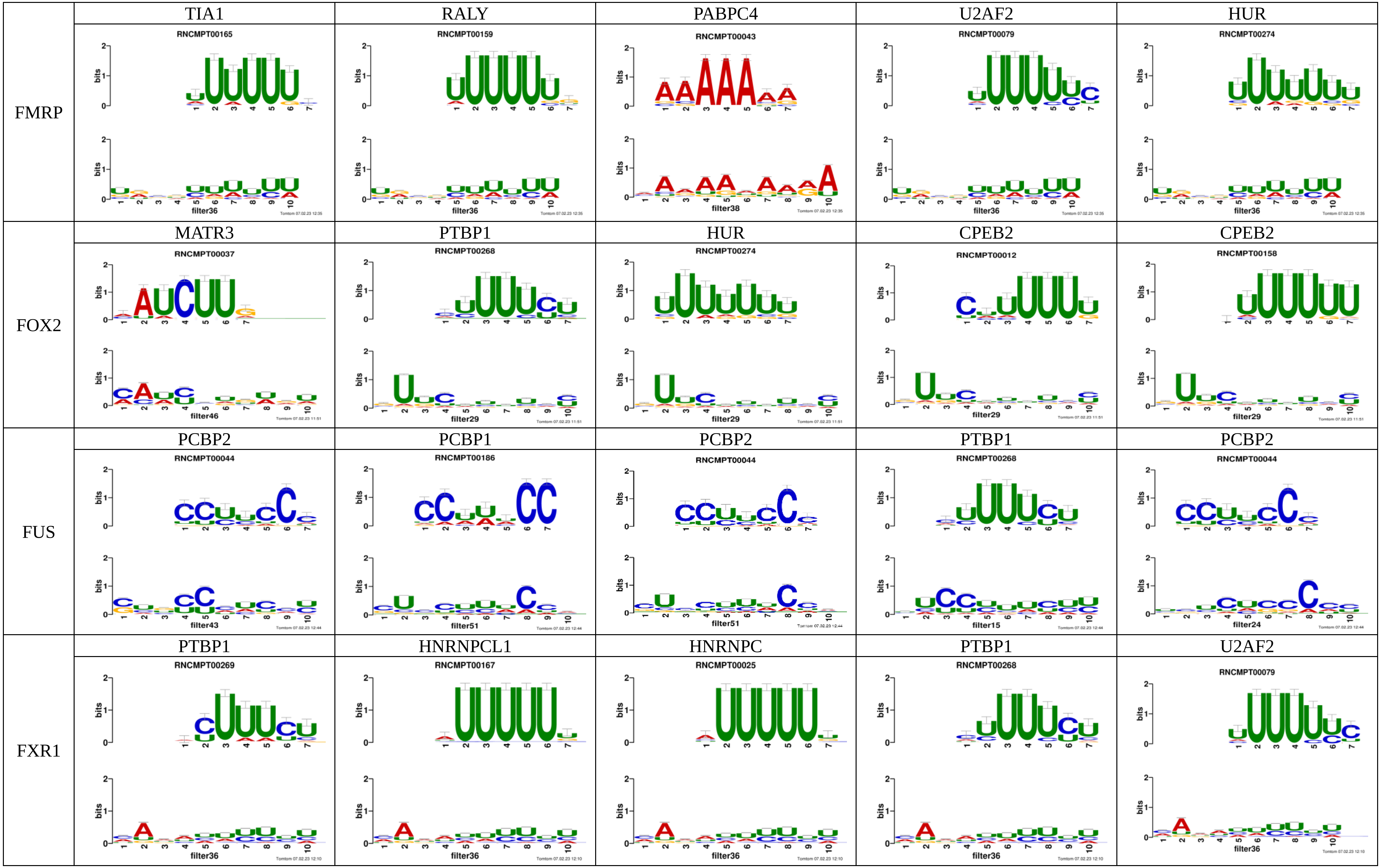
FMRP
TIA1
RNCMPT00165
filter36
RALY
RNCMPT00159
filter36
PABPC4
RNCMPT00043
filter38
U2AF2
RNCMPT00079
filter36
HUR
RNCMPT00274
filter36
FOX2
MATR3
RNCMPT00037
filter46
PTBP1
RNCMPT00268
filter29
HUR
RNCMPT00274
filter29
CPEB2
RNCMPT00012
filter29
CPEB2
RNCMPT00158
filter29
FUS
PCBP2
RNCMPT00044
filter43
PCBP1
RNCMPT00186
filter51
PCBP2
RNCMPT00044
filter51
PTBP1
RNCMPT00268
filter15
PCBP2
RNCMPT00044
filter24
FXR1
PTBP1
RNCMPT00269
filter36
HNRNPCL1
RNCMPT00167
filter36
HNRNPC
RNCMPT00025
filter36
PTBP1
RNCMPT00268
filter36
U2AF2
RNCMPT00079
filter36

| | | | | | |
|---|---|---|---|---|---|
| FXR2 | TIA1 | RALY | HUR | HNRNPK | YBX1 |
| | 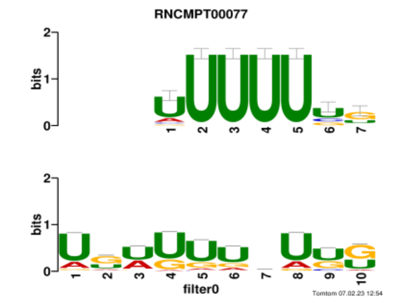 | 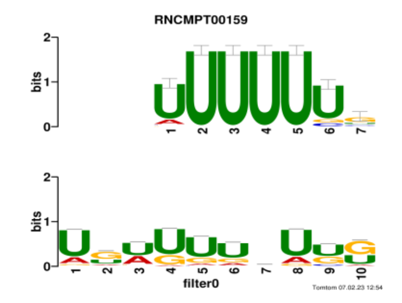 | 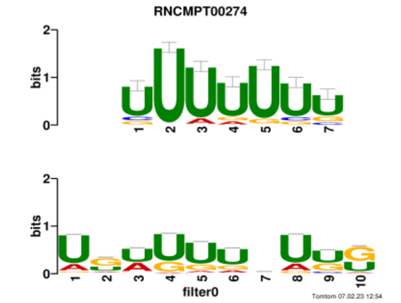 | 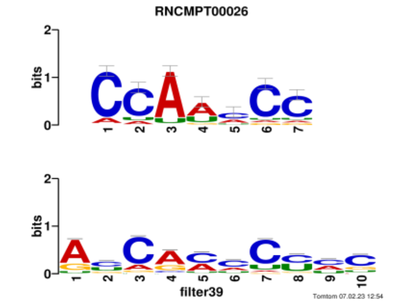 | 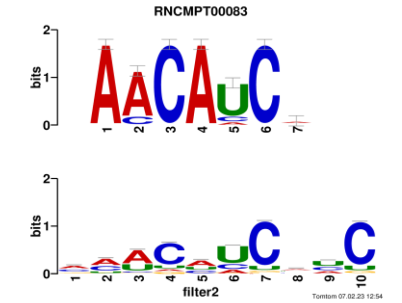 |
| HNRNPC | CPEB4 | HUR | CPEB2 | HUR | HNRNPCL1 |
| | 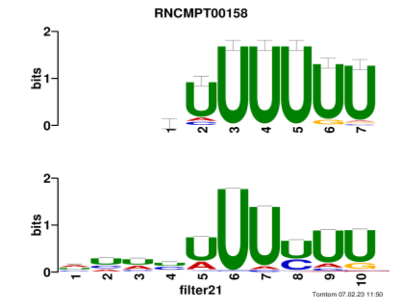 | 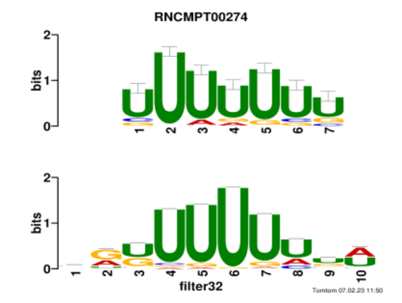 | 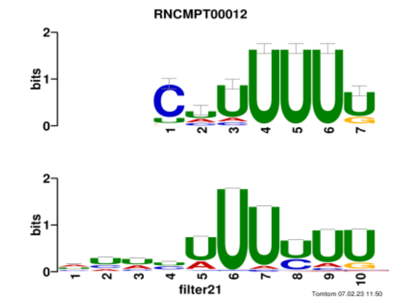 | 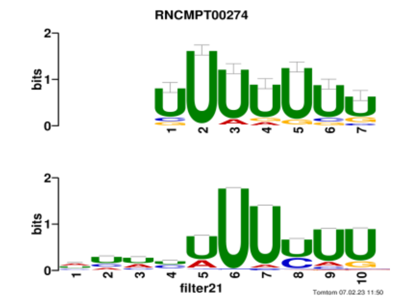 | 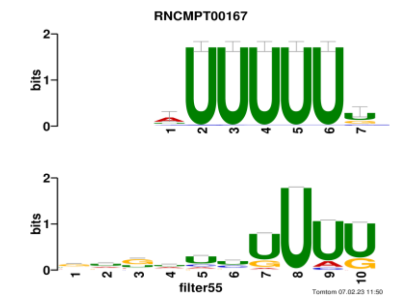 |
| HUR | SRSF7 | RALY | U2AF2 | TIA1 | HUR |
| | 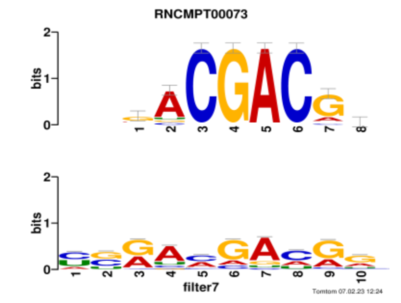 | 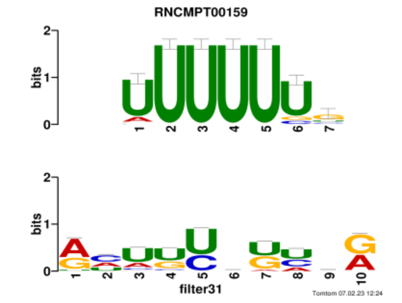 | 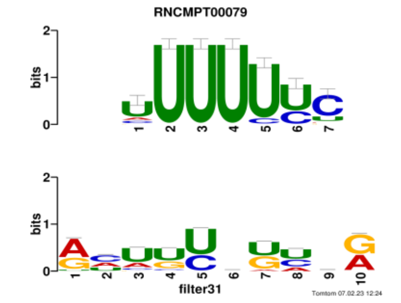 | 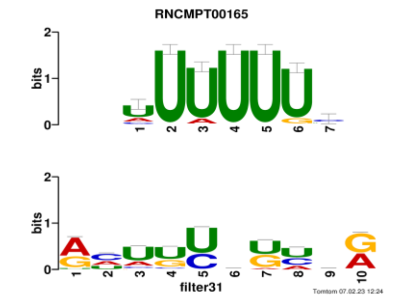 | 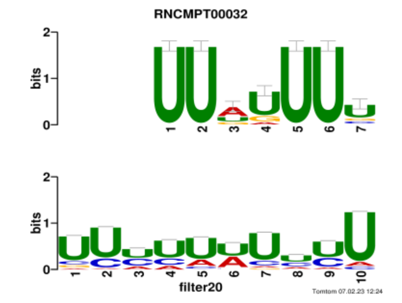 |
| IGF2BP1 | YBX1 | YBX1 | U2AF2 | HUR | ZC3H14 |
| | 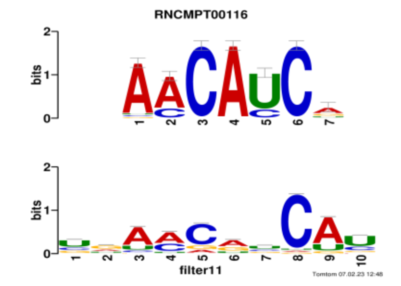 | 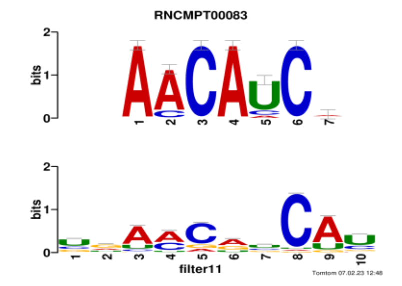 | 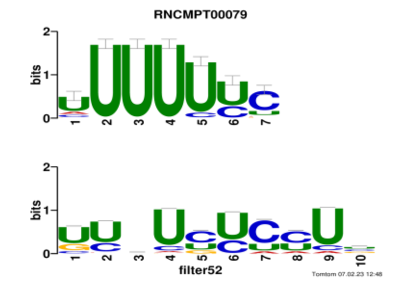 | 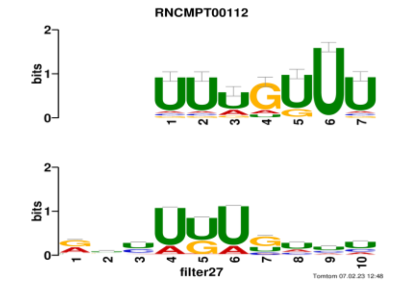 | 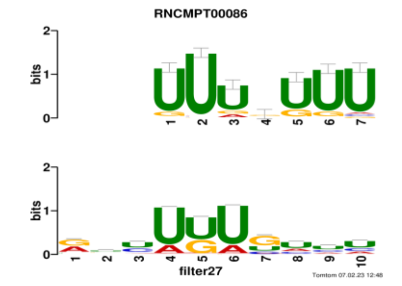 |

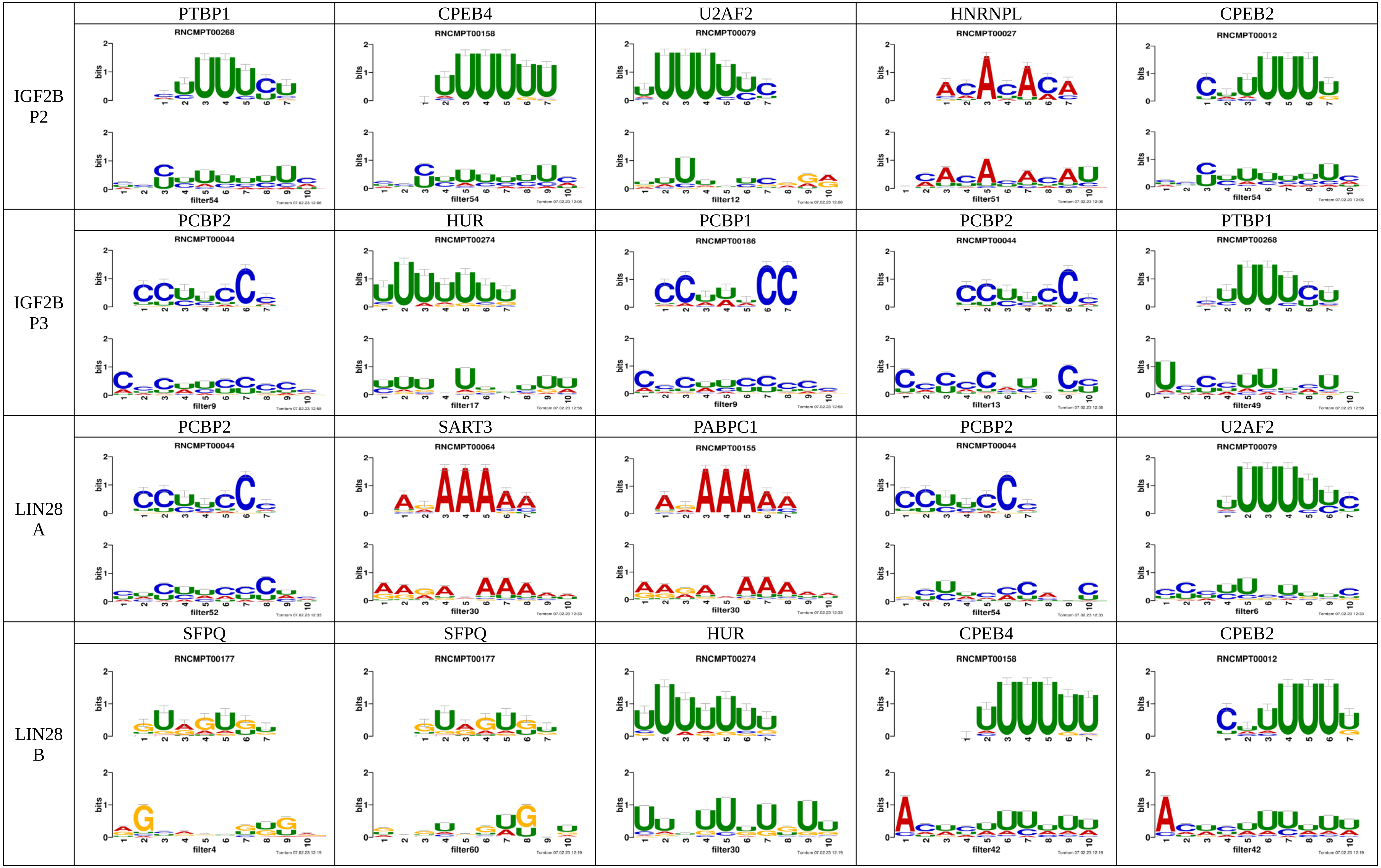
IGF2BP2
PTBP1
RNCMPT00268
filter54
CPEB4
RNCMPT00158
filter54
U2AF2
RNCMPT00079
filter12
HNRNPL
RNCMPT00027
filter51
CPEB2
RNCMPT00012
filter54
IGF2BP3
PCBP2
RNCMPT00044
filter9
HUR
RNCMPT00274
filter17
PCBP1
RNCMPT00186
filter9
PCBP2
RNCMPT00044
filter13
PTBP1
RNCMPT00268
filter49
LIN28A
PCBP2
RNCMPT00044
filter52
SART3
RNCMPT00064
filter30
PABPC1
RNCMPT00155
filter30
PCBP2
RNCMPT00044
filter54
U2AF2
RNCMPT00079
filter6
LIN28B
SFPQ
RNCMPT00177
filter4
SFPQ
RNCMPT00177
filter60
HUR
RNCMPT00274
filter30
CPEB4
RNCMPT00158
filter42
CPEB2
RNCMPT00012
filter42

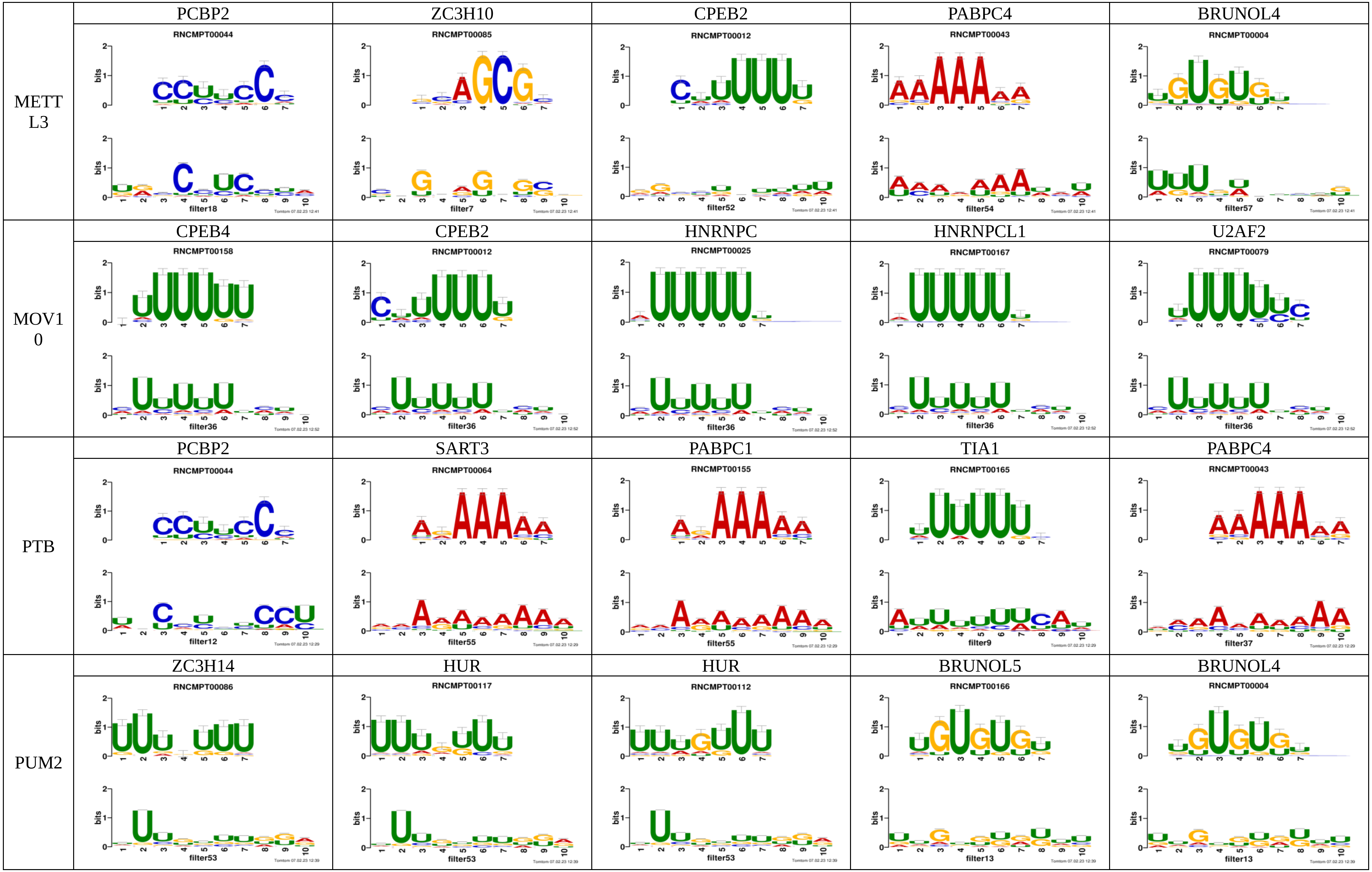
METTL3
PCBP2
RNCMPT00044
filter18
ZC3H10
RNCMPT00085
filter7
CPEB2
RNCMPT00012
filter52
PABPC4
RNCMPT00043
filter54
BRUNOL4
RNCMPT00004
filter57
MOV10
CPEB4
RNCMPT00158
filter36
CPEB2
RNCMPT00012
filter36
HNRNPC
RNCMPT00025
filter36
HNRNPCL1
RNCMPT00167
filter36
U2AF2
RNCMPT00079
filter36
PTB
PCBP2
RNCMPT00044
filter12
SART3
RNCMPT00064
filter55
PABPC1
RNCMPT00155
filter55
TIA1
RNCMPT00165
filter9
PABPC4
RNCMPT00043
filter37
PUM2
ZC3H14
RNCMPT00086
filter53
HUR
RNCMPT00117
filter53
HUR
RNCMPT00112
filter53
BRUNOL5
RNCMPT00166
filter13
BRUNOL4
RNCMPT00004
filter13

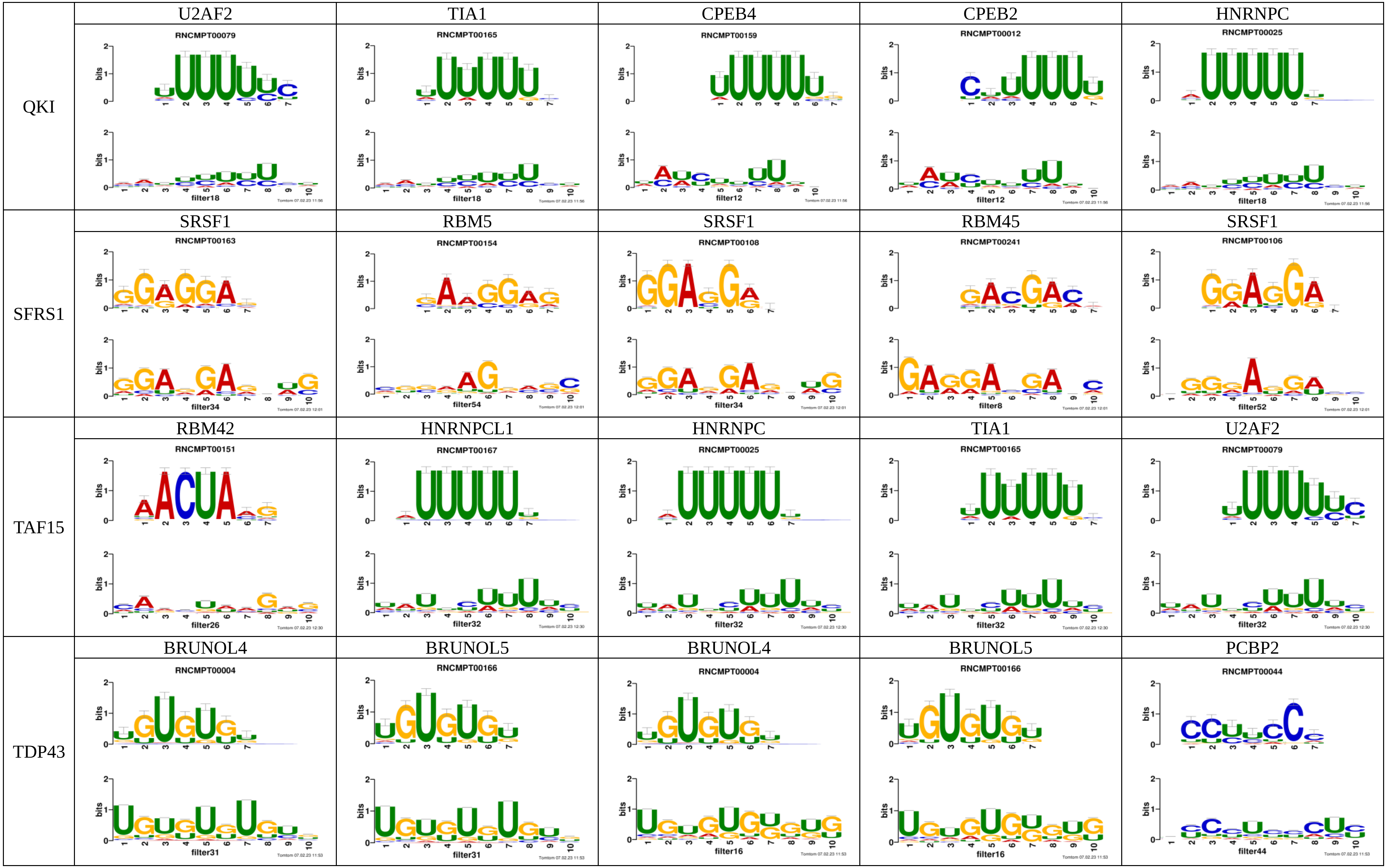
QKI
U2AF2
RNCMPT00079
filter18
TIA1
RNCMPT00165
filter18
CPEB4
RNCMPT00159
filter12
CPEB2
RNCMPT00012
filter12
HNRNPC
RNCMPT00025
filter18
SFRS1
SRSF1
RNCMPT00163
filter34
RBM5
RNCMPT00154
filter54
SRSF1
RNCMPT00108
filter34
RBM45
RNCMPT00241
filter8
SRSF1
RNCMPT00106
filter52
TAF15
RBM42
RNCMPT00151
filter26
HNRNPCL1
RNCMPT00167
filter32
HNRNPC
RNCMPT00025
filter32
TIA1
RNCMPT00165
filter32
U2AF2
RNCMPT00079
filter32
TDP43
BRUNOL4
RNCMPT00004
filter31
BRUNOL5
RNCMPT00166
filter31
BRUNOL4
RNCMPT00004
filter16
BRUNOL5
RNCMPT00166
filter16
PCBP2
RNCMPT00044
filter44

| | | | | | |
|---|---|---|---|---|---|
| TIA1 | HUR | HUR | ZC3H14 | TIA1 | PTBP1 |
| | 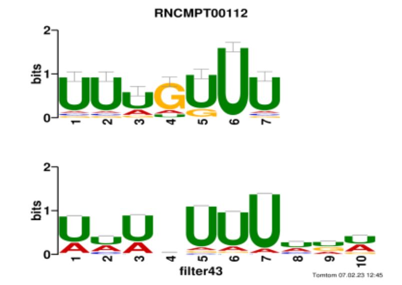  | 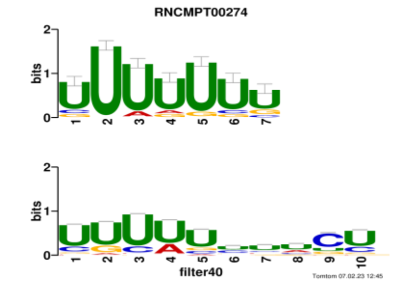  | 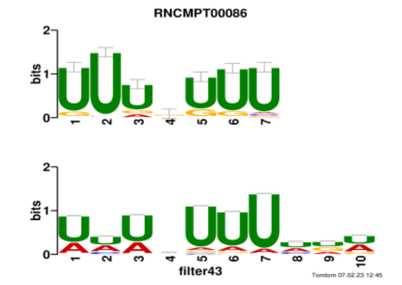  | 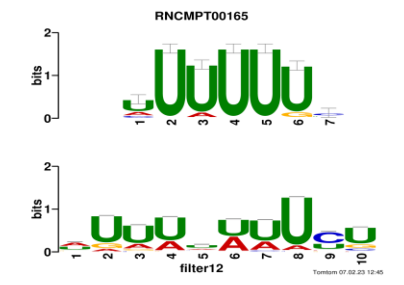  | 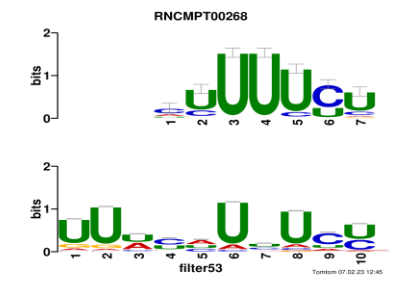  |
| TIAL1 | HUR | HUR | HUR | HUR | PCBP2 |
| | 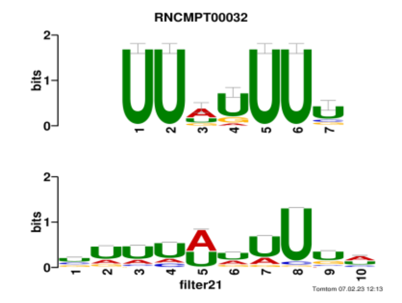  | 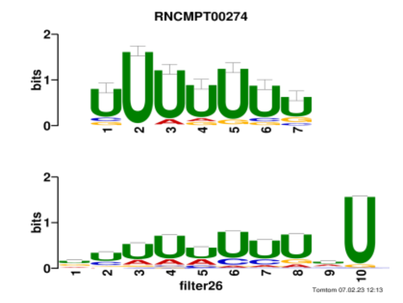  | 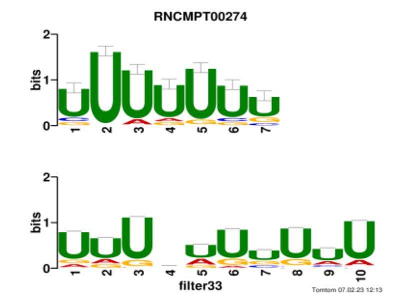  | 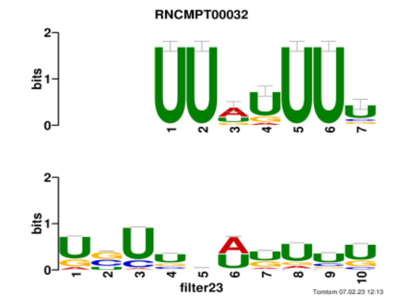  | 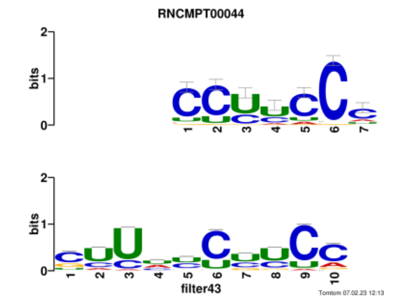  |
| TNRC 6 | PCBP1 | HNRNPC | HNRNPCL1 | HUR | QKI |
| | 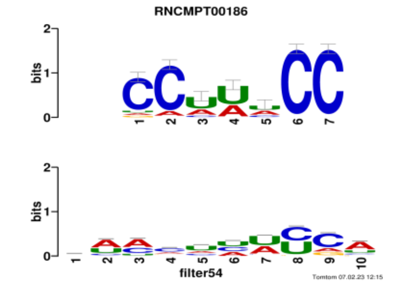  | 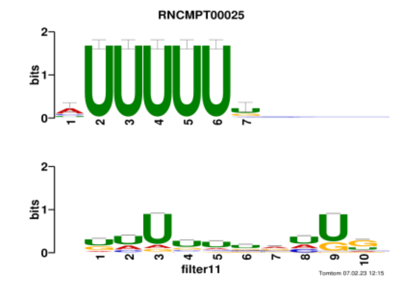  | 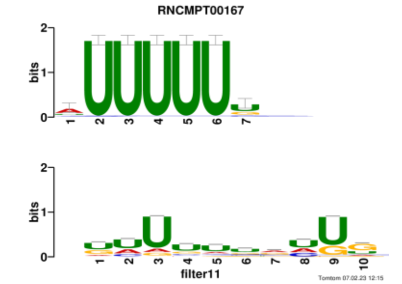  | 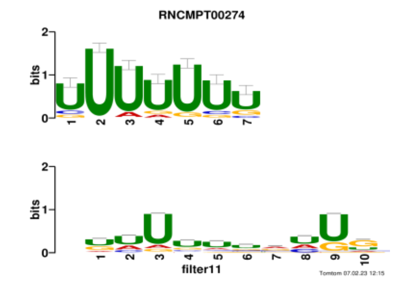  | 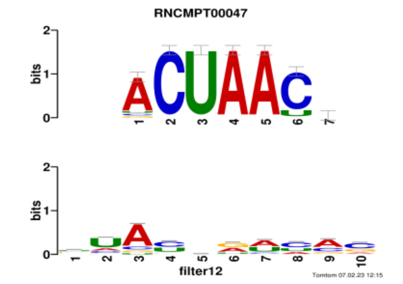  |
| U2AF 65 | U2AF2 | HUR | CPEB4 | PTBP1 | TIA1 |
| | 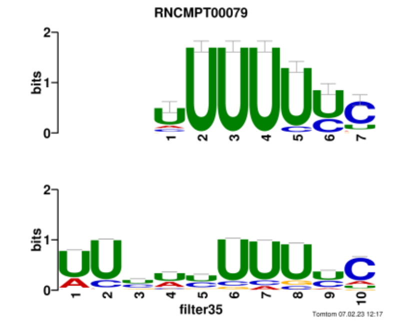  | 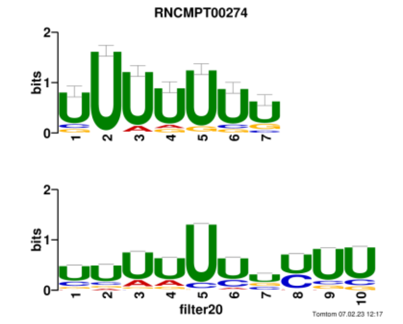  | 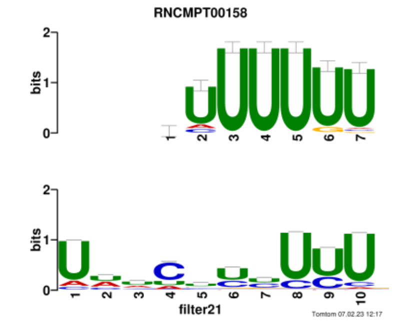  | 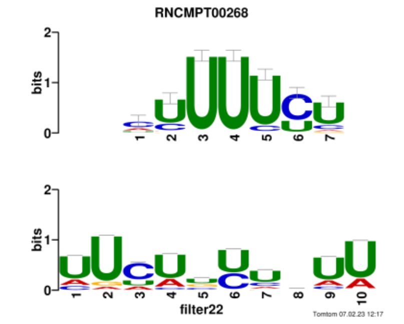  | 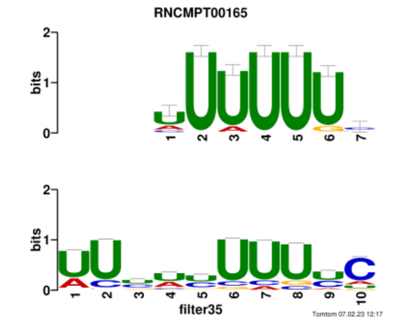  |

| | | | | | |
|---|---|---|---|---|---|
| WTAP | PCBP2 | HNRNPH2 | RBM45 | SRSF10 | SRSF10 |
| | 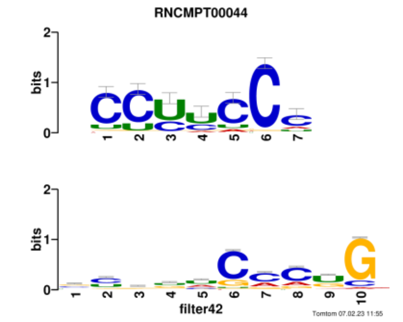  | 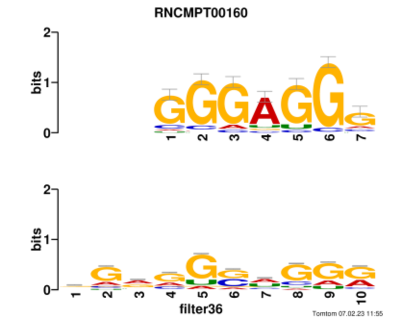  | 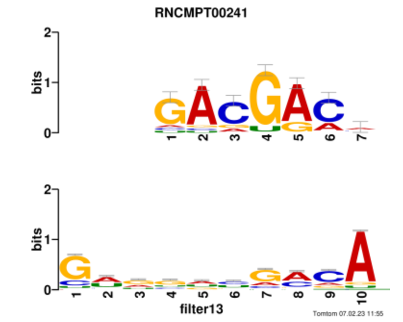  | 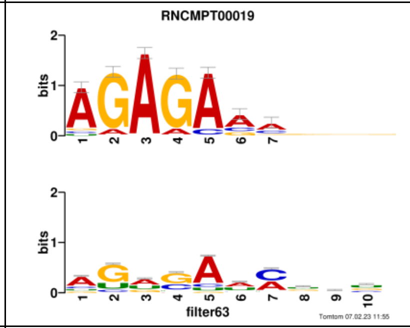  | 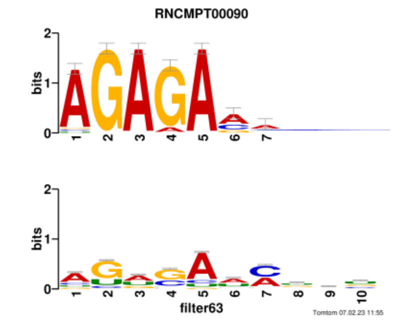  |
| ZC3H 7B | HUR | HNRNPCL1 | HNRNPC | U2AF2 | HUR |
| | 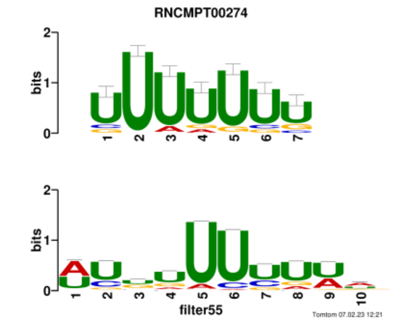  | 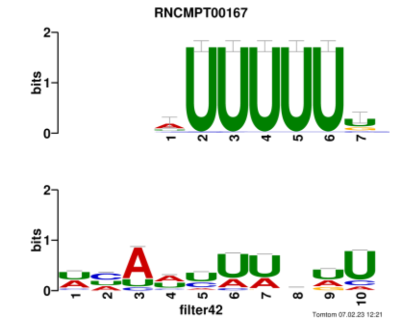  | 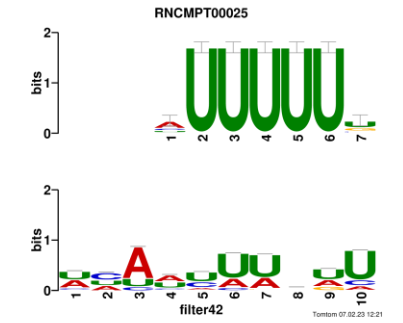  | 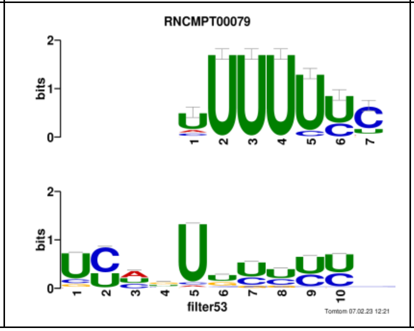  | 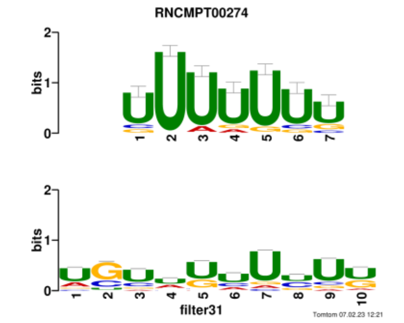  |

**Table S19.** Top5 Motif found by circSLNN in each dataset. Here, the sum of p-value, e-value and q-value was used to rank all the matched motifs (ascending order).

| Protein Name | Motif | | | | |
|---|---|---|---|---|---|
| AGO1 | PCBP2 | PCBP1 | RBM41 | PTBP1 | PTBP1 |
| | 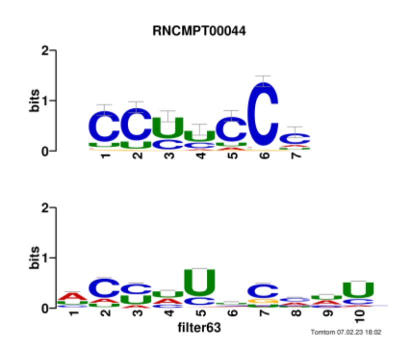 | 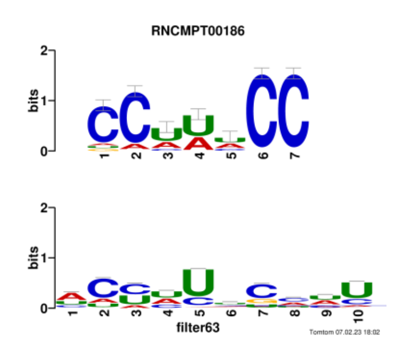 | 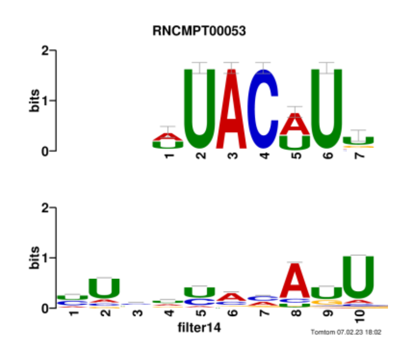 | 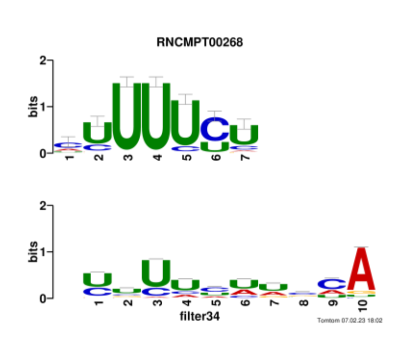 | 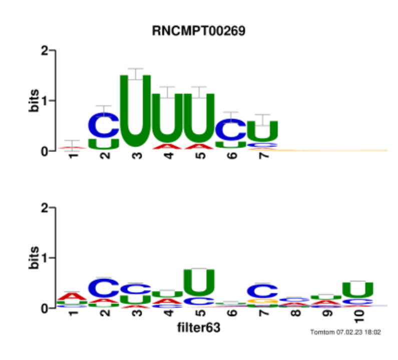 |
| AGO2 | HNRNPCL1 | HNRNPC | U2AF2 | PTBP1 | HNRNPL |
| | 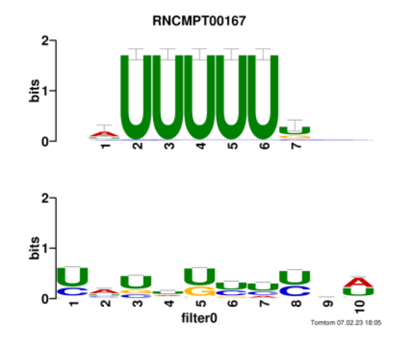 | 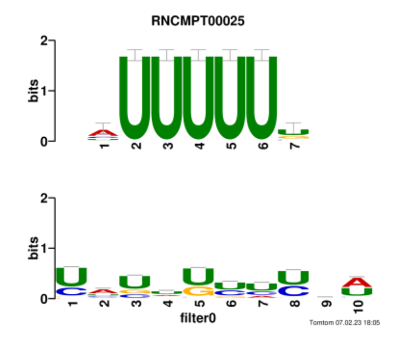 | 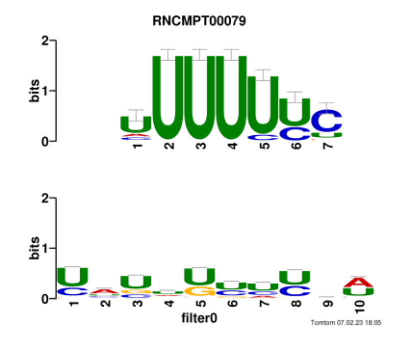 | 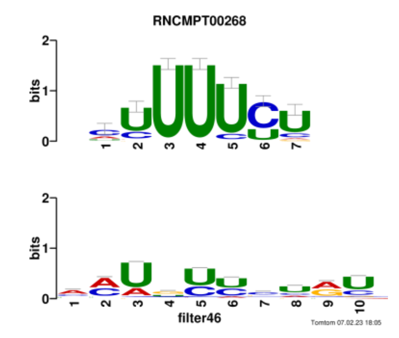 | 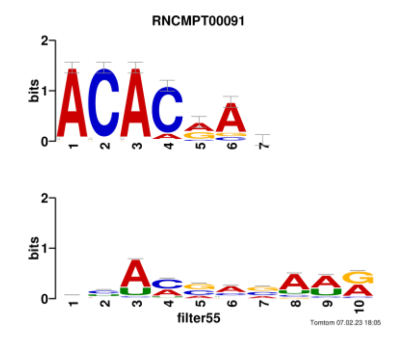 |
| AGO3 | TIA1 | RALY | TIA1 | CNOT4 | U2AF2 |
| | 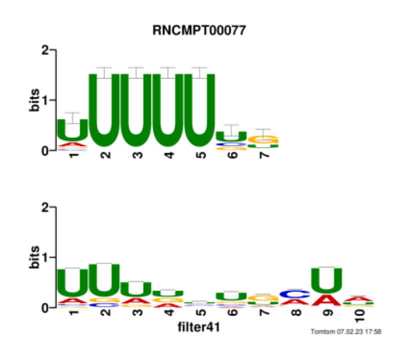 | 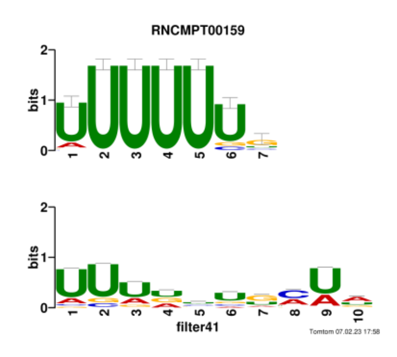 | 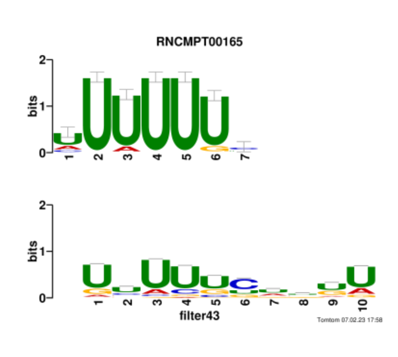 | 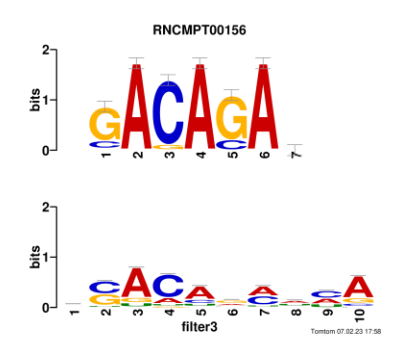 | 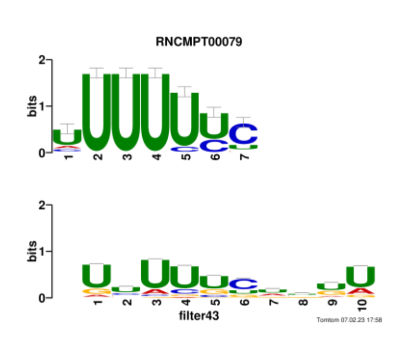 |

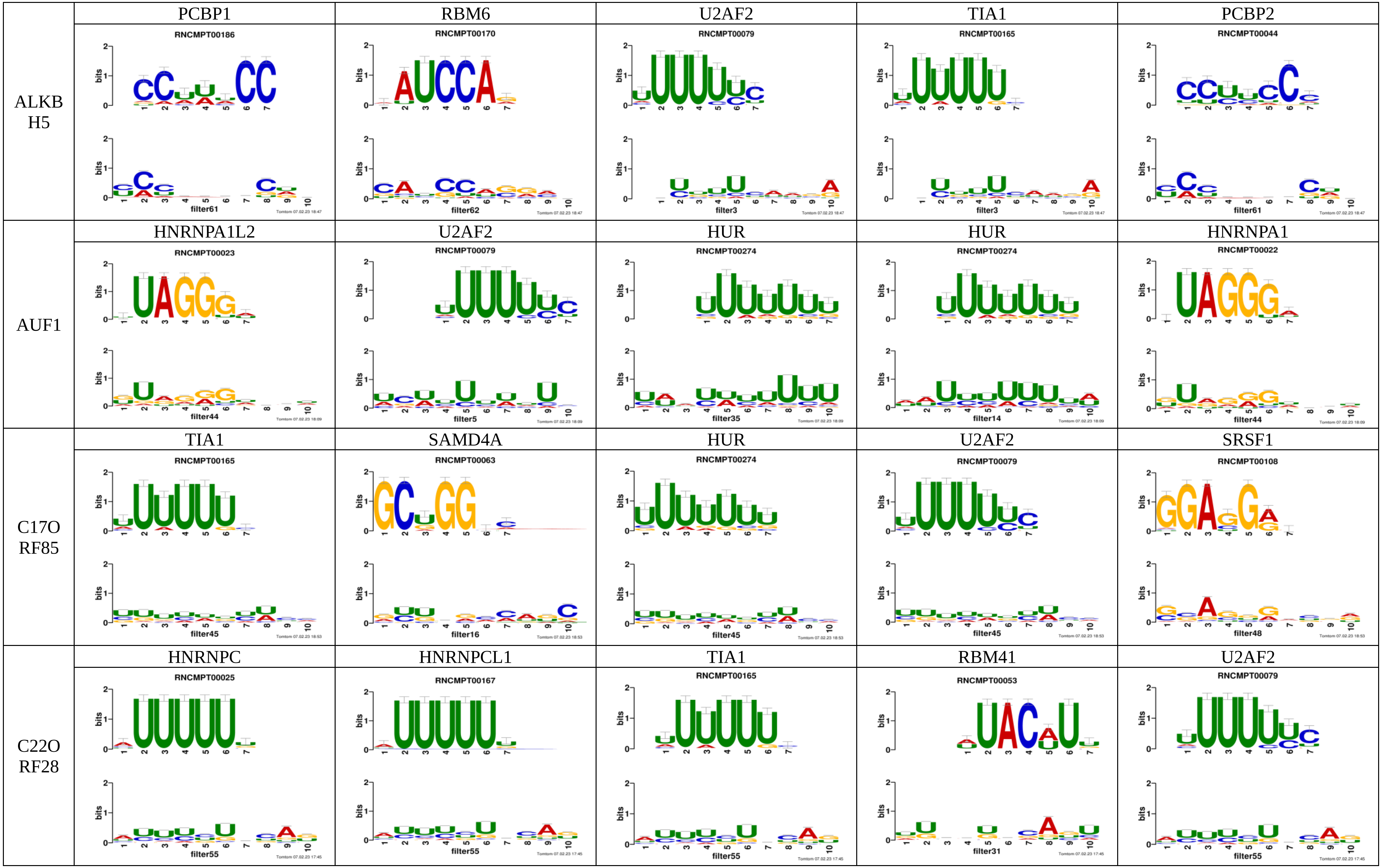
ALKBH5
PCBP1
RNCMPT00186
filter61
RBM6
RNCMPT00170
filter62
U2AF2
RNCMPT00079
filter3
TIA1
RNCMPT00165
filter3
PCBP2
RNCMPT00044
filter61
AUF1
HNRNPA1L2
RNCMPT00023
filter44
U2AF2
RNCMPT00079
filter5
HUR
RNCMPT00274
filter35
HUR
RNCMPT00274
filter14
HNRNPA1
RNCMPT00022
filter44
C17ORF85
TIA1
RNCMPT00165
filter45
SAMD4A
RNCMPT00063
filter16
HUR
RNCMPT00274
filter45
U2AF2
RNCMPT00079
filter45
SRSF1
RNCMPT00108
filter48
C22ORF28
HNRNPC
RNCMPT00025
filter55
HNRNPCL1
RNCMPT00167
filter55
TIA1
RNCMPT00165
filter55
RBM41
RNCMPT00053
filter31
U2AF2
RNCMPT00079
filter55

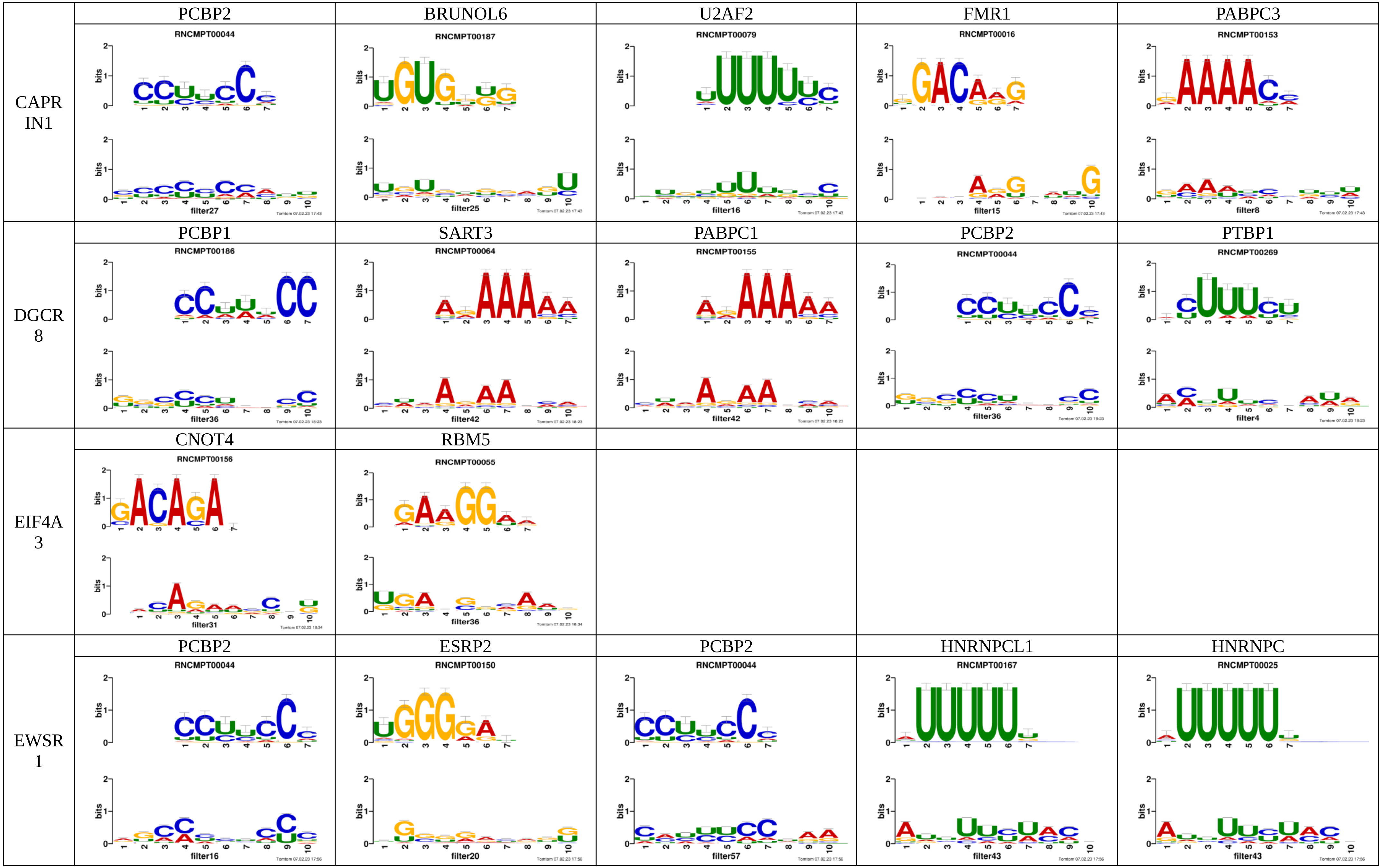
CAPRIN1
PCBP2
RNCMPT00044
filter27
BRUNOL6
RNCMPT00187
filter25
U2AF2
RNCMPT00079
filter16
FMR1
RNCMPT00016
filter15
PABPC3
RNCMPT00153
filter8
DGCR8
PCBP1
RNCMPT00186
filter36
SART3
RNCMPT00064
filter42
PABPC1
RNCMPT00155
filter42
PCBP2
RNCMPT00044
filter36
PTBP1
RNCMPT00269
filter4
EIF4A3
CNOT4
RNCMPT00156
filter31
RBM5
RNCMPT00055
filter36
EWSR1
PCBP2
RNCMPT00044
filter16
ESRP2
RNCMPT00150
filter20
PCBP2
RNCMPT00044
filter57
HNRNPCL1
RNCMPT00167
filter43
HNRNPC
RNCMPT00025
filter43

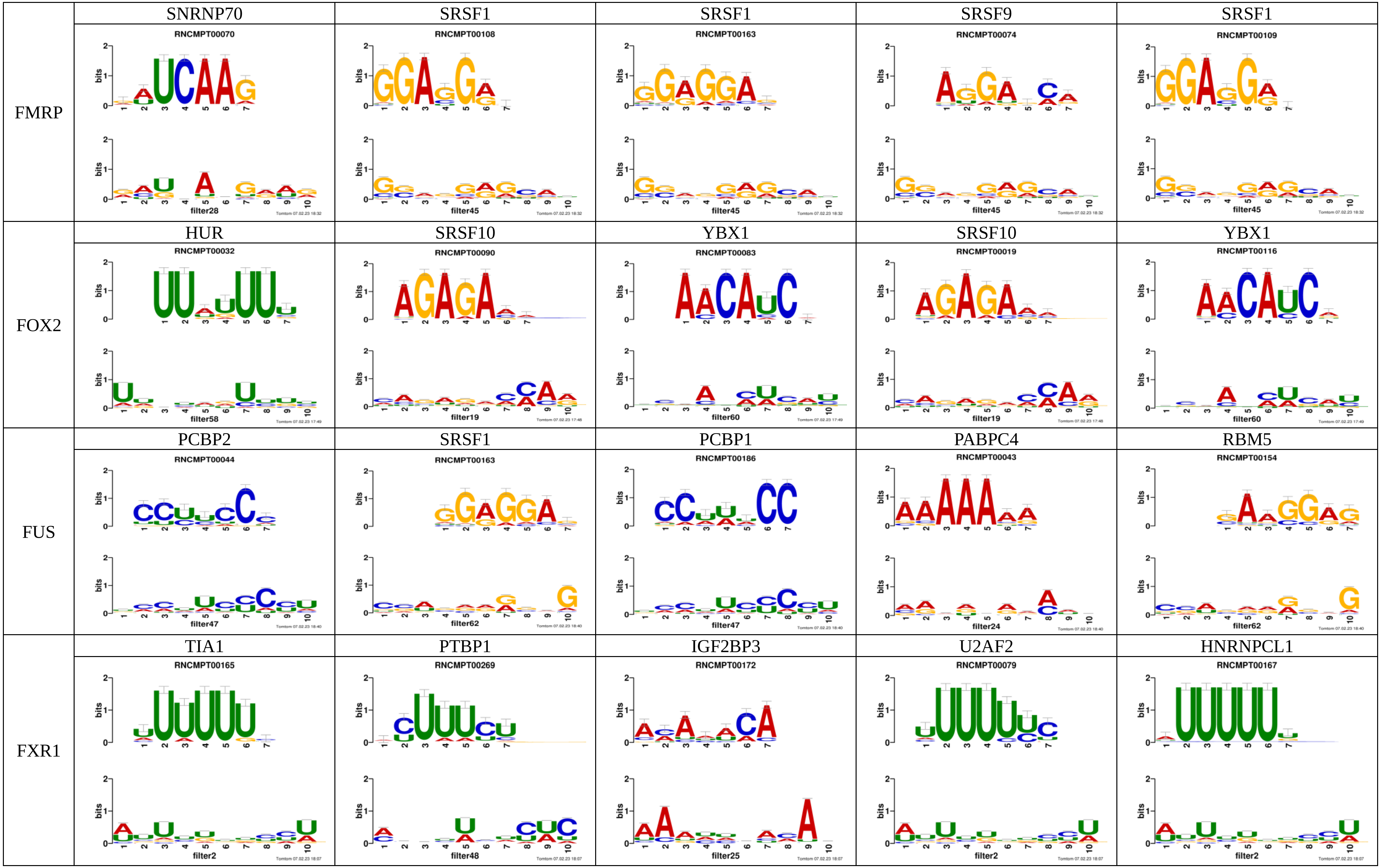
FMRP
SNRNP70
RNCMPT00070
filter28
SRSF1
RNCMPT00108
filter45
SRSF1
RNCMPT00163
filter45
SRSF9
RNCMPT00074
filter45
SRSF1
RNCMPT00109
filter45
FOX2
HUR
RNCMPT00032
filter58
SRSF10
RNCMPT00090
filter19
YBX1
RNCMPT00083
filter60
SRSF10
RNCMPT00019
filter19
YBX1
RNCMPT00116
filter60
FUS
PCBP2
RNCMPT00044
filter47
SRSF1
RNCMPT00163
filter62
PCBP1
RNCMPT00186
filter47
PABPC4
RNCMPT00043
filter24
RBM5
RNCMPT00154
filter62
FXR1
TIA1
RNCMPT00165
filter2
PTBP1
RNCMPT00269
filter48
IGF2BP3
RNCMPT00172
filter25
U2AF2
RNCMPT00079
filter2
HNRNPCL1
RNCMPT00167
filter2

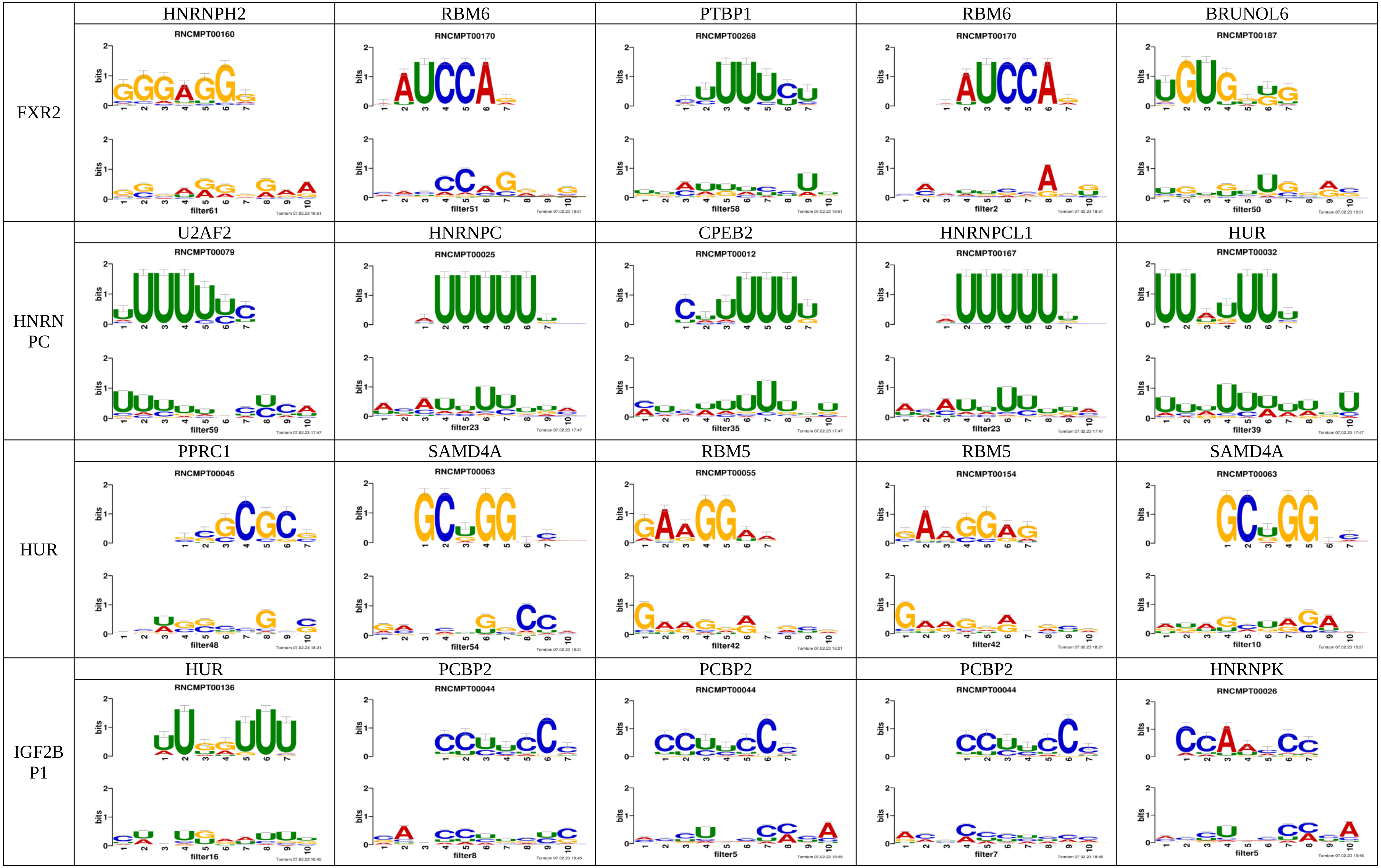

FXR2
HNRNPH2
RNCMPT00160
filter61
RBM6
RNCMPT00170
filter51
PTBP1
RNCMPT00268
filter58
RBM6
RNCMPT00170
filter2
BRUNOL6
RNCMPT00187
filter50
HNRNPC
U2AF2
RNCMPT00079
filter59
HNRNPC
RNCMPT00025
filter23
CPEB2
RNCMPT00012
filter35
HNRNPCL1
RNCMPT00167
filter23
HUR
RNCMPT00032
filter39
HUR
PPRC1
RNCMPT00045
filter48
SAMD4A
RNCMPT00063
filter54
RBM5
RNCMPT00055
filter42
RBM5
RNCMPT00154
filter42
SAMD4A
RNCMPT00063
filter10
IGF2BP1
HUR
RNCMPT00136
filter16
PCBP2
RNCMPT00044
filter8
PCBP2
RNCMPT00044
filter5
PCBP2
RNCMPT00044
filter7
HNRNPK
RNCMPT00026
filter5
bits

| IGF2BP2 | PTBP1 | CPEB4 | CPEB2 | PTBP1 | PCBP1 |
|---|---|---|---|---|---|

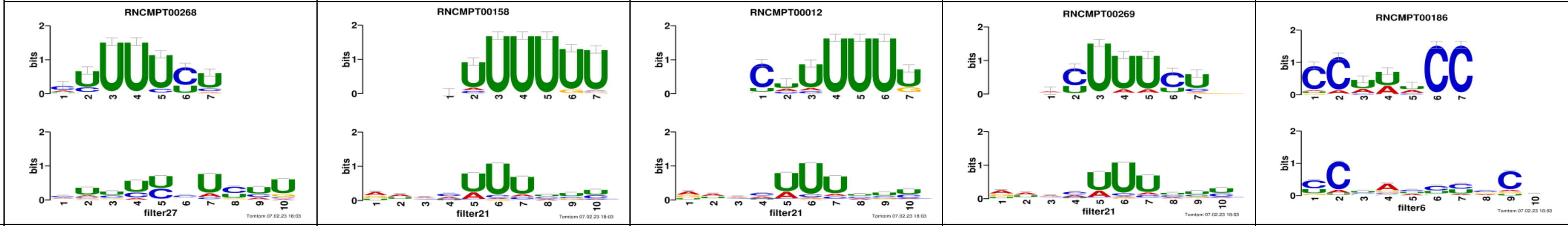


| IGF2BP3 | HUR | U2AF2 | CPEB4 | CPEB2 | CPEB4 |
|---|---|---|---|---|---|

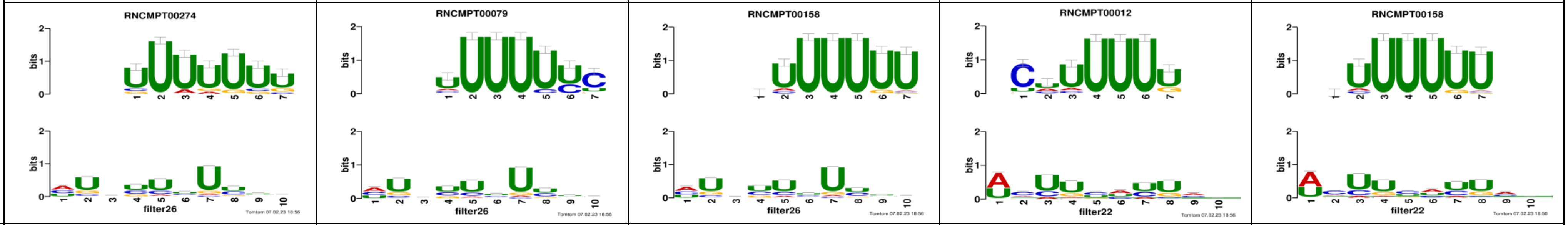


| LIN28A | RBM5 | HUR | SRSF1 | HUR | HNRNPC |
|---|---|---|---|---|---|

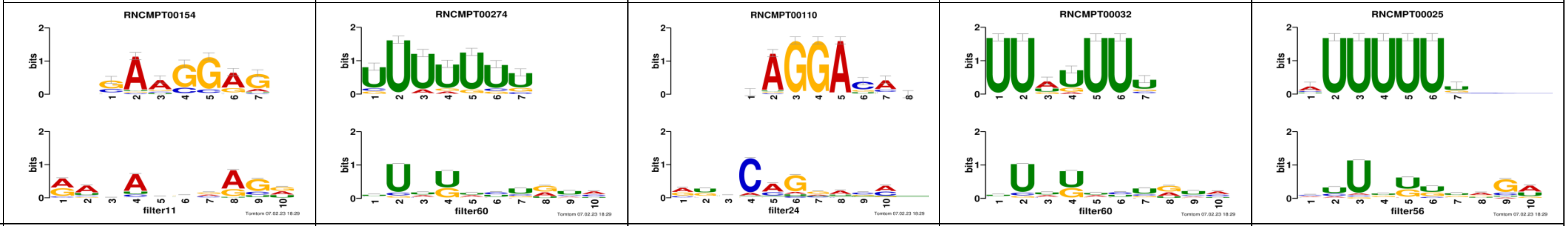


| LIN28B | CPEB2 | CPEB4 | G3BP2 | PTBP1 | CPEB2 |
|---|---|---|---|---|---|

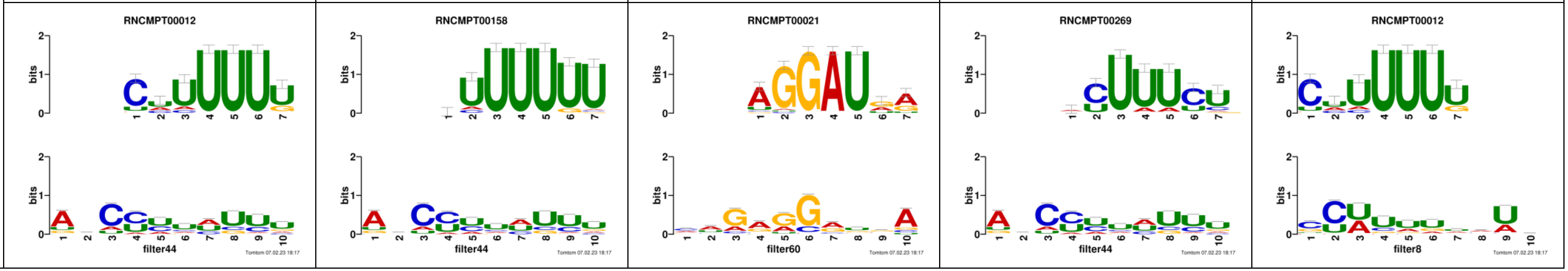

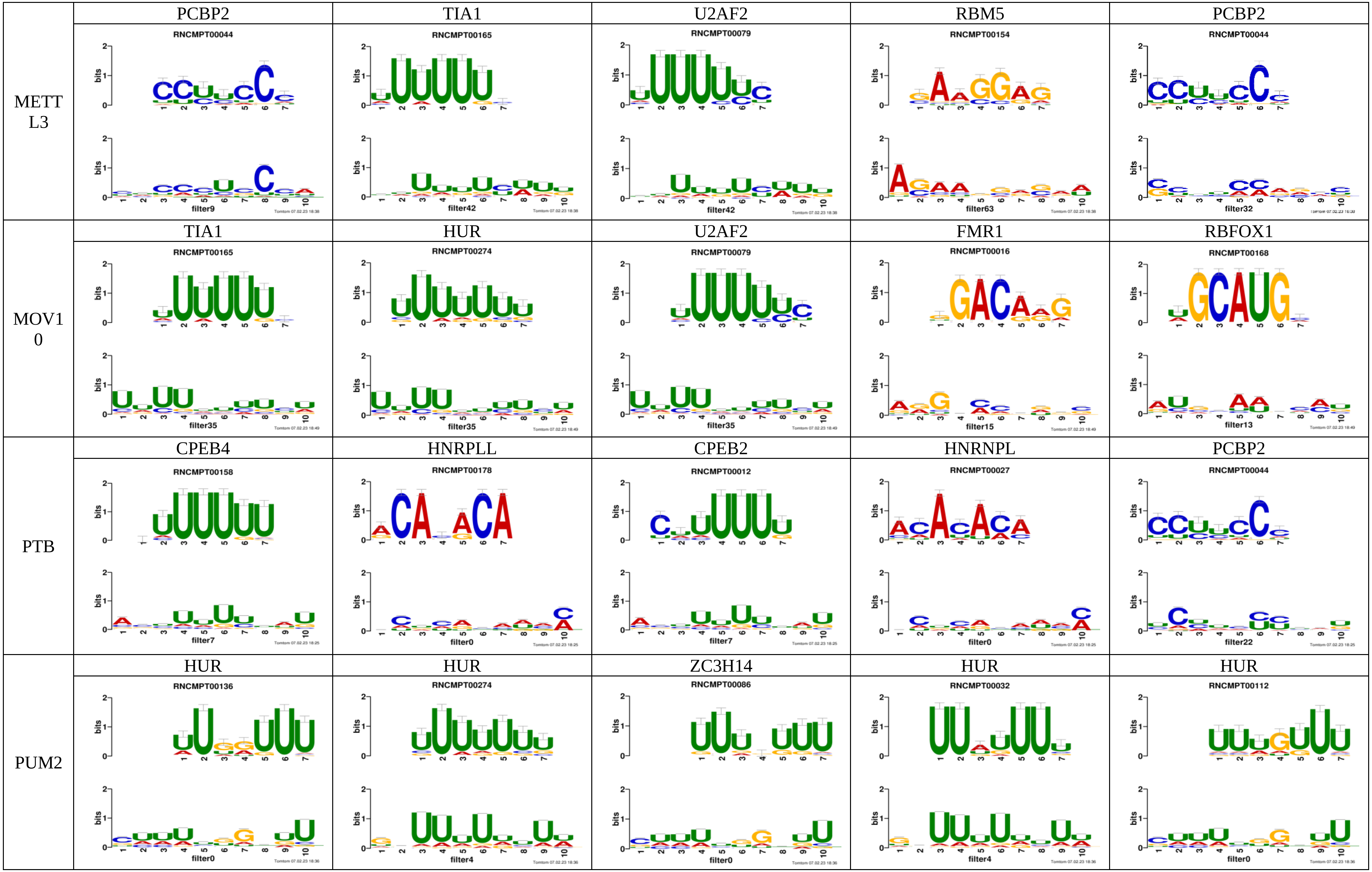
METTL3
PCBP2
RNCMPT00044
filter9
TIA1
RNCMPT00165
filter42
U2AF2
RNCMPT00079
filter42
RBM5
RNCMPT00154
filter63
PCBP2
RNCMPT00044
filter32
MOV10
TIA1
RNCMPT00165
filter35
HUR
RNCMPT00274
filter35
U2AF2
RNCMPT00079
filter35
FMR1
RNCMPT00016
filter15
RBFOX1
RNCMPT00168
filter13
PTB
CPEB4
RNCMPT00158
filter7
HNRPLL
RNCMPT00178
filter0
CPEB2
RNCMPT00012
filter7
HNRNPL
RNCMPT00027
filter0
PCBP2
RNCMPT00044
filter22
PUM2
HUR
RNCMPT00136
filter0
HUR
RNCMPT00274
filter4
ZC3H14
RNCMPT00086
filter0
HUR
RNCMPT00032
filter4
HUR
RNCMPT00112
filter0

| | | | | | |
|---|---|---|---|---|---|
| QKI | HNRNPH2 | PCBP2 | PCBP2 | PTBP1 | RALY |
| | 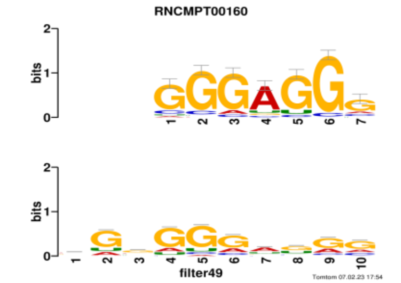  | 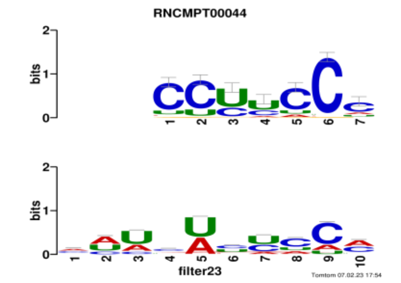  | 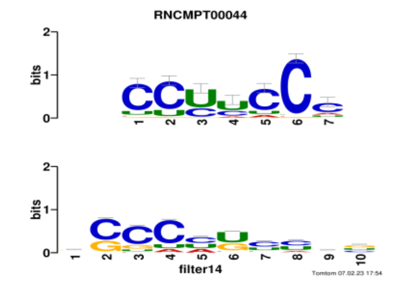  | 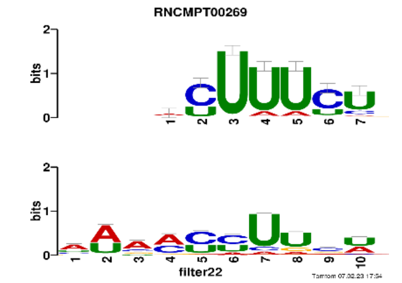  | 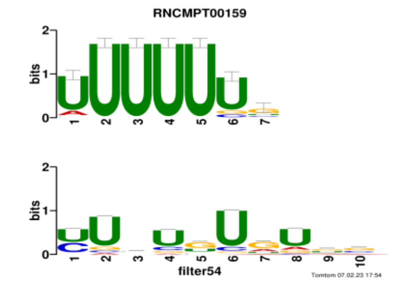  |
| SFRS1 | SRSF10 | SRSF10 | SAMD4A | PCBP2 | SRSF10 |
| | 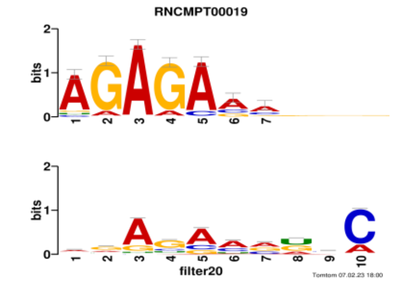  | 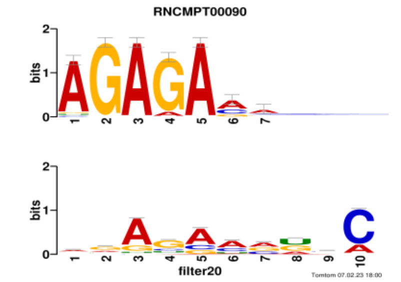  | 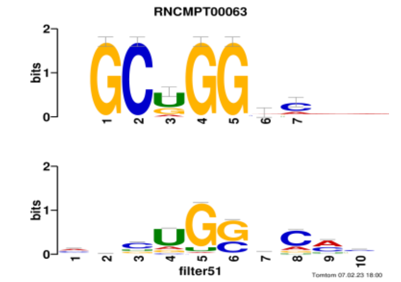  | 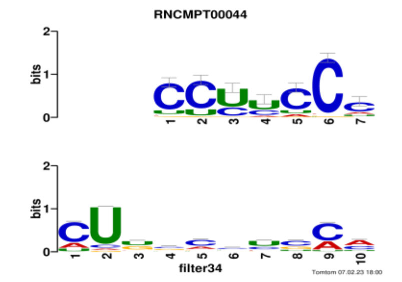  | 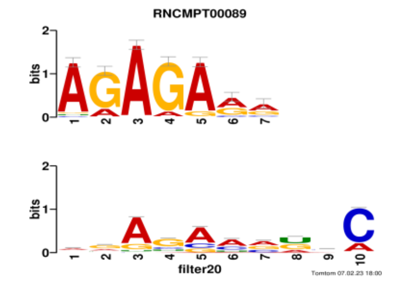  |
| TAF15 | IGF2BP3 | HUR | HUR | HNRNPH2 | BRUNOL4 |
| | 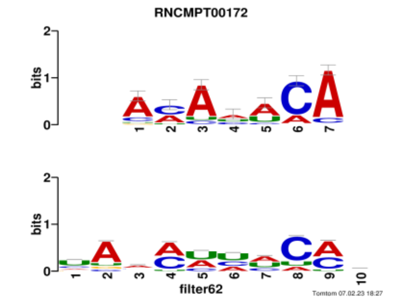  | 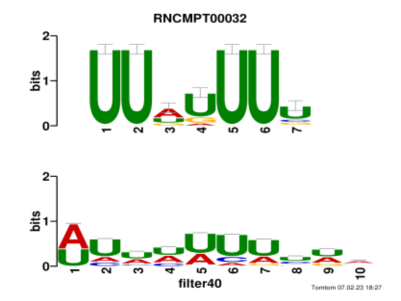  | 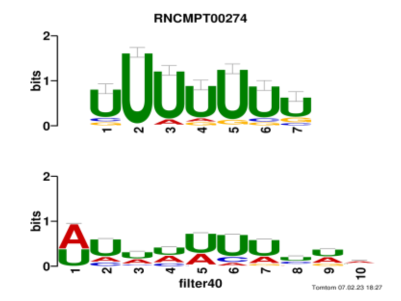  | 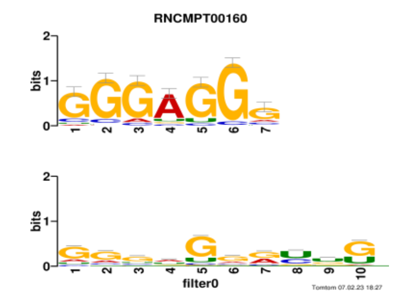  | 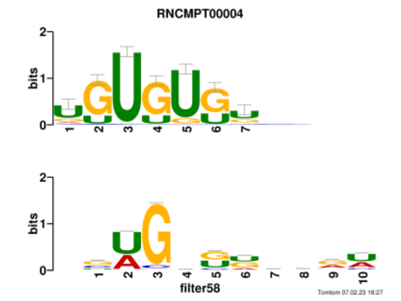  |
| TDP43 | PTBP1 | PCBP2 | PTBP1 | HUR | HUR |
| | 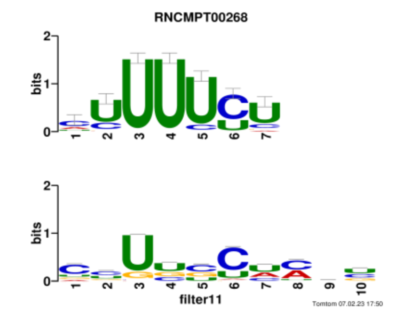  | 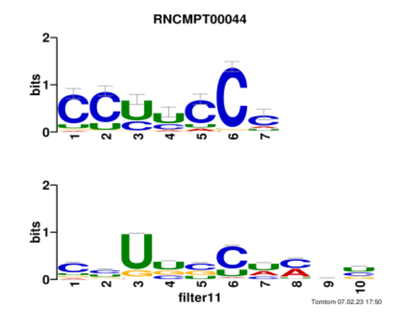  | 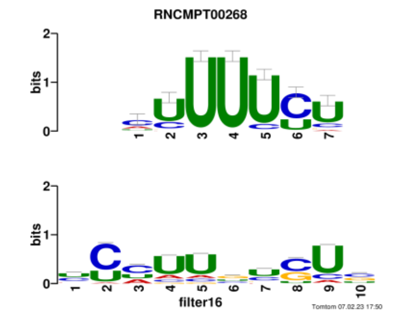  | 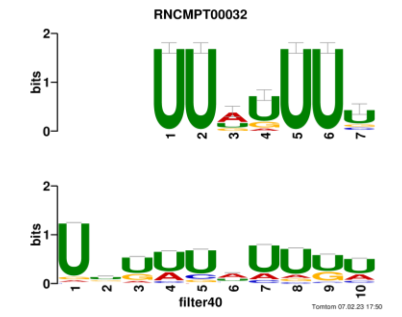  | 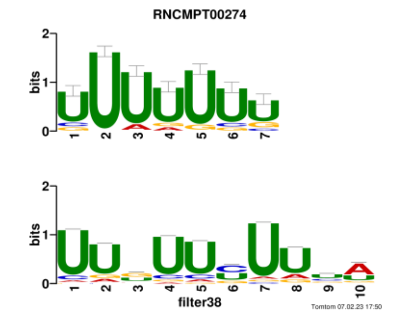  |

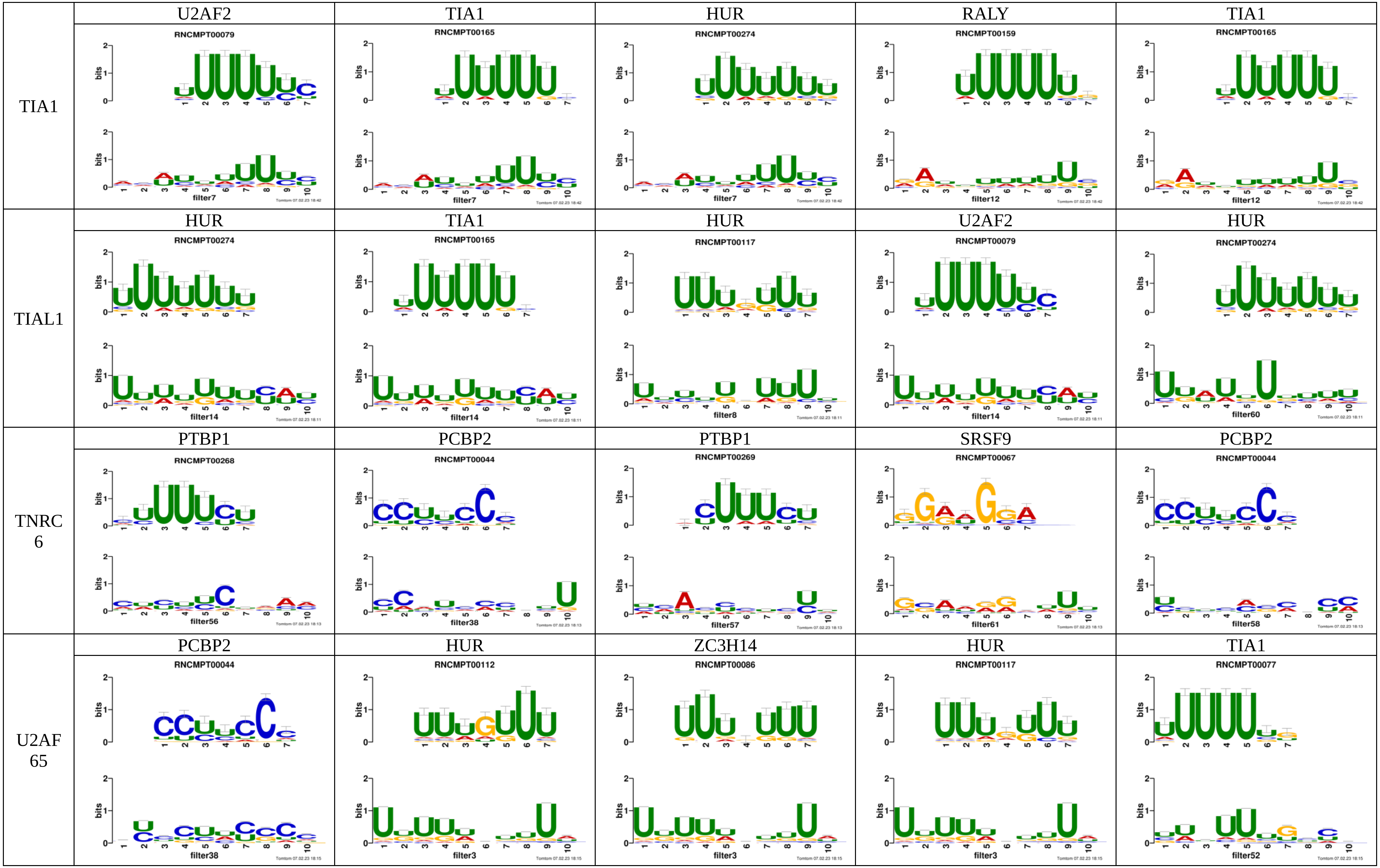
TIA1
U2AF2
RNCMPT00079
filter7
TIA1
RNCMPT00165
filter7
HUR
RNCMPT00274
filter7
RALY
RNCMPT00159
filter12
TIA1
RNCMPT00165
filter12
TIAL1
HUR
RNCMPT00274
filter14
TIA1
RNCMPT00165
filter14
HUR
RNCMPT00117
filter8
U2AF2
RNCMPT00079
filter14
HUR
RNCMPT00274
filter60
TNRC 6
PTBP1
RNCMPT00268
filter56
PCBP2
RNCMPT00044
filter38
PTBP1
RNCMPT00269
filter57
SRSF9
RNCMPT00067
filter61
PCBP2
RNCMPT00044
filter58
U2AF 65
PCBP2
RNCMPT00044
filter38
HUR
RNCMPT00112
filter3
ZC3H14
RNCMPT00086
filter3
HUR
RNCMPT00117
filter3
TIA1
RNCMPT00077
filter52

| | | | | | |
|---|---|---|---|---|---|
| WTAP | SRSF2 | PABPC5 | ZC3H10 | | |
| | 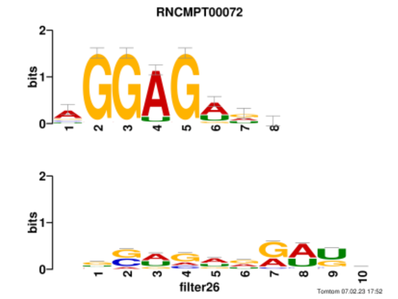 | 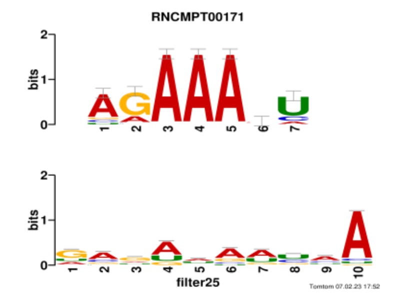 | 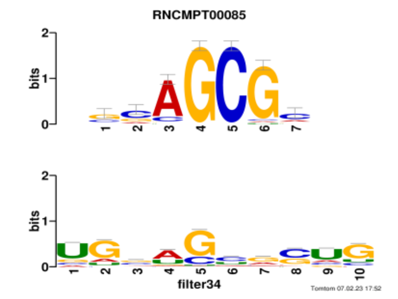 | | |
| ZC3H<br>7B | SAMD4A | HUR | HUR | HUR | ZC3H14 |
| | 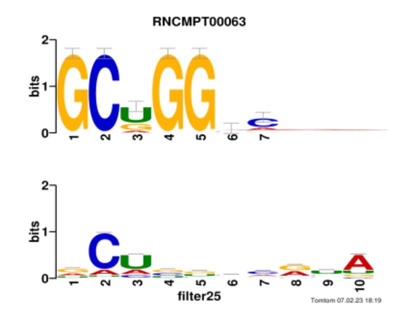 | 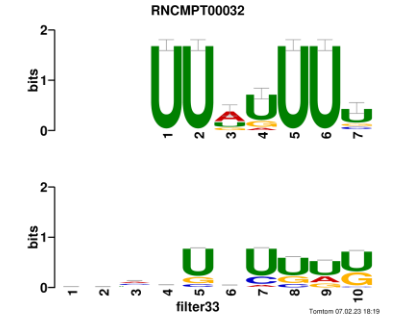 | 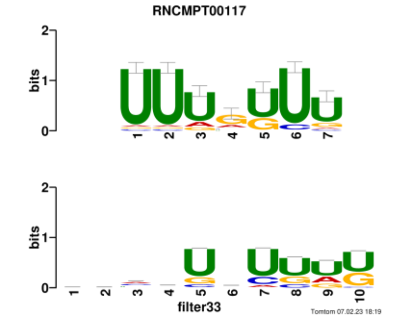 | 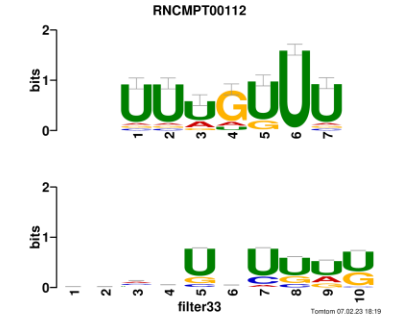 | 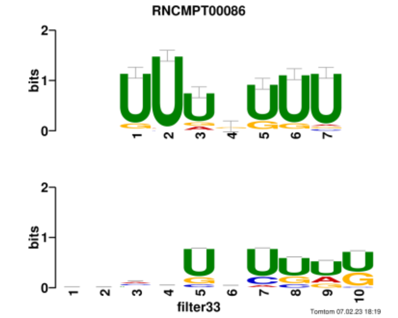 |

Table S20. Top5 Motif found by iCircRBP-DHN in each dataset. Here, the sum of p-value, e-value and q-value was used to rank all the matched motifs (ascending order).

| Protein Name | Motif | | | | |
|---|---|---|---|---|---|
| AGO1 | SART3 | PABPC1 | HUR | HUR | HUR |
| | 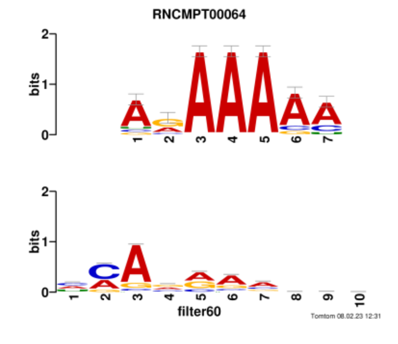 | 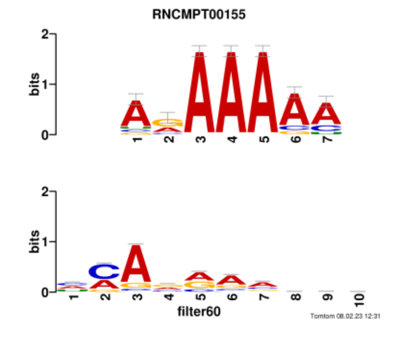 | 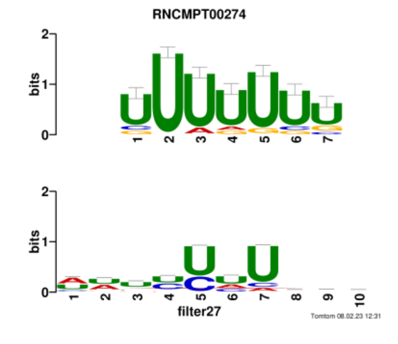 | 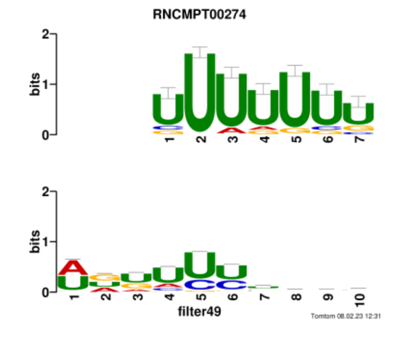 | 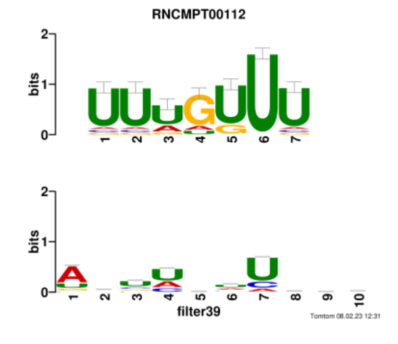 |
| AGO2 | HUR | HUR | U2AF2 | TIA1 | HUR |
| | 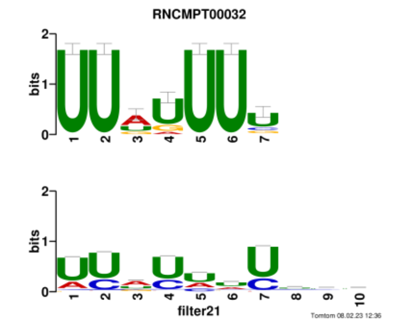 | 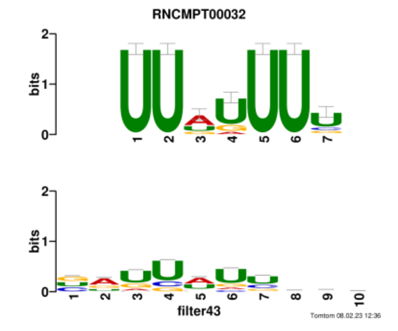 | 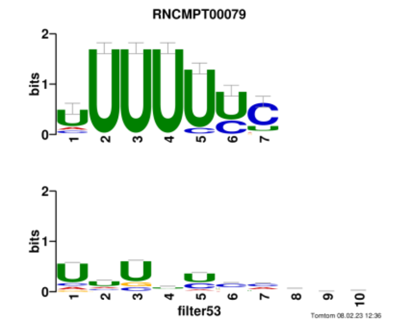 | 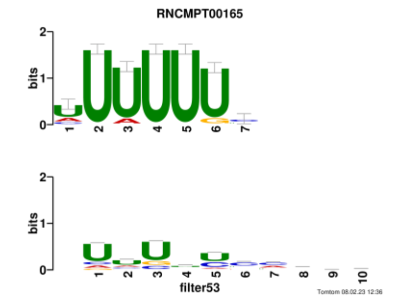 | 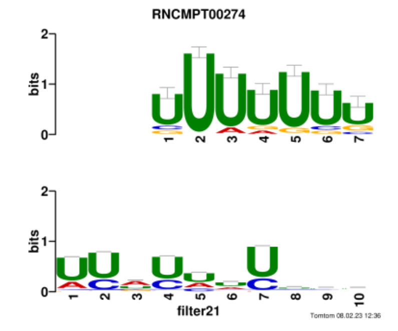 |
| AGO3 | U2AF2 | PCBP2 | HNRNPC | HNRNPCL1 | PCBP2 |
| | 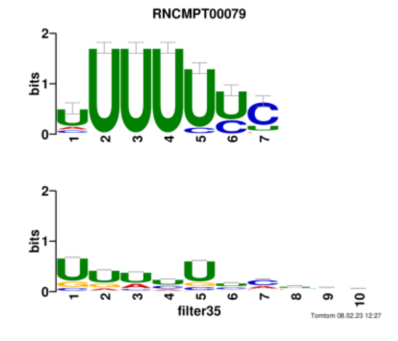 | 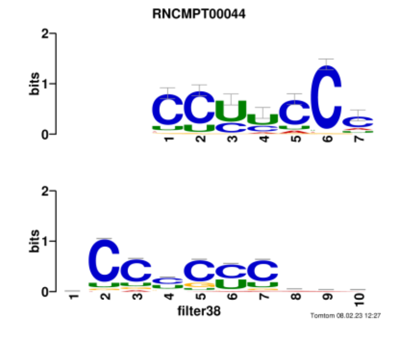 | 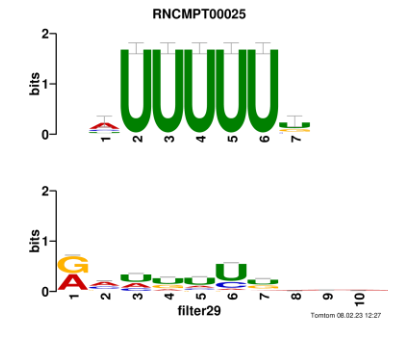 | 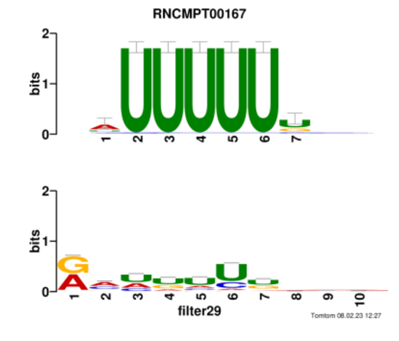 | 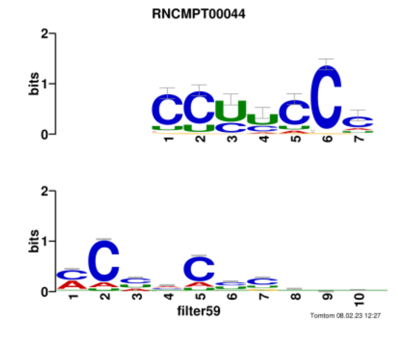 |

| | | | | | |
|---|---|---|---|---|---|
| ALKBH5 | TIA1 | HUR | TIA1 | U2AF2 | CPEB4 |
| | 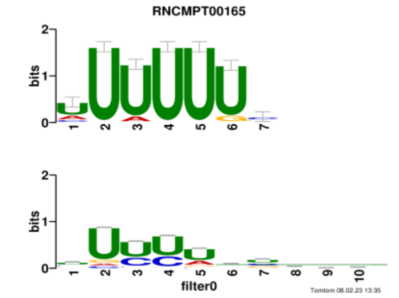  | 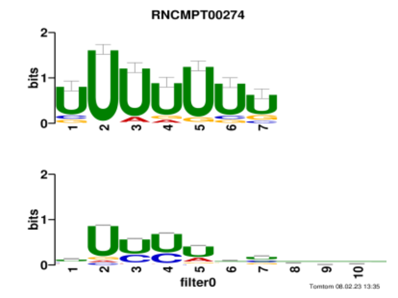  | 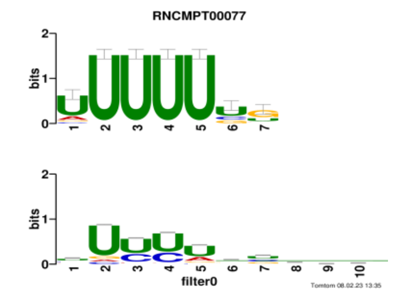  | 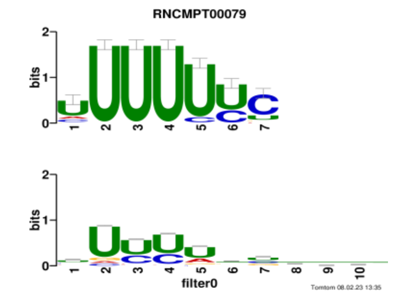  | 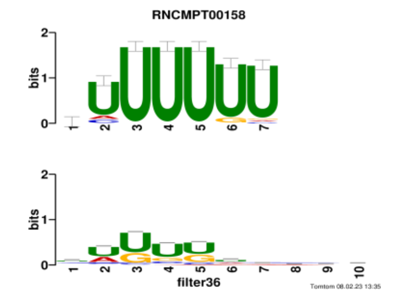  |
| AUF1 | CPEB4 | CPEB2 | HUR | HNRNPC | HNRNPCL1 |
| | 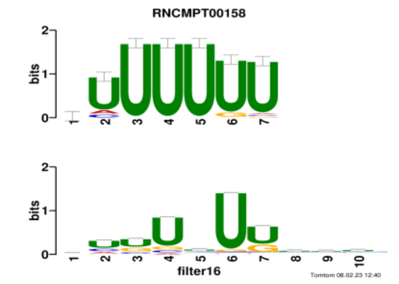  | 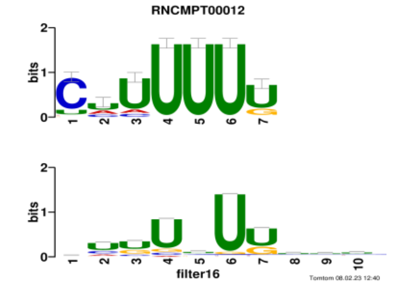  | 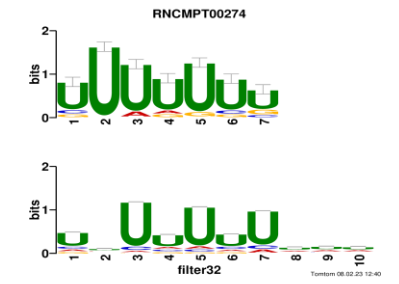  | 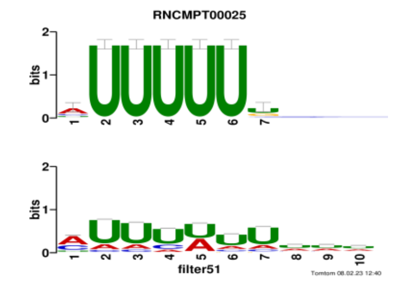  | 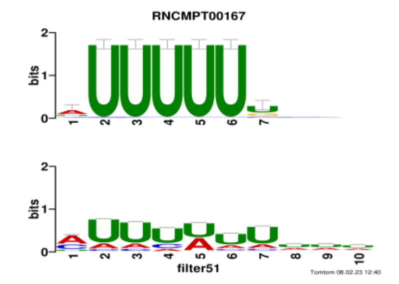  |
| C17ORF85 | PCBP2 | PABPC3 | HNRNPH2 | PABPC3 | PPRC1 |
| | 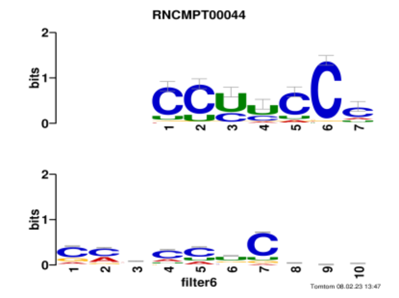  | 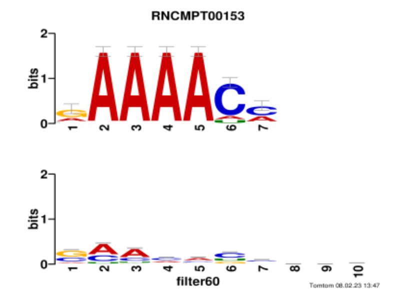  | 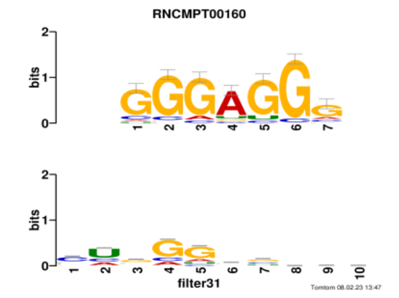  | 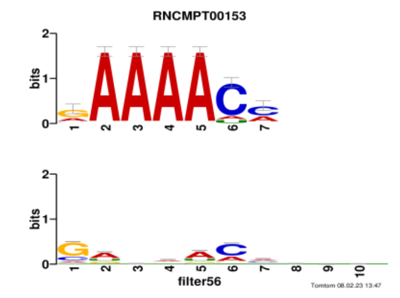  | 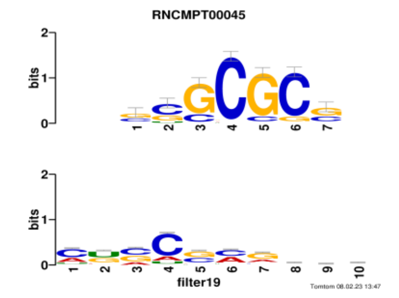  |
| C22ORF28 | RBM4 | RBM4 | TARDBP | CPEB4 | CPEB2 |
| | 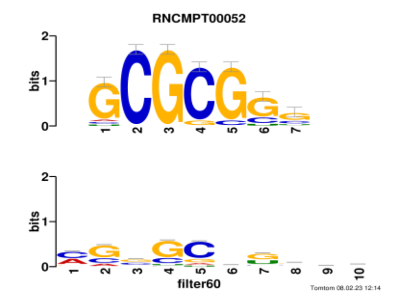  | 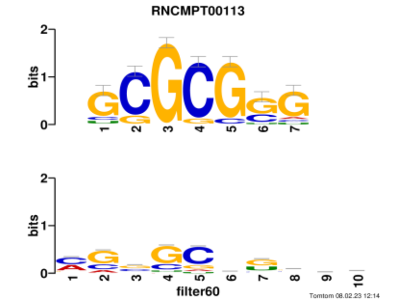  | 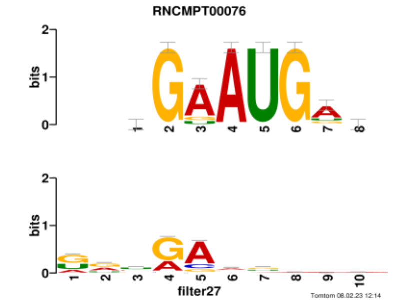  | 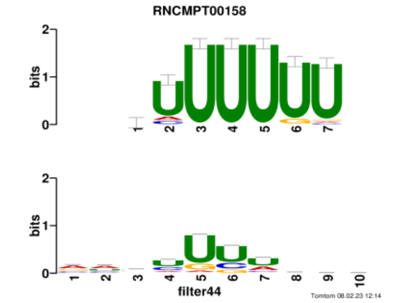  | 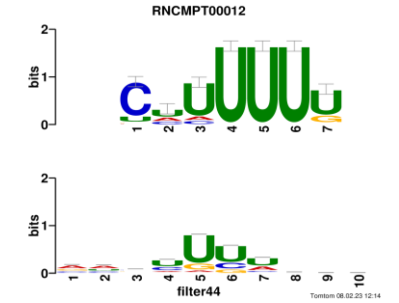  |

| | | | | | |
|---|---|---|---|---|---|
| CAPR<br>IN1 | RBM4 | RBM4 | CPEB4 | CPEB4 | HUR |
| | 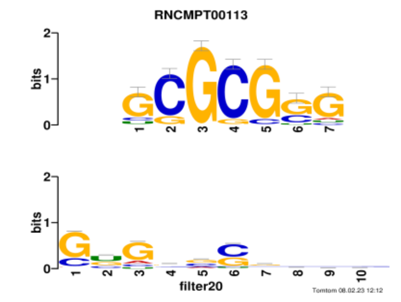 | 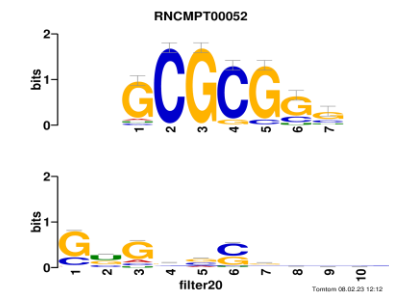 | 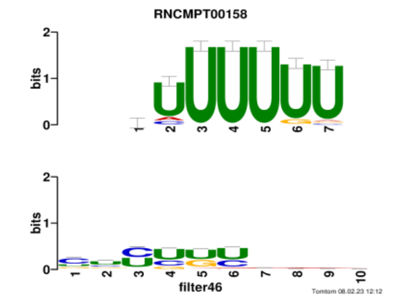 | 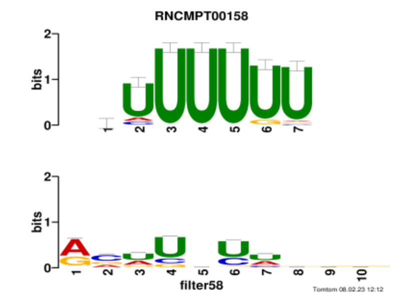 | 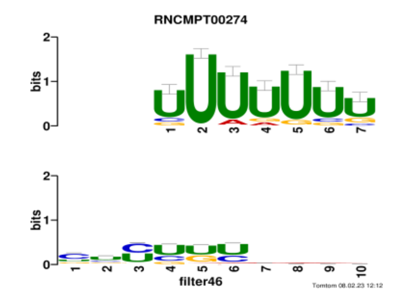 |
| DGCR<br>8 | SAMD4A | PPRC1 | CPEB2 | SRSF7 | CPEB4 |
| | 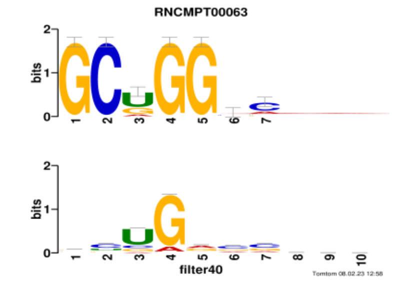 | 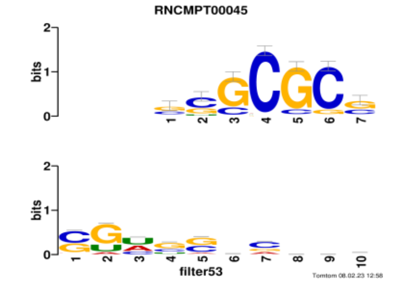 | 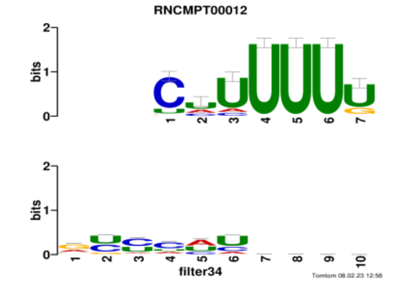 | 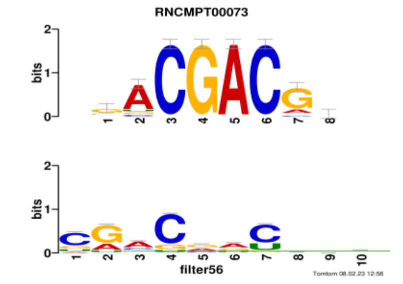 | 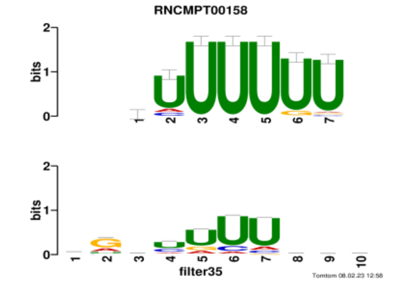 |
| EIF4A<br>3 | SRSF9 | SRSF10 | SRSF1 | SRSF10 | RBM28 |
| | 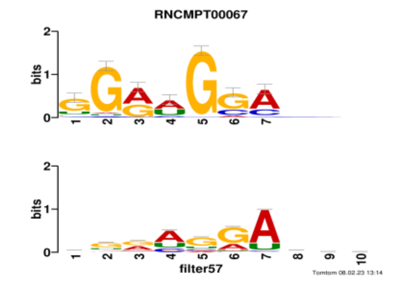 | 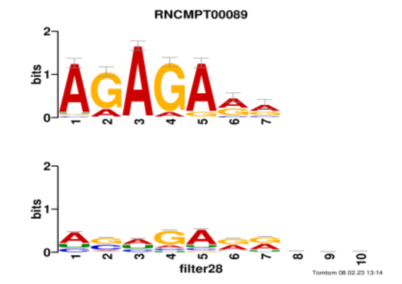 | 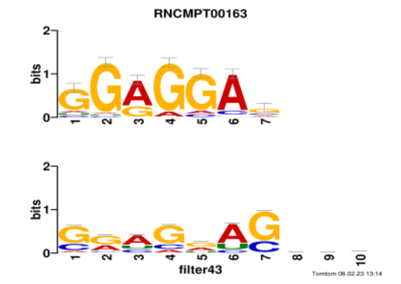 | 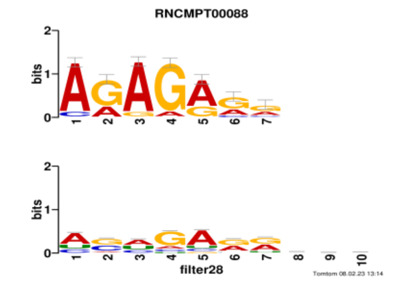 | 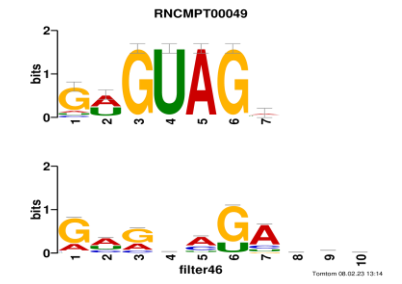 |
| EWSR<br>1 | MATR3 | HUR | CPEB4 | CPEB2 | CPEB4 |
| | 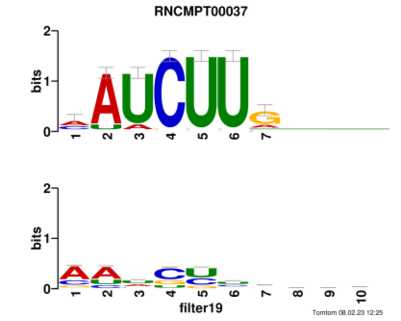 | 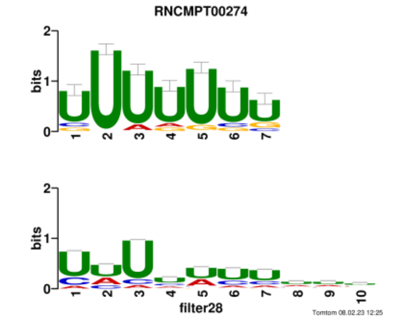 | 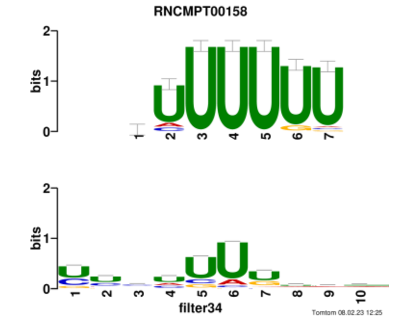 | 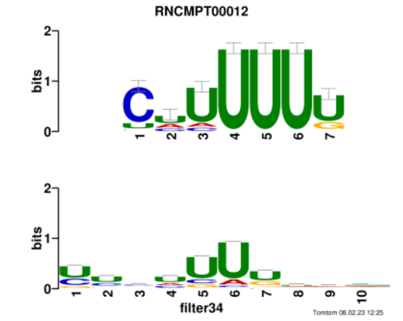 | 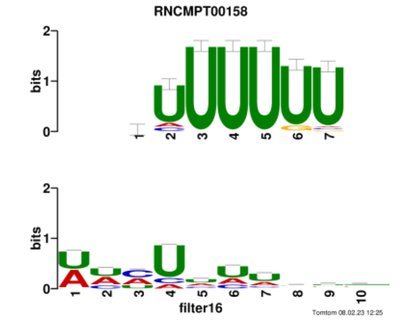 |

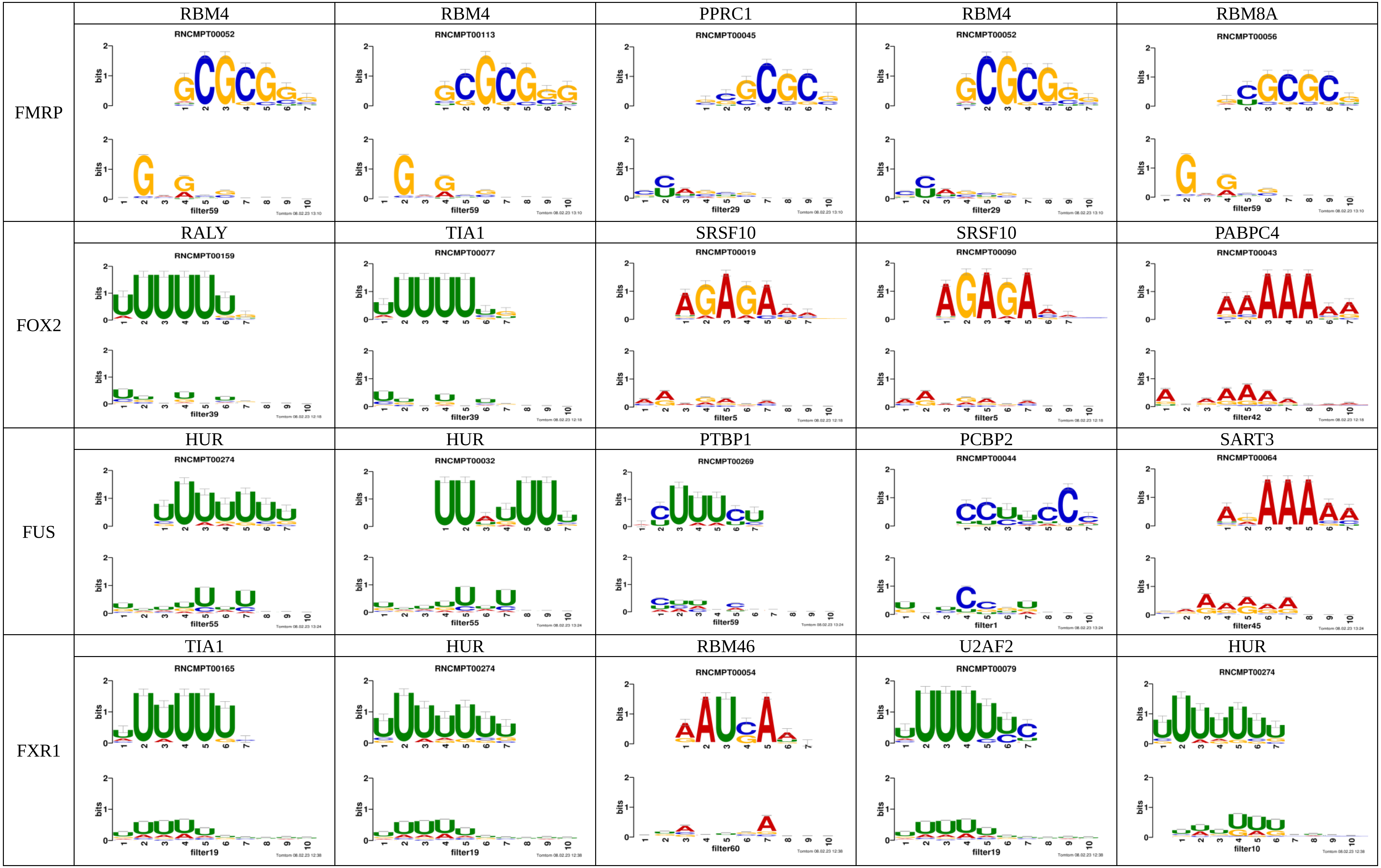
FMRP
RBM4
RNCMPT00052
filter59
RBM4
RNCMPT00113
filter59
PPRC1
RNCMPT00045
filter29
RBM4
RNCMPT00052
filter29
RBM8A
RNCMPT00056
filter59
FOX2
RALY
RNCMPT00159
filter39
TIA1
RNCMPT00077
filter39
SRSF10
RNCMPT00019
filter5
SRSF10
RNCMPT00090
filter5
PABPC4
RNCMPT00043
filter42
FUS
HUR
RNCMPT00274
filter55
HUR
RNCMPT00032
filter55
PTBP1
RNCMPT00269
filter59
PCBP2
RNCMPT00044
filter1
SART3
RNCMPT00064
filter45
FXR1
TIA1
RNCMPT00165
filter19
HUR
RNCMPT00274
filter19
RBM46
RNCMPT00054
filter60
U2AF2
RNCMPT00079
filter19
HUR
RNCMPT00274
filter10

| FXR2 | RBM4 | RBM4 | HUR | U2AF2 | RBM45 |
|---|---|---|---|---|---|
| | 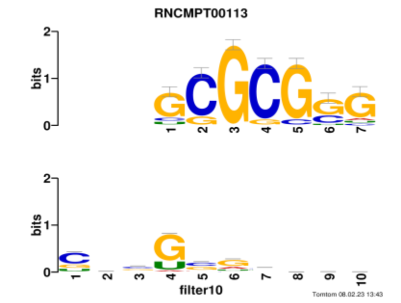 | 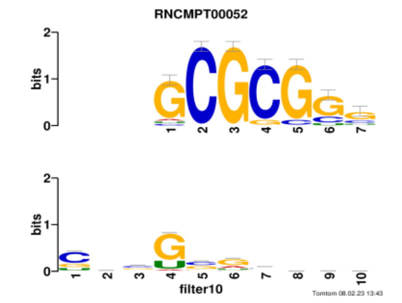 | 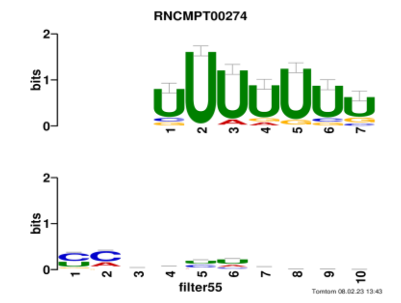 | 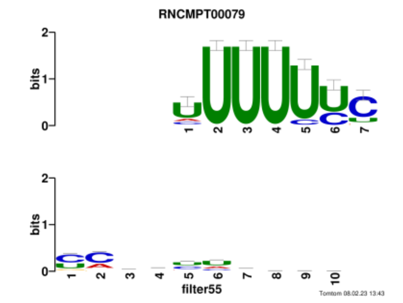 | 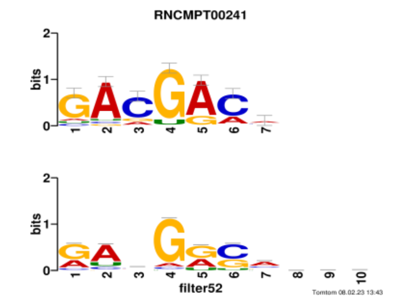 |
| HNRN<br>PC | HUR | ZC3H14 | CPEB4 | HUR | HUR |
| | 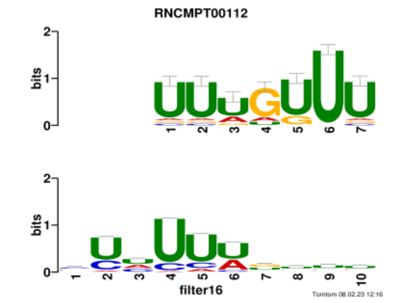 | 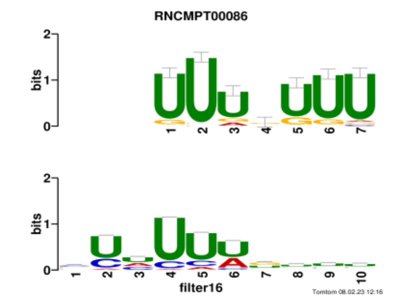 | 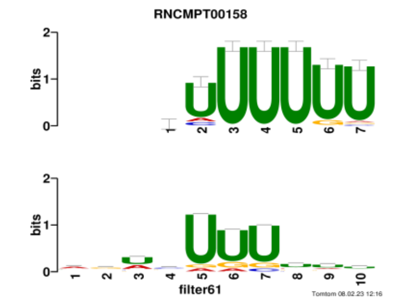 | 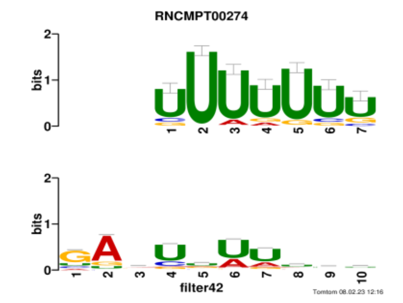 | 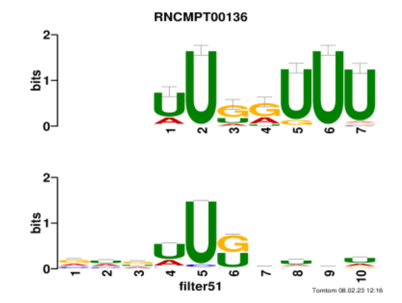 |
| HUR | HUR | PPRC1 | CPEB2 | HNRNPCL1 | ZC3H14 |
| | 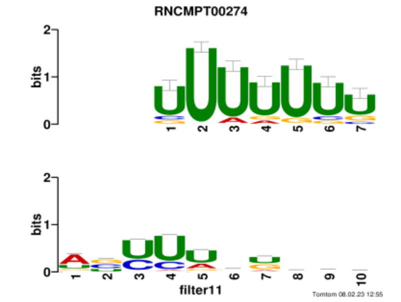 | 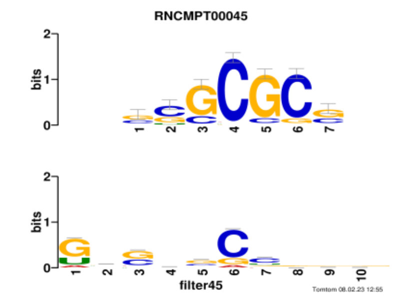 | 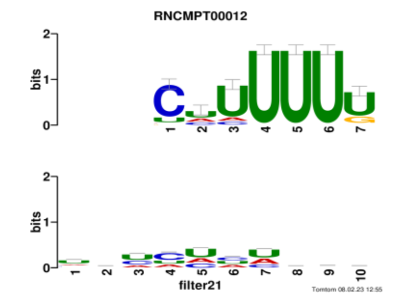 | 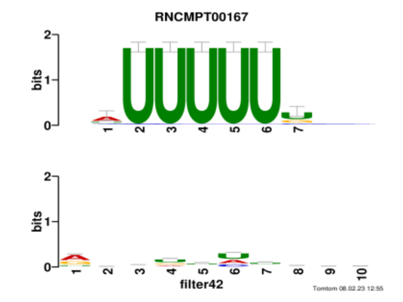 | 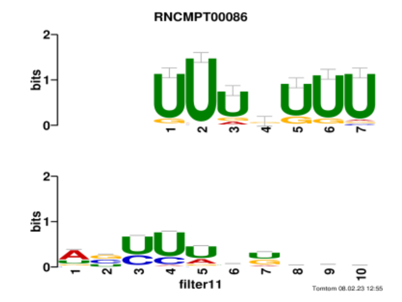 |
| IGF2B<br>P1 | IGF2BP3 | HNRNPK | PCBP1 | RBM45 | IGF2BP3 |
| | 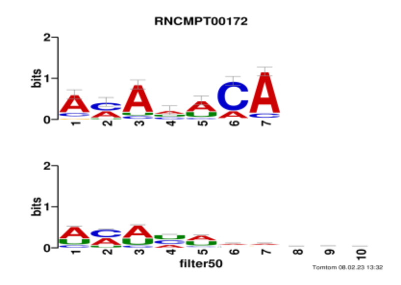 | 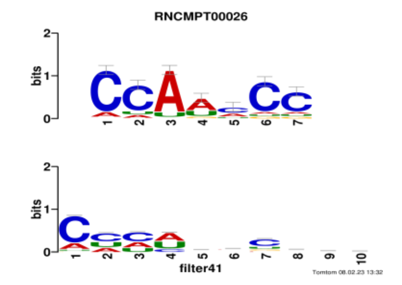 | 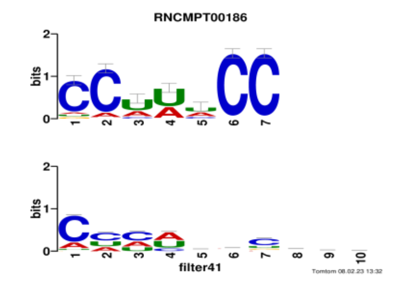 | 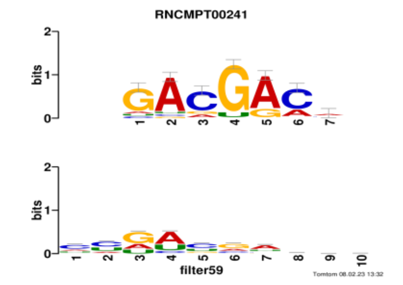 | 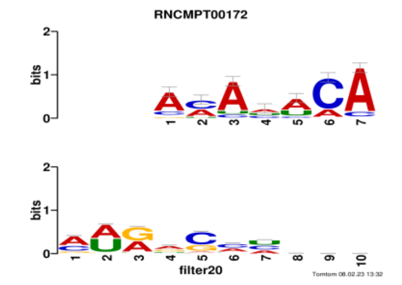 |

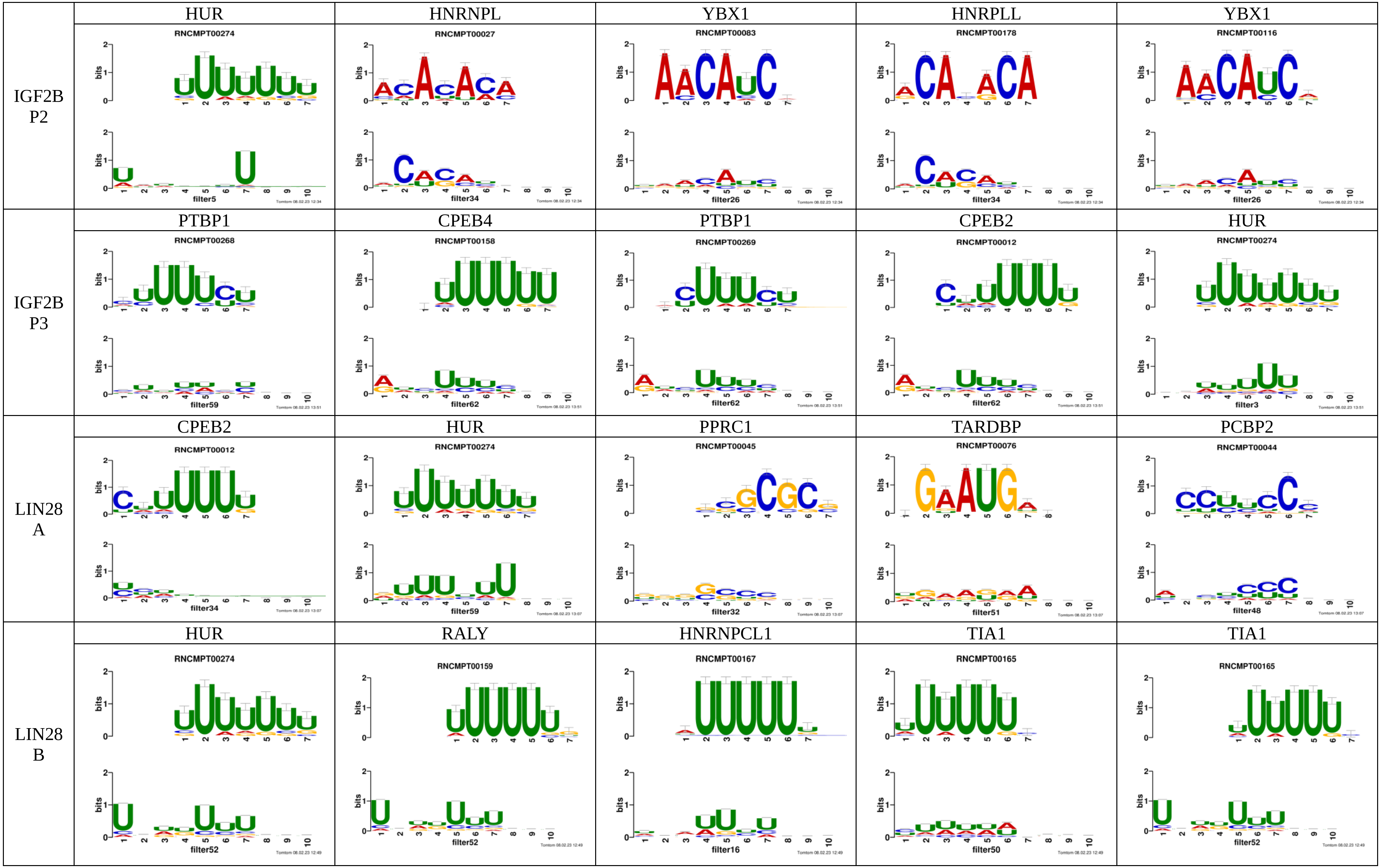
IGF2BP2
HUR
RNCMPT00274
filter5
HNRNPL
RNCMPT00027
filter34
YBX1
RNCMPT00083
filter26
HNRPLL
RNCMPT00178
filter34
YBX1
RNCMPT00116
filter26
IGF2BP3
PTBP1
RNCMPT00268
filter59
CPEB4
RNCMPT00158
filter62
PTBP1
RNCMPT00269
filter62
CPEB2
RNCMPT00012
filter62
HUR
RNCMPT00274
filter3
LIN28A
CPEB2
RNCMPT00012
filter34
HUR
RNCMPT00274
filter59
PPRC1
RNCMPT00045
filter32
TARDBP
RNCMPT00076
filter51
PCBP2
RNCMPT00044
filter48
LIN28B
HUR
RNCMPT00274
filter52
RALY
RNCMPT00159
filter52
HNRNPCL1
RNCMPT00167
filter16
TIA1
RNCMPT00165
filter50
TIA1
RNCMPT00165
filter52

**METTL3**

PCBP1

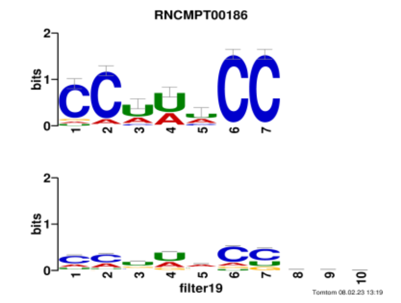


RBMS3

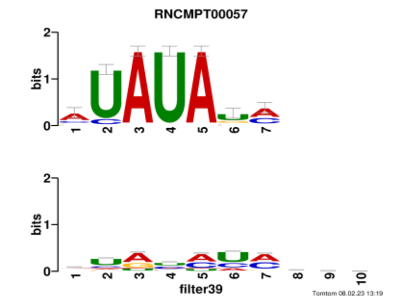


PCBP2

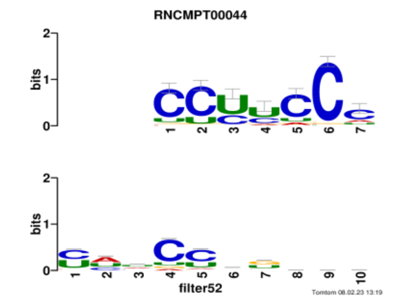


PCBP2

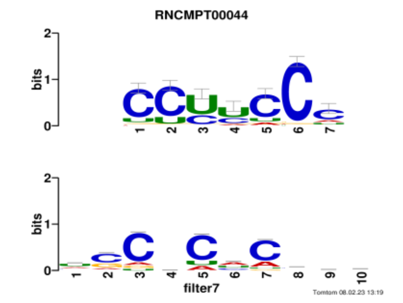


PPRC1

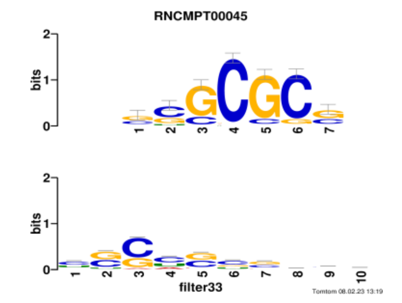


**MOV10**

HUR

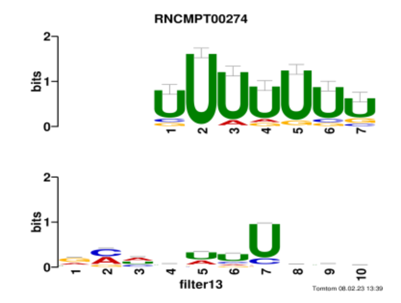


CPEB2

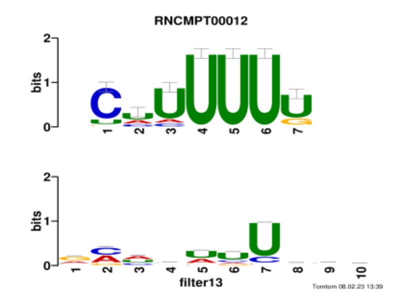


SRSF10

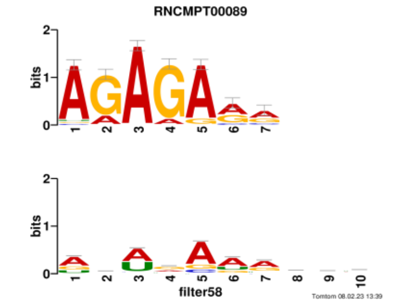


HNRNPCL1

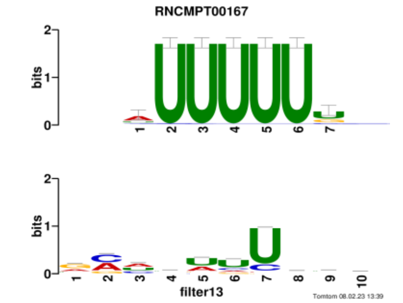


PTBP1

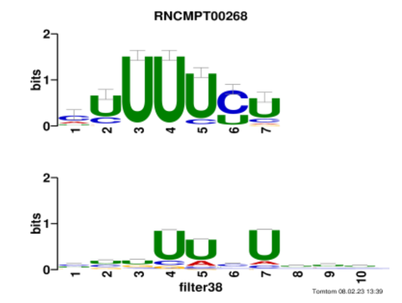


**PTB**

HUR

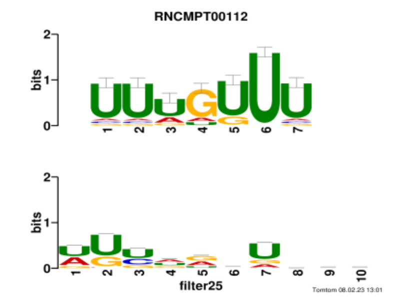


ZC3H14

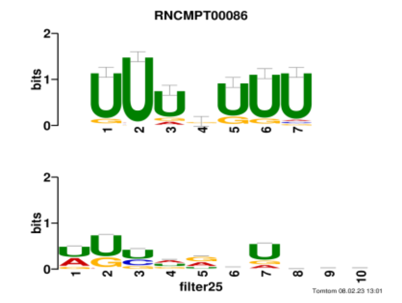


HUR

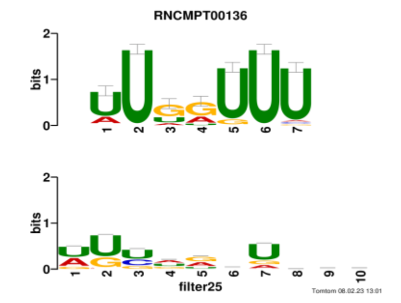


CPEB2

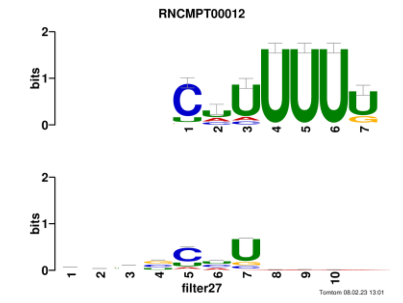


HUR

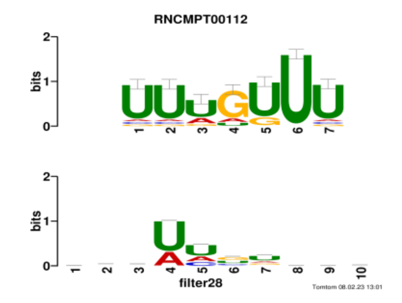


**PUM2**

HUR

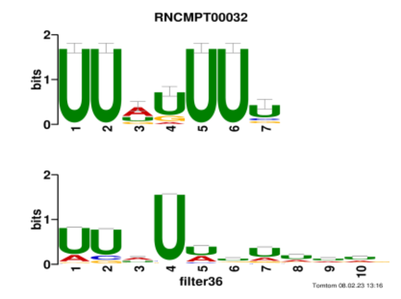


RALY

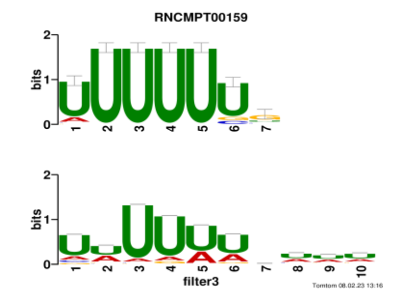


HUR

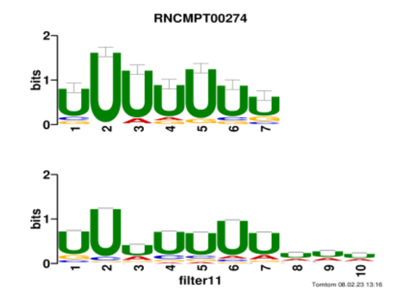


TIA1

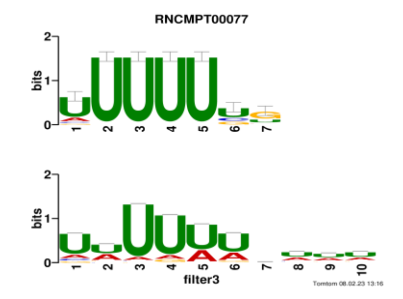


HNRNPCL1

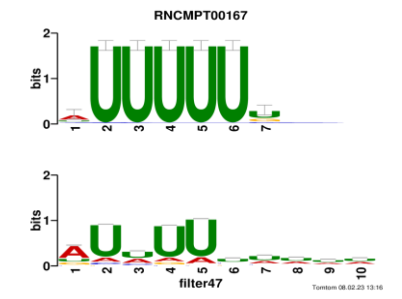

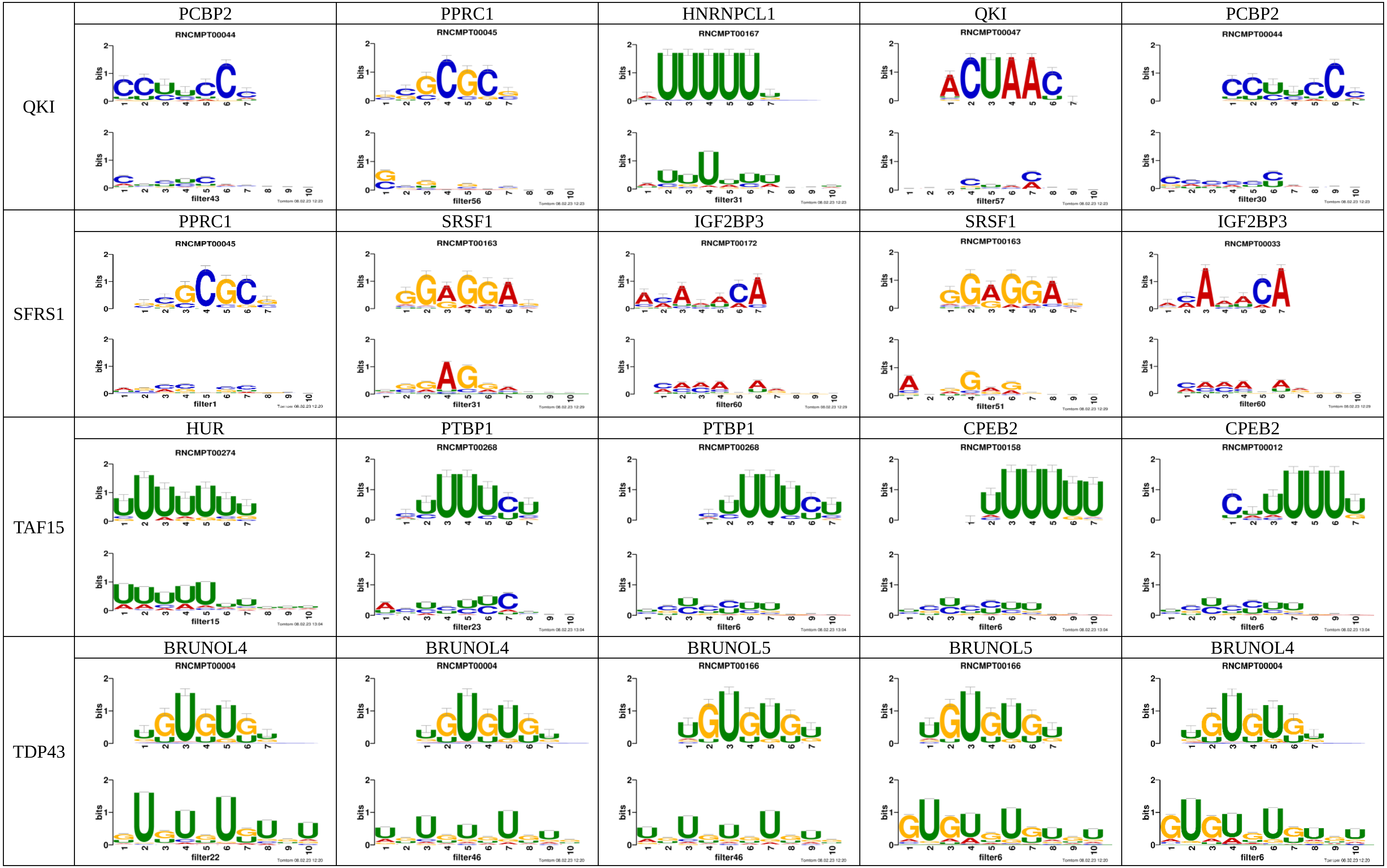
QKI
PCBP2
RNCMPT00044
filter43
PPRC1
RNCMPT00045
filter56
HNRNPCL1
RNCMPT00167
filter31
QKI
RNCMPT00047
filter57
PCBP2
RNCMPT00044
filter30
SFRS1
PPRC1
RNCMPT00045
filter1
SRSF1
RNCMPT00163
filter31
IGF2BP3
RNCMPT00172
filter60
SRSF1
RNCMPT00163
filter51
IGF2BP3
RNCMPT00033
filter60
TAF15
HUR
RNCMPT00274
filter15
PTBP1
RNCMPT00268
filter23
PTBP1
RNCMPT00268
filter6
CPEB2
RNCMPT00158
filter6
CPEB2
RNCMPT00012
filter6
TDP43
BRUNOL4
RNCMPT00004
filter22
BRUNOL4
RNCMPT00004
filter46
BRUNOL5
RNCMPT00166
filter46
BRUNOL5
RNCMPT00166
filter6
BRUNOL4
RNCMPT00004
filter6

| | | | | | |
|---|---|---|---|---|---|
| TIA1 | CPEB4 | HUR | HUR | HUR | HUR |
| | 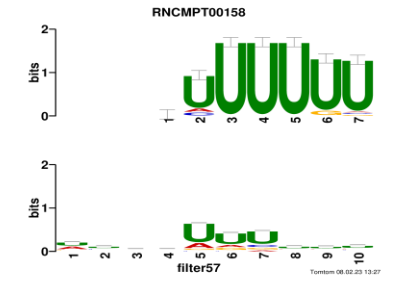  | 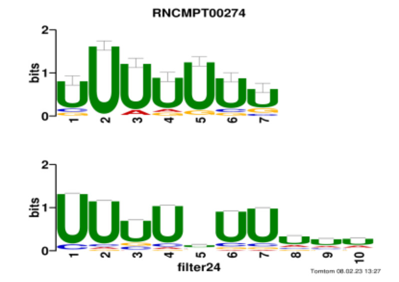  | 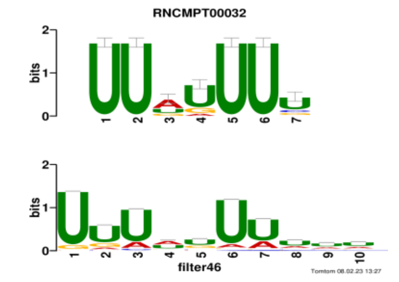  | 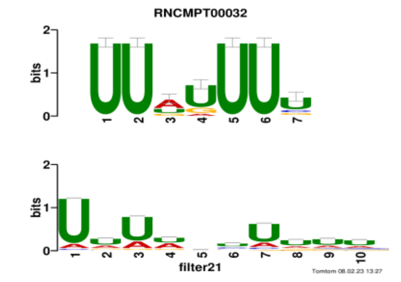  | 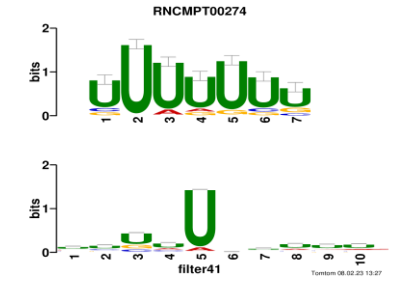  |
| TIAL1 | HUR | PABPC4 | CPEB4 | HUR | YBX1 |
| | 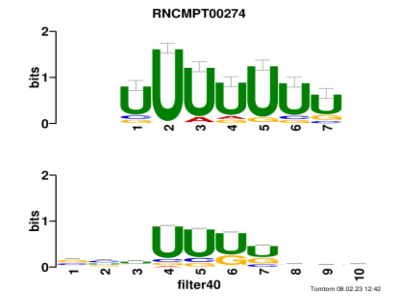  | 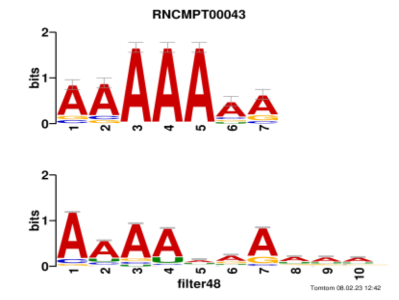  | 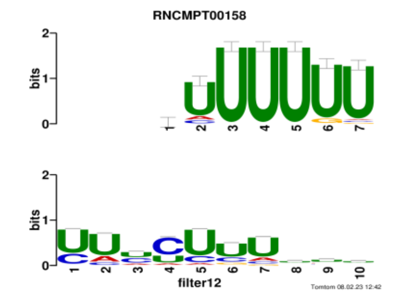  | 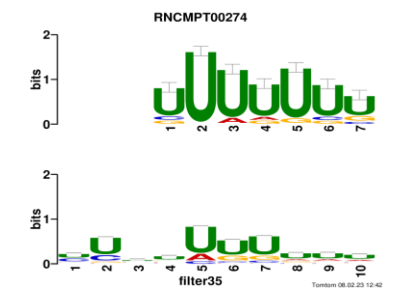  | 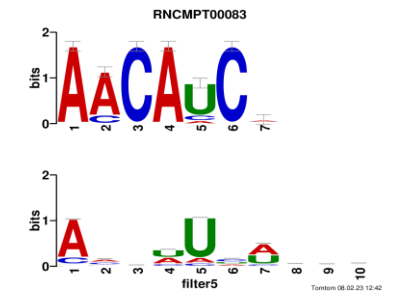  |
| TNRC6 | PCBP2 | PCBP2 | HUR | ZC3H14 | HUR |
| | 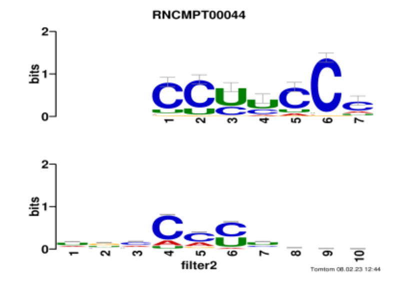  | 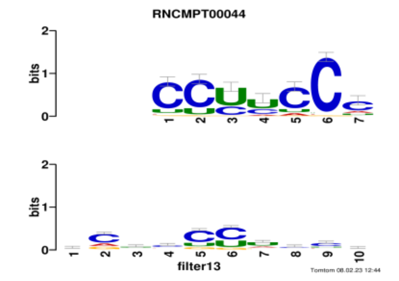  | 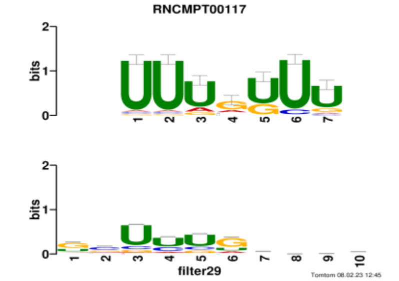  | 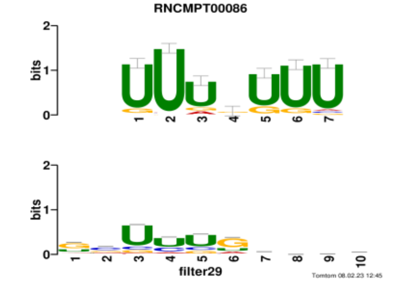  | 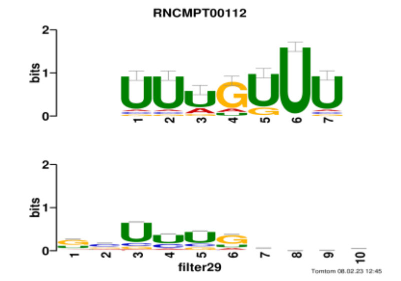  |
| U2AF65 | U2AF2 | HUR | HUR | ZC3H14 | HUR |
| | 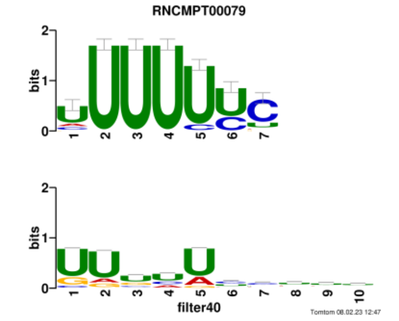  | 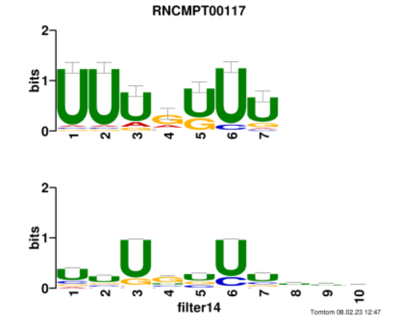  | 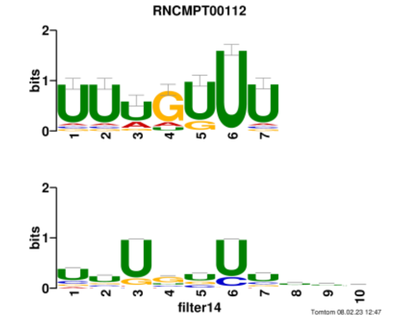  | 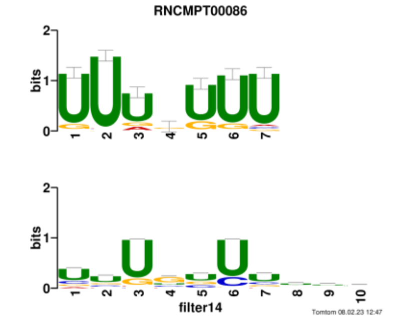  | 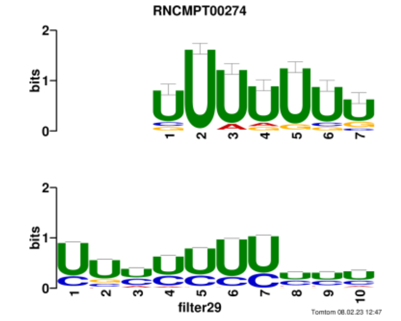  |

| | | | | | |
|---|---|---|---|---|---|
| WTAP | SRSF1 | PCBP2 | SRSF2 | SRSF10 | SRSF10 |
| | 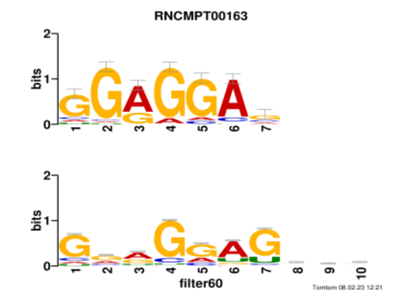  | 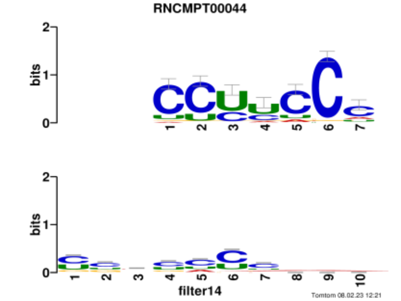  | 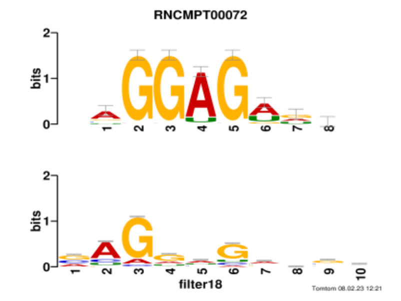  | 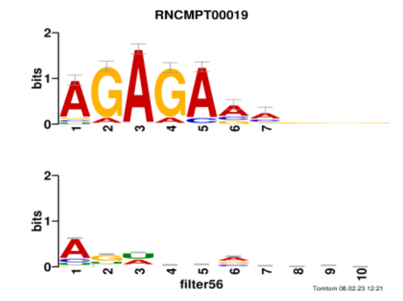  | 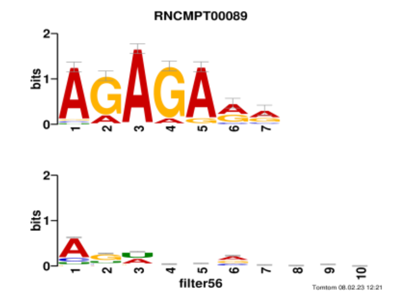  |
| ZC3H7B | HUR | CPEB4 | CPEB2 | CPEB2 | HUR |
| | 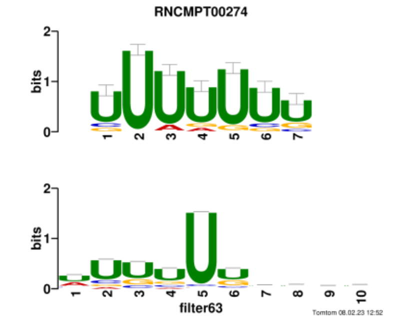  | 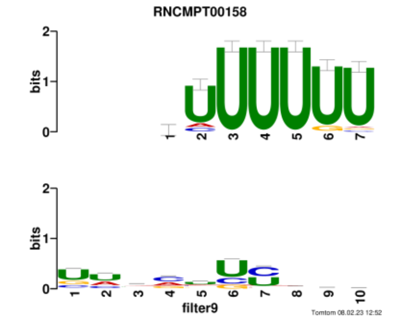  | 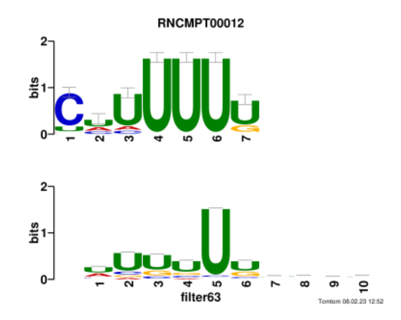  | 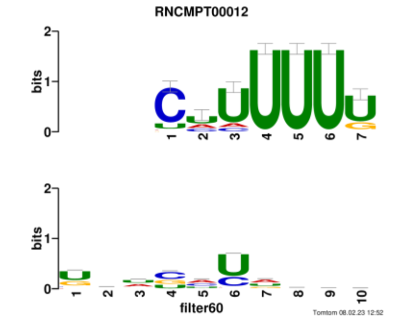  | 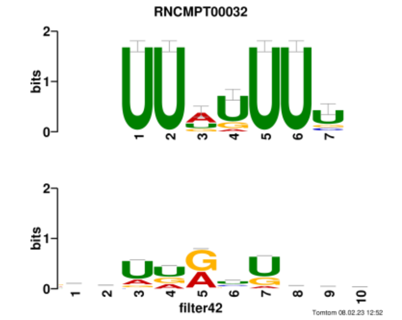  |

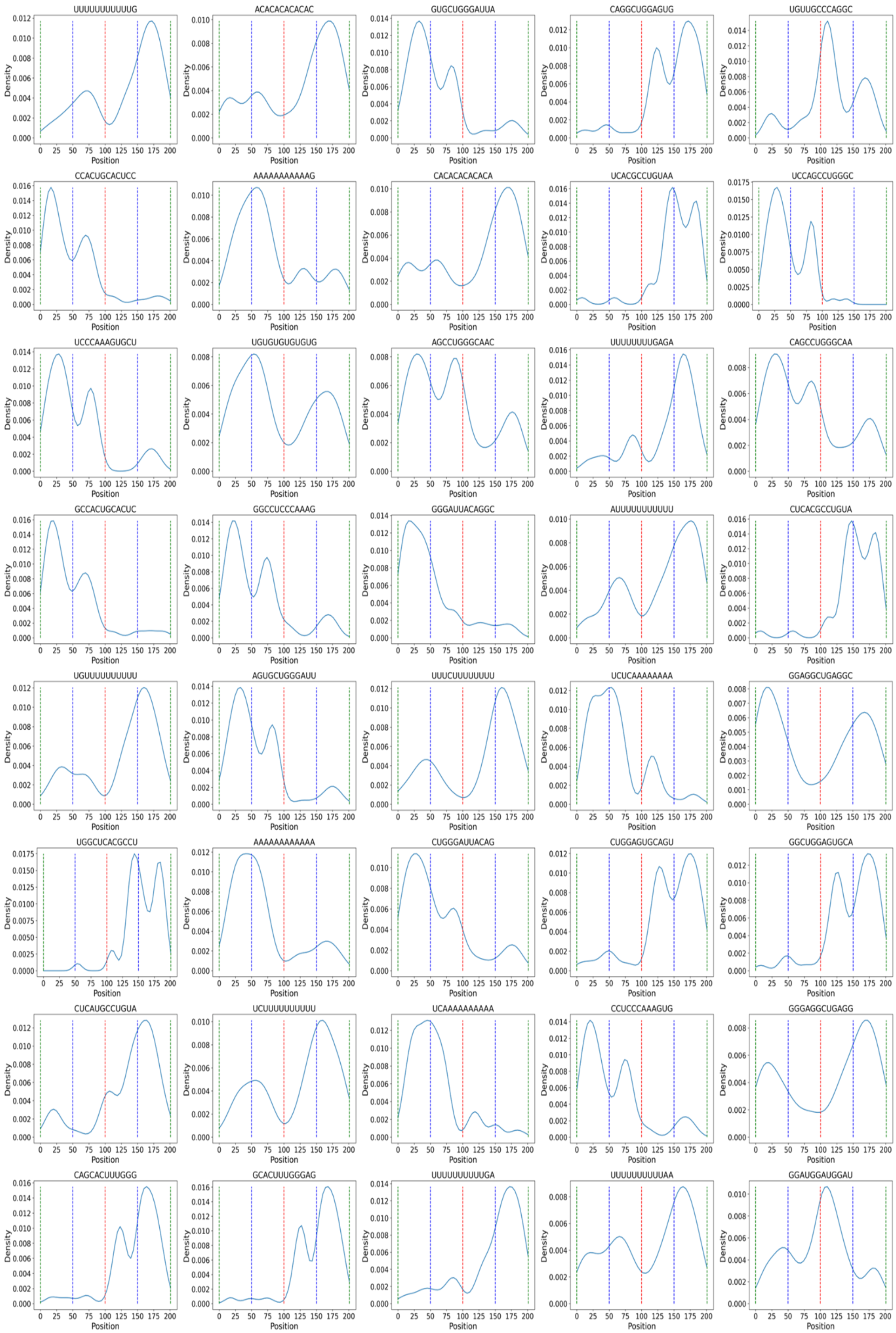

UUUUUUUUUUUG
ACACACACACAC
GUGCUGGGAUUA
CAGGCUGGAGUG
UGUUGCCCAGGC
CCACUGCACUCC
AAAAAAAAAAAG
CACACACACACA
UCACGCCUGUAA
UCCAGCCUGGGC
UCCCAAAGUGCU
UGUGUGUGUGUG
AGCCUGGGCAAC
UUUUUUUUGAGA
CAGCCUGGGCAA
GCCACUGCACUC
GGCCUCCCAAAG
GGGAUUACAGGC
AUUUUUUUUUUU
CUCACGCCUGUA
UGUUUUUUUUUU
AGUGCUGGGAUU
UUUCUUUUUUUU
UCUCAAAAAAAA
GGAGGCUGAGGC
UGGCUCACGCCU
AAAAAAAAAAAA
CUGGGAUUACAG
CUGGAGUGCAGU
GGCUGGAGUGCA
CUCAUGCCUGUA
UCUUUUUUUUUU
UCAAAAAAAAAA
CCUCCCAAAGUG
GGGAGGCUGAGG
CAGCACUUUGGG
GCACUUUGGGAG
UUUUUUUUUUGA
UUUUUUUUUUAA
GGAUGGAUGGAU
Density
Position

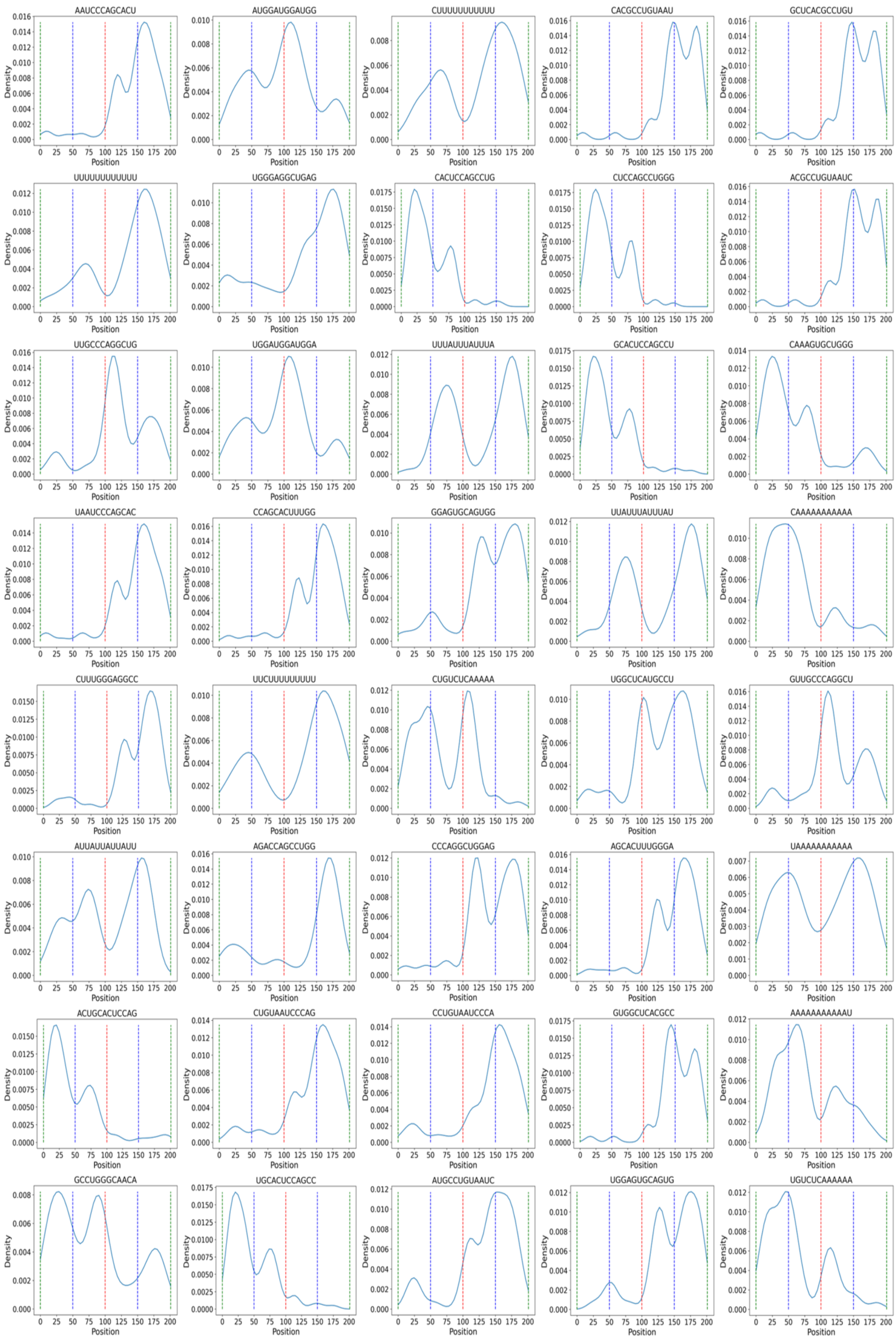
AAUCCCAGCACU
AUGGAUGGAUGG
CUUUUUUUUUUU
CACGCCUGUAAU
GCUCACGCCUGU
UUUUUUUUUUUU
UGGGAGGCUGAG
CACUCCAGCCUG
CUCCAGCCUGGG
ACGCCUGUAAUC
UUGCCCAGGCUG
UGGAUGGAUGGA
UUUAUUUAUUUA
GCACUCCAGCCU
CAAAGUGCUGGG
UAAUCCCAGCAC
CCAGCACUUUGG
GGAGUGCAGUGG
UUAUUUAUUUAU
CAAAAAAAAAAA
CUUUGGGAGGCC
UUCUUUUUUUUU
CUGUCUCAAAAA
UGGCUCAUGCCU
GUUGCCCAGGCU
AUUAUUAUUAUU
AGACCAGCCUGG
CCCAGGCUGGAG
AGCACUUUGGGA
UAAAAAAAAAAA
ACUGCACUCCAG
CUGUAAUCCCAG
CCUGUAAUCCCA
GUGGCUCACGCC
AAAAAAAAAAAU
GCCUGGGCAACA
UGCACUCCAGCC
AUGCCUGUAAUC
UGGAGUGCAGUG
UGUCUCAAAAAA
Density
Position

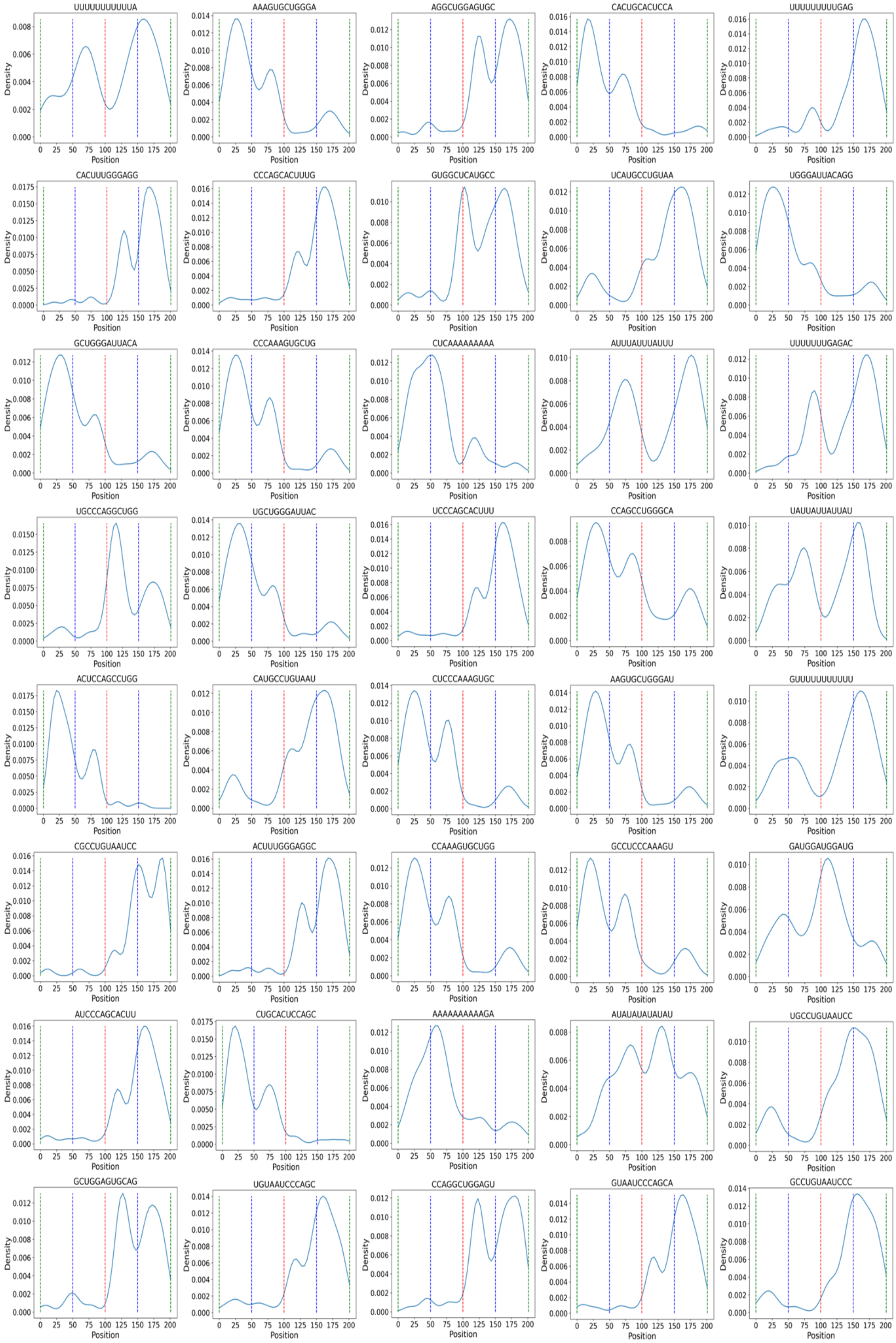
UUUUUUUUUUUA
AAAGUGCUGGGA
AGGCUGGAGUGC
CACUGCACUCCA
UUUUUUUUUGAG
CACUUUGGGAGG
CCCAGCACUUUG
GUGGCUCAUGCC
UCAUGCCUGUAA
UGGGAUUACAGG
GCUGGGAUUACA
CCCAAAGUGCUG
CUCAAAAAAAAA
AUUUAUUUAUUU
UUUUUUUGAGAC
UGCCCAGGCUGG
UGCUGGGAUUAC
UCCCAGCACUUU
CCAGCCUGGGCA
UAUUAUUAUUAU
ACUCCAGCCUGG
CAUGCCUGUAAU
CUCCCAAAGUGC
AAGUGCUGGGAU
GUUUUUUUUUUU
CGCCUGUAAUCC
ACUUUGGGAGGC
CCAAAGUGCUGG
GCCUCCCAAAGU
GAUGGAUGGAUG
AUCCCAGCACUU
CUGCACUCCAGC
AAAAAAAAAAGA
AUAUAUAUAUAU
UGCCUGUAAUCC
GCUGGAGUGCAG
UGUAAUCCCAGC
CCAGGCUGGAGU
GUAAUCCCAGCA
GCCUGUAAUCCC
Density
Position

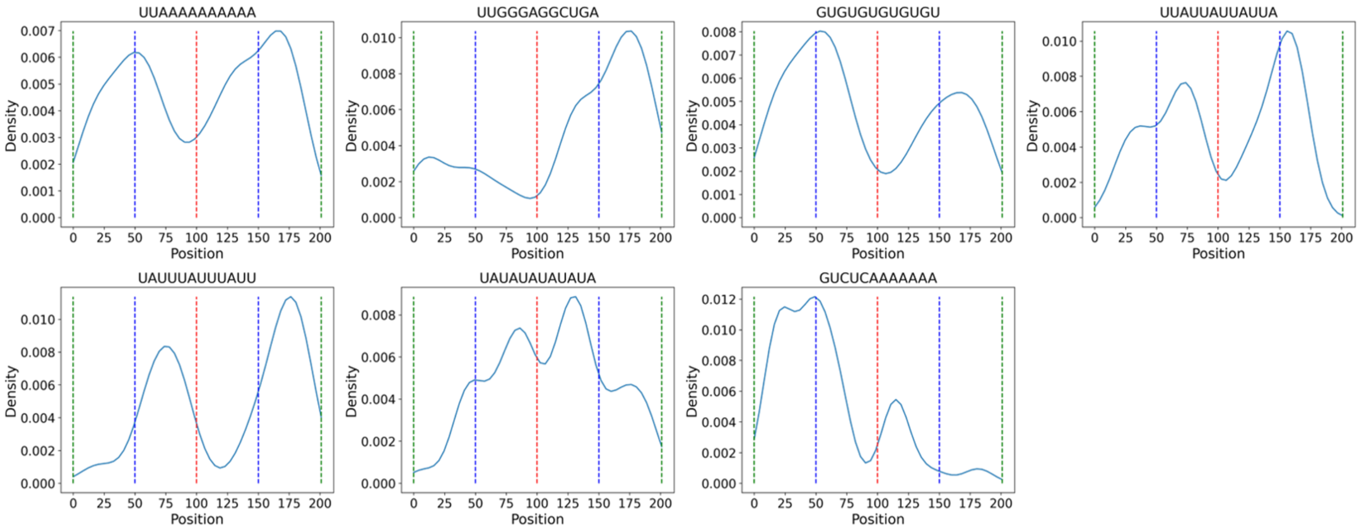


**Figure S3.** 127 motif distribution found by CPBFCN in EIF4A3 of length 201.

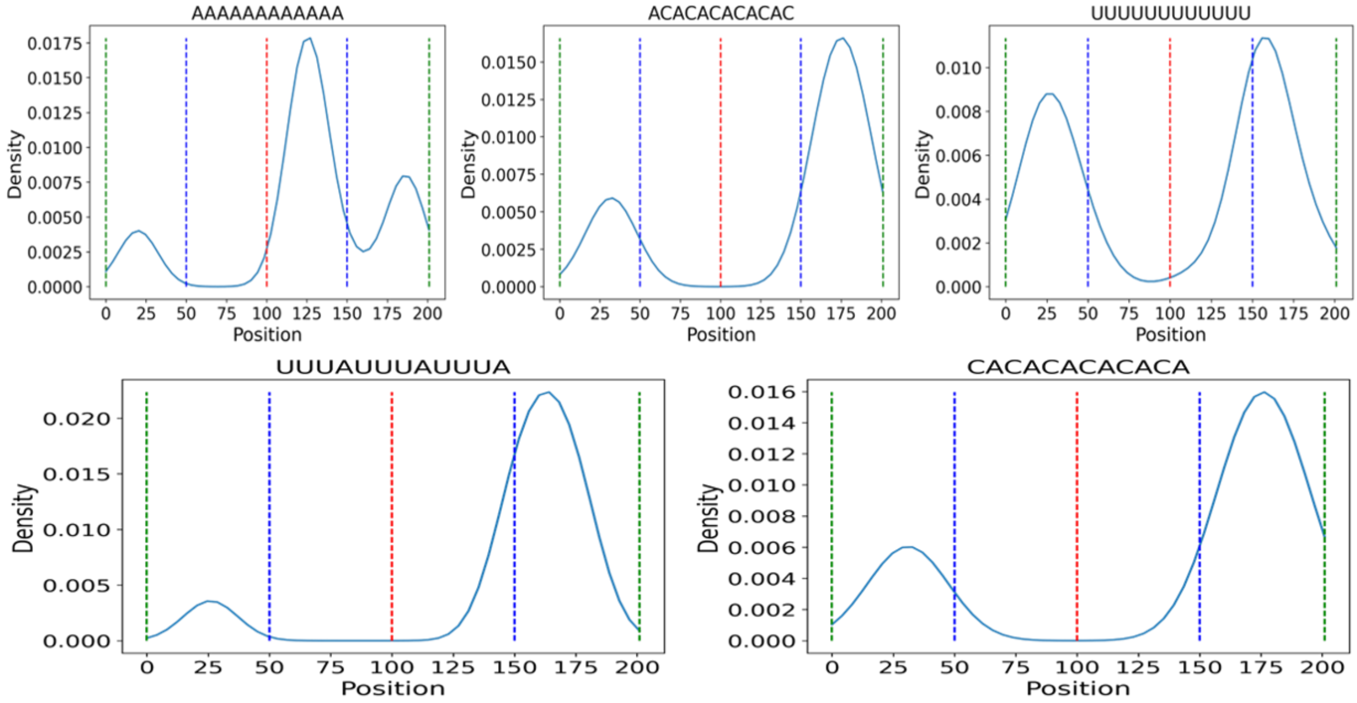


**Figure S4.** 5 motif distribution found by CPBFCN in FOX2 of length 201.

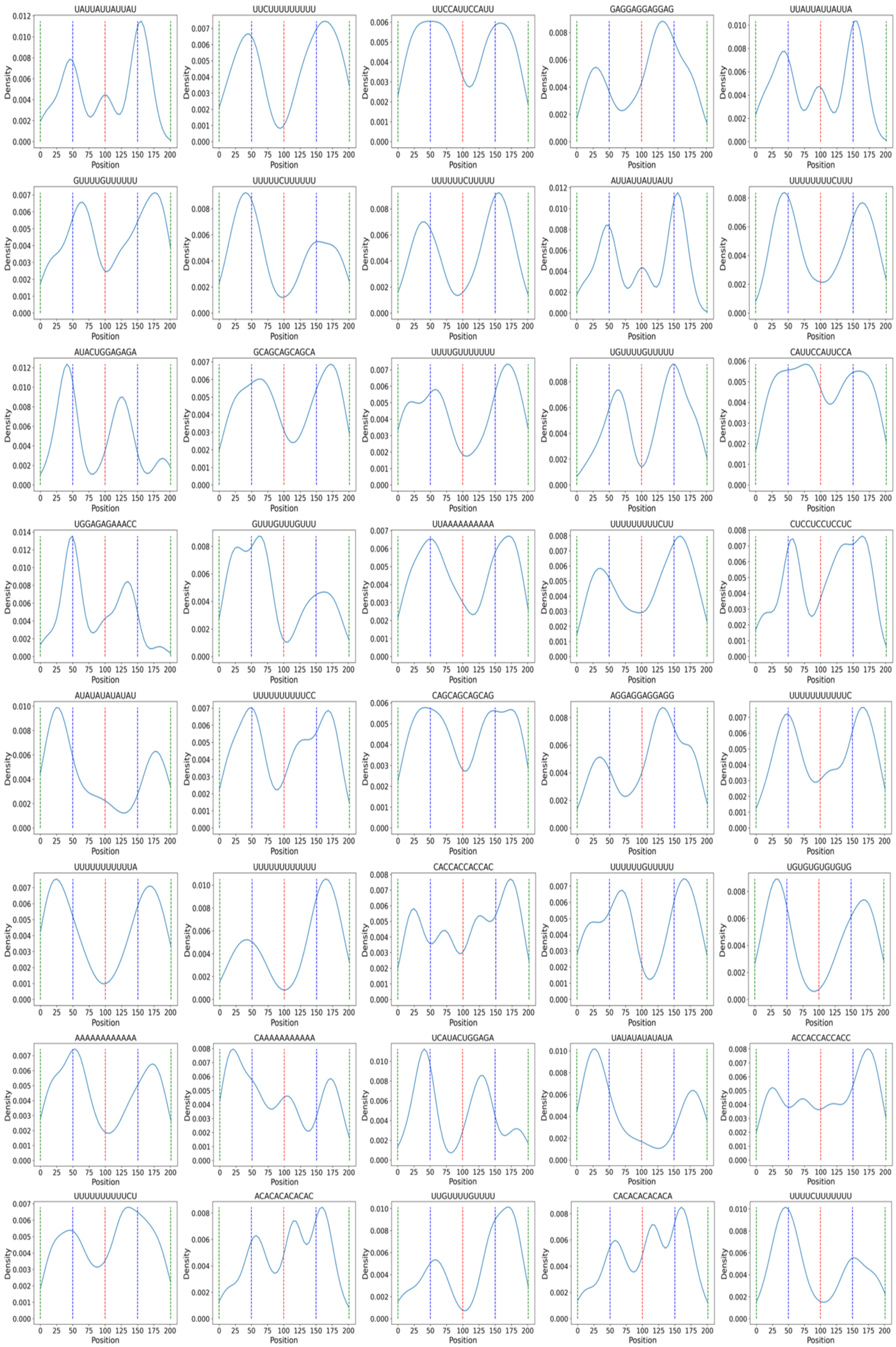
UAUUAUUAUUAU
UUCUUUUUUUUU
UUCCAUUCCAUU
GAGGAGGAGGAG
UUAUUAUUAUUA
GUUUUGUUUUUU
UUUUUCUUUUUU
UUUUUUCUUUUU
AUUAUUAUUAUU
UUUUUUUUCUUU
AUACUGGAGAGA
GCAGCAGCAGCA
UUUUGUUUUUUU
UGUUUUGUUUUU
CAUUCCAUUCCA
UGGAGAGAAACC
GUUUGUUUGUUU
UUAAAAAAAAAA
UUUUUUUUUCUU
CUCCUCCUCCUC
AUAUAUAUAUAU
UUUUUUUUUUCC
CAGCAGCAGCAG
AGGAGGAGGAGG
UUUUUUUUUUUC
UUUUUUUUUUUA
UUUUUUUUUUUU
CACCACCACCAC
UUUUUUGUUUUU
UGUGUGUGUGUG
AAAAAAAAAAAA
CAAAAAAAAAAA
UCAUACUGGAGA
UAUAUAUAUAUA
ACCACCACCACC
UUUUUUUUUUCU
ACACACACACAC
UUGUUUUGUUUU
CACACACACACA
UUUCUUUUUUUU
Density
Position

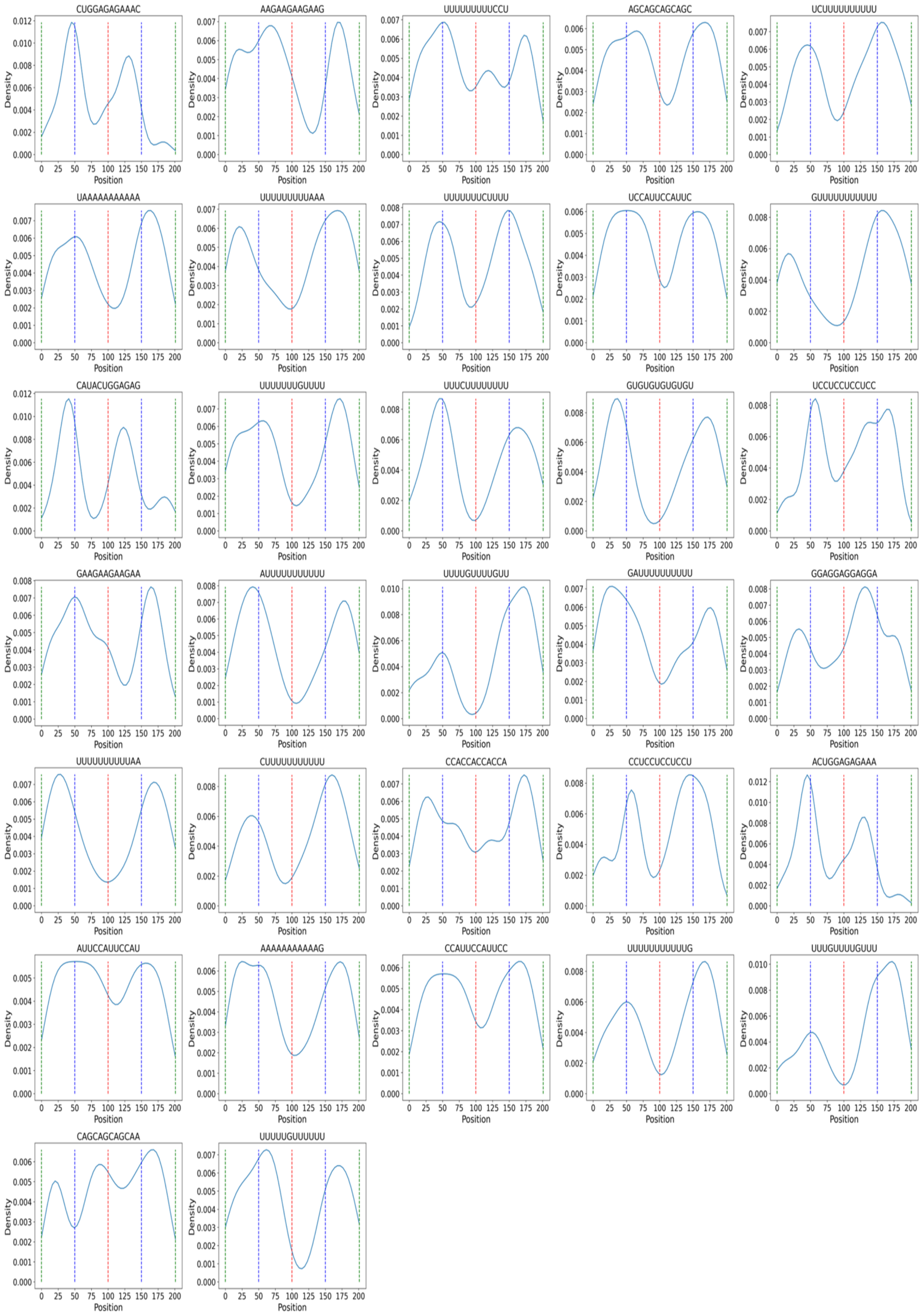


**Figure S5.** 72 motif distribution found by CPBFCN in IGF2BP1 of length 201.

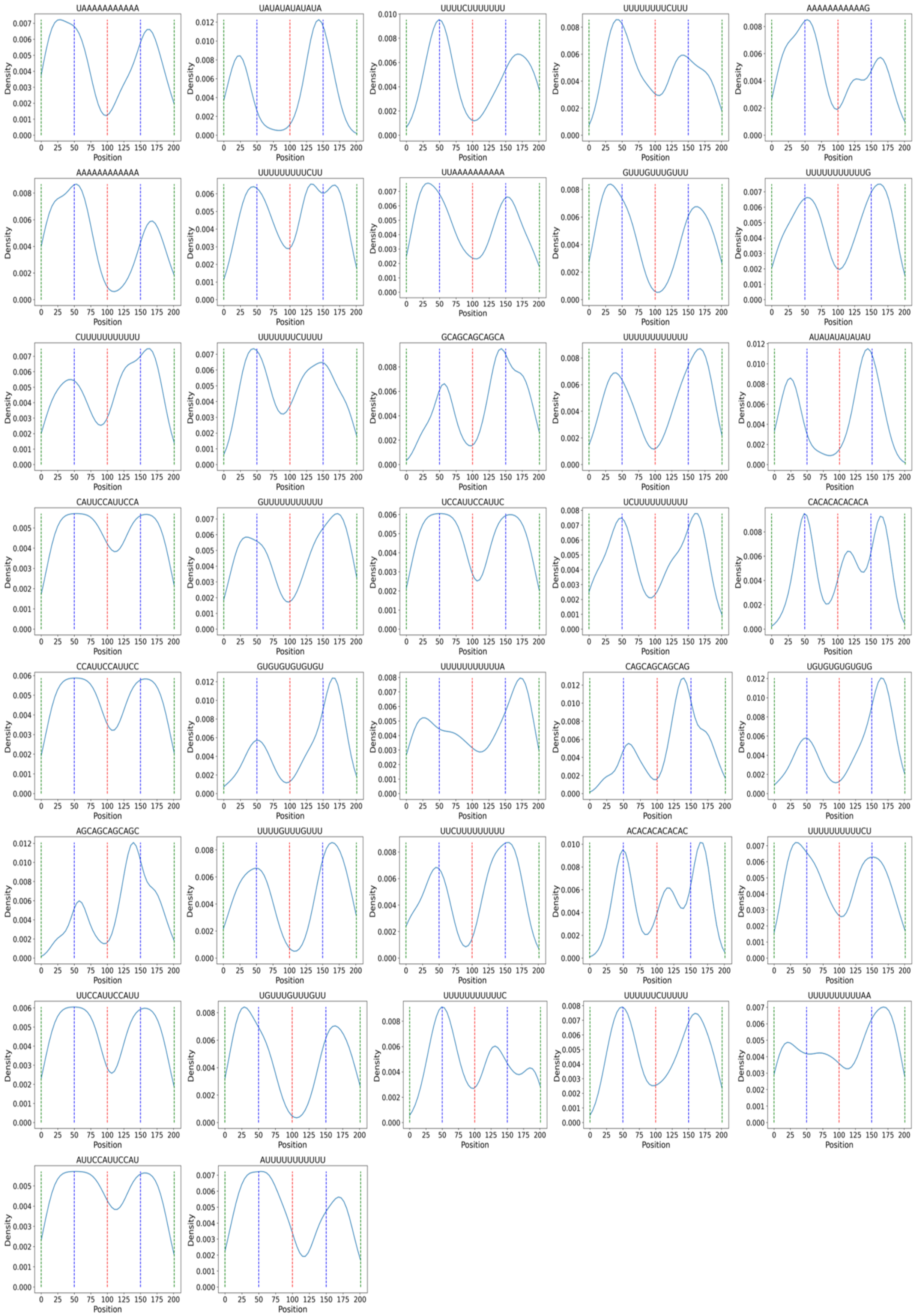


**Figure S6.** 37 motif distribution found by CPBFCN in IGF2BP2 of length 201.

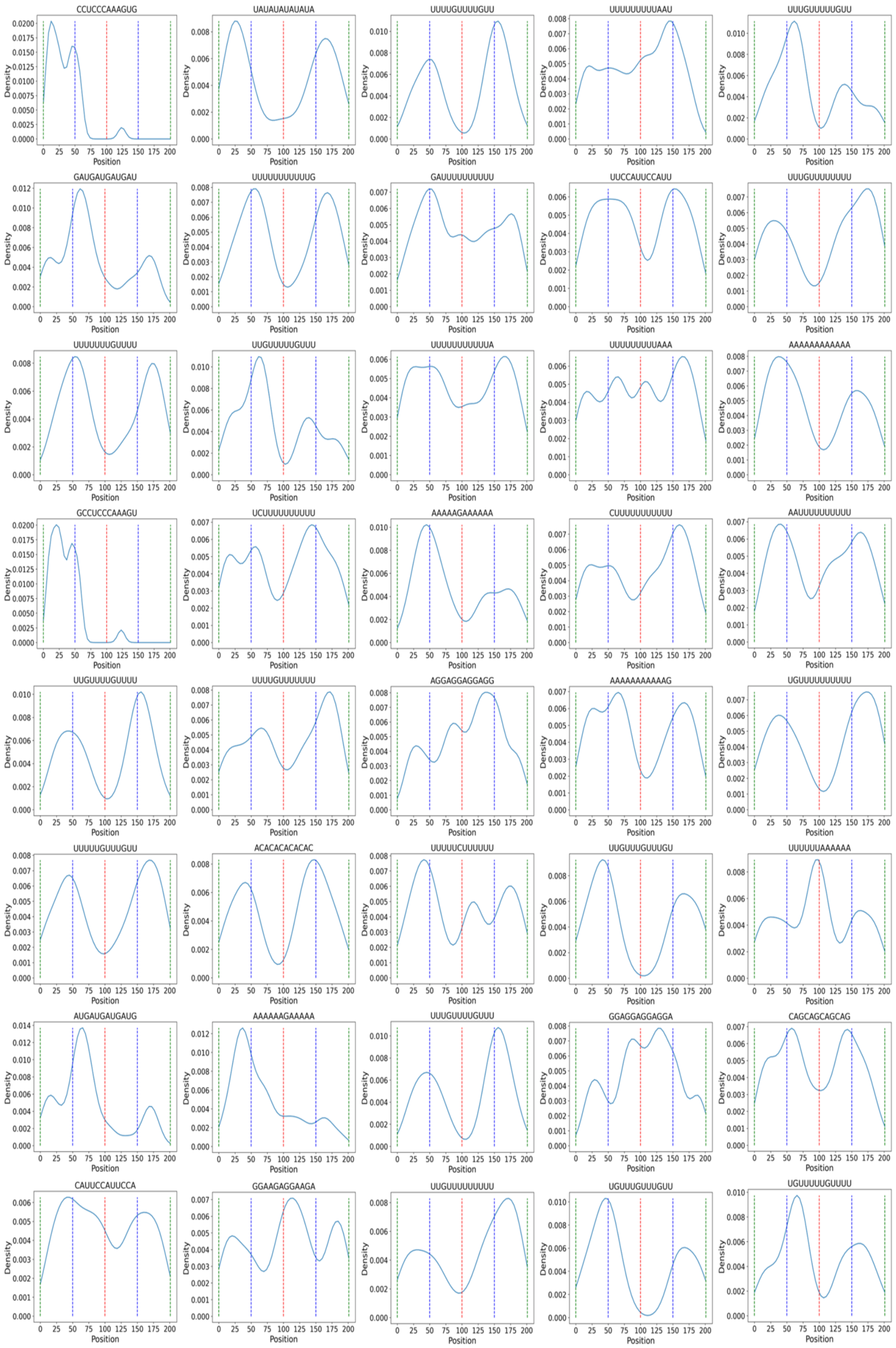
CCUCCCAAAGUG
UAUAUAUAUAUA
UUUUGUUUUGUU
UUUUUUUUUAAU
UUUGUUUUUGUU
GAUGAUGAUGAU
UUUUUUUUUUUG
GAUUUUUUUUUU
UUCCAUUCCAUU
UUUGUUUUUUUU
UUUUUUUGUUUU
UUGUUUUUGUUU
UUUUUUUUUUUA
UUUUUUUUUAAA
AAAAAAAAAAAA
GCCUCCCAAAGU
UCUUUUUUUUUU
AAAAAGAAAAAA
CUUUUUUUUUUU
AAUUUUUUUUUU
UUGUUUUGUUUU
UUUUGUUUUUUU
AGGAGGAGGAGG
AAAAAAAAAAAG
UGUUUUUUUUUU
UUUUUGUUUGUU
ACACACACACAC
UUUUUCUUUUUU
UUGUUUGUUUGU
UUUUUUAAAAAA
AUGAUGAUGAUG
AAAAAAGAAAAA
UUUGUUUUGUUU
GGAGGAGGAGGA
CAGCAGCAGCAG
CAUUCCAUUCCA
GGAAGAGGAAGA
UUGUUUUUUUUU
UGUUUGUUUGUU
UGUUUUUGUUUU
Density
Position

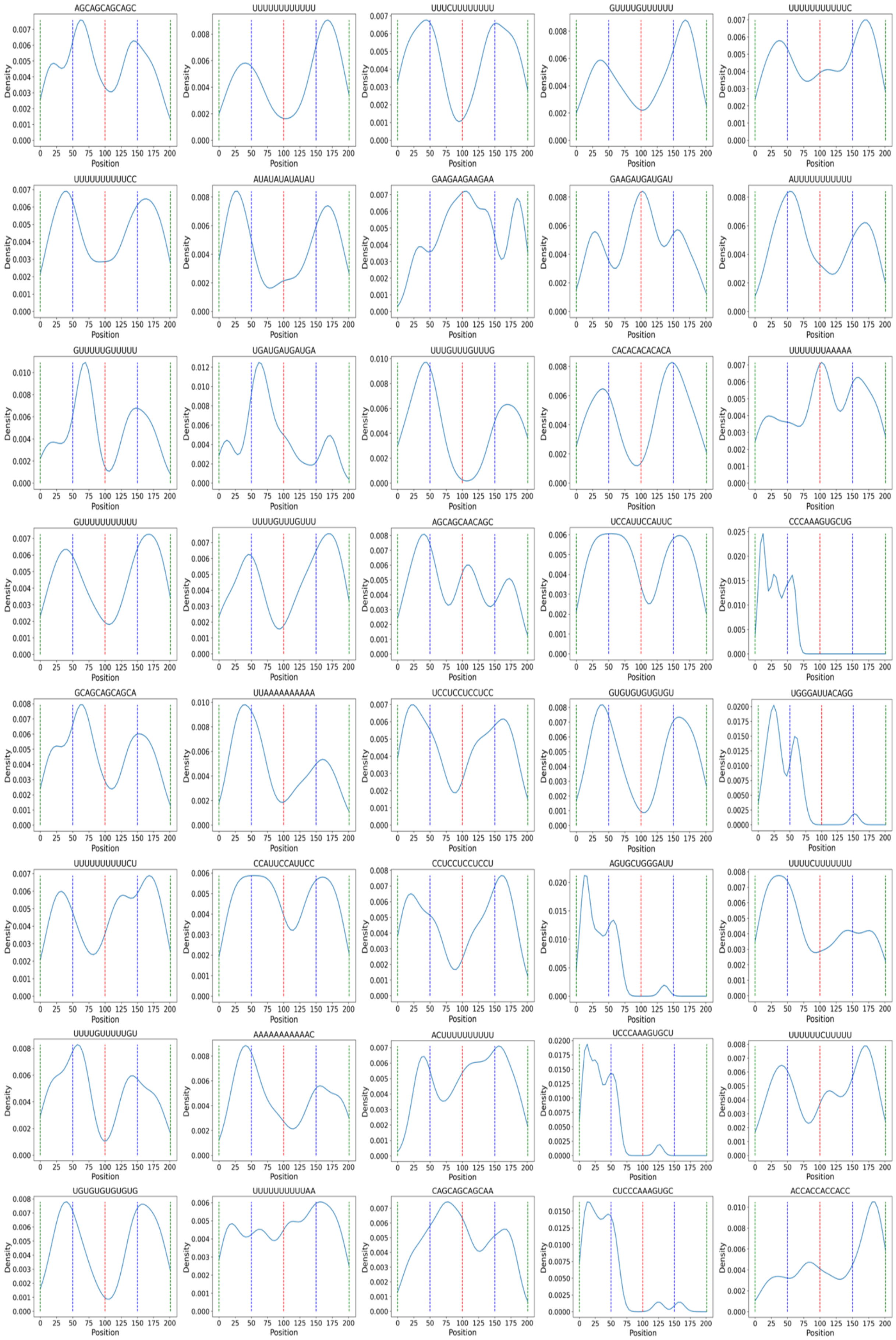
AGCAGCAGCAGC
UUUUUUUUUUUU
UUUCUUUUUUUU
GUUUUGUUUUUU
UUUUUUUUUUUC
UUUUUUUUUUCC
AUAUAUAUAUAU
GAAGAAGAAGAA
GAAGAUGAUGAU
AUUUUUUUUUUU
GUUUUUGUUUUU
UGAUGAUGAUGA
UUUGUUUGUUUG
CACACACACACA
UUUUUUUAAAAA
GUUUUUUUUUUU
UUUUGUUUGUUU
AGCAGCAACAGC
UCCAUUCCAUUC
CCCAAAGUGCUG
GCAGCAGCAGCA
UUAAAAAAAAAA
UCCUCCUCCUCC
GUGUGUGUGUGU
UGGGAUUACAGG
UUUUUUUUUUCU
CCAUUCCAUUCC
CCUCCUCCUCCU
AGUGCUGGGAUU
UUUUCUUUUUUU
UUUUGUUUUUGU
AAAAAAAAAAAC
ACUUUUUUUUUU
UCCCAAAGUGCU
UUUUUUCUUUUU
UGUGUGUGUGUG
UUUUUUUUUUAA
CAGCAGCAGCAA
CUCCCAAAGUGC
ACCACCACCACC
Density
Position

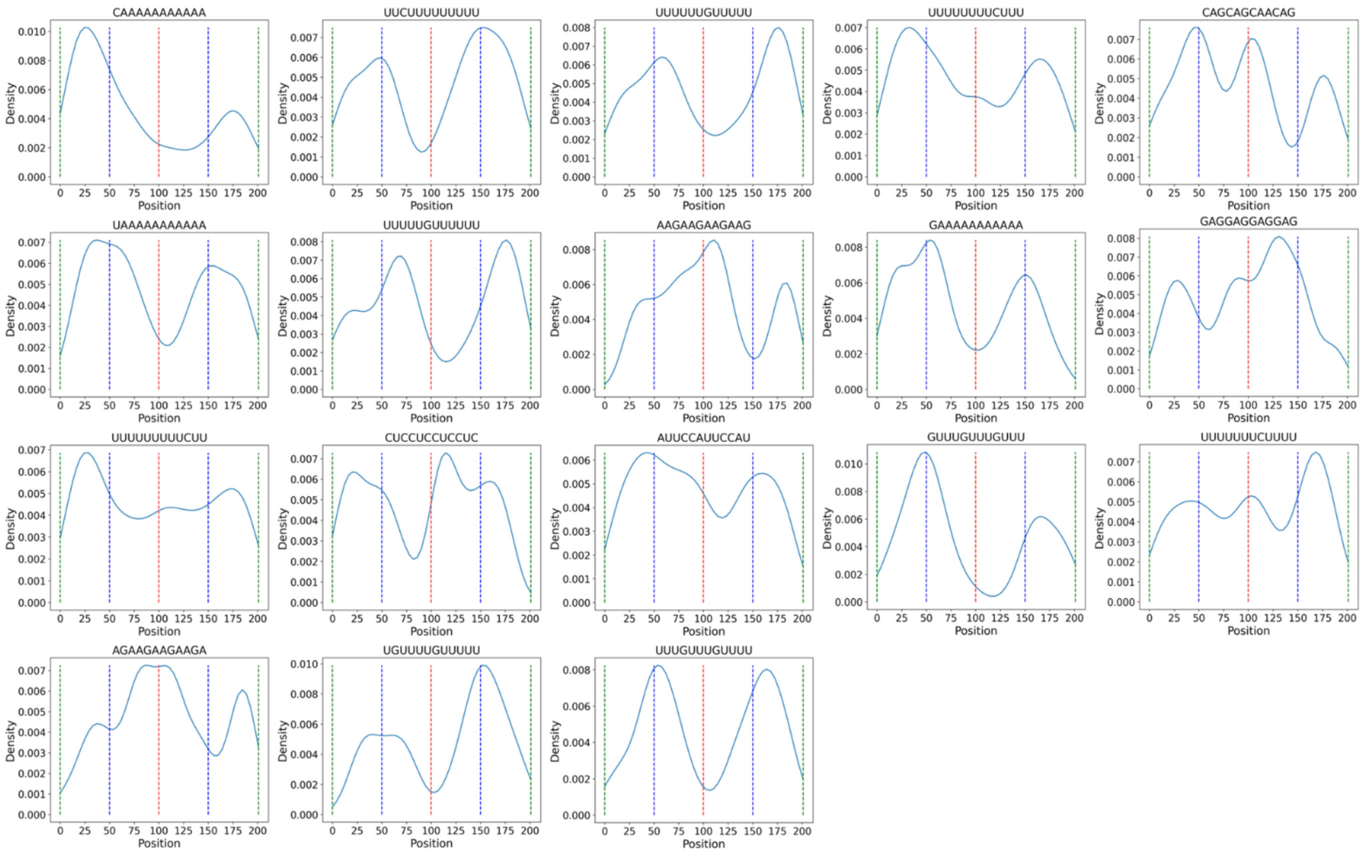


**Figure S7.** 98 motif distribution found by CPBFCN in IGF2BP3 of length 201.

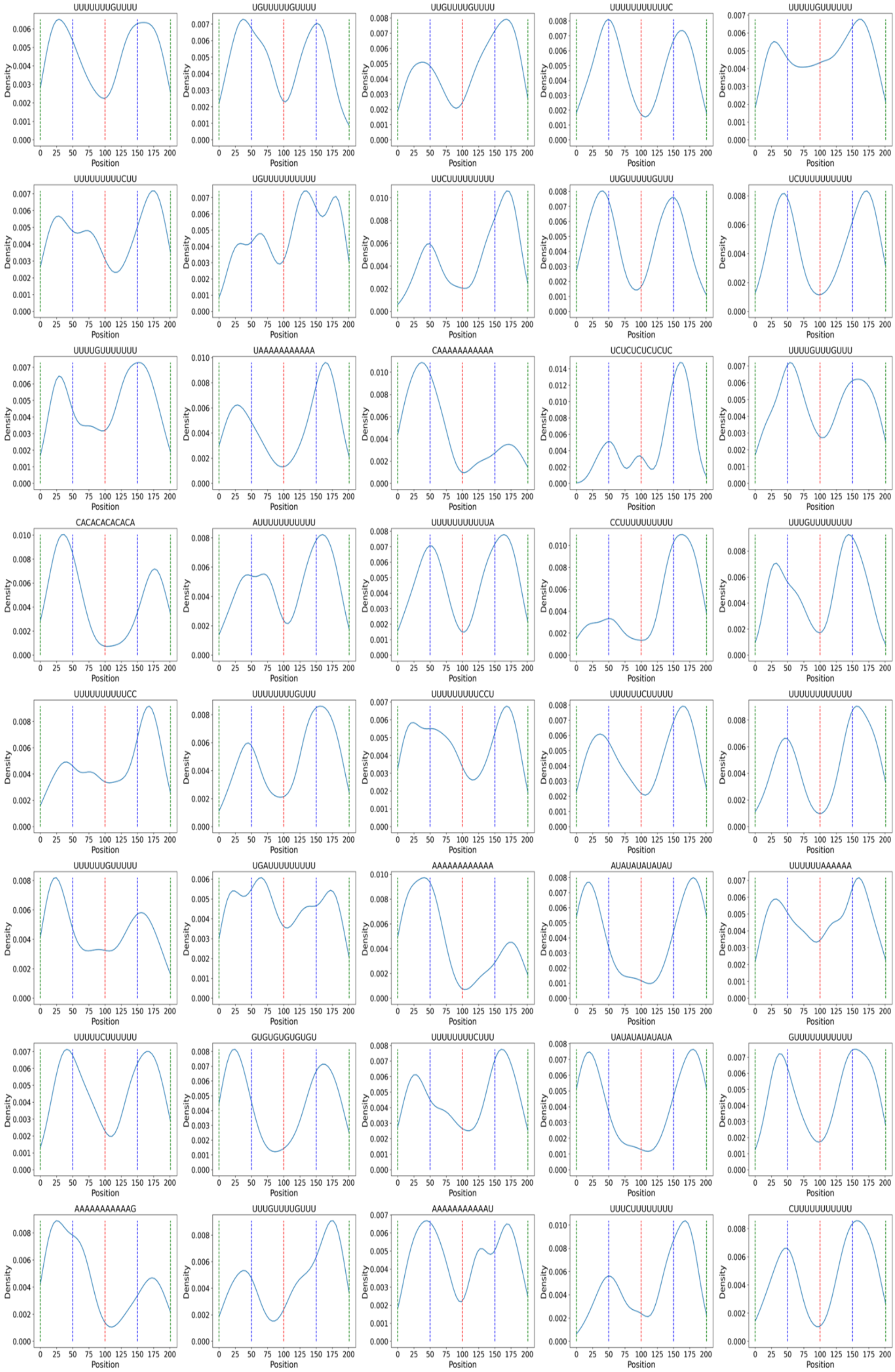
UUUUUUUGUUUU
UGUUUUUGUUUU
UUGUUUUGUUUU
UUUUUUUUUUUC
UUUUUGUUUUUU
UUUUUUUUUCUU
UGUUUUUUUUUU
UUCUUUUUUUUU
UUGUUUUUGUUU
UCUUUUUUUUUU
UUUUGUUUUUUU
UAAAAAAAAAAA
CAAAAAAAAAAA
UCUCUCUCUCUC
UUUUGUUUGUUU
CACACACACACA
AUUUUUUUUUUU
UUUUUUUUUUUA
CCUUUUUUUUUU
UUUGUUUUUUUU
UUUUUUUUUUCC
UUUUUUUUGUUU
UUUUUUUUUCCU
UUUUUUCUUUUU
UUUUUUUUUUUU
UUUUUUGUUUUU
UGAUUUUUUUUU
AAAAAAAAAAAA
AUAUAUAUAUAU
UUUUUUAAAAAA
UUUUUCUUUUUU
GUGUGUGUGUGU
UUUUUUUUCUUU
UAUAUAUAUAUA
GUUUUUUUUUUU
AAAAAAAAAAAG
UUUGUUUUGUUU
AAAAAAAAAAAU
UUUCUUUUUUUU
CUUUUUUUUUUU
Density
Position

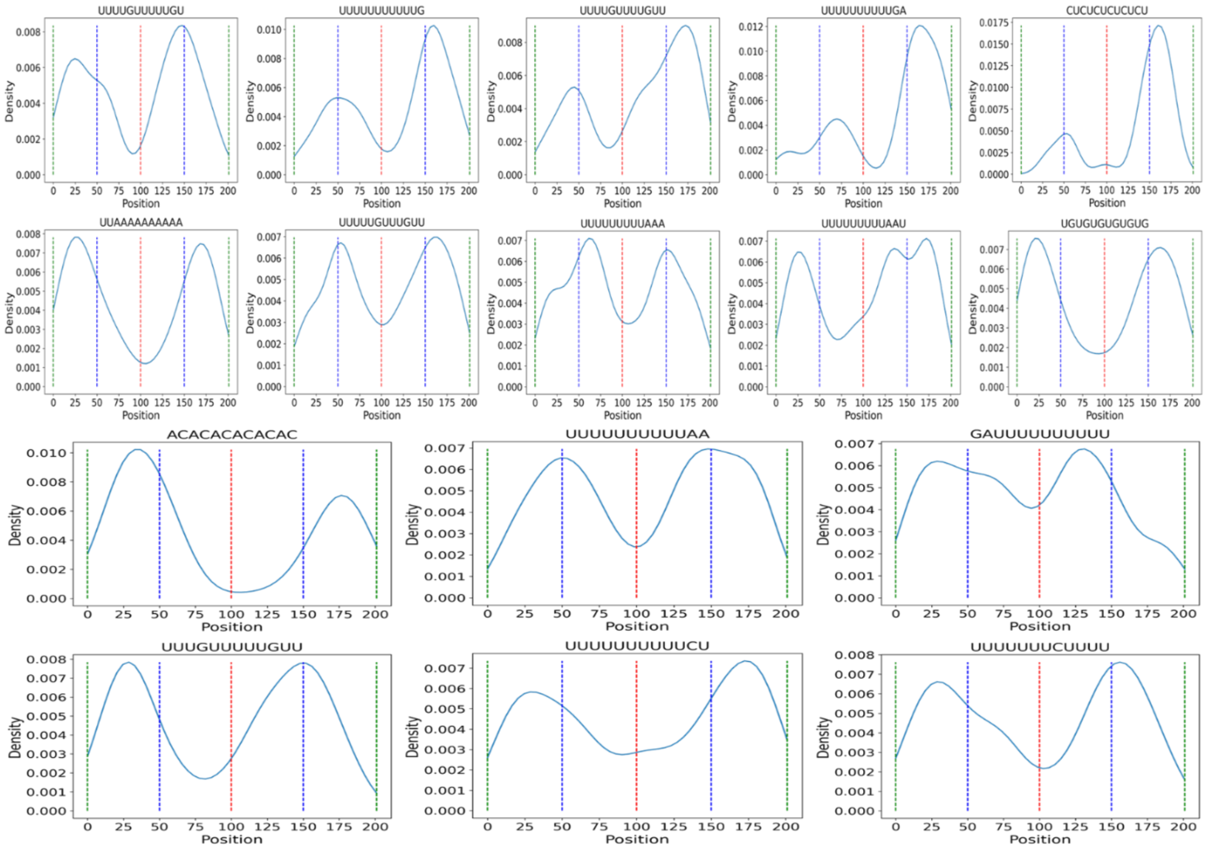


**Figure S8.** 56 motif distribution found by CPBFCN in ZC3H7B of length 201.
